# Self-Assembly Hydrosoluble Coronenes: A Rich Source of Supramolecular Turn-On Fluorogenic Sensing Materials in Aqueous Media

**DOI:** 10.1021/acs.orglett.1c03175

**Published:** 2021-11-09

**Authors:** Daisy C. Romero, Patricia Calvo-Gredilla, José García-Calvo, Alberto Diez-Varga, José Vicente Cuevas, Andrea Revilla-Cuesta, Natalia Busto, Irene Abajo, Gabriel Aullón, Tomás Torroba

**Affiliations:** † Department of Chemistry, Faculty of Science, University of Burgos, 09001 Burgos, Spain; ‡ Institut de Química Teòrica i Computacional (IQTCUB), Universitat de Barcelona, 08028 Barcelona, Spain

## Abstract

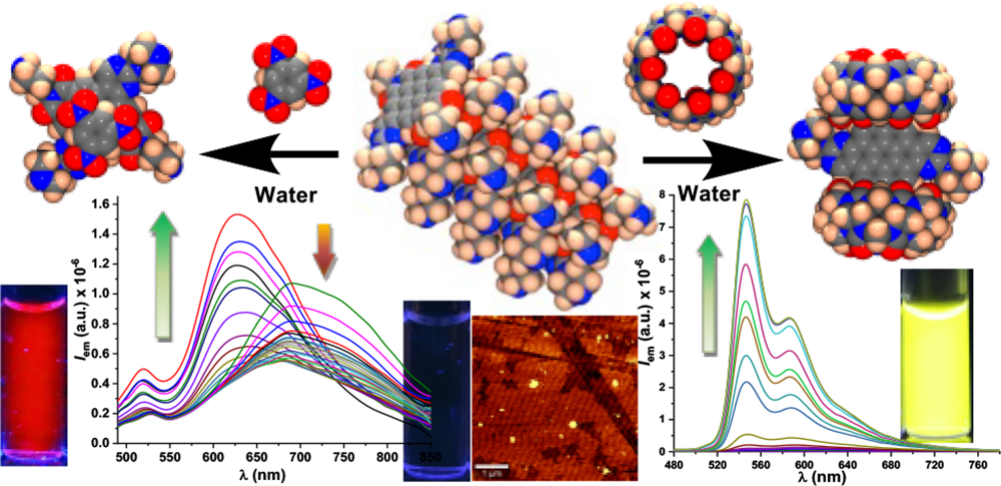

Water-soluble coronenes, that form nanoparticles by self-association, work as new fluorescent materials by complexation with cucurbit[7]uril, as well as selective turn-on fluorogenic sensors for nitroaromatic explosives with remarkable selectivity, by using only water as solvent.

Self-assembling of organic dyes is a rich source of nanomaterials for practical applications.^1^ Aggregation of perylenediimides has been deeply studied and these studies constitute a paradigm in organic nanoaggregation.^2−5^ The closely related coronenediimide derivatives have been studied from a structural point of view^6−9^ or in the preparation of aromatic acceptors for solar cells and high-end electronics.^10,11^ Simpler coronenediimides have been used for the preparation of discotic liquid crystals^12^ or water-soluble dendrimers.^13^ However, the excellent characteristics for π–π stacking or self-assembling make coronenediimides the best choice for molecular recognition studies. We are interested in the preparation of sensing devices for the detection of explosives or toxins in water or vapor.^14−16^ To prepare useful new fluorescent nanomaterials, we have designed an easy route to coronenediimide derivatives having an extended aromatic core surrounded by a hydrophilic periphery, suitable for applications as sensing materials. In this paper, we want to introduce their synthesis and self-assembling characteristics in comparison to related hemicoronene- or perylenediimides. Their unique applications as discrete nanoparticles to supramolecular turn-on fluorescent recognition and sensing of nitroaromatic explosives in water will be presented.

Our synthesis started by the Suzuki reaction of the dibromoperylenediimide **1** with two equivalents of a *N*-Boc protected piperazinyl-pyrimidine boronic ester **2** in conditions used for related Suzuki reactions (Figure 1).^15,17^ The bis-substituted, four *N*-Boc protected derivative **3** (85% yield) was obtained after workup of the reaction. Irradiation of **3** in dichoromethane (DCM) under visible light (halogen lamp, 50W, 4 cm distance) and air for 7 h gave the *N*-Boc protected coronene **4** (89% yield). Traces of an unexpected intermediate of cyclization were also detected. The monocyclized intermediate product **5** (76%) was subsequently obtained as the main product under a shorter irradiation time (3 h). *N*-Boc deprotection of all compounds by treatment with trifluoroacetic acid (TFA) in DCM for 20 min quantitatively gave the unprotected compounds **6**, **7**, and **8**, bearing four secondary amine groups on the periphery (Figure 1). Extended hemicoronediimides from monosubstituted perylenediimides have shown interest as semiconducting materials.^18−20^

**Figure 1:**
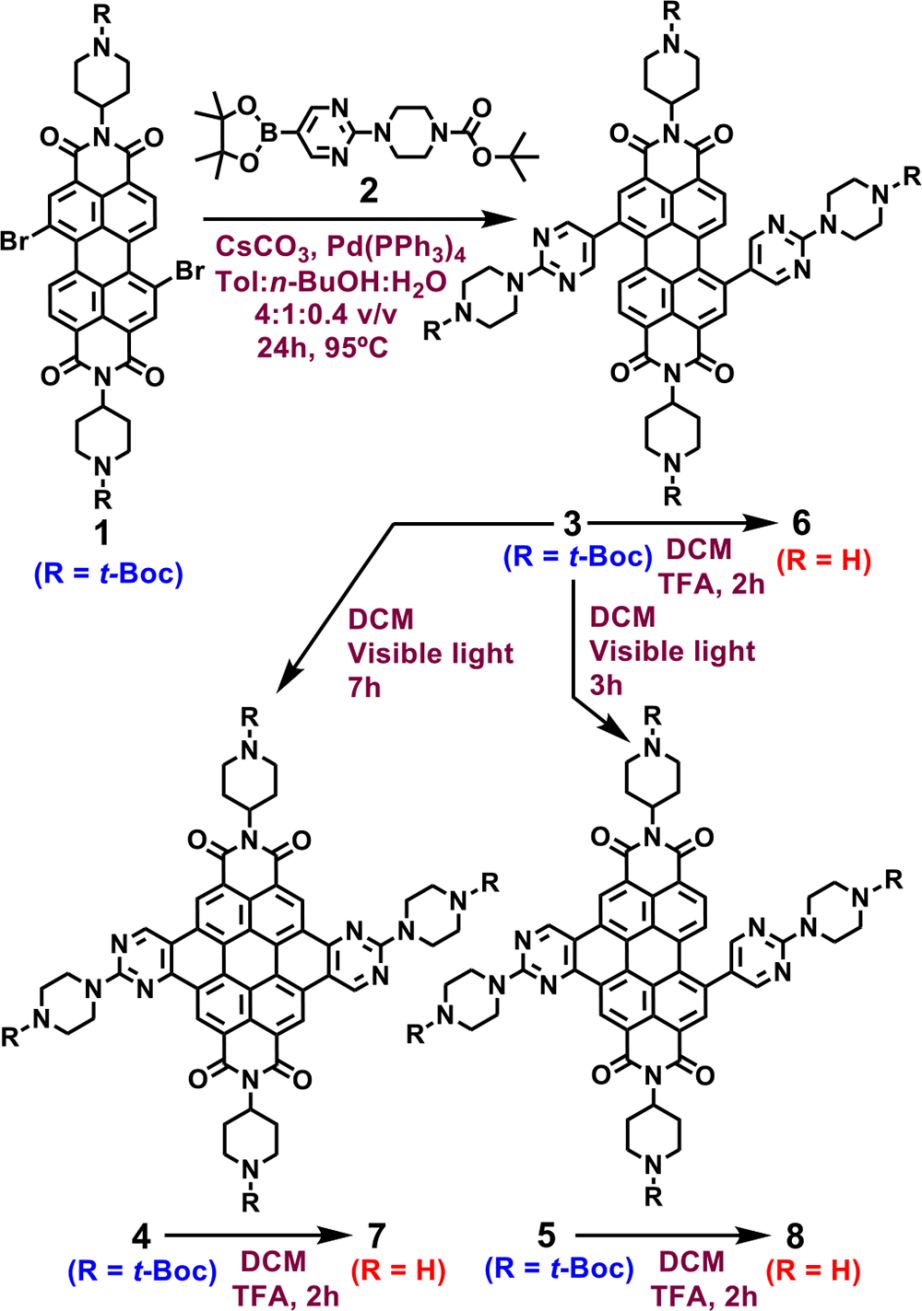
Synthesis of hemicoronene- and coronenediimides.

After study of the physicochemical characteristics of the obtained compounds, we realized that the *N*-Boc protected compounds **3**, **4**, and **5** were bright fluorescent compounds, soluble in common organic solvents, that showed high quantum yields (0.3–0.6 in DCM or CHCl_3_) and lifetimes similar to the starting material **1** (3–10 ns) (Figures S20, S24, and S29). However, the unprotected compounds **6**, **7**, and **8** were almost non fluorescent compounds, soluble in water but almost insoluble in most organic solvents (Figures S35, S54, and S95). As an exception, **6** initially gave a nonfluorescent solution in water, but the water solution became brightly fluorescent after 24 h. The kinetic study showed the appearance and continuous growth of a fluorescent band at 502 nm and a shoulder at 520 nm after dissolving **6** in water (Figure S38). Then **6**, **7** and **8** showed the presence of stable spherical nanoparticles in AFM by deposition of 2 μL of aqueous solutions of samples on mica sheets and evaporation of water (tapping mode, force constant 2.8 N m^–1^, room temperature, scan rate 1–2 lines per second) (Figure 2). Because of the large ability of these compounds to self-associate in water, NMR spectra were not sufficiently informative, especially for **7**; therefore, we prepared a derivative of **7** with long hydrophilic tails that prevented self-association, **9** (Figure 2), which permitted a full characterization by spectroscopy, confirming the structures. The water-soluble nanoparticles from **6**, **7**, and **8**, were also characterized by DLS (Figures S40–S44, S59–S60, and S98–S101).

**Figure 2:**
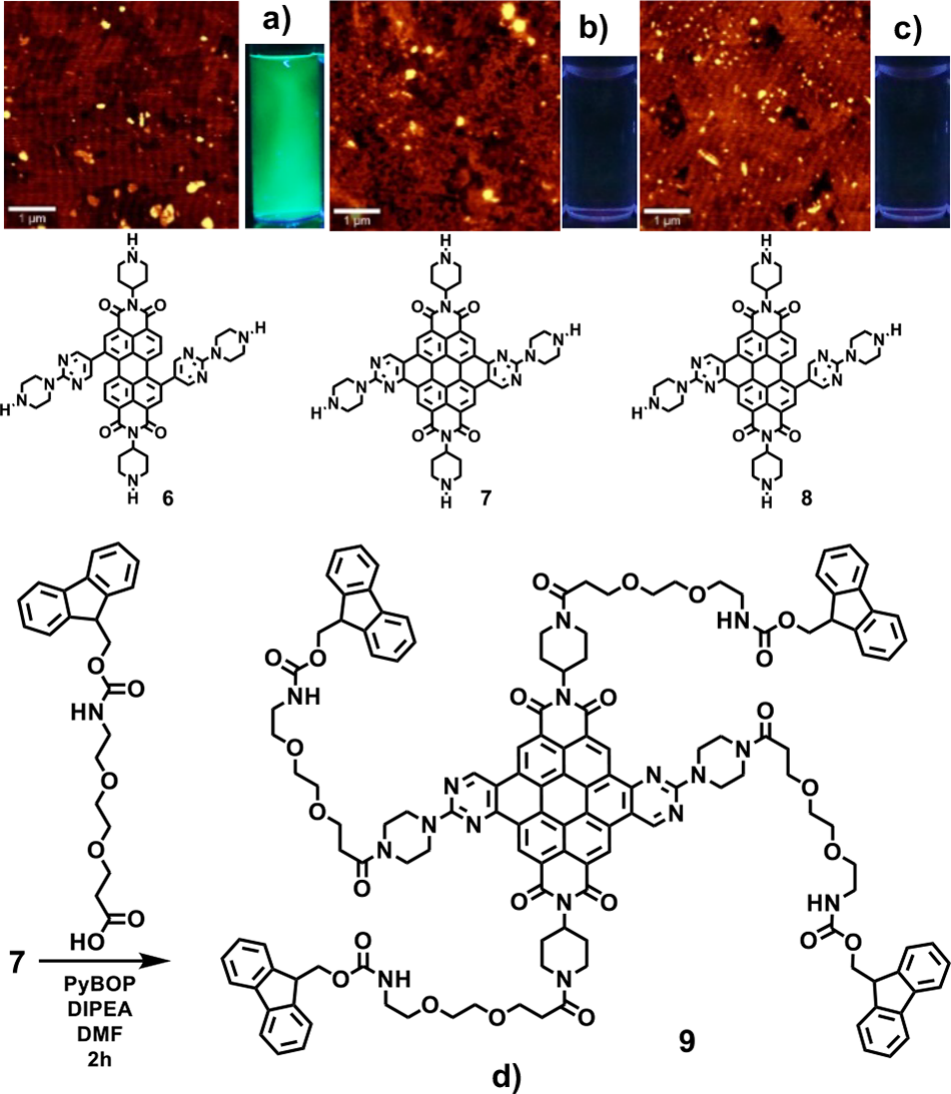
AFM images of samples deposited on mica sheets from water solutions 0.1 μg/mL: (a) **6** (b) **7** (c) **8**. (d) Derivatization of coronene **7** to **9**.

Low molecular weight organic nanoparticles in water constitute an excellent material for studies of aggregation/disaggregation linked to fluorescence variations on the way to new supramolecular sensing devices. Therefore, we studied in deep disaggregation mechanisms based on molecular recognition. The piperidine/piperazine groups on the periphery of the compounds are expected to be good guests for host–guest chemistry of cucurbiturils in water.^21,22^ Cucurbiturils have been used for the preparation of supramolecular luminescent sensors^23^ or for enhancing fluorescence of perylenediimides in water.^24^ Therefore, we performed several tests by adding aqueous solutions of cucurbit[n]urils, CB[5], CB[6], CB[7], and CB[8], in 1:1, 1:5, 1:10, and 1:20 dye/cucurbituril molar proportions to 10 μM aqueous solutions of **6**, **7**, and **8**. Except for **6**, compounds **7** and **8** showed a neat increase of fluorescence in the presence of CB[7] (Figures S45, S61, and S102). Then we performed fluorescent titrations of 10 μM solutions of **7** and **8** in water by adding increasing amounts of concentrated solutions of CB[7] (Figure 3).

**Figure 3:**
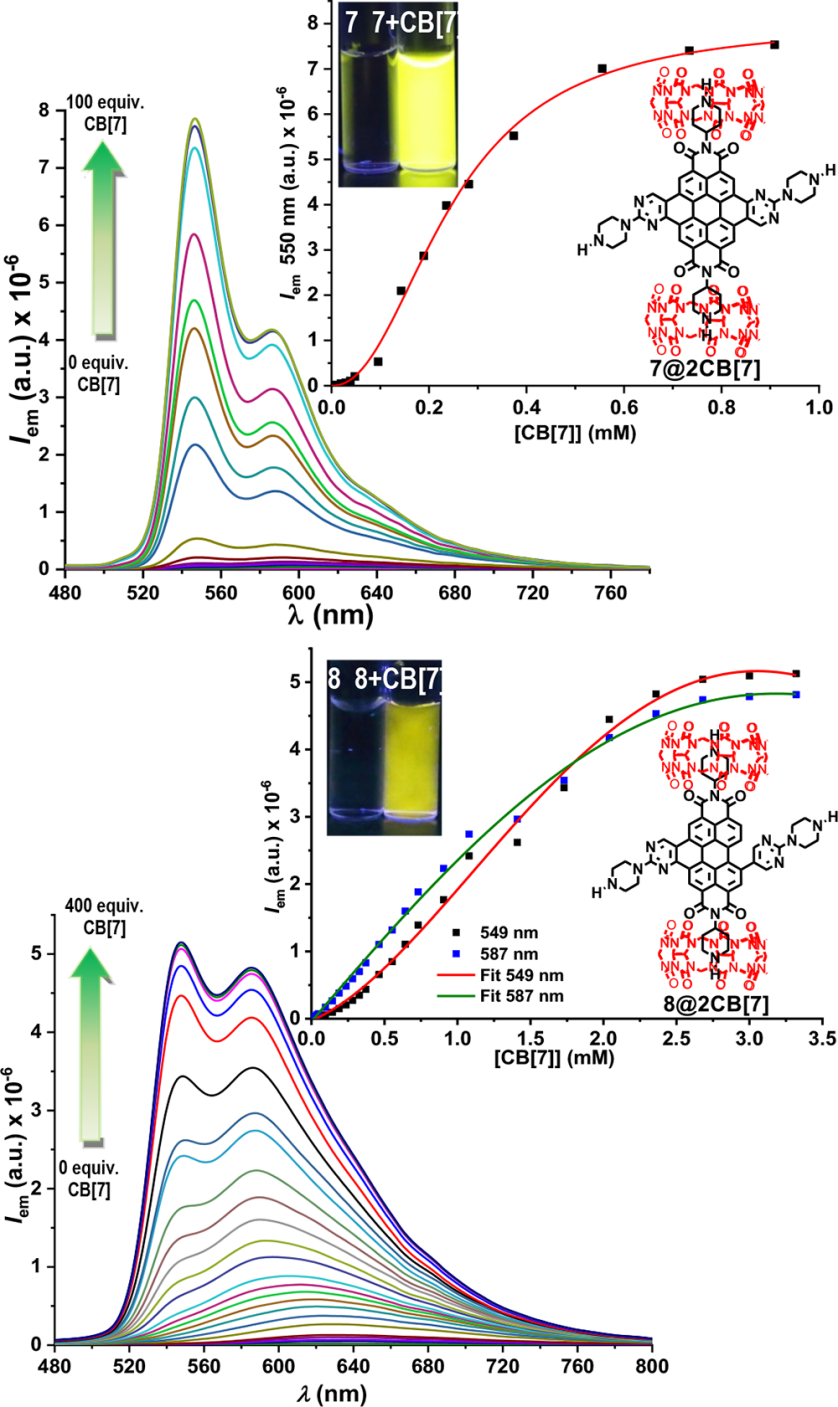
Fluorescent titration curves and titration profiles of 10 μM solutions of **7** and **8** in water with increasing amounts of CB[7], 0 to 100 equiv CB[7] for titration of **7** and 0 to 400 equiv CB[7] for titration of **8**. Inset: solution samples of **7** and **8** under UV light, 365 nm, before and after titrations with CB[7].

The fluorescent titration curves and titration profiles of 10 μM solutions of **7** and **8** in water, with CB[7], showed asymptotic increases of fluorescence when a large excess of CB[7] was added (Figure 3). Additional UV–vis absorption titrations are shown in Figures S62 and S109. In this way, **7** and **8** solutions in water became brightly fluorescent in the presence of a large excess of CB[7], expanding the narrow range of fluorescent hemicoronene/coronenediimides in water solutions. CB[7] is expected to host small molecular guests;^25,26^ therefore, in this series, the complexation with CB[7] should happen by the piperidine/piperazine groups,^26^ giving rise to disaggregation to form individual complexes in solution. The disaggregation effect worked in the opposite way in the case of **6**, where the presence of excess CB[5], CB[6], or CB[7] in 10 μM water solutions of **6** hindered the development of fluorescence (Figures S45 and S46). The type of complex formed between CB[7] and **7** or **8** was studied by Job’s plot experiments and isothermal titration calorimetry (ITC) measurements, but the results were inconclusive. Instead, accurate mass spectrometry measurements afforded high resolution *m*/*z* peaks of the lowest terms in the series of expected complexes, **7**@CB[7] (*m*/*z* 2040.7227) and **8**@CB[7] (*m*/*z* 2043.7303), a low resolution peak of the complex **7**@2CB[7] (*m*/*z* 3208.1), and self-associated compounds such as [**7**]_2_ (*m*/*z* 1757.6), [**7**]_3_ (*m*/*z* 2634.9), and [**7**]_4_ (*m*/*z* 3515.9) (Figures S51–S53 and S92–S94). With these results, we looked for new applications of the aggregation/disaggregation mechanism that could afford light on the mechanism as well as new sound applications in sensing. In the initial tests, **7** or **8** were not sensitive to common cations, anions, acids or oxidants in water, but **7** showed a dramatic appearance of red fluorescence in the presence of 1,3,5-trinitrobenzene (TNB), a common explosive. Consequently, the study was extended to trinitrotoluene (TNT), a commonly used explosive that is usually detected by quenching of fluorescence from suitable fluorophores^27−32^ with very few exceptions.^33−35^ For this reason, TNT lacks a turn-on fluorogenic method for its detection in water with practical use. Taking into account the large number of sunken warfare materials still existing in the oceans from the Word Wars I and II,^36^ the design of new fluorogenic sensing materials for the detection of TNB/TNT in water is worthy of study. Titration of a 10 μM solution of **7** in water with TNB, 0 to 6 mg/mL TNB, showed a decrease in a 690 nm far red band and an increase of a 632 nm red band in fluorescence with the addition of increasing amounts of TNB. Analogously, titration of a 10 μM solution of **7** in water with TNT, 0 to 1.77 mg/mL TNT, also showed a decrease in the 700 nm band and an increase of a 595 nm band in fluorescence with the addition of increasing amounts of TNT (Figure 4). The fluorescent titration curves and ratiometric titration profiles of 10 μM solutions in water of **7** with increasing amounts of TNB and TNT are shown in Figure 4. In addition, the lifetime decay changed from an initial value of 4.95 to 4.62 ns after addition of TNB or TNT to solutions of **7** in water, but there were no significant changes in the absorbance values under the same conditions. The evolution of changes seems to be correlated to selective disaggregation processes, which produced ratiometric changes in fluorescence, with increases of the final red fluorescence from almost 0 to 2/3%. From the titration plots in every case, we calculated the LODs by IUPAC-consistent methods.^37^ From the standard deviation equation [LOD = 3.3 × SD/s], LOD = 3.3 × 0.00583/0.894 = 0.02 μM for TNB and LOD = 3.3 × 1.056/59 = 0.06 μM for TNT, comparable to LODs from previous reports,^33,34^ but in this case with the unprecedented feature of using only water in the titration. We also calculated the LODs within the values measured (different than 0) by adjusting the initial values to a mean square linear regression and using the R program (SI page S114), which can be considered as a good approach to the limits of quantification of the system. By this way, measured values were LOQ = 0.046 μM for TNB and LOQ = 0.21 μM for TNT. With a solubility of 100 mg/L for TNT (0.44 mM) (SI, page S115) in pure water or seawater at 20 °C, the LOQs found are suitable for the detection of traces of TNT in environmental aquatic samples by a turn-on fluorescence mechanism. The system can also be applied to TNB, which is roughly as three-times more soluble in water/seawater than TNT (SI, page S115). Indeed, **7** was not sensitive to the presence of common cations, anions, or usual interferents found in water (Figures S72–S88); therefore, its presence did not interfere the TNB/TNT detection.

**Figure 4:**
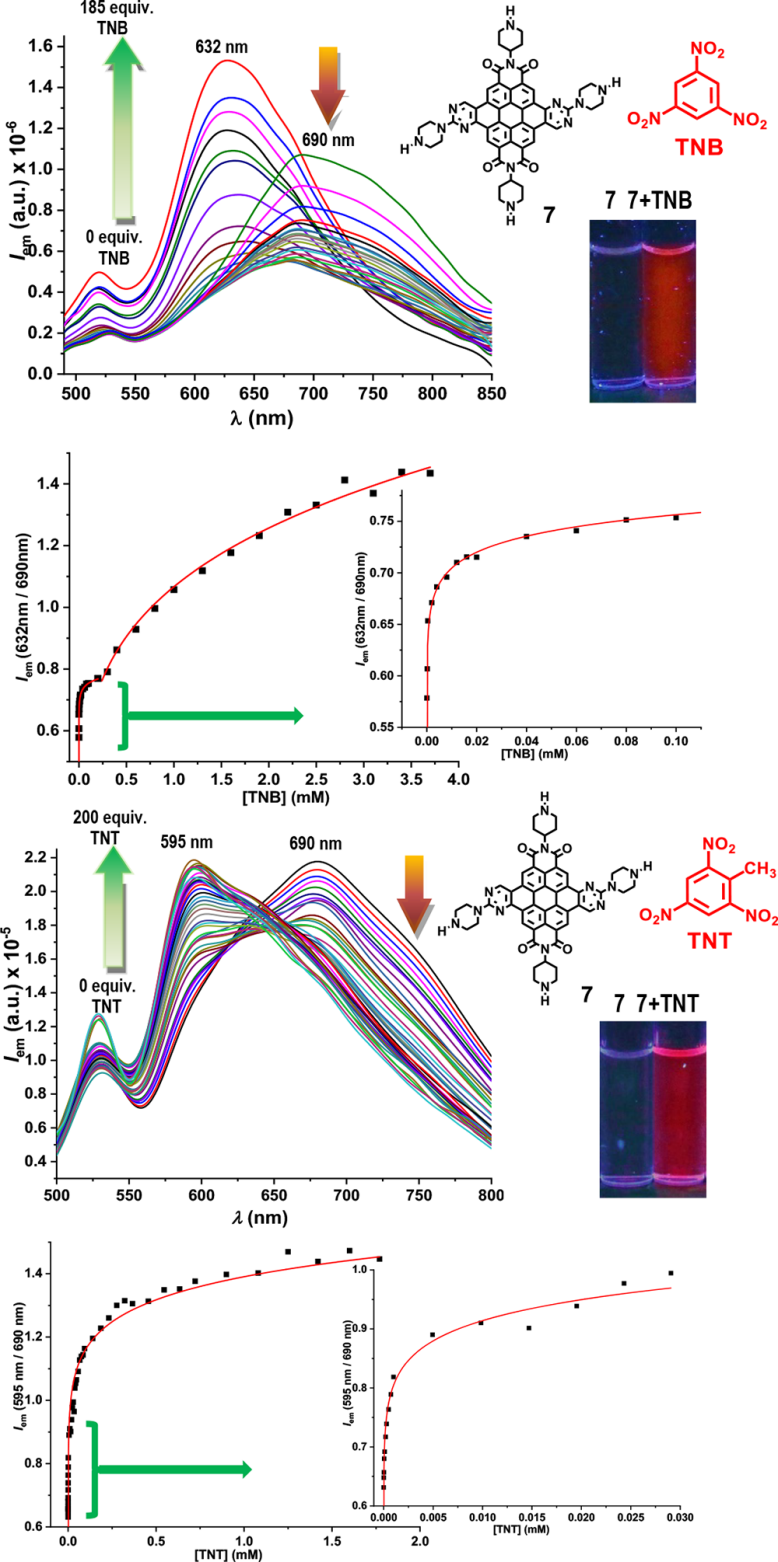
Fluorescent titration curves and ratiometric titration profiles of 10 μM solutions in water of **7** with increasing amounts of TNB and TNT, with expansions of the first parts of the titration plots. Insets: water solution samples of **7** under UV light, 365 nm, before and after titration with TNB/TNT.

Attempts of determining binding constants from the fluorescence titration experiments gave the best fitting from the titration of **7** and TNT for a 1:2 model (supramolecular.org), being *K*_1_ = (2.3 ± 1.3) × 10^5^ M^–1^ and *K*_2_ = (3.4 ± 1.0) × 10^3^ M^–1^ the apparent formation constants of the **7**/TNT (1:1) and **7**/(TNT)_2_ (1:2) complexes, respectively. Because of the large uncertainties, they have only a qualitative value, indicating that multiple association accounted for the disaggregation of **7** in the presence of TNT. Aggregation of **7** was studied by ITC measurements (Figure S89). The binding isotherm was fitted by a dimer dissociation model (NanoAnalyze Software, TA Instruments). We obtained thermodynamic parameters, *K*_agg_ = (1.39 ± 0.4) × 10^4^ M^–1^, Δ*H* = −66 ± 2 kJ mol^–1^, and Δ*S* = −142 ± 9 J mol^–1^ K^–1^, that agreed with the aggregation process.

To understand the interactions, we performed theoretical DFT calculations (SI, p S76) of the complexes between two self-aggregated **7**, and then **7** with two CB[7] or one TNB (Figure 5).

**Figure 5:**
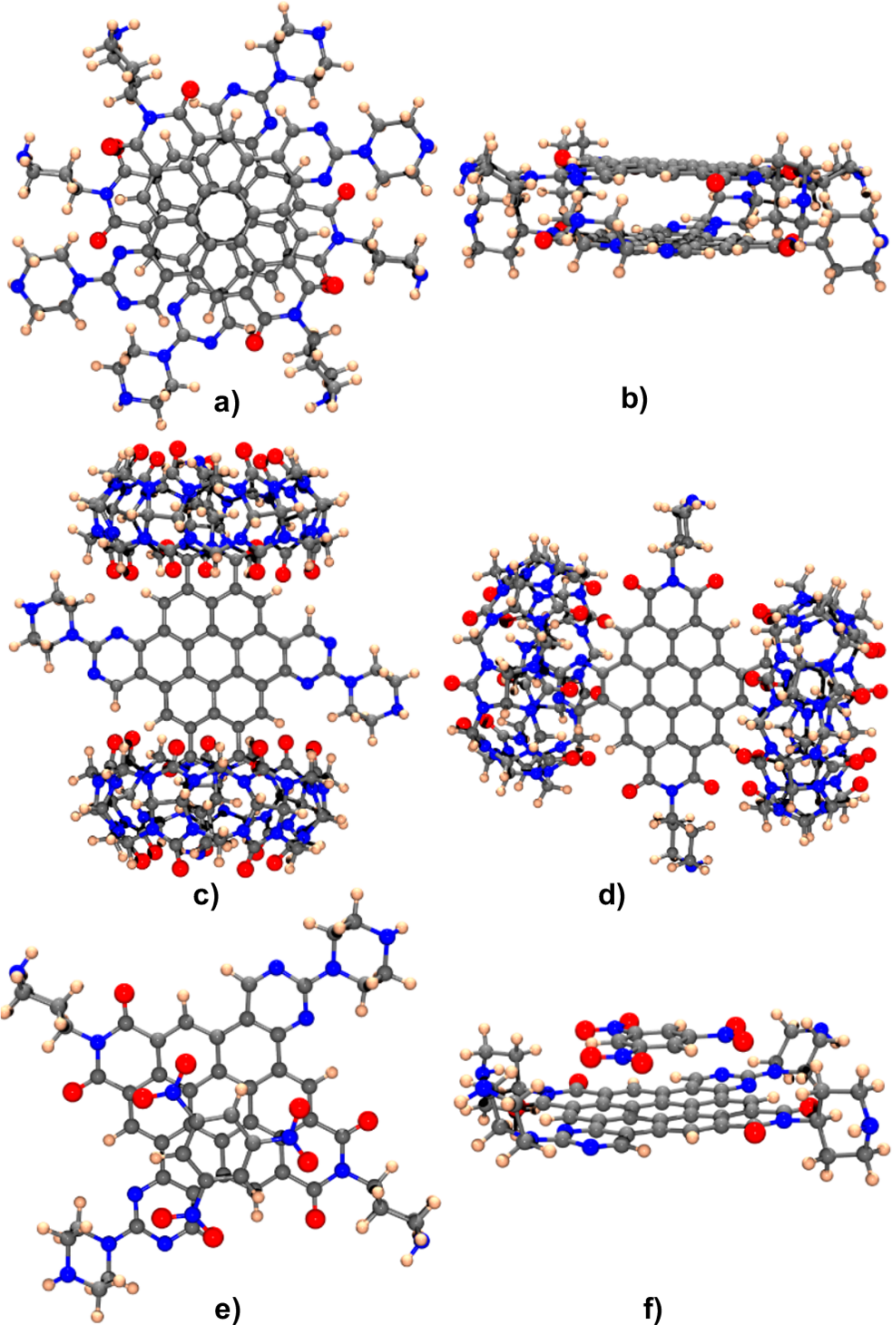
(a) Calculated structure of dimer [**7**]_2_ seen from a parallel plane to the structure and (b) from a perpendicular plane. (c) Calculated structure of complex **7**@2(*apical*)CB[7], relative energy (0 kcal/mol), and (d) **7**@2(*equatorial*)CB[7], relative energy (25.16 kcal/mol). (e) Calculated structure of complex **7**·TNB seen from a parallel plane to the aromatic structure or (f) from a perpendicular plane.

The results showed that dimer [**7**]_2_ had stabilization energy of −52.24 kcal/mol with respect to the two isolated molecules (Figure 5a,b). The most stable calculated structure for the complex **7**@2CB[7] was **7**@2(*apical*)CB[7] (Figure 5c), followed by **7**@2(*equatorial*)CB[7] (Figure 5d), less stable by 25.16 kcal/mol. The interaction of **7**·CB[7] was theoretically modeled, finding that for 1:1 complexes the entry through the apical position had a calculated complexation energy [CCE] = −44.59 kcal/mol, while the interaction through the equatorial position displayed [CCE] = −39.71 kcal/mol. The entry of a second CB[7] in complexes 1:2 had [CCE] = −34.26 kcal/mol for **7**@2(*apical*)CB[7] and [CCE] = −28.36 kcal/mol for **7**@2(*equatorial*)CB[7], indicating an apical preferred interaction. The [**7**·TNB] complex was modeled by calculating the complexation energy using the counterpoise correction (SI, p S66) (Figure 5e,f), giving [CCE] = −18.17 kcal/mol.

In conclusion, we have prepared a new series of perylene-, hemicoronene-, and coronene diimides that were soluble in water, giving nanoparticles by self-association, which in turn worked as new fluorescent materials by self-aggregation or complexation with cucurbit[7]uril, as well as selective turn-on fluorogenic sensors for explosive trinitroaromatic compounds, with remarkable selectivity, by using only water as solvent.

## Supporting Information Available

Compound structures (XYZ)
Synthetic procedures, complete characterization of all compounds, additional experiments, theoretical calculations (PDF)

## Supplementary Material

ol1c03175_si_001.xyz
