# Supplementary material for: Self-Assembly Hydrosoluble Coronenes: A Rich Source of Supramolecular Turn-On Fluorogenic Sensing Materials in Aqueous Media
Source: Org Lett. 2021 Nov 9;23(22):8727–32. doi: 10.1021/acs.orglett.1c03175 (PMC8609571; doi:10.1021/acs.orglett.1c03175)
Supplement: Supplementary file 2 — ol1c03175_si_002.pdf [file ol1c03175_si_002.pdf]

SUPPLEMENTARY INFORMATION

Self-Assembly Hydrosoluble Coronenes: A Rich Source of Supramolecular Turn-On Fluorogenic Sensing Materials in Aqueous Media.

Daisy C. Romero,[a] Patricia Calvo-Gredilla,[a] José García-Calvo,[a] Alberto Diez-Varga,[a] José Vicente Cuevas,[a] Andrea Revilla-Cuesta,[a] Natalia Busto,[a] Irene Abajo,[a] Gabriel Aullón[b] and Tomás Torroba*[a]

a. Departamento de Química, Facultad de Ciencias, Universidad de Burgos, 09001 Burgos, Spain. E-mail: ttorroba@ubu.es
b. Institut de Química Teòrica i Computacional (IQTCUB). Universitat de Barcelona, 08028 Barcelona, Spain

INDEX:

General Methods……………………………………………………………………….…….…….....S02

Synthesis and characterization of compounds..……………………………………………….…………S04

Further characterization studies……………………………………………………….…….…….….S68

Theoretical calculations………………………………………………………………………….…….S76

Binding constants from fluorescence titration experiments…………………………….…...…..….S114

The use of R program..……………………………………………………………………....………S114

Solubility of TNT or TNB in water.…………………………………………………….....………S115

General methods: Melting points were determined on an electrothermal melting point Gallenkamp apparatus and are uncorrected. Infrared Spectra were recorded with the potassium bromide pellet method, with a JASCO FT/IR-4200 spectrometer. Nuclear magnetic resonance (NMR) spectra were recorded with Varian Mercury-300 and Varian Unity Inova-400 spectrometers at room temperature (25ºC), with $CDCl_3$, CD_3CN and CD_3OD as solvents. Chemical shifts (δ) were reported in parts per million (*ppm*) relative to the residual solvent peaks, rounded to the nearest 0.01 for ^{1}H-NMR and 0.1 for ^{13}C-NMR. Spin-spin coupling constants (*J*) in ^{1}H-NMR were given in Hz, rounded to the nearest 0.1 Hz. Peak multiplicity was indicated as follows: *s* (singlet), *d* (doublet), *t* (triplet), *q* (quartet), *m* (multiplet) and *br* (broad). Relative integrals were given in ^{1}H-NMR too. All ^{13}C NMR were recordered with complete proton decoupling. Carbon types, structure assignments and attribution of peaks were determined from ^{13}C-DEPT-NMR and, in some cases, two dimensional correlation experiments (HMQC (^{1}H-^{13}C), COSY (^{1}H-^{1}H) and HMBC (^{1}H-^{13}C). NMR spectra were analyzed using MestReNova NMR data processing software. High High-resolution mass spectra were acquired with a Micromass AutoSpec instrument, by electronic impact (70 eV). MALDI-TOF mass spectra were measured with a MALDI-TOF Bruker Autoflex Mass Spectrometry instrument, using DCTB (*trans*-2-[3-(4-tert-butylphenyl)-2-methyl-2-propenylidene])malononitrile) or DIT (dithranol) as matrixes, in modes positive or negative. The atomic mass of the molecular ion (and/or fragments) per elementary charge were reported in dimensionless quantities. Absorption spectra were acquired with a Varian Cary Eclipse or a Hitachi U-3900 spectrometers, in one centimetre quartz cells at 25ºC. Emission spectra were recorded with a Hitachi F-7000 FL or a modular Edinburgh Instruments FLS980 spectrofluorometers, in one centimetre quartz cells at 25ºC. Solvatochromism Tests: All samples were freshly prepared in a concentration of 10 µM in each different solvent. Photos were taken with a Canon (EOS M3) camera and a lens of 22 mm. Absorption spectra were acquired with a Varian Cary Eclipse or a Hitachi U-3900 spectrometers, in one centimetre quartz cells at 25ºC. Emission spectra were recorded with a in a Varian Cary Eclipse or a Hitachi F-7000 FL spectrofluorometers, in one centimetre quartz cells at 25ºC, exciting at corresponding wavelength, indicated below. The solvents were, if it is not said otherwise: 1: H_2O, 2: MeOH (methanol), 3: DMSO (dimethylsulfoxide), 4: DMF (*N,N'*-dimethylformamide), 5: MeCN (acetonitrile), 6: Acetone, 7: EtOAc (ethyl acetate), 8: THF (tetrahydrofuran), 9: $CHCl_3$, 10: CH_2Cl_2 (dichloromethane), 11: Toluene, 12: Et_2O (diethyl ether), 13: *n*-Hx (hexane), 14: *c*-Hx (cyclohexane). Fluorescence Lifetime Decays: Chromophere solutions (or mixtures of chromophere with an additive) were freshly prepared in a concentration of 1-10 µM in the corresponding solvent. The decay was fitted (black) with a sum of two exponentials by convolution with the instrumental response function (red). The quality of the fit was judged by χ2 values and the plot of the weighted residues. Measurements were made with a modular spectrometer Edinburgh Instruments FLS980, with a source excitation pulsed lasers of picosecond diode: 366-380 nm, 398-410 nm, 437-446 nm, 470-478 nm, 505-515 nm and 635 nm. One centimetre quartz cells, at 25ºC, were employed. Quantum Yields: Fresh solutions made from high-purity dyes (or mixtures of dye with an additive) and solvent were used. Integrating sphere was used as the method and three measurements were performed for each sample in order to calculate the average. Solutions were prepared in a concentration of 1-10 µM in the corresponding solvent. The excitation wavelength is indicated in each case. Measurements were made with a modular spectrometer Edinburgh Instruments FLS980. One centimetre quartz cells, at 25ºC, were employed. Dynamic Light Scattering (DLS) studies were

performed with a Malvern Zetasizer Ultra machine, in 1 cm cuvettes. Morphologies (AFM imaging): All compounds were dissolved in a corresponding solvent or in a mixture of them, at different concentrations. Solutions were freshly prepared, if it is not said otherwise. Then 2 µl of these solutions were transferred to a mica surface and dried at room temperature. Surface topography was studied with an Alpha300A AFM Witec., in tapping mode (force constant of 2.8 $N\cdot m^{-1}$) under ambient conditions using moderate scan rates (1–2 lines per second). Topography and phase images were recorded simultaneously, with a resolution of 512 x 512 pixels. Images were processed and analyzed by using WITec Project Four 4.1 Software. Microwave assisted reactions were performed in a Biotage Initiator EXP EU Microwave System. Temperature and reaction time were fixed and power oscillates to reach it. The temperature was monitored by an IR sensor on the outer surface of the reaction vessel. Reactions were carried out in 10 or 20 mL sealed glass vials. Qualitative tests: (a) Cucurbituril: Dyes were dissolved in the corresponding solvent (or mixture of them), indicated below, in a concentration of 10 µM. Then, aqueous solutions of CB[5], CB[6], CB[7] and CB[8] were added in a molar proportion of 1:1, 1:5, 1:10 and 1:20 (dye:cucurbituril). Photos were taken under white and 366 nm lights. The test is *positive* when any change is observed; it is *negative* in the other case. (b) Cations, anions and oxidizing and reducing agents: Analytes were dissolved in the corresponding solvent (or mixture of them), indicated below, in a concentration of 10 µM. In some cases, an additive was added (it is reported in the data). Then, different solutions of cations, anions and oxidizing or reducing agents, dissolved in the appropriate solvent, were added in a molar proportion of 1:1, 1:5, 1:10 and 1:20 (compound:analyte). Photos were taken under white and 366 nm lights. The test is *positive* when any change is observed; it is *negative* in the other case. Analytes tested, if it is not said otherwise, were: Cations (in water): Ag^+ ($AgClO_4\times H_2O$), Ni^{2+} ($Ni(ClO_4)_2\times 6H_2O$), Sn^{2+} ($Sn(OTf)_2$), Cd^{2+} ($Cd(ClO_4)_2$), Zn^{2+} ($Zn(OTf)_2$), Pb^{2+} ($Pb(ClO_4)_2$), Cu^{2+} ($Cu(ClO_4)_2\times 6H_2O$), Fe^{3+} ($Fe(ClO_4)_3\times H_2O$), Sc^{3+} ($Sc(OTf)_3$), Al^{3+} ($Al(ClO_4)_3\times 9H_2O$), Hg^{2+} ($Hg(ClO_4)_2\times H_2O$), Au^{2+} ($HAuCl_4\times 3H_2O$), $MeHg^+$ (MeHgCl). Anions (in water): Cl^- (TBACl), F^- (TBAF),Br^- (TBABr), I^- (TBAI), BzO^- (TBA(BzO)), NO_3^- ($TBA(NO_3)$), $H_2PO_4^-$ ($TBA(H_2PO_4)$), HSO_4^- ($TBA(HSO_4)$), AcO^- (TBA(AcO)), CN^- (TBA(CN)), SCN^- (TBA(SCN)). Oxidizing agents: oxone, benzoyl peroxide, H_2O_2 and *m*-CPBA, in H_2O; TNB and TNT in methanol. Test of pH effect: Dyes were dissolved in the corresponding solvent (or mixture of them) indicated below, in a concentration of 10 µM. Then, 10 µl of this solution were added to 240 µl of each buffer solution. These solutions were (pH (salt, concentration): 5.4 (NaH_2PO_4, 5 mM), 5.7 (NaH_2PO_4, 5 mM), 6.8 (HEPES,5 mM), 6.9 (NaH_2PO_4, 5 mM), 7.1 (NaH_2PO_4, 5 mM), 7.1 (HEPES, 5 mM), 7.3 (NaH_2PO_4, 5 mM), 7.4 (NaH_2PO_4, 5 mM), 7.5 (HEPES, 5 mM), 8.0 (HEPES, 5 mM), 9.1 (NaH_2PO_4, 5 mM) and 10.5 (HEPES, 5 mM). pH of the resulting solution was regulated with aqueous NaOH (5%) and HCl (1 M). Photos were taken under white and 366 nm lights. Kinetic effect studies: Dyes were dissolved in the corresponding solvent (or mixture of them), indicated below, in a concentration of 10 µM. Absorbance and luminescence emission were measured using a Hitachi U-3900 spectrometer and a Hitachi F-7000 FL spectrofluorometer respectively, in one centimetre quartz cells at 25ºC. One spectrum was taken every three minutes. Lambert-Beer studies: In order to choose the optimum work concentration, absorbance and emission intensity of the dye was checked to be linear. Dyes were dissolved in the corresponding solvent (or mixture of them), indicated below, in a concentration range of 0.1-10 µM. Absorbance and emission intensity were measured with a Hitachi U-3900 spectrometer and a Hitachi F-7000 FL spectrofluorometer respectively, in 1 cm quartz cells at 25ºC.

Synthesis and characterization of compounds.

Synthetic procedures:

General procedure for the Suzuki coupling. *N*,*N*'-Bis-(1-(tert-butoxycarbonyl)piperidin-4-yl)-1,7-dibromoperylene-3,4:9,10-tetracarboxylic diimide **1** (0.25 g, 0.27 mmol) was dissolved in toluene:*n*-butanol:water (4:1:0.4) (100 mL) in a dried schlenk flask under inert atmosphere. Then, $Pd(PPh_3)$ (31.2 mg, 0.03 mmol), cesium carbonate (0.27 g, 0.82 mmol) and 2-(4-(*N*-tert-butyl-1-carboxylate)piperazin-1-yl)pyrimidine-5-boronic acid pinacol ester **2** (Frontier Scientific 500068572) (0.22 g, 0.56 mmol) were added. The reaction mixture was stirred under reflux (95 °C) for 24 hours in a hot-plate magnetic stirrer with aluminum adapter. A colour change of the reaction mixture from red to black was observed. The solvent was removed under reduced pressure and the residue was subjected to column chromatography (silica gel, CH_2Cl_2:MeCN, 2:1 to CH_2Cl_2:MeOH, 50:1) to afford the disubstituted perylenediimide **3** as a red solid (297.5 mg, 85%).

General procedure for the photochemical ring-closing reaction. Disubstituted perylenediimide **3** (48.0 mg, 0.04 mmol) in CH_2Cl_2 (58 ml), in a borosilicate flask, was stirred under visible light irradiation (halogen lamp, GE M280/FNV/CG 50W 12V, 50W, GU5.3 2900K, General Electric) (https://staticde.multi-lite.de/shop-b2b/30315_data-sheet-EN.pdf) at 4 cm of distance for seven hours. The solvent was removed under reduced pressure and the residue was subjected to column chromatography (silica gel, CH_2Cl_2:MeCN, 5:1) to afford the coronenediimide **4** (42.5 mg, 89% yield) and traces of hemicoronenediimide **5** (2.5 mg, 5% yield). Similarly, irradiation of **3** (48.0 mg, 0.04 mmol) in CH_2Cl_2 (58 ml), under visible light (halogen lamp, 50W) at 4 cm of distance for three hours afforded **5** (36.5 mg, 76% yield) and **4** (5.5 mg, 11% yield) as purple solids.

General procedure for removal of *N*-Boc protecting groups. Trifluoroacetic acid (210 µl) was added dropwise to a stirred solution of coronenediimide **4** (50.0 mg, 0.039 mmol) in CH_2Cl_2 (2.0 ml). The reaction mixture was stirred for two hours. Then, aqueous NaOH (1 M) was added on the solution until basic pH (8-9). Finally, the mixture was extracted with CH_2Cl_2 (2x50 mL), the combined organic extracts washed with water (50 mL) and dried ($MgSO_4$). Then the solvent was removed under reduced pressure to afford the coronenediimide **7** (34 mg, 99% yield) as a red solid. Similarly, trifluoroacetic acid treatment of perylenediimide **3** (50.0 mg, 0.039 mmol) afforded **6** (34 mg, 99% yield) and hemicoronenediimide **5** (50.0 mg, 0.039 mmol) afforded **8** (34 mg, 99% yield), all obtained as red solids.

Characterization of compounds:

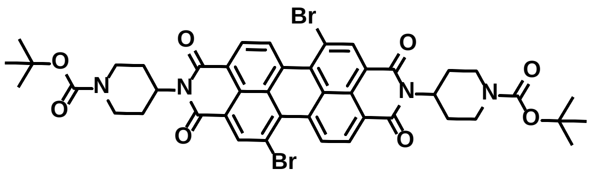

1

***N*,*N'*-Bis-(1-(*tert*-butoxycarbonyl)piperidin-4-yl)-1,7-dibromoperylene-3,4:9,10-tetracarboxylic diimide 1**. 1,7-Dibromoperylene-3,4:9,10-tetracarboxylic dianhydride (TCI D3871) (0.40 g, 0.73 mmol) and 1,4-diazabicyclo[2.2.2]octane (DABCO, 0.81 g, 0.73 mmol) were dried in a microwave vial under inert atmosphere. Then, 20 ml of anhydrous DMF and 4-amino-1-tert-butoxycarbonyl-piperidine (0.29 g, 1.45 mmol) were added. The microwave vial was sealed with a septum and irradiated for one hour at 110ºC. A colour change of the reaction mixture from red to very dark red was observed. The crude product was purified by column chromatography (silica gel, CH_2Cl_2:MeCN, 5:1) to yield the perylene diimide **1** as a red solid (577.5 mg, 87%). MP (ºC): > 350ºC. R_f (CH_2Cl_2:MeOH, 50:2): 0.18. FT-IR (KBr, cm^{-1}): 2971 (C-H, aromatic), 2926 (C-H, aliphatic), 2850 (C-H, aliphatic), 1699 (C=O, carbamate), 1658 (C=O, imide), 1588, 1560, 1501 (C=C), 1452, 1421 (C-N), 1389 (C-N), 1365, 1326, 1242, 1170, 1149 (C-N), 1114, 1041. ^{1}H NMR (400 MHz, $CDCl_3$) δ: 9.47 (d, J = 8.2 Hz, 2H, Ar-H), 8.88 (s, 2H, Ar-H), 8.67 (d, J = 8.2 Hz, 2H, Ar-H), 5.18 (tt, J = 12.1 and 3.9 Hz, 2H, N-CH), 4.49 – 4.17 (m, 4H, CH_2), 3.01 – 2.82 (m, 4H, CH_2), 2.74 (qd, J = 12.1 and 3.9 Hz, 4H, CH_2), 1.75 – 1.67 (m, 4H, CH_2), 1.51 (s, 18H, CH_3). ^{13}C NMR (101 MHz, $CDCl_3$) δ: 163.3 (C), 162.8 (C), 155.0 (C), 138.2 (CH), 133.1 (C), 132.9 (C), 130.3 (CH), 129.3 (C), 128.7 (CH), 127.1 (C), 123.6 (C), 123.2 (C), 121.0 (C), 80.1 (C), 52.4 (CH), 44.2 (CH_2), 29.8 (CH_2), 28.6 (CH_3), 28.4 (CH_2). HR-MS (MALDI-, DIT): *m/z* calcd. for $C_{44}H_{42}Br_2N_4O_8$ ($[M]^-$): 912.1364; found: 912.1315. UV-VIS (CH_2Cl_2) λ_{max}/nm ($\varepsilon/M^{-1}\cdot cm^{-1}$): 519 (72400). Emission (CH_2Cl_2, λ_{ex} = 455 nm) λ_{max}/nm: 550. τ/ns (CH_2Cl_2, χ2): 4.94, (1.12). Φ (CH_2Cl_2, λ_{ex} = 455 nm): 0.93 ± 0.01.

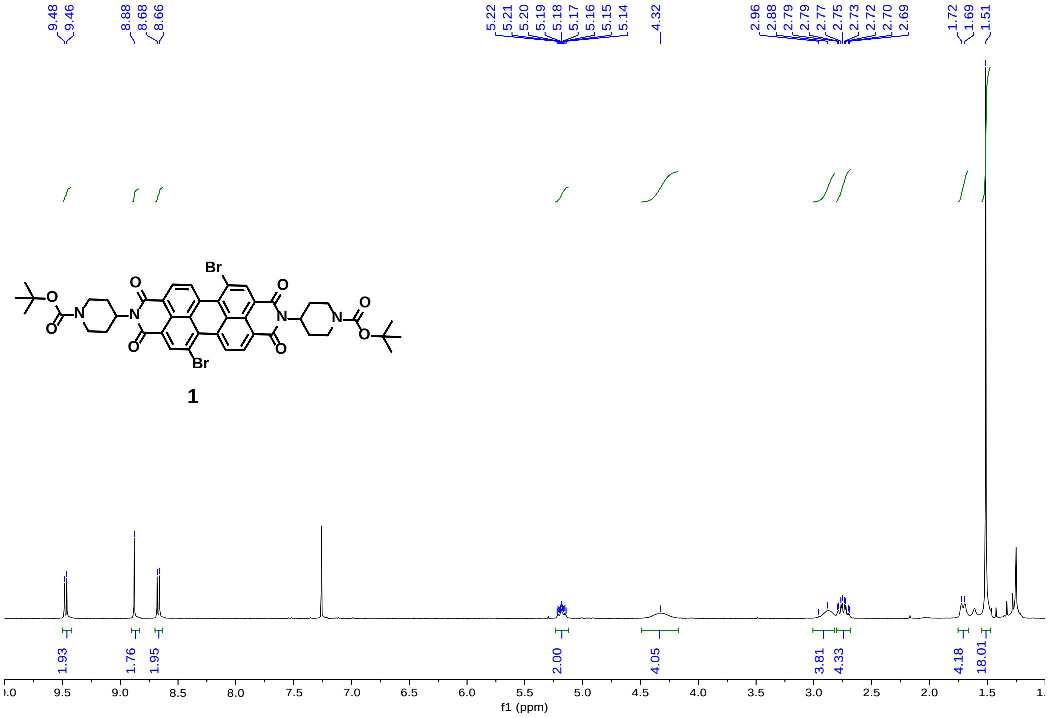

Figure S01: ^{1}H NMR (400 MHz, $CDCl_3$) of 1.

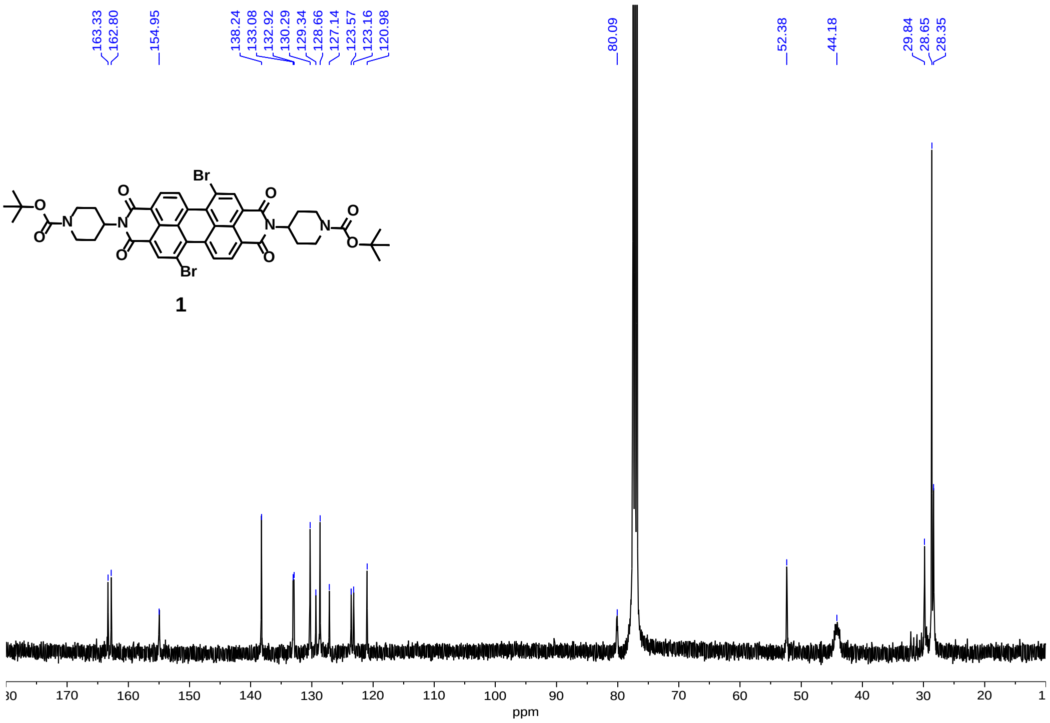

Figure S02: ^{13}C NMR (101 MHz, $CDCl_3$) of 1.

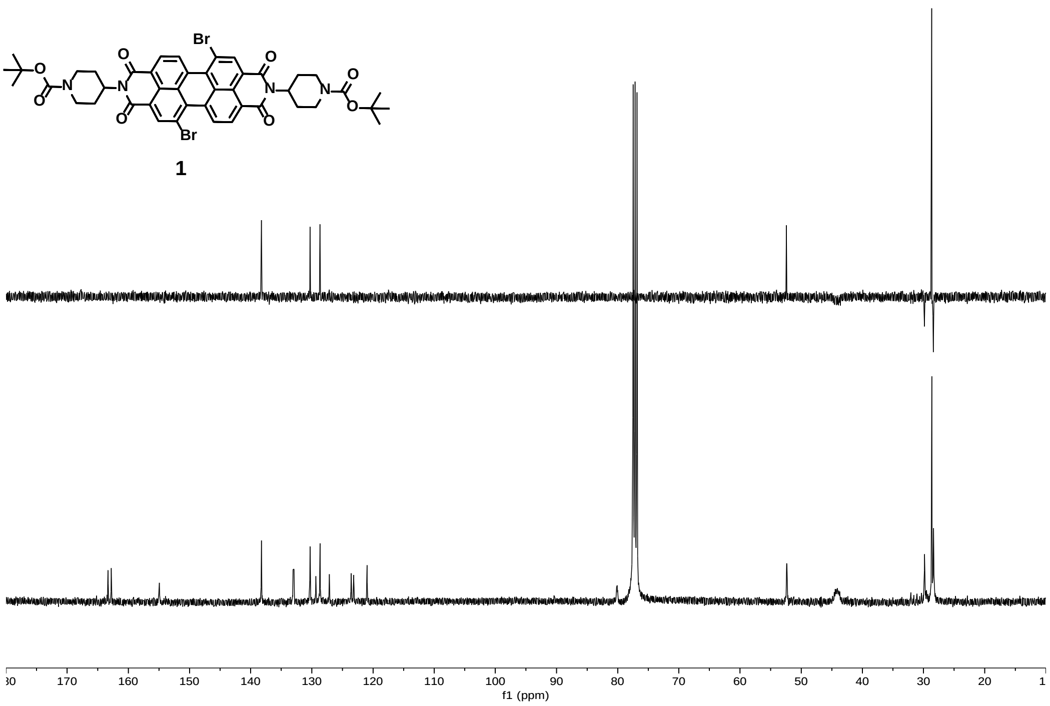

Figure S03: ^{13}C-DEPT 135 NMR (101 MHz, $CDCl_3$) of 1.

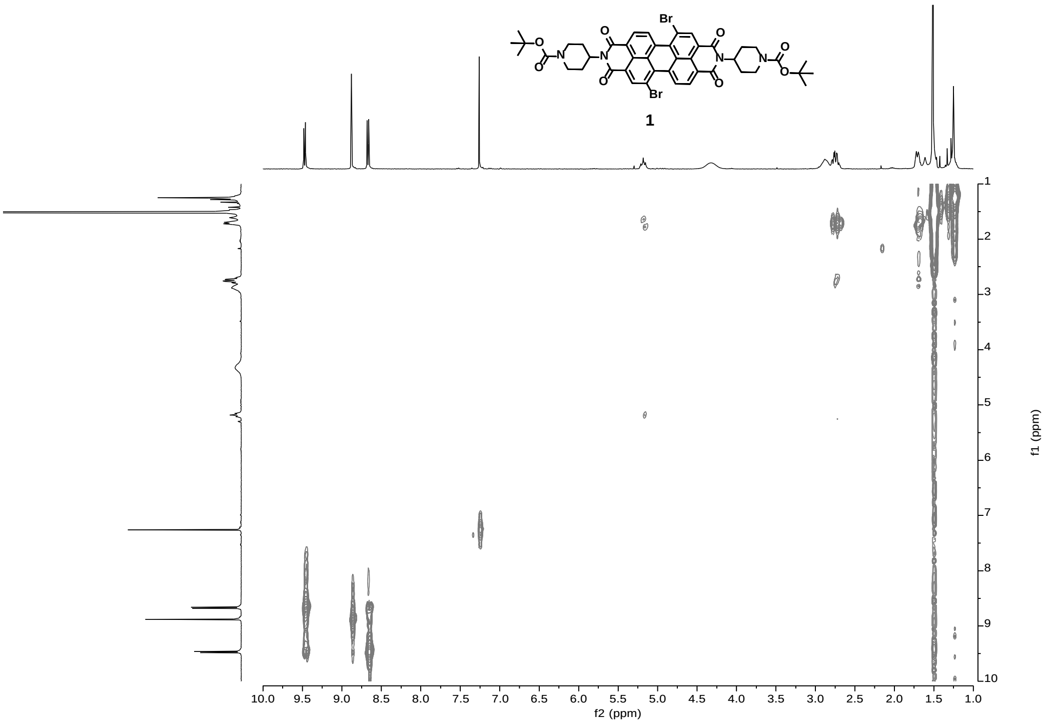

Figure S04: ^{1}H-^{1}H-COSY NMR (400 MHz, $CDCl_3$) of 1.

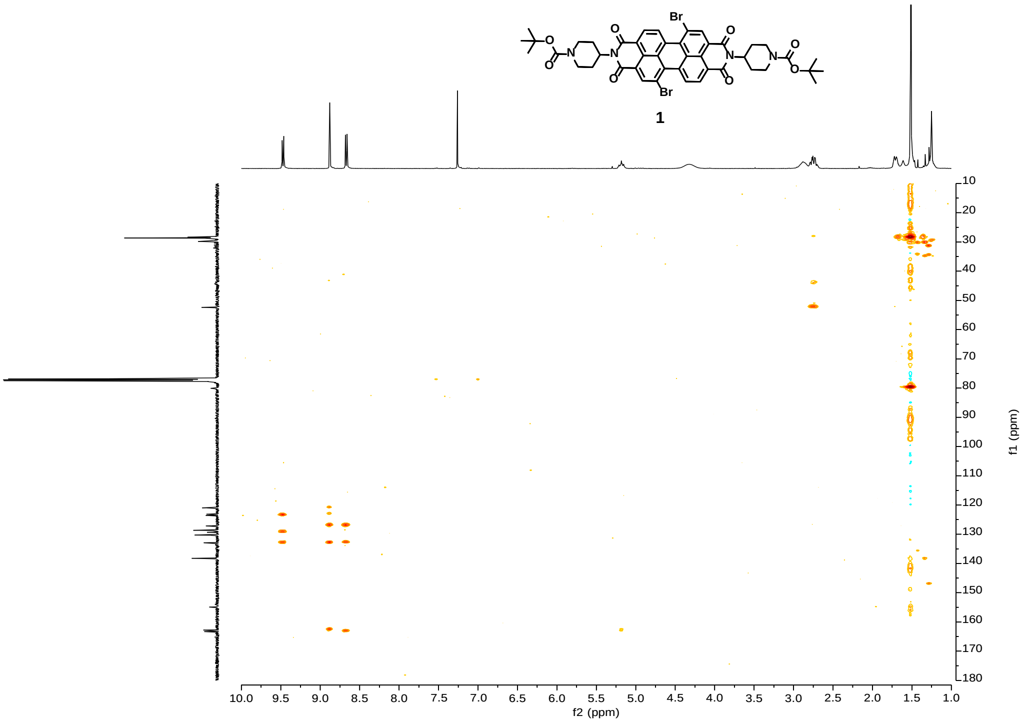

Figure S05: ^{1}H-^{13}C-HMBC NMR (400 MHz, $CDCl_3$) of 1.

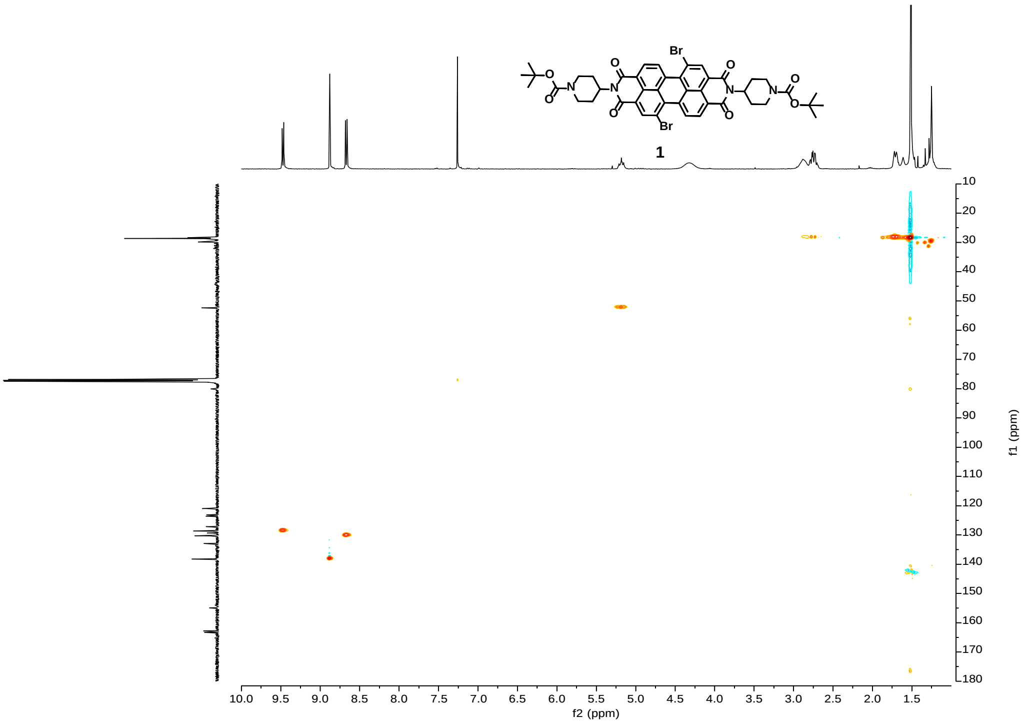

Figure S06: ^{1}H-^{13}C-HMQC NMR (400 MHz, $CDCl_3$) of 1.

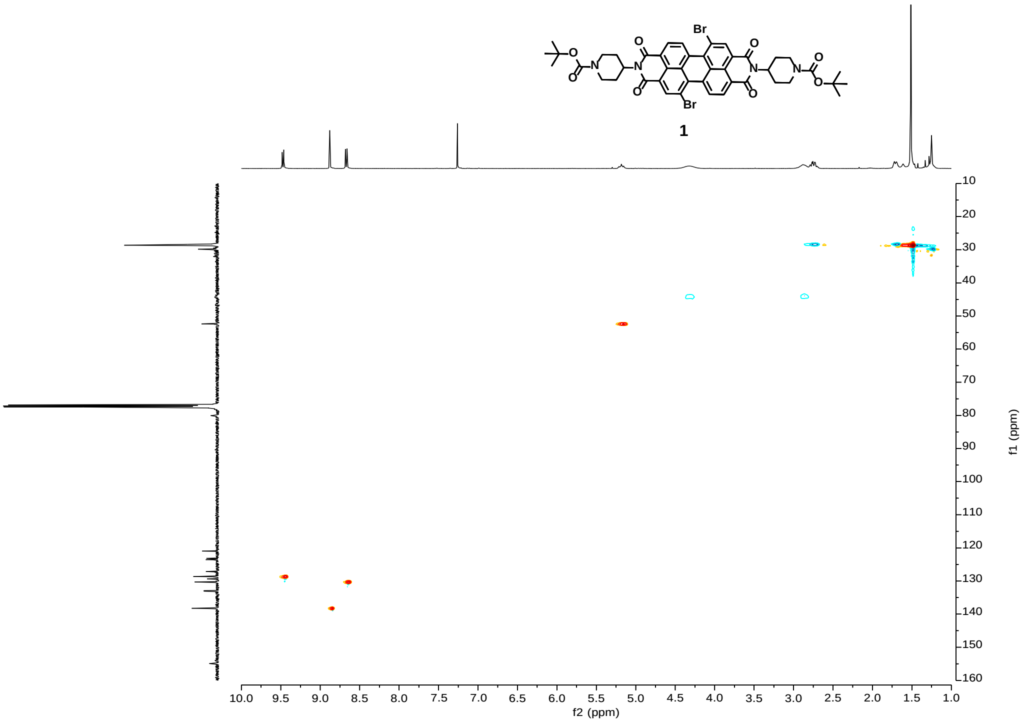

Figure S07: ^{1}H-^{13}C-HSQC NMR (400 MHz, $CDCl_3$) of 1.

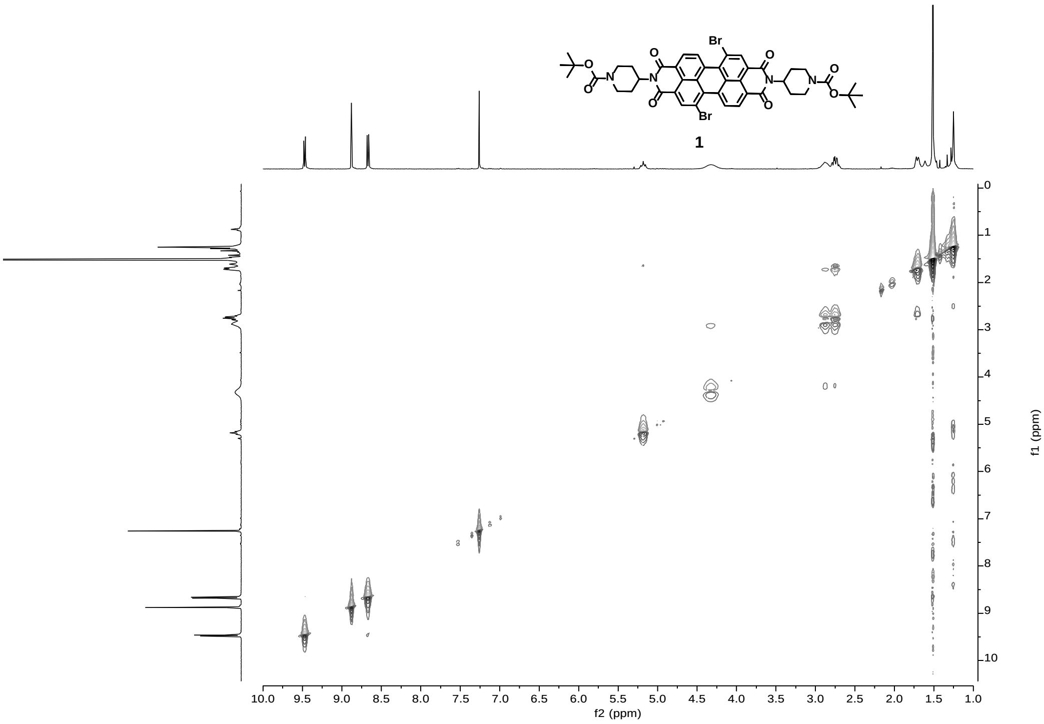

Figure S08: ^{1}H-^{1}H-NOESY NMR (400 MHz, $CDCl_3$) of 1.

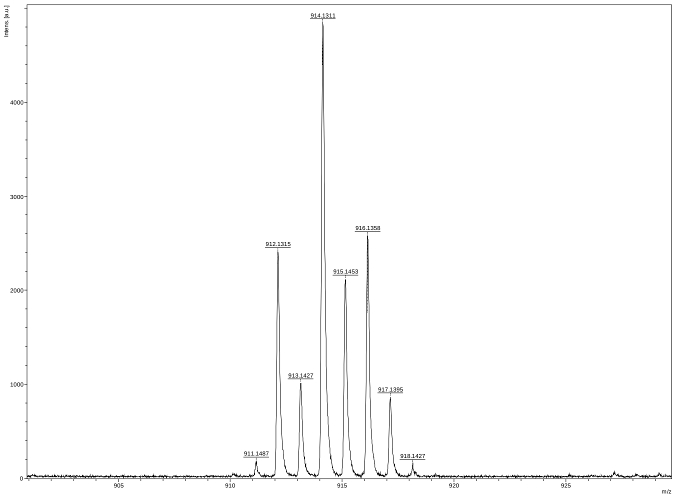

Figure S09: HR-MS (MALDI-, DIT) of 1.

Solvatochromism tests

1 2 3 4 5 6 7 8 9 10 11 12 13 14

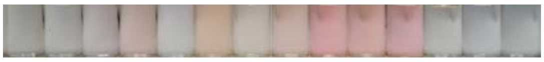

1 2 3 4 5 6 7 8 9 10 11 12 13 14

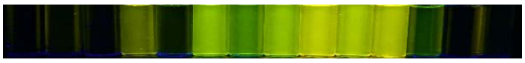

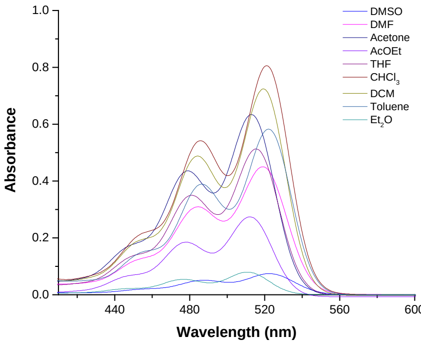

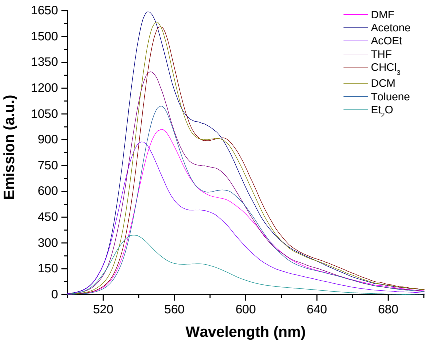

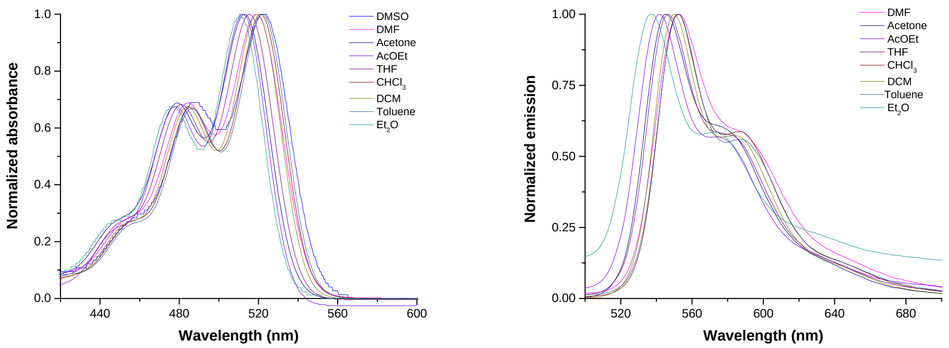

Figure S10: Solvatochromism test of 1: Up: Photos under white and 366 nm lights. Middle: Absorption (left) and emission spectra (right) under excitation wavelength of 455 nm. Down: Normalized absorption spectrum (left) and normalized emission one (right) under excitation wavelength of 455 nm. The employed solvents were: 1: H_2O, 2: MeOH (methanol), 3: DMSO (dimethylsulfoxide), 4: DMF (*N*,*N'*-dimethylformamide), 5: MeCN (acetonitrile), 6: Acetone, 7: EtOAc (ethyl acetate), 8: THF (tetrahydrofuran), 9: $CHCl_3$, 10: CH_2Cl_2 (dichloromethane), 11: Toluene, 12: Et_2O (diethyl ether), 13: *n*-Hx (hexane), 14: *c*-Hx (cyclohexane).

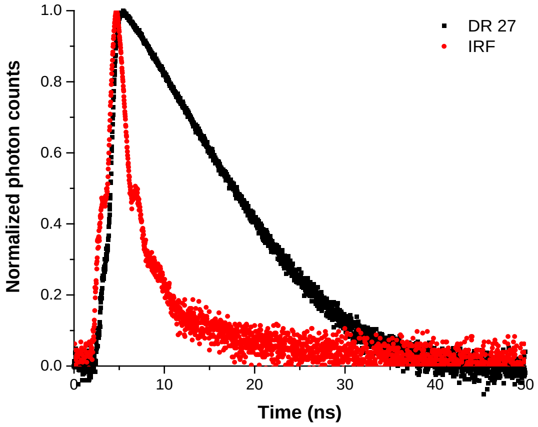

Figure S11: Normalized emission lifetime decay curve of fluorophore 1 (black) and blank (red). Laser employed was 445 nm and emission wavelength 550 nm.

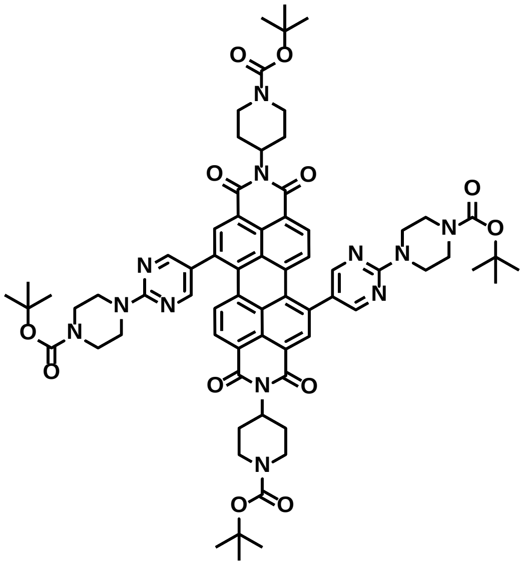

3

***N*,*N'*-Bis-(1-(*tert*-butoxycarbonyl)piperidin-4-yl)-1,7-di((4-(*tert*-butoxy carbonyl)piperazin-1-yl)pyrimidin-1-yl)perylene-3,4:9,10-tetracarboxylic diimide 3**. 85% yield, MP (ºC): > 350ºC. R_f (CH_2Cl_2:MeOH, 50:2): 0.27. FT-IR (KBr, cm^{-1}): 2975 (C-H, aromatic), 2930 (C-H, aliphatic), 2857 (C-H, aliphatic), 1696 (C=O, carbamate), 1661 (C=O, imide), 1588, 1511 (C=C), 1452, 1410 (C-N), 1393 (C-N), 1361, 1341, 1320, 1242, 1166 (C-N), 989. ^{1}H NMR (300 MHz, $CDCl_3$) *δ*: 8.51 (s, 2H, Ar-H), 8.49 (s, 4H, Ar-H), 8.29 (d, *J* = 8.2 Hz, 2H, Ar-H), 8.11 (d, *J* = 8.2 Hz, 2H, Ar-H), 5.24 – 5.10 (m, 2H, N-CH), 4.44 – 4.18 (m, 4H, CH_2), 3.95 – 3.87 (m, 8H, CH_2), 3.61 – 3.54 (m, 8H, CH_2), 2.95 – 2.81 (m, 4H, CH_2), 2.79 – 2.67 (m, 4H, CH_2), 1.73 – 1.66 (m, 4H, CH_2), 1.51 (s, 18H, CH_3), 1.50 (s, 18H, CH_3). ^{13}C NMR (101 MHz, $CDCl_3$) *δ*: 163.7 (C), 163.6 (C), 160.8 (C), 158.1 (CH), 154.9 (C), 154.8 (C), 135.1 (C), 135.1 (CH), 135.0 (C), 132.4 (C), 130.1 (CH), 129.6 (C), 129.4 (CH), 128.1 (C), 124.3 (C), 123.2 (C), 122.5 (C), 80.3 (C), 79.8 (C), 52.2 (CH), 43.9 (CH_2), 31.1 (CH_2), 29.9 (CH_2), 28.6 (CH_3), 28.6 (CH_3), 28.4 (CH_2). HR-MS (MALDI-, DCTB): *m/z* calcd. for $C_{70}H_{80}N_{12}O_{12}$ ($[M]^-$): 1280.6013; found: 1280.5937. UV-VIS (CH_2Cl_2) λ_{max}/nm ($\varepsilon/M^{-1}\cdot cm^{-1}$): 587 (35800). Emission (CH_2Cl_2, λ_{ex}=434 nm) λ_{max} / nm: 670. τ / ns (CH_2Cl_2, χ2): 3.28 (39.60%) and 8.08 (1.05). Φ (CH_2Cl_2, λ_{ex} = 455 nm): 0.34 ± 0.01.

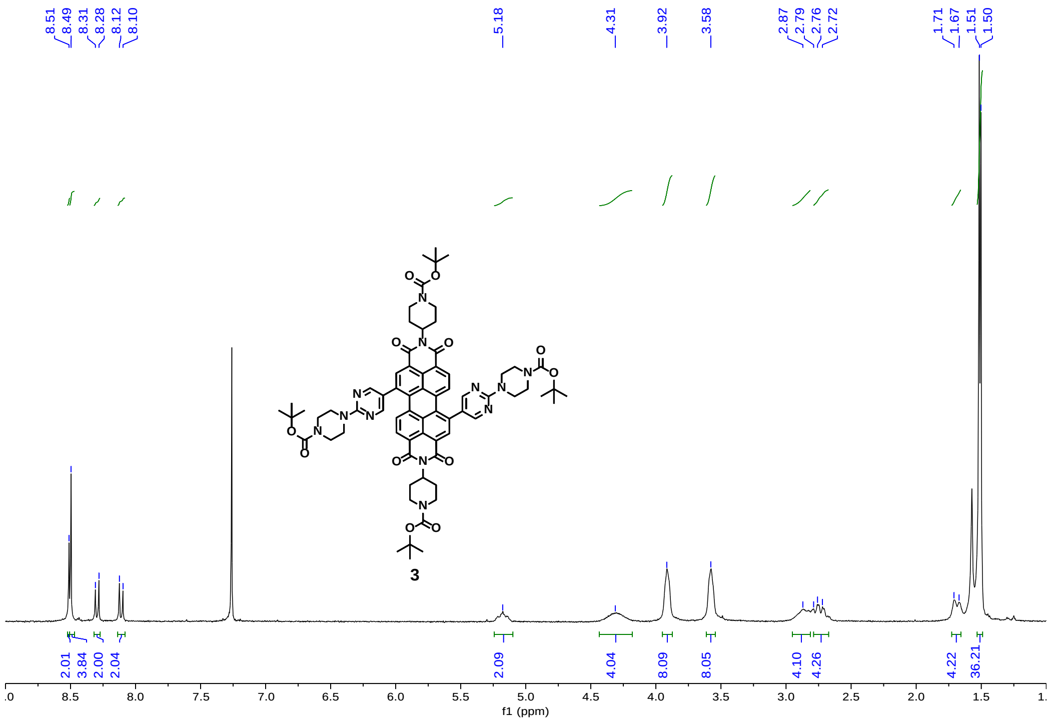

Figure S12: ^{1}H NMR (400 MHz, $CDCl_3$) of 3.

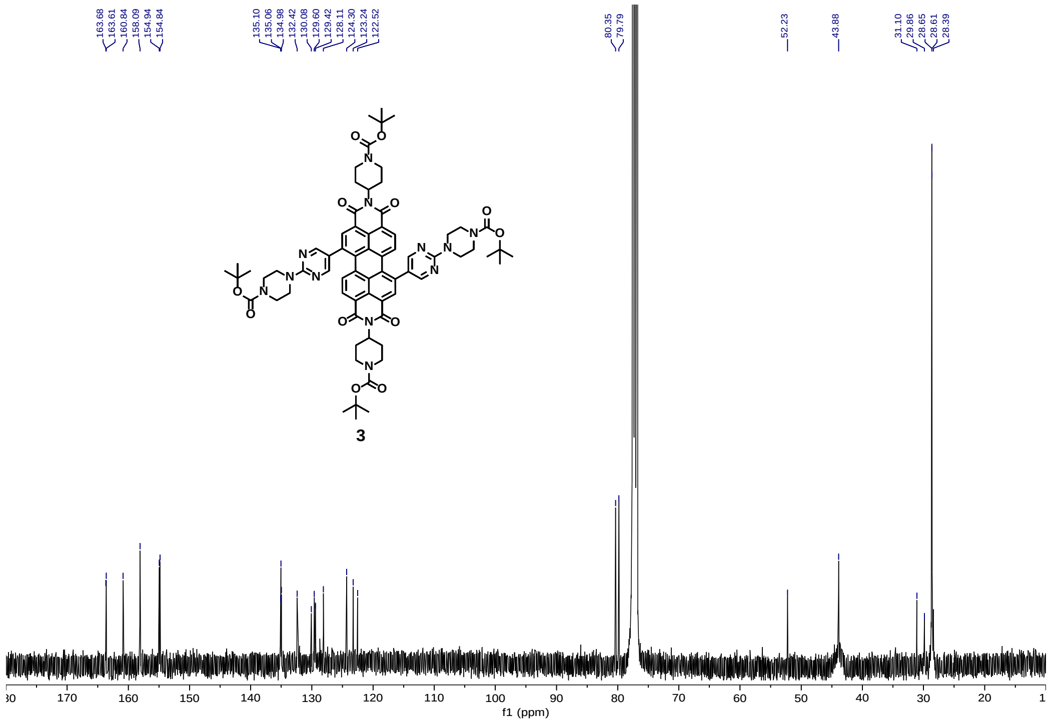

Figure S13: ^{13}C NMR (101 MHz, $CDCl_3$) of 3.

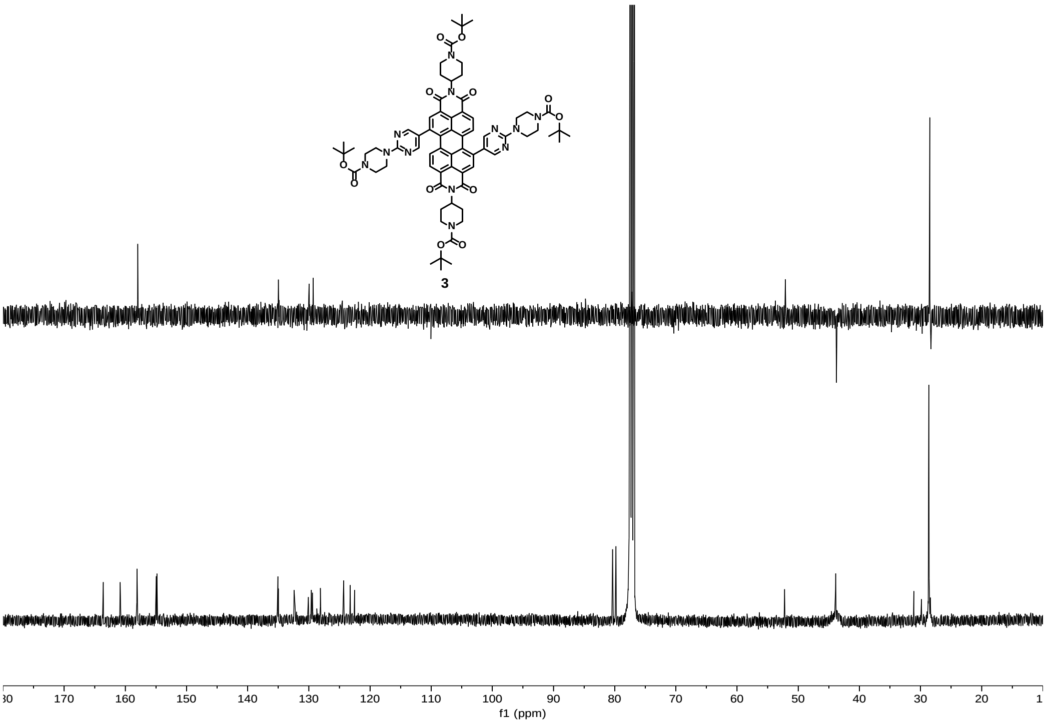

Figure S14: ^{13}C-DEPT 135 NMR (101 MHz, $CDCl_3$) of 3.

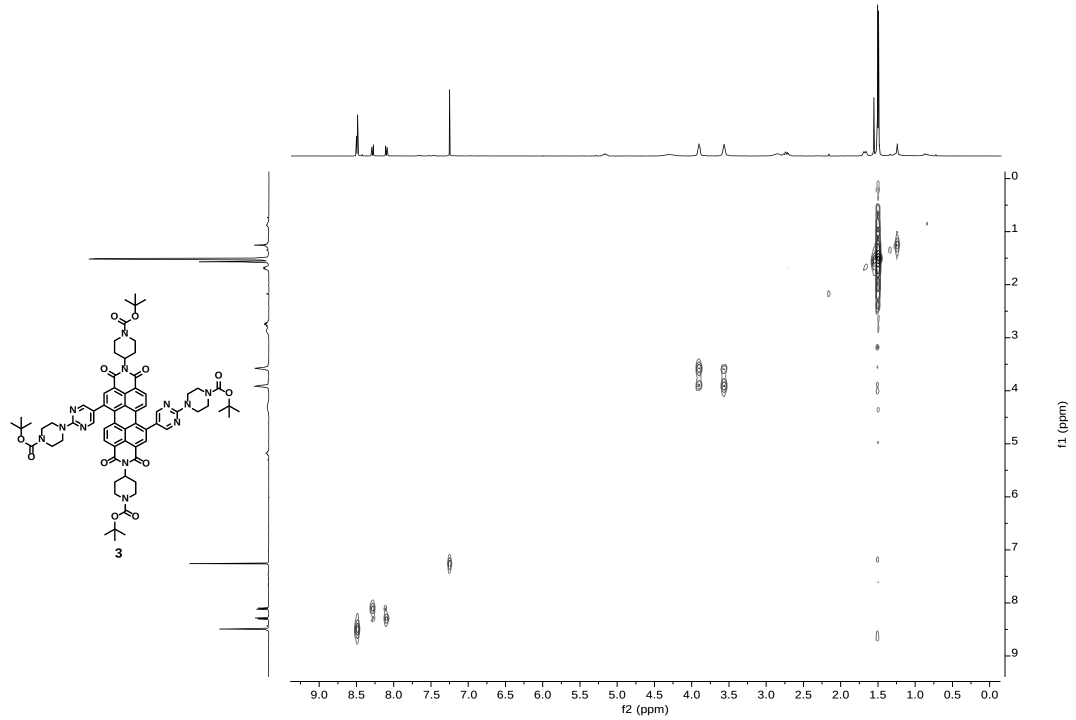

Figure S15: ^{1}H-^{1}H-COSY NMR (400 MHz, $CDCl_3$) of 3.

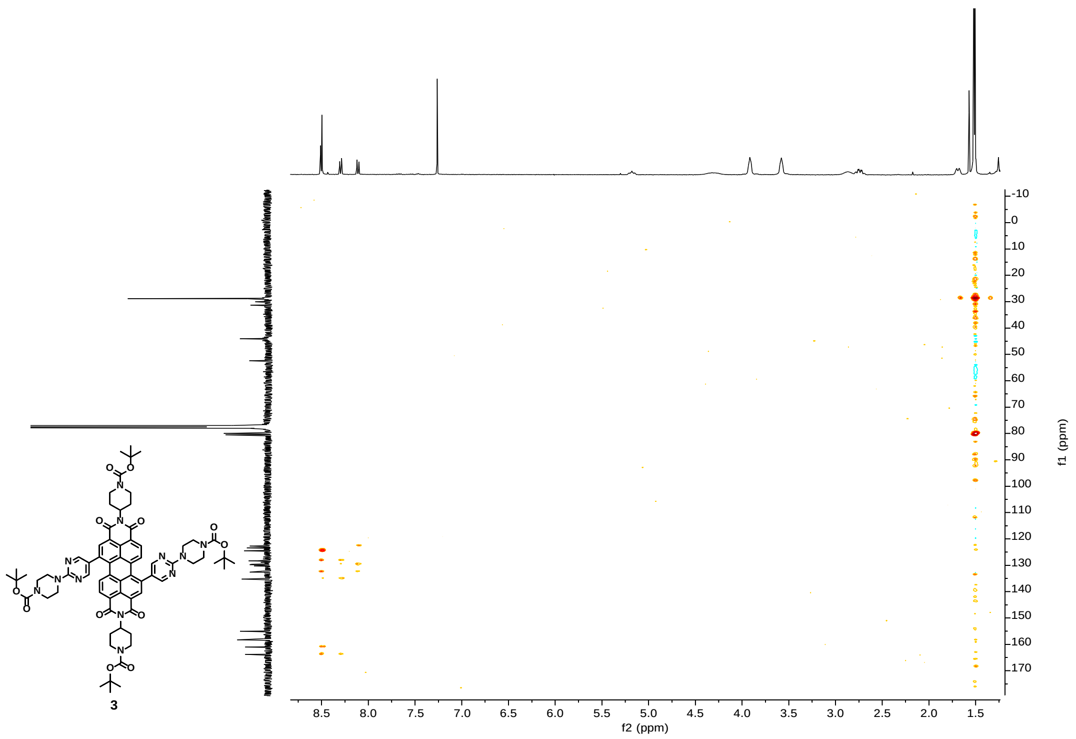

Figure S16: ^{1}H-^{13}C-HMBC NMR (400 MHz, CDCl$_3$) of 3.

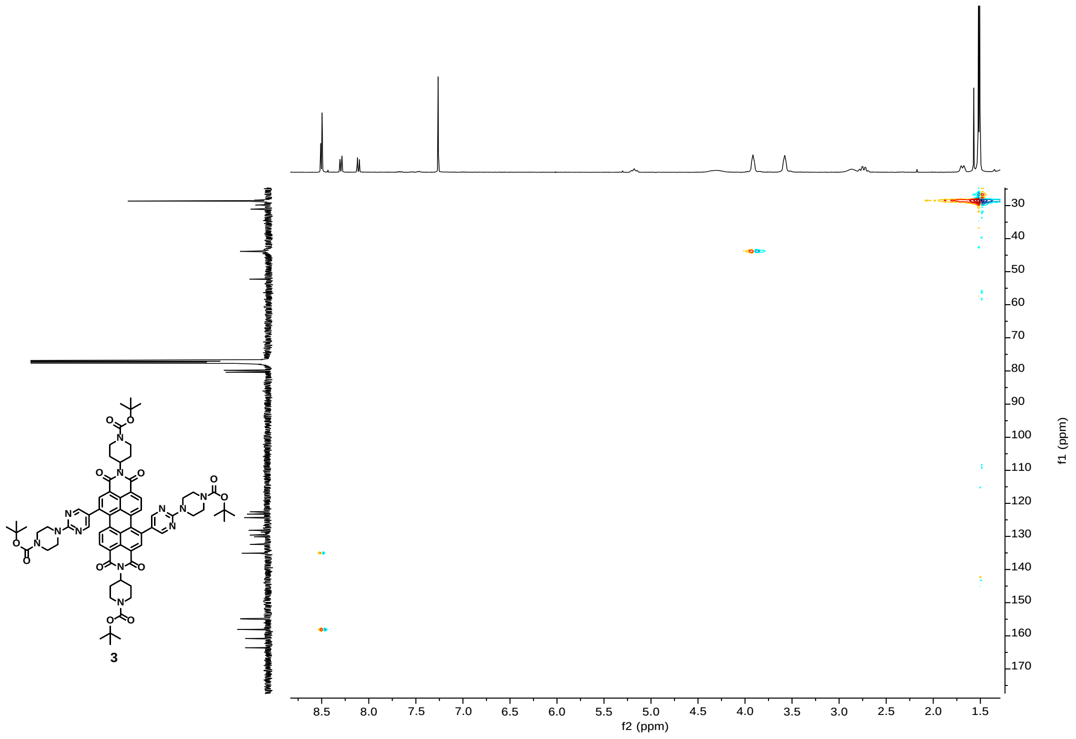

Figure S17: ^{1}H-^{13}C-HMQC NMR (400 MHz, CDCl$_3$) of 3.

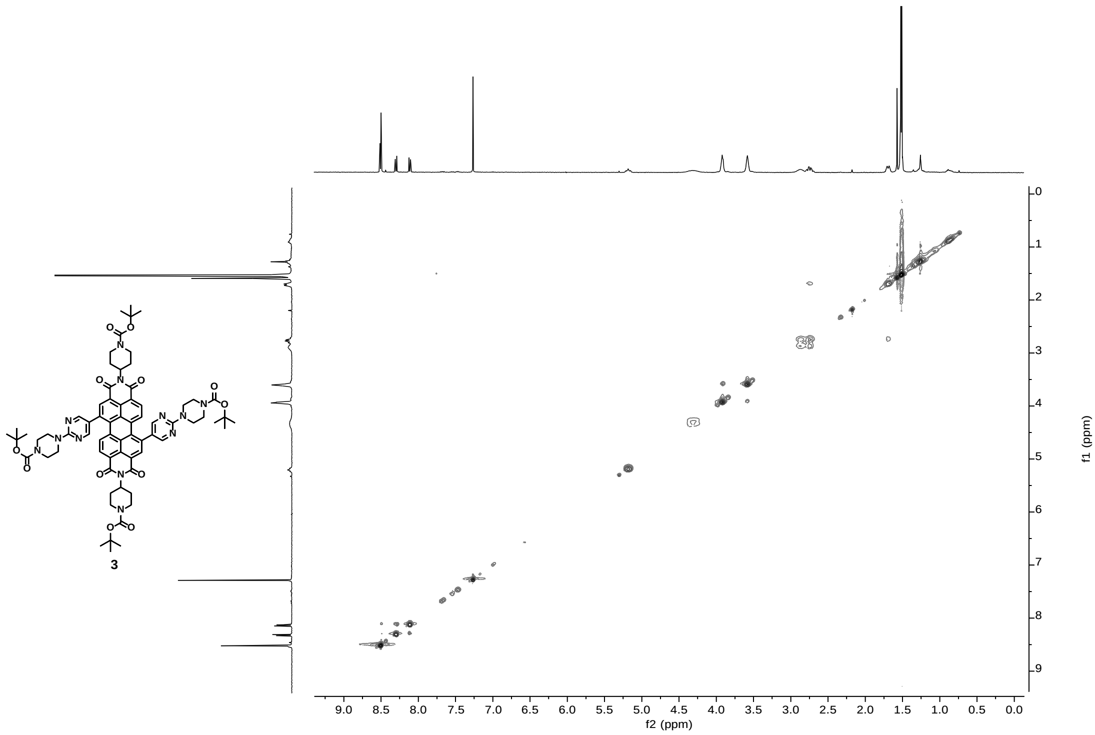

Figure S18: ^{1}H-^{1}H-NOESY NMR (400 MHz, $CDCl_3$) of 3.

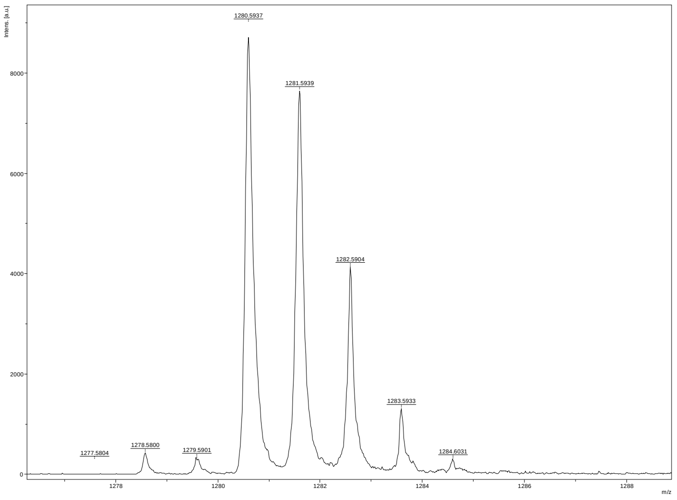

Figure S19: HRMS (MALDI-, DCTB) of 3.

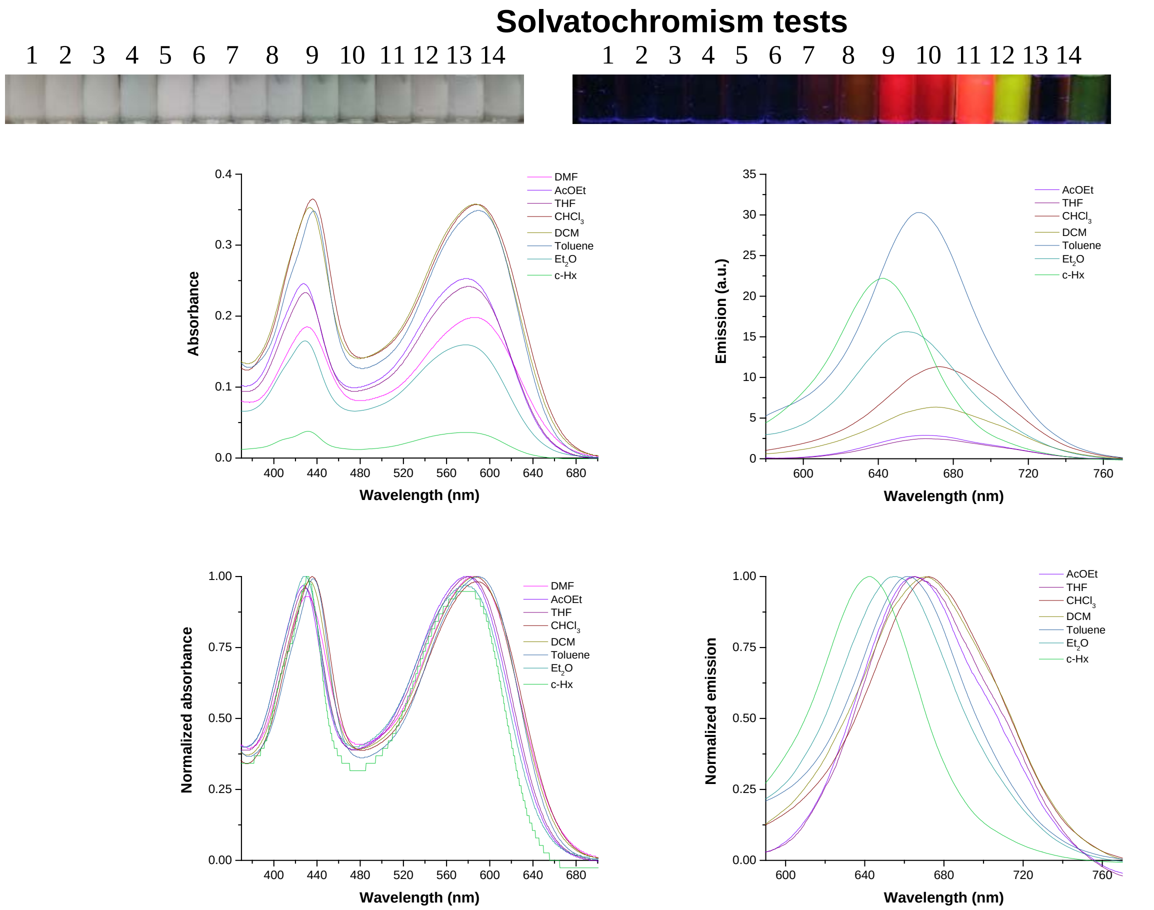

Figure S20: Solvatochromism test of 3: Up: Photos under white and 366 nm lights. Middle: Absorption (left) and emission spectra (right) under excitation wavelength of 434 nm. Down: Normalized absorption spectrum (left) and normalized emission one (right) under excitation wavelength of 434 nm. The employed solvents were: 1: H_2O, 2: MeOH (methanol), 3: DMSO (dimethylsulfoxide), 4: DMF (*N*,*N'*-dimethylformamide), 5: MeCN (acetonitrile), 6: Acetone, 7: EtOAc (ethyl acetate), 8: THF (tetrahydrofuran), 9: $CHCl_3$, 10: CH_2Cl_2 (dichloromethane), 11: Toluene, 12: Et_2O (diethyl ether), 13: *n*-Hx (hexane), 14: *c*-Hx (cyclohexane).

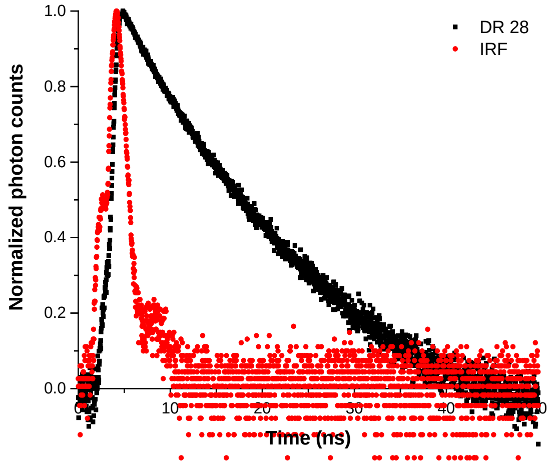

Figure S21: Normalized emission lifetime decay curve of fluorophore 3 (black) and blank (red). The laser employed was 405 nm and the emission wavelength 661 nm.

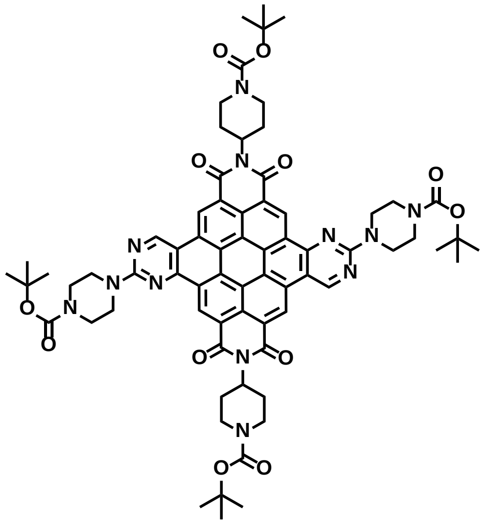

4

***N*,*N'*-Bis-(1-(*tert*-butoxycarbonyl)piperidin-4-yl)-[3,4-*e*]-[9,10-*e*]-bis-(2-(4-(*tert*-butoxycarbonyl)-1,4-piperazin-1-yl)-1,3-pyrimidin)coronene-1,12:6,7-tetracarboxylic diimide 4**. MP (ºC): > 350ºC. R_f (CH_2Cl_2:MeOH, 50:2): 0.41. FT-IR (KBr, cm^{-1}): 2958 (C-H, aromatic), 2923 (C-H, aliphatic), 2853 (C-H, aliphatic), 1699 (C=O, carbamate), 1658 (C=O, imide), 1609, 1570, 1525 (C=C), 1452, 1414 (C-N), 1365 (C-N), 1354, 1341, 1316, 1274, 1246, 1170 (C-N), 1226, 1166, 1083, 992. ^1H NMR (300 MHz, $CDCl_3$) δ: 10.00 – 8.55 (m, 6H, Ar-H), 5.51 – 5.22 (m, 2H, CH_2), 4.66 – 4.43 (m, 4H, CH_2), 4.11 – 3.58 (m, 16H, CH_2), 3.13 – 2.88 (m, 8H, CH_2), 2.31 – 2.07 (m, 4H, CH_2), 1.66 (s, 18H, CH_3), 1.62 (s, 18H, CH_3). HR-MS (MALDI-, DIT): *m/z* calcd. for $C_{70}H_{76}N_{12}O_{12}$ ($[M]^-$): 1276.5760; found: 1276.5760. UV-VIS ($CHCl_3$) λ_{max} / nm (ε / $M^{-1}\cdot cm^{-1}$): 571 (9288). Emission ($CHCl_3$, λ_{ex}=450 nm) λ_{max} / nm: 611. τ / ns ($CHCl_3$ χ2): 9.00 (1.07).
Φ ($CHCl_3$, λ_{ex}=450 nm): 0.46 ± 0.01.

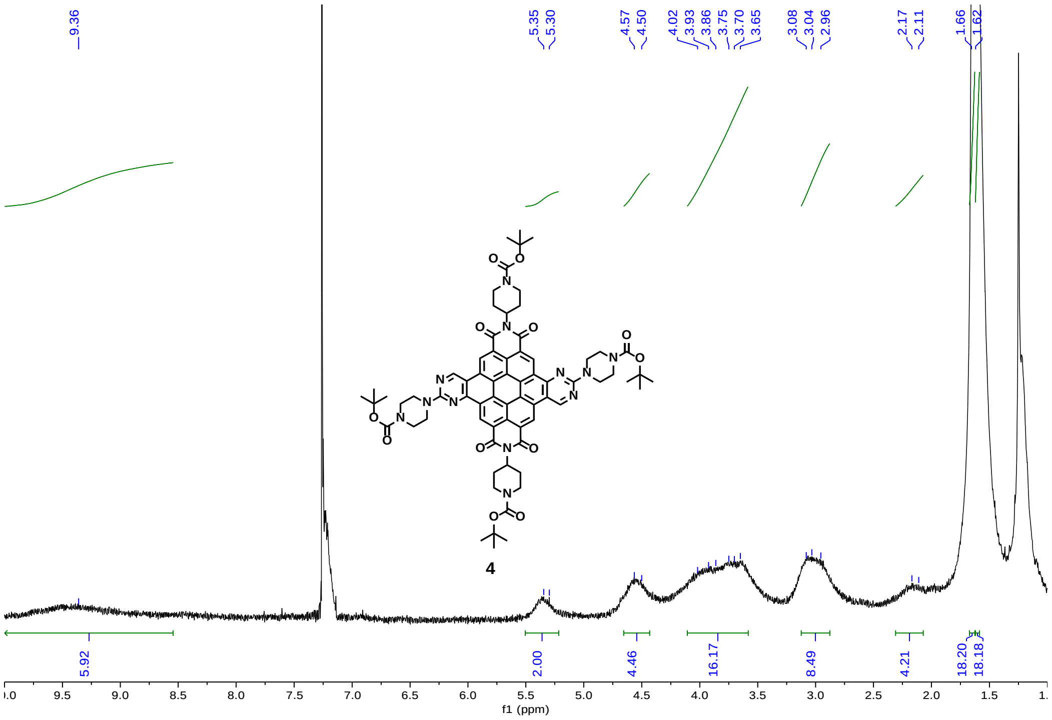

Figure S22: ^1H NMR (300 MHz, $CDCl_3$) of 4.

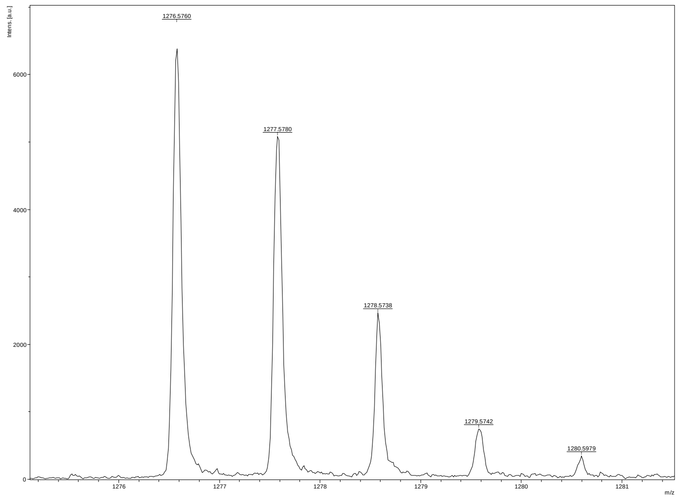

Figure S23: HRMS (MALDI-, DIT) of 4.

Solvatochromism tests

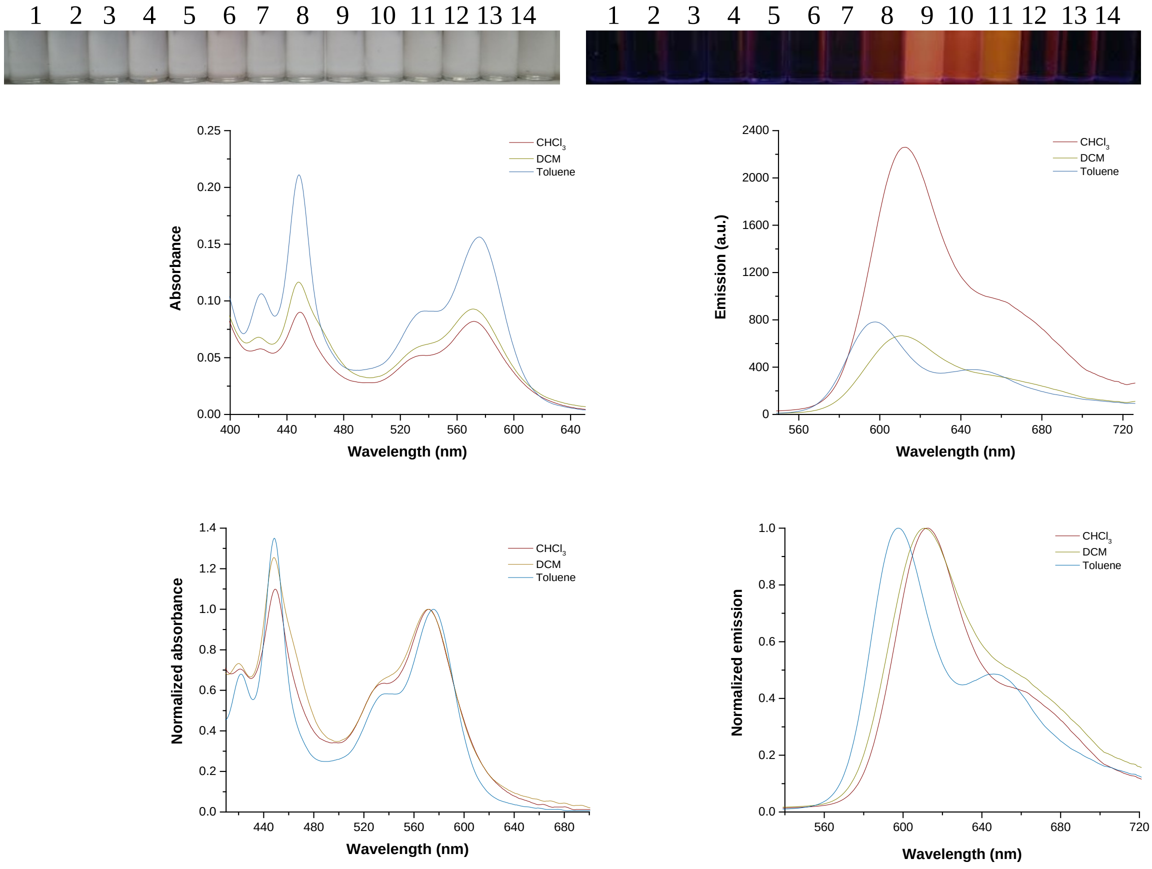

Figure S24: Solvatochromism tests of 4: Up: Photos under white and 366 nm lights. Medium: Absorption (left) and emission spectra (right) under excitation wavelength of 450 nm. Down: Normalized absorption spectrum (left) and normalized emission one (right) under excitation wavelength of 450 nm. The employed solvents were: 1: H_2O, 2: MeOH (methanol), 3: DMSO (dimethylsulfoxide), 4: DMF (*N*,*N'*-dimethylformamide), 5: MeCN (acetonitrile), 6: Acetone, 7: EtOAc (ethyl acetate), 8: THF (tetrahydrofuran), 9: $CHCl_3$, 10: CH_2Cl_2 (dichloromethane), 11: Toluene, 12: Et_2O (diethyl ether), 13: *n*-Hx (hexane), 14: *c*-Hx (cyclohexane).

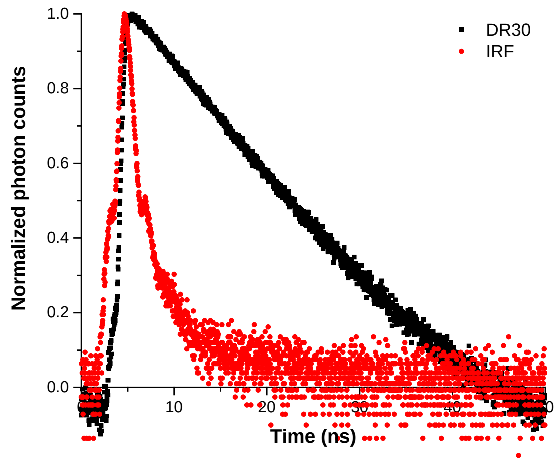

Figure S25: Normalized emission lifetime decay curve of fluorophore 4 (black) and blank (red). The laser employed was 445 nm and the emission wavelength 611 nm.

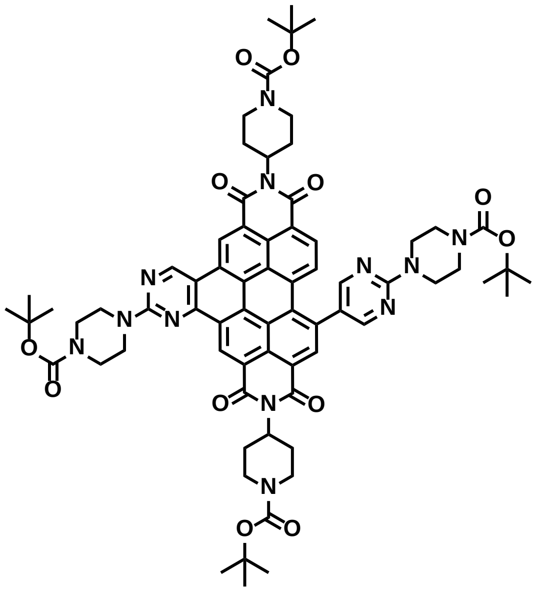

5

N**,*****N'*****-Bis-(1-(*****tert*****-butoxycarbonyl)piperidin-4-yl)-12-((4-(*****tert*****-butoxycarbonyl)-1,4-piperazin-1-yl)-1,3-pyrimidin-5-yl)-[6,7-*****benzo*****-*****e*****]-(2-(4-(*****tert*****-butoxycarbonyl)-1,4-piperazin-1-yl)-1,3-pyrimidin)perylene-3,4:9,10-tetracarboxylic diimide 5**. MP (ºC): > 350ºC. R_f (CH_2Cl_2:MeOH, 50:3): 0.41. FT-IR (KBr, cm^{-1}): 2971 (C-H, aromatic), 2926 (C-H, aliphatic), 2853 (C-H, aliphatic), 1699 (C=O, carbamate), 1661 (C=O, imide), 1602, 1591, 1574, 1511 (C=C), 1452, 1414 (C-N), 1365 (C-N), 1341, 1320, 1242, 1166 (C-N), 992. ^{1}H NMR (300 MHz, $CHCl_2$) δ: 9.69 – 9.11 (m, 3H, Ar-H), 8.89 – 8.27 (m, 5H, Ar-H), 5.36 – 5.22 (m, 2H, N-CH), 4.63 – 4.31 (m, 4H, CH_2), 4.16 – 3.93 (m, 8H, CH_2), 3.77 – 3.58 (m, 8H, CH_2), 3.08 – 2.85 (m, 8H, CH_2), 1.99 – 1.91 (m, 4H, CH_2), 1.59 (s, 18H, CH_3), 1.55 (s, 9H, CH_3). ^{13}C NMR (75 MHz, $CDCl_3$) δ: 163.9, 163.7, 163.4, 163.1, 161.1, 159.1, 157.6, 155.0, 155.0, 154.9, 149.5, 141.3, 135.5, 135.5, 135.5, 135.4, 132.5, 132.5, 131.9, 129.1, 129.1, 127.6, 127.6, 127.0, 127.0, 125.4, 124.7, 123.8, 121.9, 121.8, 121.3, 121.3, 121.3, 80.5 (C), 80.3 (C), 79.9 (C), 79.9 (C), 52.8 (CH), 52.8 (CH), 52.7 (CH), 44.0 (CH_2), 44.0 (CH_2), 29.8 (CH_2), 28.8 (CH_2), 28.8 (CH_3), 28.7 (CH_3), 28.7 (CH_3), 28.6 (CH_2). HR-MS (MALDI-, DCTB): *m/z* calcd. for $C_{70}H_{78}N_{12}O_{12}$ ($[M]^-$): 1278.5857; found:

1278.5905. UV-VIS ($CHCl_3$) λ_{max}/nm (ε/$M^{-1}\cdot cm^{-1}$): 573 (17288). Emission ($CHCl_3$, λ_{ex} = 450 nm) λ_{max}/nm: 667. τ/ns ($CHCl_3$, $\chi 2$): 8.02, (1.12). Φ ($CHCl_3$, λ_{ex} = 450 nm): 0.63 ± 0.01.

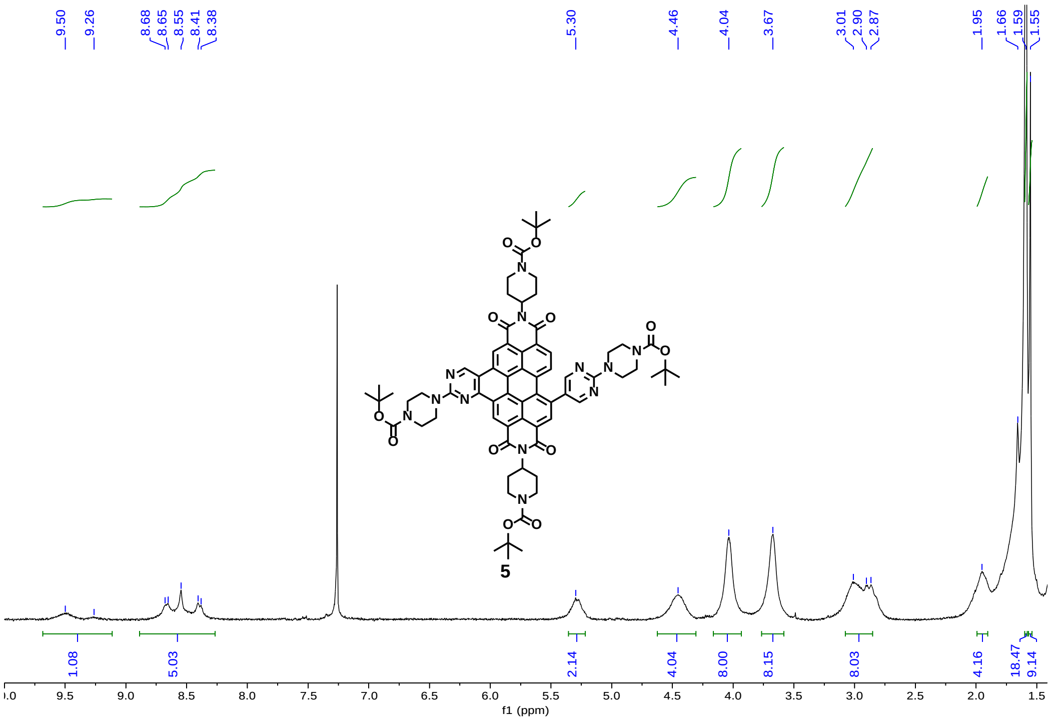

Figure S26: ^{1}H NMR (300 MHz, $CDCl_3$) of 5.

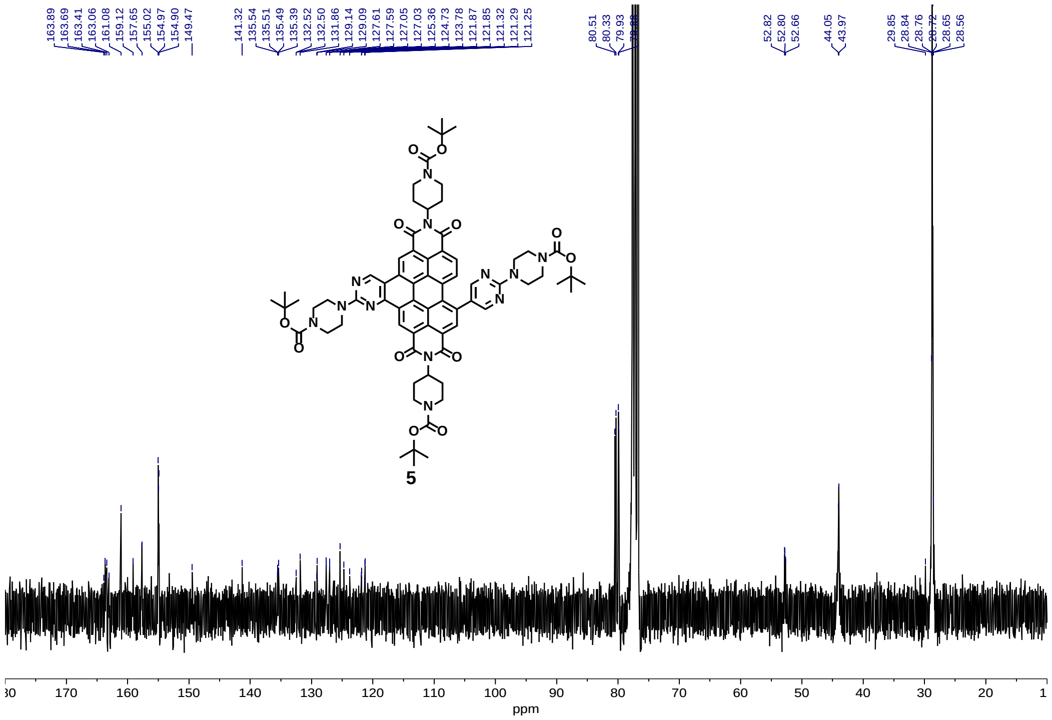

Figure S27: ^{13}C NMR (75 MHz, $CDCl_3$) of 5.

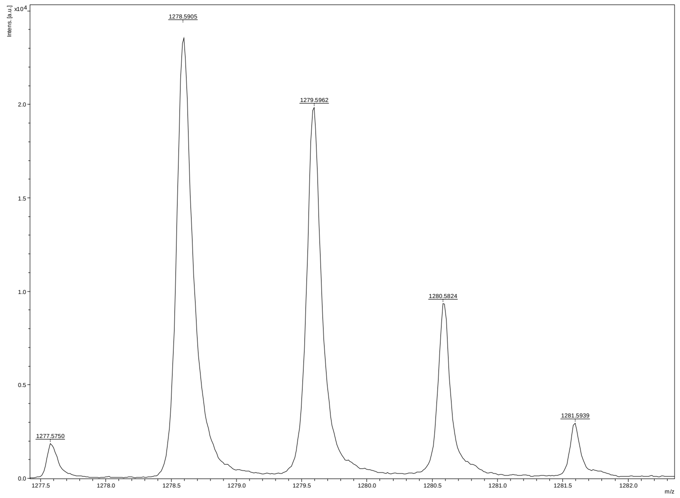

Figure S28: HRMS (MALDI-, DCTB) of 5.

Solvatochromism tests:

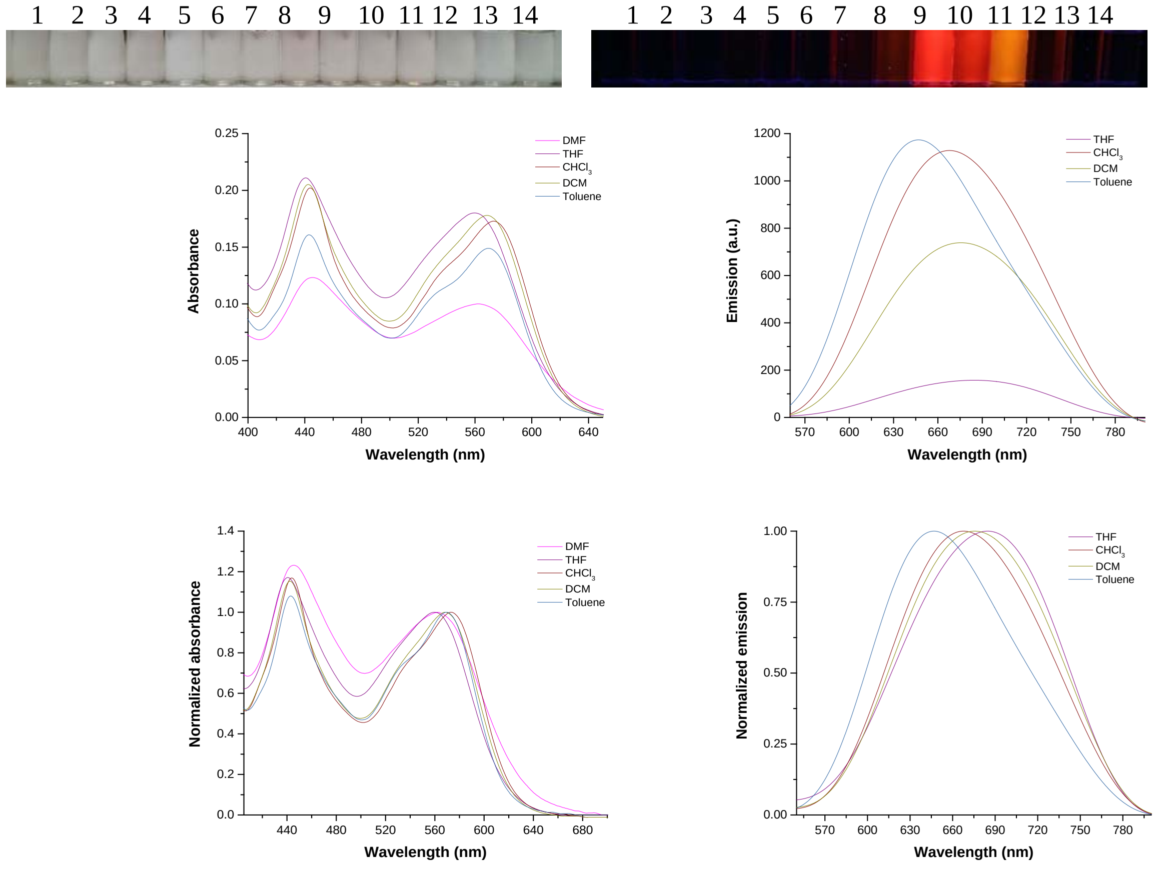

Figure S29: Solvatochromism test of 5: Up: Photos under white and 366 nm lights. Middle: Absorption (left) and emission spectra (right) under excitation wavelength of 450 nm. Down: Normalized absorption spectrum (left) and normalized emission one (right) under excitation wavelength of 450 nm. The employed solvents were: 1: H_2O, 2: MeOH (methanol), 3: DMSO (dimethylsulfoxide), 4: DMF (*N,N'*-dimethylformamide), 5: MeCN (acetonitrile), 6: Acetone, 7: EtOAc (ethyl acetate), 8: THF (tetrahydrofuran), 9: $CHCl_3$, 10: CH_2Cl_2 (dichloromethane), 11: Toluene, 12: Et_2O (diethyl ether), 13: *n*-Hx (hexane), 14: *c*-Hx (cyclohexane).

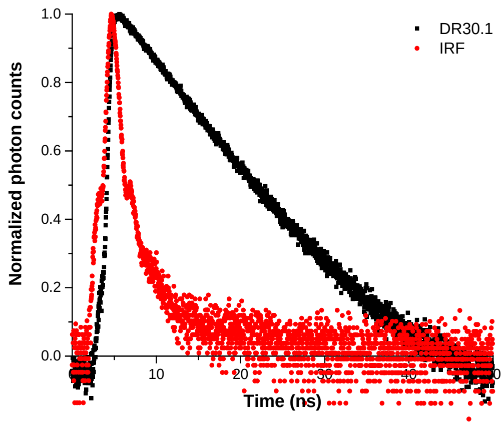

Figure S30: Normalized emission lifetime decay curve of 5 (black) and blank (red). The laser employed was 445 nm and the emission wavelength 621 nm.

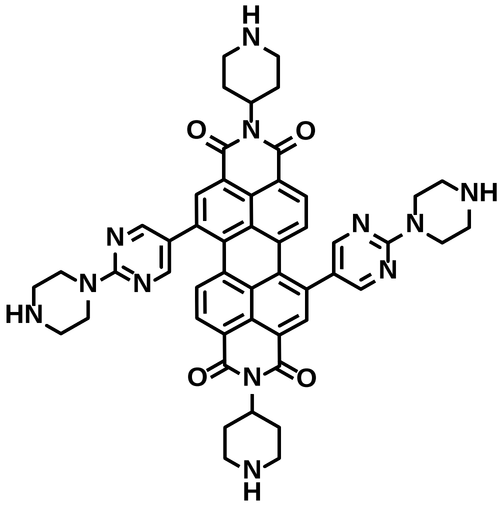

6

***N*,*N'*-Bis(piperidin-1-yl)-1,7-di((piperazin-1-yl)pyrimidin-1-yl)perylene-3,4:9,10-tetracarboxylic diimide 6**. MP (ºC): > 350ºC. R_f (CH_2Cl_2:MeOH, 50:4): 0.01. FT-IR (KBr, cm^{-1}): 3438 (N-H, amine), 2965 (C-H, aromatic), 2923 (C-H, aliphatic), 2853 (C-H, aliphatic), 1769, 1706 (C=O, carbamate), 1678 (C=O, imide), 1637, 1602, 1563, 1511 (C=C), 1435 (C-N), 1365 (C-N), 1236, 1208 (C-C), 1135 (C-N), 1184, 1097. ^{1}H NMR (300 MHz, D_2O) δ: 8.29 – 7.76 (m, 6H, Ar-H), 7.67 – 7.28 (m, 4H, Ar-H), 5.37 – 5.13 (m, 2H, N-CH), 4.08 – 3.98 (m, 8H, CH_2), 3.77 – 3.64 (m, 4H, CH_2), 3.38 – 3.28 (m, 12H, CH_2), 2.95 – 2.80 (m, 2H, CH_2), 2.66 – 2.48 (m, 2H, CH_2), 2.17 – 2.15 (m, 4H, CH_2). HR-MS (MALDI+, DCTB): *m/z* calcd. for $C_{50}H_{48}N_{12}O_4$ ($[M+H]^+$): 881.3994; found: 881.3979. MS (MALDI+, DCTB) 2DR36: m/z calcd. for $2(C_{50}H_{48}N_{12}O_4)$ ($[2M+H]^+$): 1763.7988; found: 1767.66. MS (MALDI+, DCTB) [6]+CB[7]: m/z calcd. for ($C_{50}H_{49}N_{12}O_4$+$C_{42}H_{42}N_{28}O_{14}$) ($[M+CB[7]+H]^+$): 2044.7429; found: 2045.73. UV-VIS (H_2O) λ_{max} / nm (ε / $M^{-1}\cdot cm^{-1}$): 463 (258). Emission (H_2O, λ_{ex} = 463 nm) λ_{max} / nm: 502. τ / ns (H_2O $\chi 2$): 2.36 (32.48%) and 7.71 (67.52%) (1.14). Φ (H_2O, λ_{ex}=463 nm): 0.02 $\pm$ 0.01.

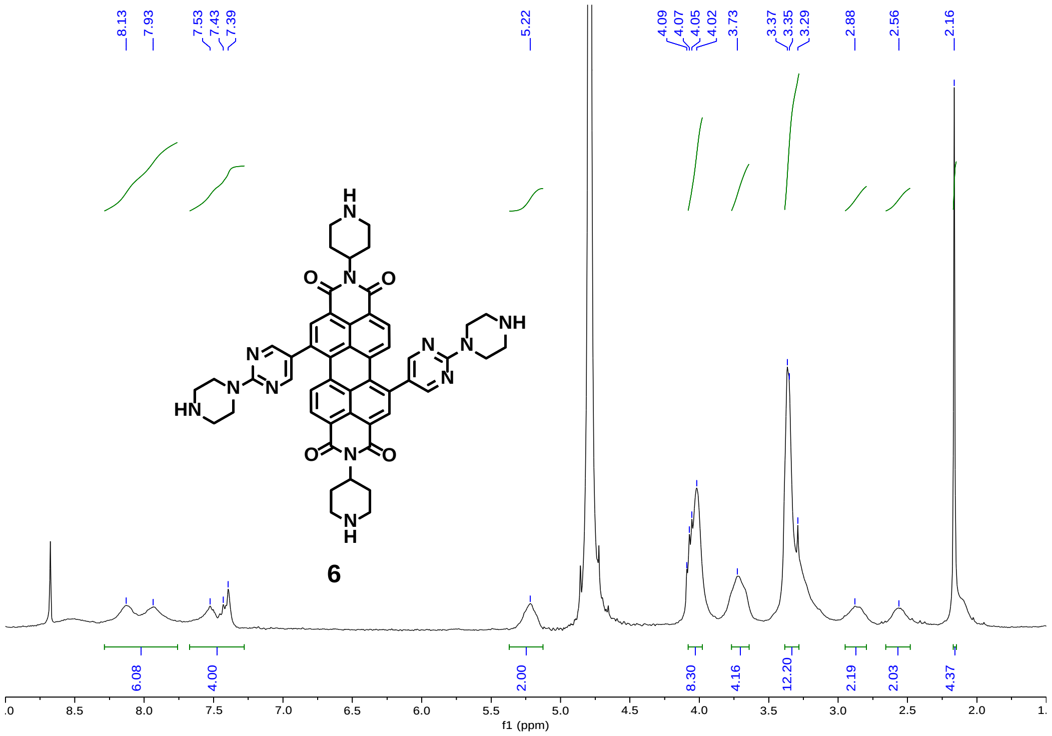

Figure S31: ^{1}H NMR (300 MHz, D_2O) of 6.

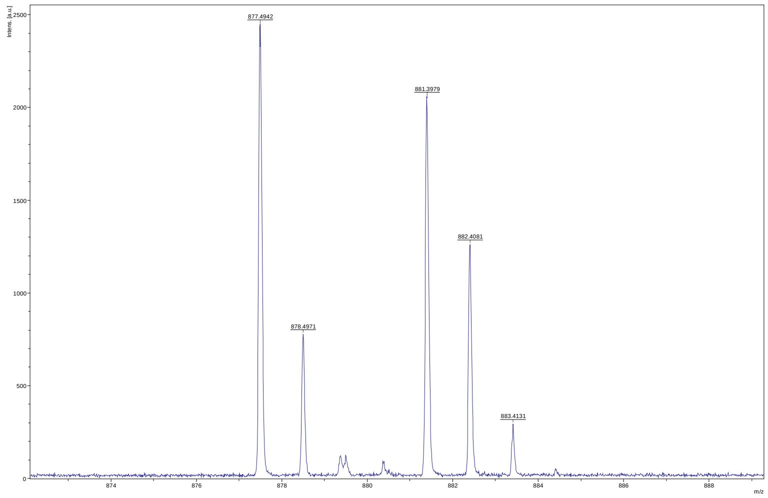

Figure S32: HRMS (MALDI+, DCTB) of 6.

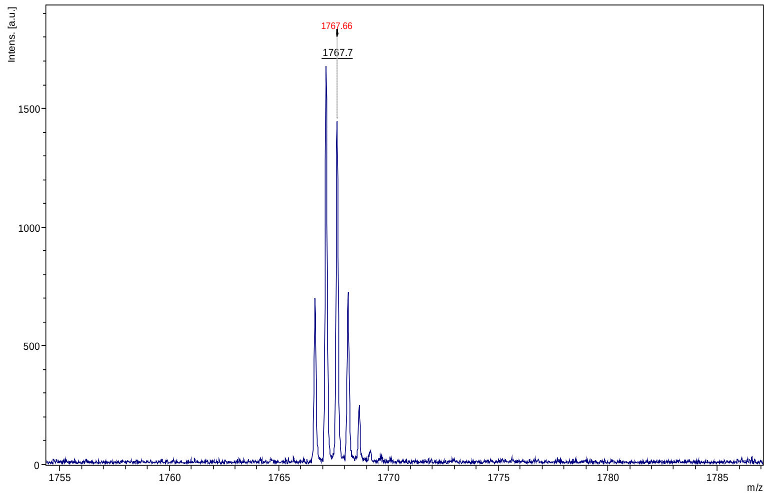

Figure S33: HRMS (MALDI+, DCTB) of 2[6] stacked.

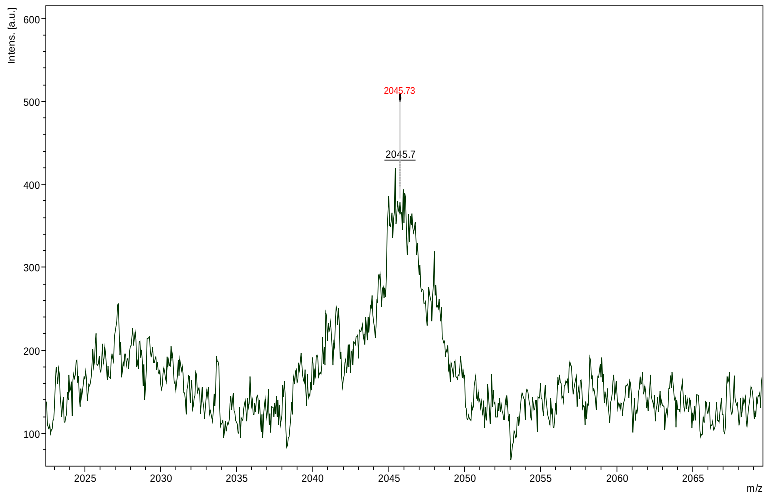

Figure S34: HRMS (MALDI+, DCTB) 6 + 1CB[7].

Solvatochromism tests:

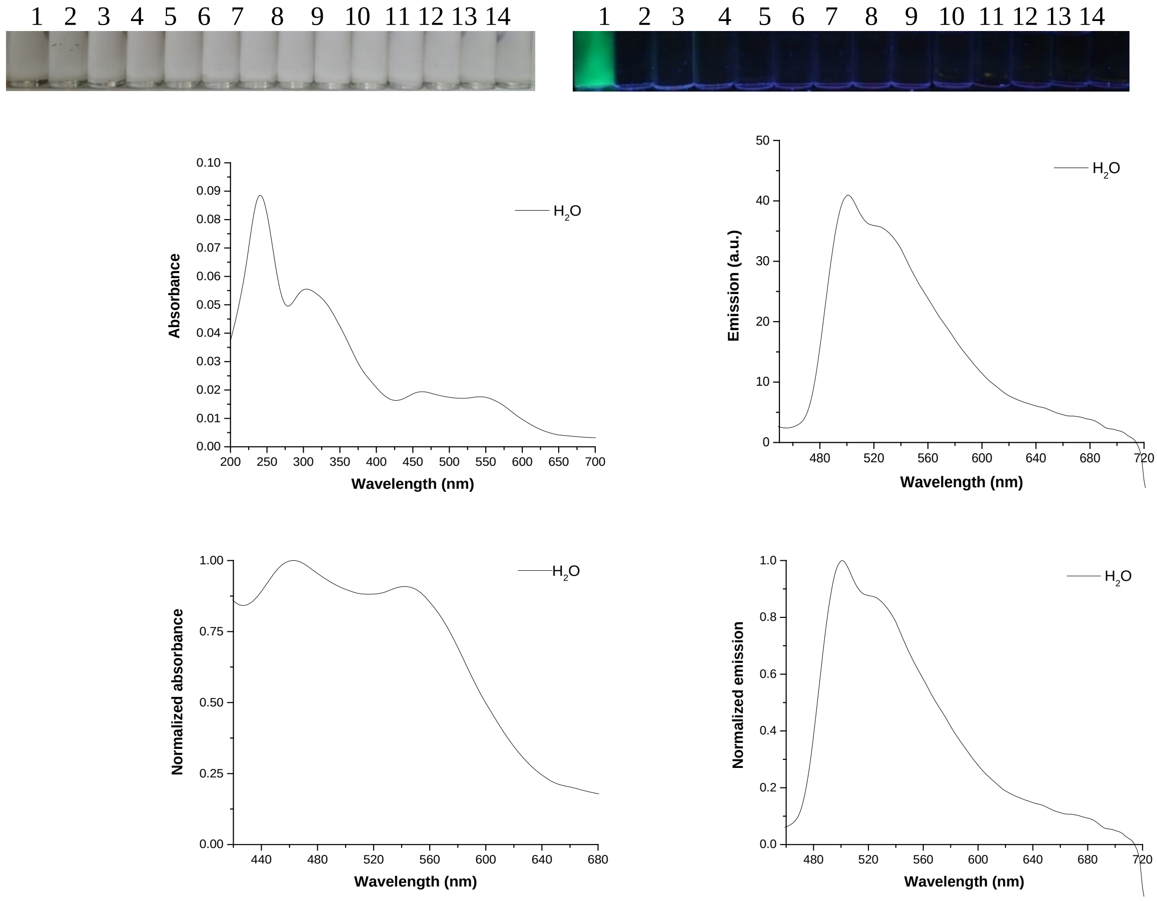

Figure S35: Solvatochromism tests of 6: Up: Photos under white and 366 nm lights. Middle: Absorption (left) and emission spectra (right) under excitation wavelength of 410 nm. Down: Normalized absorption spectrum (left) and normalized emission one (right) under excitation wavelength of 410 nm. The employed solvents were: 1: H_2O, 2: MeOH (methanol), 3: DMSO (dimethylsulfoxide), 4: DMF (*N*,*N'*-dimethylformamide), 5: MeCN (acetonitrile), 6: Acetone, 7: EtOAcO (ethyl acetate), 8: THF (tetrahydrofuran), 9: $CHCl_3$, 10: CH_2Cl_2 (dichloromethane), 11: Toluene, 12: Et_2O (diethyl ether), 13: *n*-Hx (hexane), 14: *c*-Hx (cyclohexane).

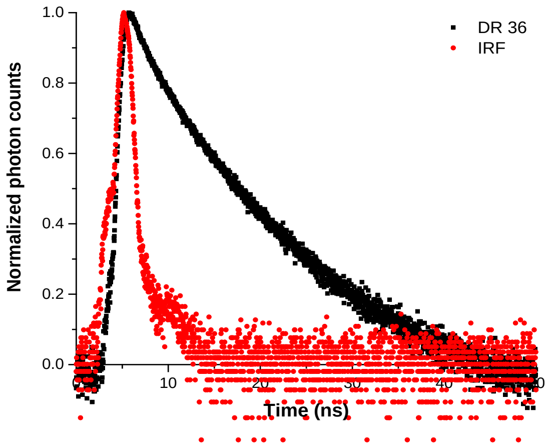

Figure S36: Normalized emission lifetime decay curve of fluorophore 6 (black) and blank (red). The laser employed was 445 nm and the emission wavelength 502 nm.

Lambert-Beer study: Compound was dissolved in water, in a concentration range of 1 to 1000 µM (11 points). Absorbance and emission (excitation wavelength 381 nm) were measured for each concentration. A significant quenching of the fluorescence (self-aggregation) was observed at concentrations above 75 µM. The selected work concentration was 10 µM.

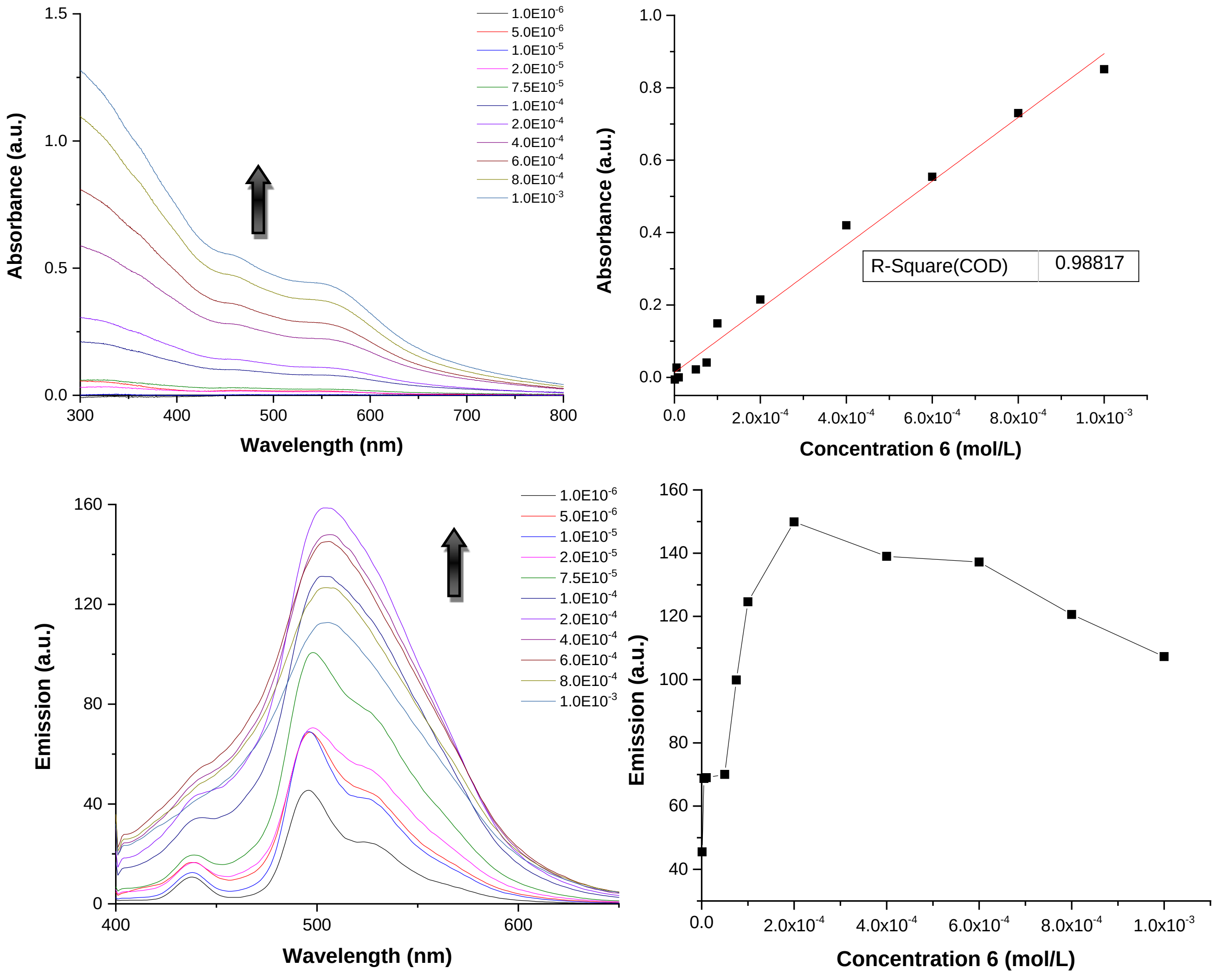

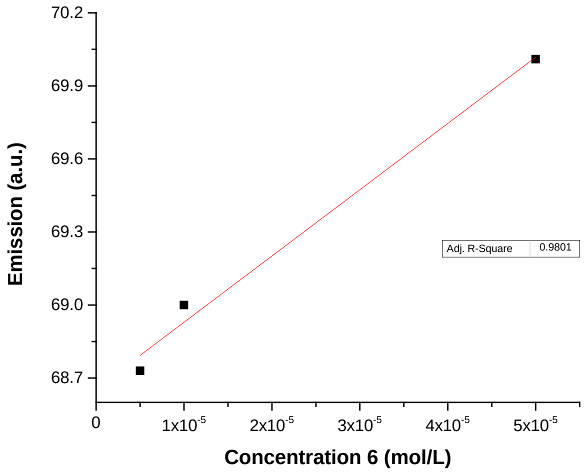

Figure S37: Up: Left: Absorbance spectra at different concentrations. Right: Representation of absorbance maxima versus concentration. Down: Left: Emission spectra at different concentrations. Right: Representation of emission maxima versus concentration. Inset: Representation of emission maxima versus concentration in the first points.

Kinetic effect study:

Compound was dissolved in water. Absorbance and emission (excitation wavelength 387 nm) were measured for 270 minutes with a rate of 1 spectrum by every 3 minutes. An increase of absorbance and emission intensity until 200 min was observed.

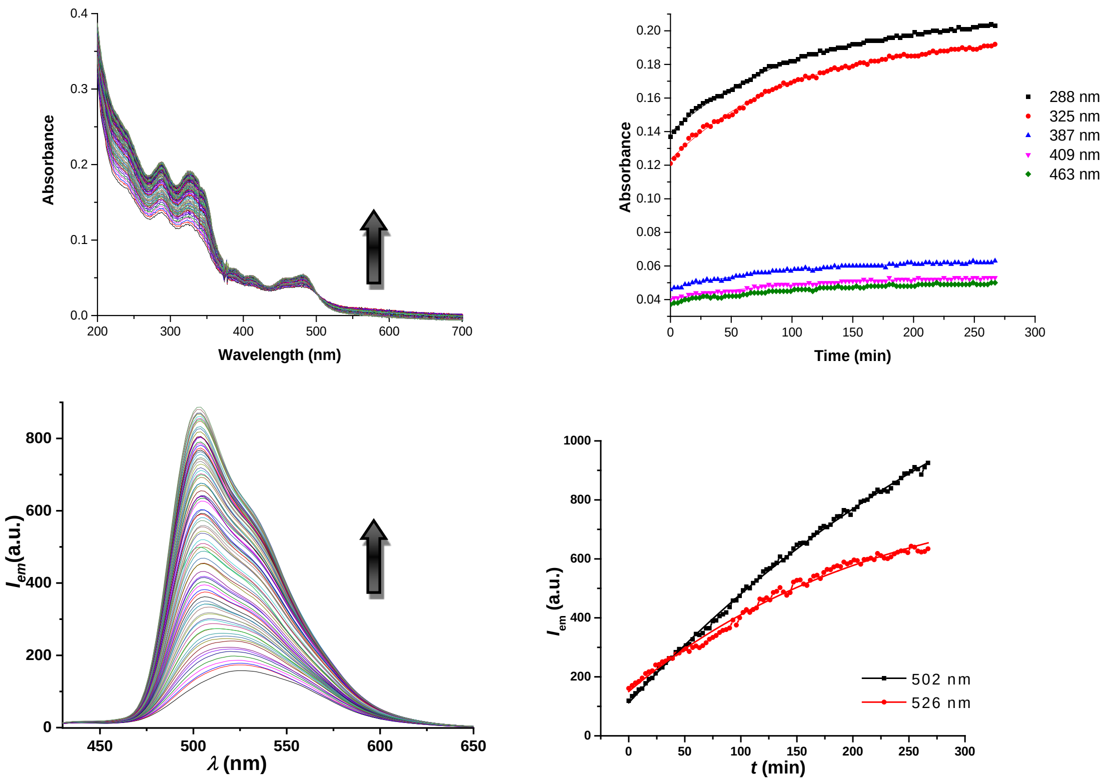

Figure S38: Up: Left: Absorbance spectra at different time points. Right: Representation of absorbance maxima along the time. Down: Left: Emission spectra at different time points. Right: Representation of emission maxima along the time.

Morphology: AFM images of samples prepared in water in different concentrations. Solutions were prepared freshly, five days or one month before.

Solution				
**Freshly prepared**		**Prepared after five days in solution**		**Prepared after one month**
**0.1 µg/mL**	**1 µg/mL**	**0.1 µg/mL**	**1 µg/mL**	**1 µg/mL**
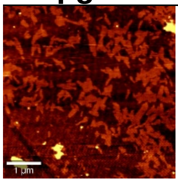	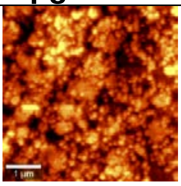	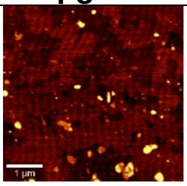	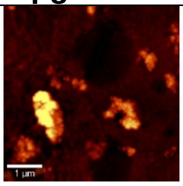	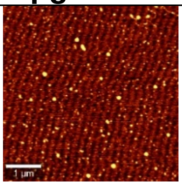
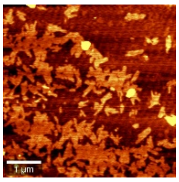		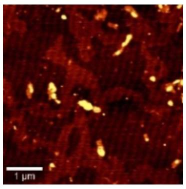	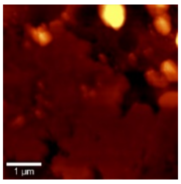	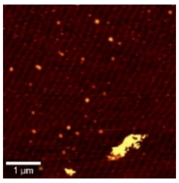
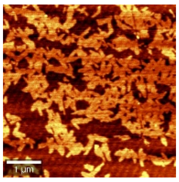		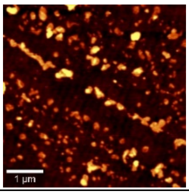		

Figure S39: AFM images of samples of 6 in water.

DLS (Dynamic Light Scattering) study, samples prepared in water 0.1 µg/mL:
As prepared, average, three repetitions:

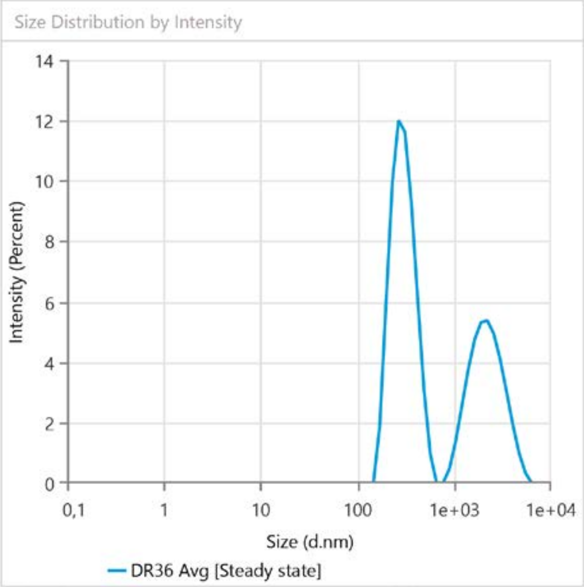

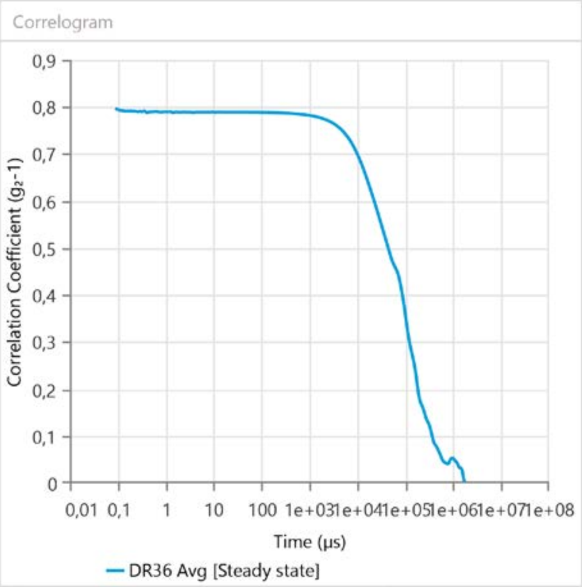

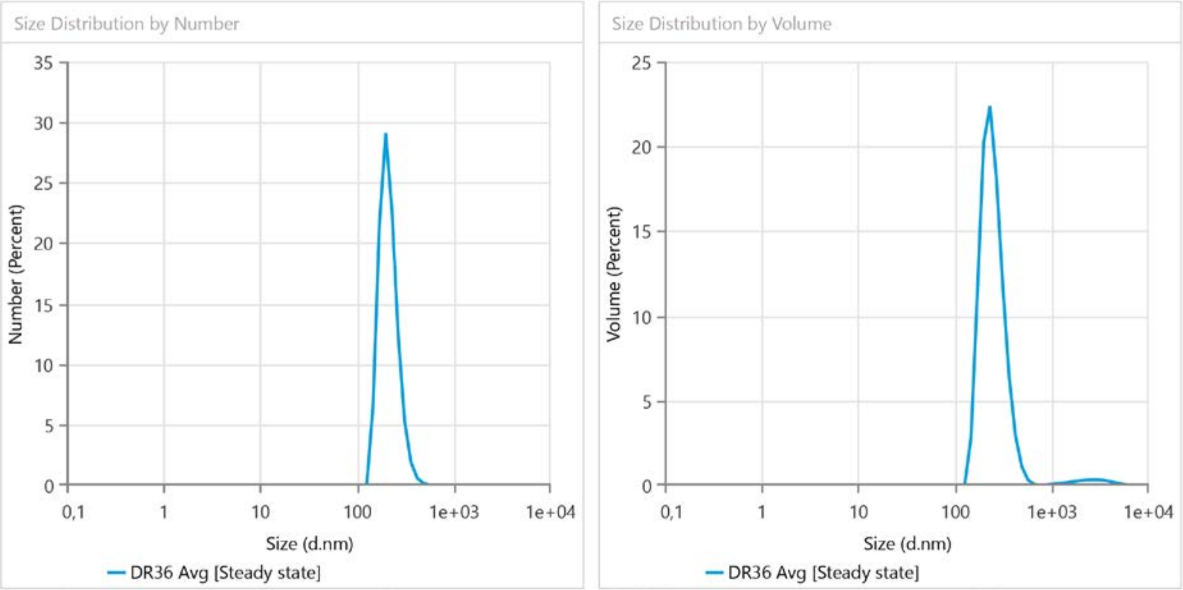

Parameter List

**Z-Average (nm)**	: 436,8
**Polydispersity Index (PI)**	: 0,3908
**Intercept**	: 0,7276
**Peak One Mean by Intensity (nm)**	: 305,4
**Peak Two Mean by Intensity (nm)**	: 2307
**Peak Three Mean by Intensity (nm)**	:
**Peak One Mean by Number (nm)**	: 215
**Peak Two Mean by Number (nm)**	:
**Peak Three Mean by Number (nm)**	:
**Instrument Serial Number**	: MAL1259671
**Software Version**	: 2.0.1.1

Parameter List

**In Range (%)**	: 50,47
**Fit Error**	: 0,005904
**Run Retention (%)**	: 63,11
**Run Duration (s)**	: 1,68
**Number Of Runs**	: 25
**Derived Mean Count Rate (kcps)**	: 6108
**Attenuator**	: 10
**Cuvette Position (mm)**	: 4,64
**Detector Angle (°)**	: 17
**Seek Optimum Attenuator Position**	: True

Figure S40: DLS results, sample of 6 prepared in water 0.1 µg/mL.

As prepared, superposed, three repetitions:

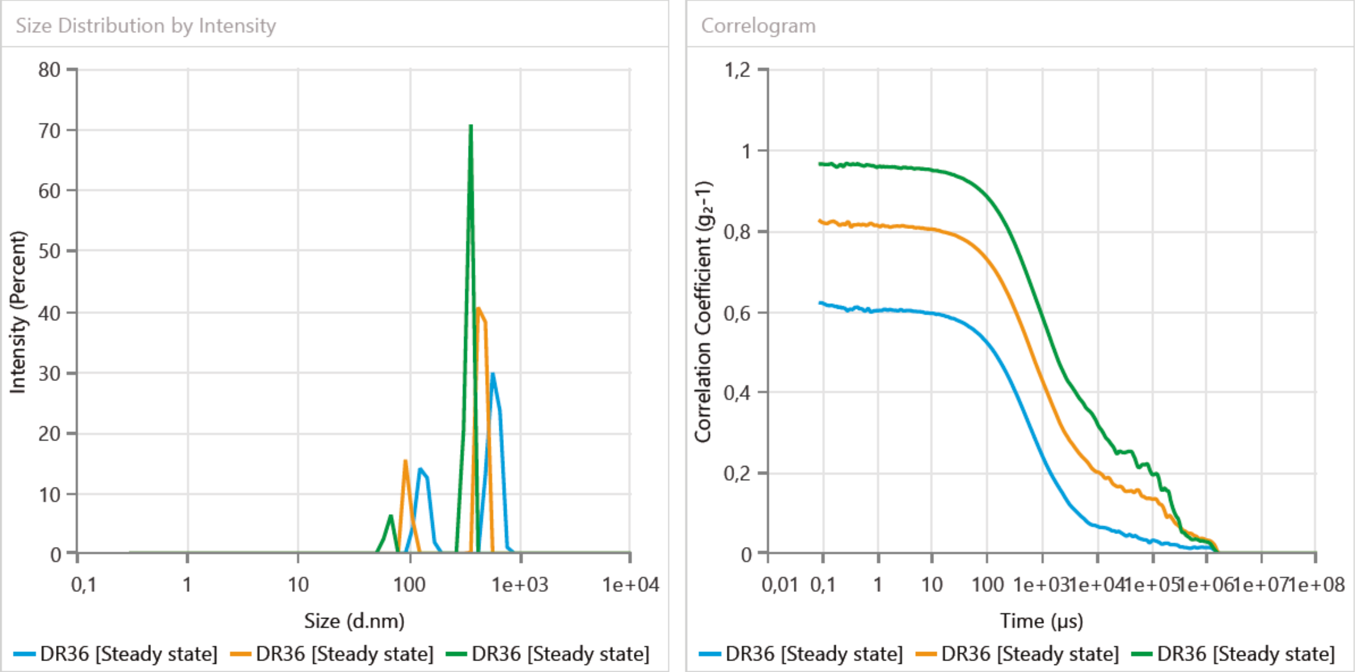

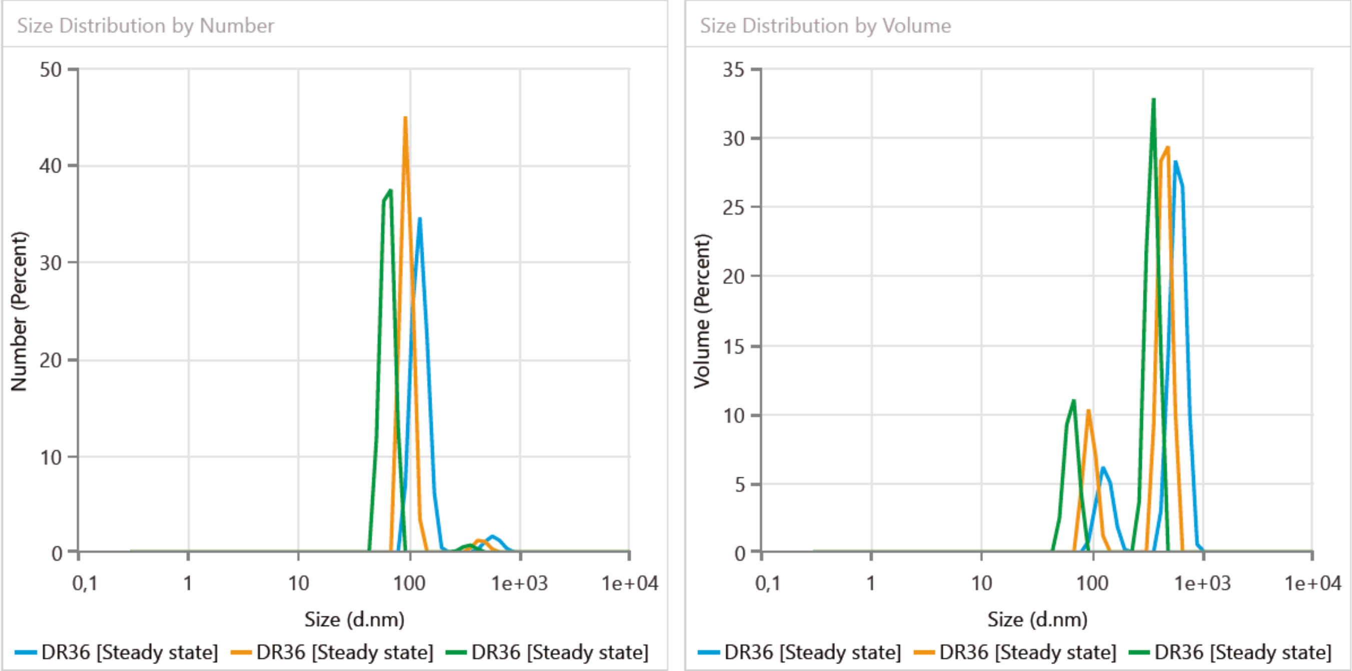

Parameter List

**Z-Average (nm)**	: 552
**Polydispersity Index (PI)**	: 0,6151
**Intercept**	: 0,7546
**Peak One Mean by Intensity (nm)**	: 588,4
**Peak Two Mean by Intensity (nm)**	: 133,7
**Peak Three Mean by Intensity (nm)**	:
**Peak One Mean by Number (nm)**	: 581,7
**Peak Two Mean by Number (nm)**	: 126,4
**Peak Three Mean by Number (nm)**	:
**Instrument Serial Number**	: MAL1259671
**Software Version**	: 2.0.1.1

Parameter List

**In Range (%)**	: 84,82
**Fit Error**	: 0,007677
**Run Retention (%)**	: 88
**Run Duration (s)**	: 1,68
**Number Of Runs**	: 25
**Derived Mean Count Rate (kcps)**	: 367,8
**Attenuator**	: 11
**Cuvette Position (mm)**	: 4,64
**Detector Angle (°)**	: 173
**Seek Optimum Attenuator Position**	: True

Figure S41: DLS results, sample of 6 prepared in water 0.1 μg/mL.

Filtered, 0.45 μm:

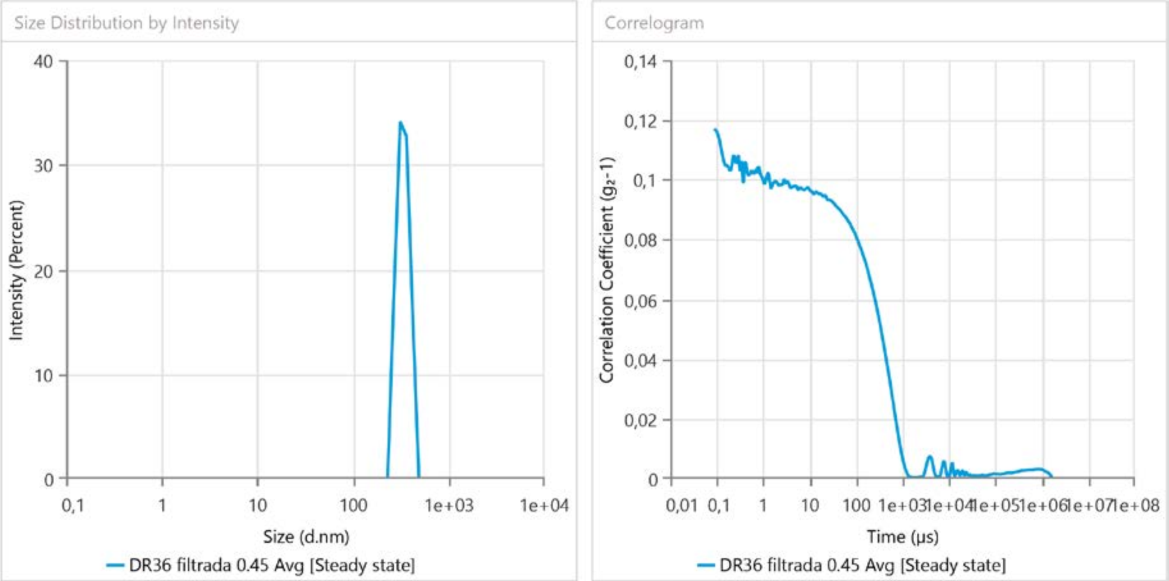

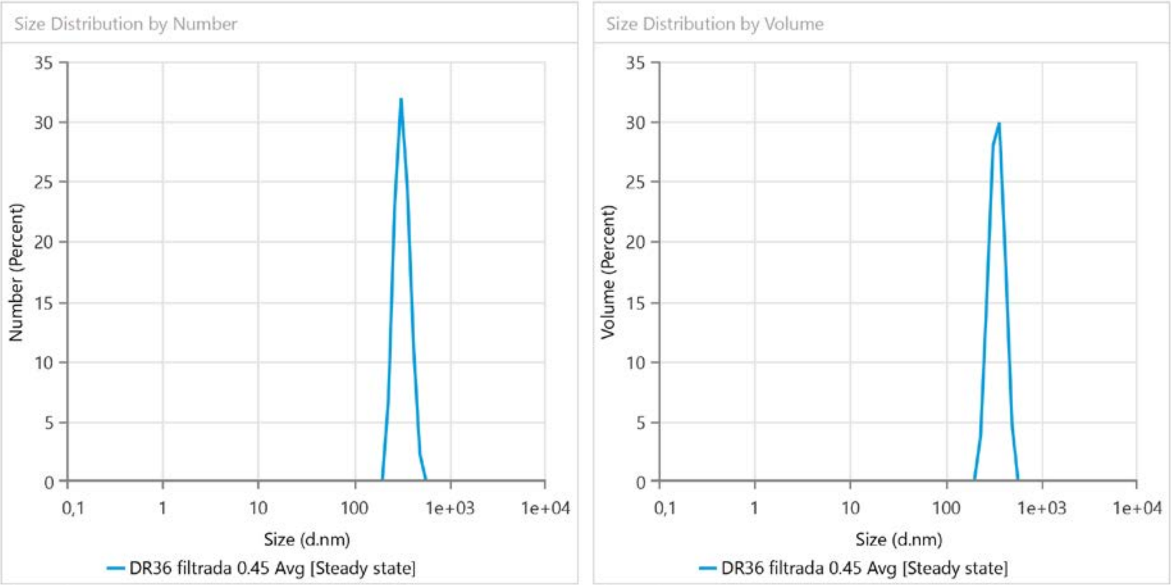

Parameter List

**Z-Average (nm)**	: 370,6
**Polydispersity Index (PI)**	: 0,8296
**Intercept**	: 0,3109
**Peak One Mean by Intensity (nm)**	: 336,6
**Peak Two Mean by Intensity (nm)**	:
**Peak Three Mean by Intensity (nm)**	:
**Peak One Mean by Number (nm)**	: 323,7
**Peak Two Mean by Number (nm)**	:
**Peak Three Mean by Number (nm)**	:
**Instrument Serial Number**	: MAL1259671
**Software Version**	: 2.0.1.1

Parameter List

**In Range (%)**	: 91,44
**Fit Error**	: 0,001611
**Run Retention (%)**	: 99,05
**Run Duration (s)**	: 1,68
**Number Of Runs**	: 35
**Derived Mean Count Rate (kcps)**	: 239
**Attenuator**	: 11
**Cuvette Position (mm)**	: 3
**Detector Angle (°)**	: 173
**Seek Optimum Attenuator Position**	: True

Figure S42: DLS results, sample of 6 prepared in water 0.1 μg/mL, filtered 0.45 μm.

Filtered, 0.22 μm:

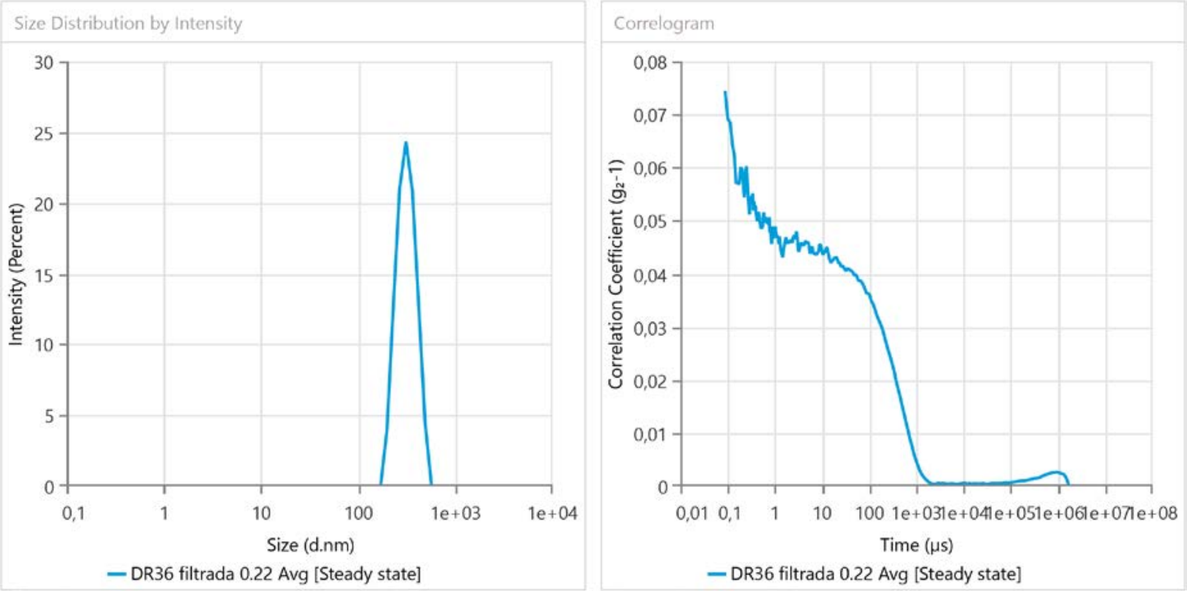

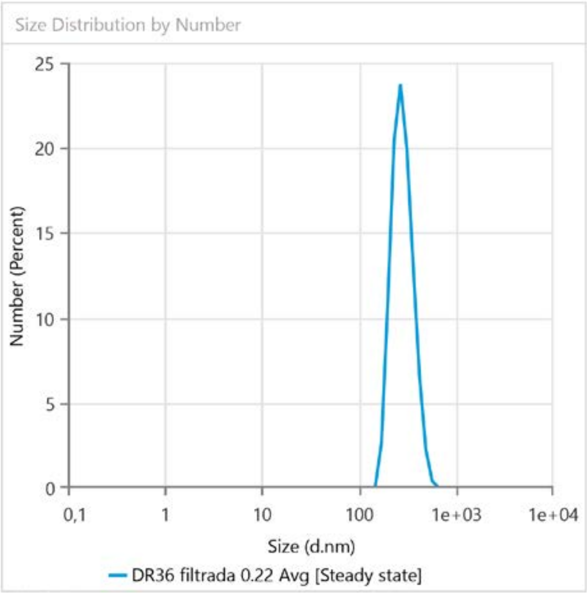

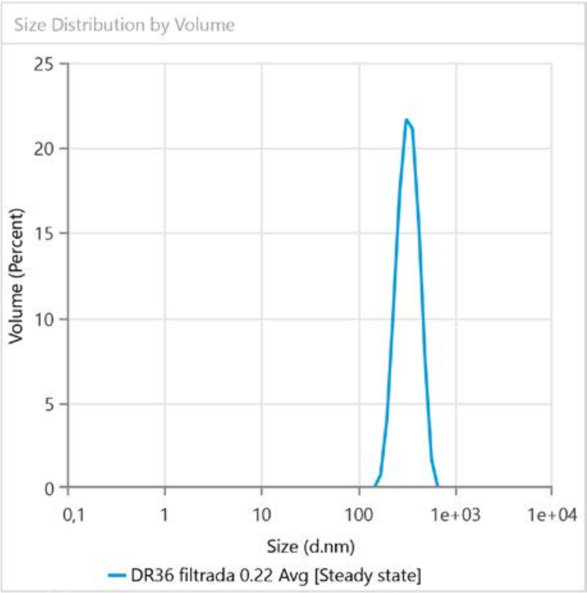

Parameter List

**Z-Average (nm)**	: 292,9
**Polydispersity Index (PI)**	: 0,0045
**Intercept**	: 0,2105
**Peak One Mean by Intensity (nm)**	: 319,2
**Peak Two Mean by Intensity (nm)**	:
**Peak Three Mean by Intensity (nm)**	:
**Peak One Mean by Number (nm)**	: 286,5
**Peak Two Mean by Number (nm)**	:
**Peak Three Mean by Number (nm)**	:
**Instrument Serial Number**	: MAL1259671
**Software Version**	: 2.0.1.1

Parameter List

**In Range (%)**	: 92,23
**Fit Error**	: 0,001936
**Run Retention (%)**	: 94,17
**Run Duration (s)**	: 1,68
**Number Of Runs**	: 40
**Derived Mean Count Rate (kcps)**	: 163,7
**Attenuator**	: 11
**Cuvette Position (mm)**	: 3
**Detector Angle (°)**	: 173
**Seek Optimum Attenuator Position**	: True

Figure S43: DLS results, sample of 6 prepared in water 0.1 µg/mL, filtered 0.22 µm.

Forward DLS (as prepared), three repetitions superposed:

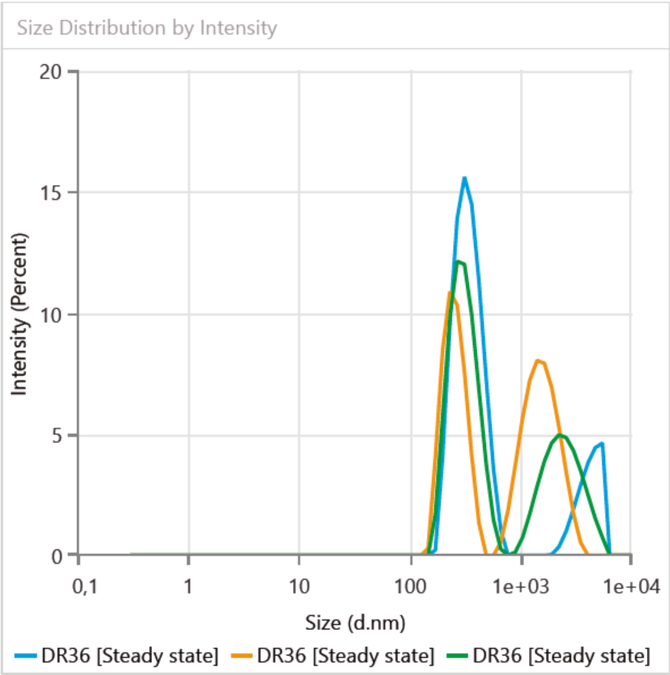

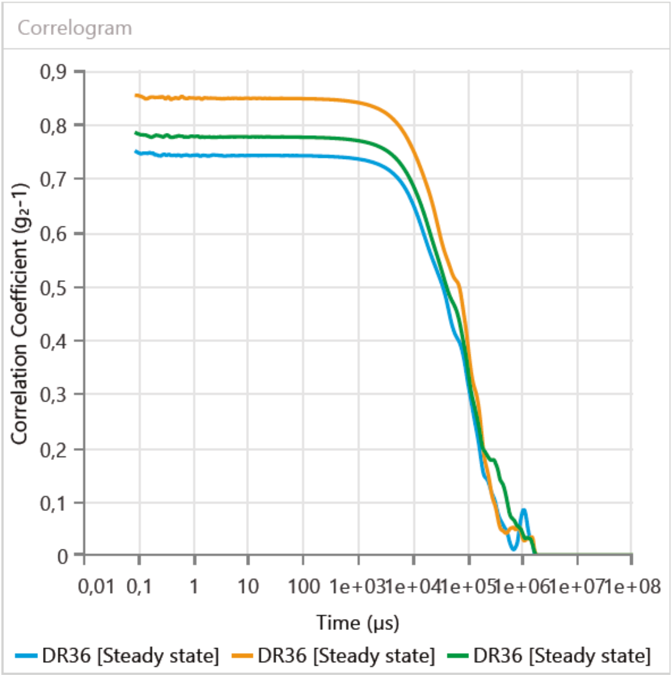

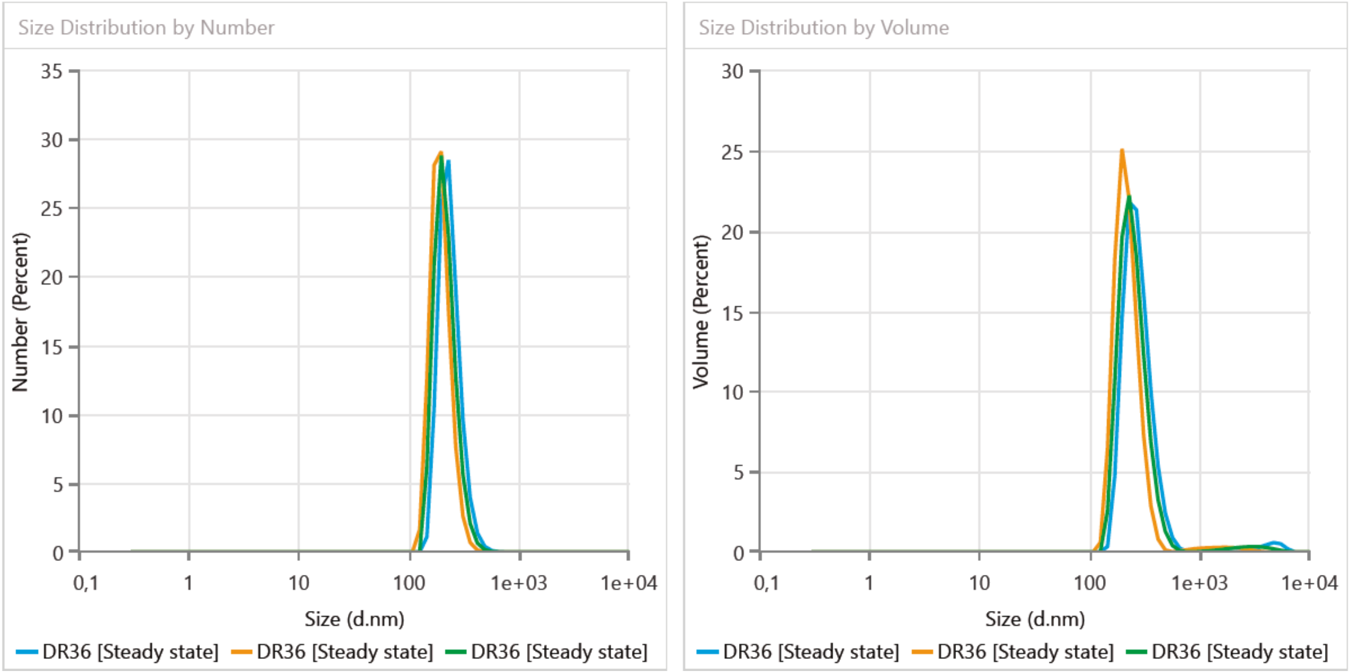

Parameter List

**Z-Average (nm)**	: 427,2
**Polydispersity Index (PI)**	: 0,3619
**Intercept**	: 0,7157
**Peak One Mean by Intensity (nm)**	: 343,7
**Peak Two Mean by Intensity (nm)**	: 4236
**Peak Three Mean by Intensity (nm)**	:
**Peak One Mean by Number (nm)**	: 238,6
**Peak Two Mean by Number (nm)**	:
**Peak Three Mean by Number (nm)**	:
**Instrument Serial Number**	: MAL1259671
**Software Version**	: 2.0.1.1

Parameter List

**In Range (%)**	: 52,66
**Fit Error**	: 0,005229
**Run Retention (%)**	: 73,33
**Run Duration (s)**	: 1,68
**Number Of Runs**	: 15
**Derived Mean Count Rate (kcps)**	: 6908
**Attenuator**	: 10
**Cuvette Position (mm)**	: 4,64
**Detector Angle (°)**	: 17
**Seek Optimum Attenuator Position**	: True

Figure S44: DLS results, sample of 6 prepared in water 0.1 µg/mL

Tests with cucurbiturils: The compound was dissolved in water. These tests resulted in a partial inhibition of the fluorescence developed on time by **6** as the experiments are developed.

6 CB[5] CB[6] CB[7] CB[8] 6 CB[5] CB[6] CB[7] CB[8]

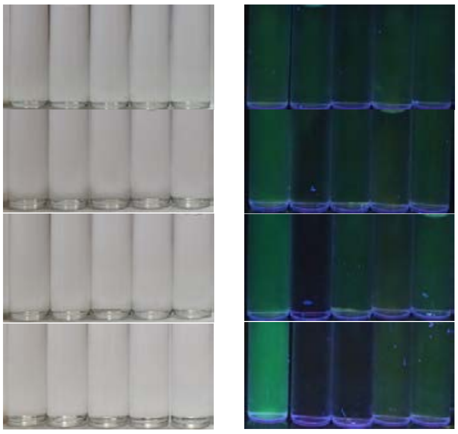

1:1

1:5

1:10

1:20

Figure S45: Photos were taken under white (left) and 366 nm (right) lights. From top to bottom, molar proportion between dye and cucurbituril of 1:1, 1:5, 1:10 and 1:20. Each tube in every image corresponds (from left to right) to: dye, dye with CB[5], CB[6], CB[7] and CB[8].

Further tests with cucurbiturils [5] and [6]: The compound was dissolved in water. These tests compared the sensitivity of **6** to different concentrations of CB[5] and CB[6].

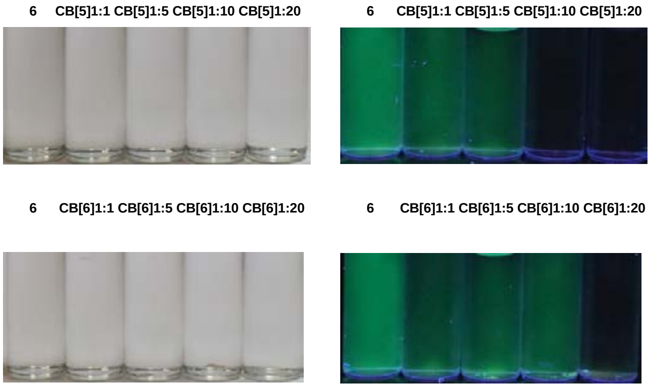

Figure S46: Photos were taken under white (left) and 366 nm (right) lights. From top to bottom, molar proportion between dye and cucurbituril of 1:1, 1:5, 1:10 and 1:20. Each tube in every image corresponds (from left to right) to: dye, dye with CB[5] 1:1, CB[5] 1:5, CB[5] 1:10 and CB[5] 1:20 (upper row) and dye, dye with CB[6] 1:1, CB[6] 1:5, CB[6] 1:10 and CB[6] 1:20 (lower row).

Test with cations, anions, oxidizing and reducing agents: The compound was dissolved in water. These tests resulted **positive** for **6** with SO_4^{2-} (from the first addition) and negative for the rest.

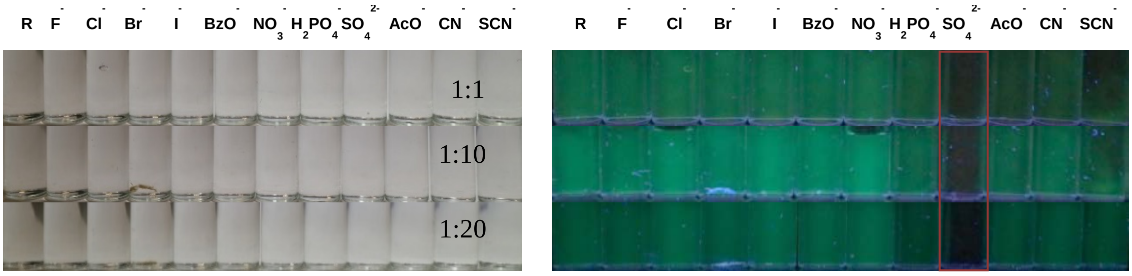

Figure S47: Photos were taken under white (left) and 366 nm (right) lights. From top to bottom, molar proportion between dye and anions of 1:1, 1:10 and 1:20. Each tube in every image corresponds (from left to right) to: dye (R), dye with F^-, Cl^-, Br^-, I^-, BzO^-, NO_3^-, $H_2PO_4^-$, SO_4^{2-}, AcO^-, CN^- and SCN^-.

Test of pH effect: This test showed that compound was not affected by changes in pH values.

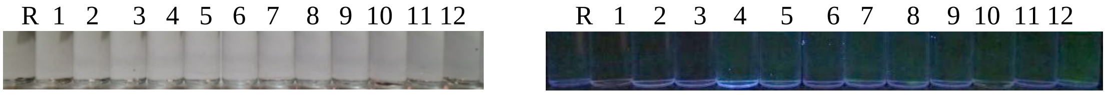

Figure S48: Photos were taken under white (left) and 366 nm (right) lights. Each tube in every image correspond (from left to right) to: dye (R), dye in buffer of pH 5.44 (1), 5.68 (2), 6.77 (3), 6.94 (4), 7.07 (5), 7.09 (6), 7.25 (7), 7.40 (8), 7.46 (9), 7.96 (10), 9.14 (11) and 10.49 (12).

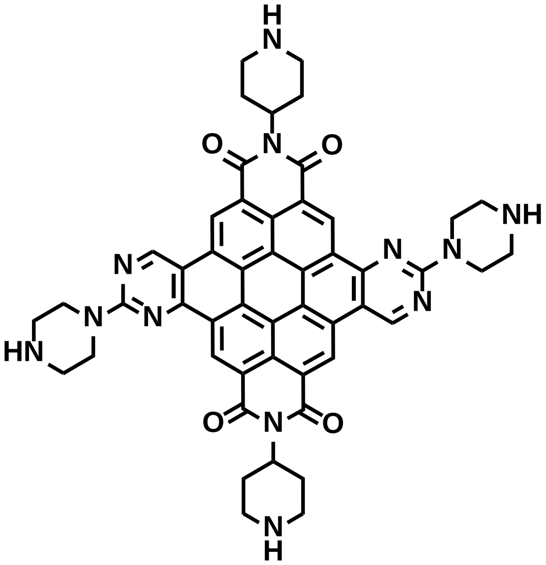

7

***N,N'*-Bis-(piperidin-4-yl)[3,4-e][9,10-e]bis-((1,4-piperazin-1-yl)-1,3-pyrimidin)coronene-1,12:6,7-tetracarboxylic diimide 7**. MP (ºC): > 350ºC. R_f (CH_2Cl_2:MeOH, 50:4): 0.01. FT-IR (KBr, cm^{-1}): 3437 (N-H, amine), 2959 (C-H, aromatic), 2921 (C-H, aliphatic), 2849 (C-H, aliphatic), 1685 (C=O, imide), 1606, 1569, 1524 (C=C), 1443 (C-N), 1358 (C-N), 1288, 1134 (C-N), 958. ^1H NMR (300 MHz, TFA) δ: 7.72 – 7.34 (m, 4H, Ar-H), 7.26 – 7.05 (m, 2H, Ar-H), 5.97 – 5.71 (m, 2H, N-CH), 5.02 – 4.82 (m, 8H, CH_2), 4.09 – 3.94 (m, 12H, CH_2), 3.62 – 3.45 (m, 8H, CH_2), 2.47 – 2.32 (m, 4H, CH_2), 0.91 – 0.84 (m, 4H, CH_2). HR-MS (MALDI+, DCTB): m/z calcd. for $C_{50}H_{45}N_{12}O_4$ ($[M+H]^+$): 877.3681; found: 877.4124. MS (MALDI+, DCTB) 2[7]: m/z calcd. for 2($C_{50}H_{45}N_{12}O_4$) ($[2M+H]^+$): 1754; found: 1757. HR-MS (MALDI+, DCTB) [7]+CB[7]: m/z calcd. for ($C_{50}H_{45}N_{12}O_4$+$C_{42}H_{42}N_{28}O_{14}$) ($[M+CB[7]+H]^+$): 2039.7117; found: 2039.7148. MS (MALDI+, DCTB) [7]+CB[7]: m/z calcd. for ($C_{50}H_{45}N_{12}O_4$+2$C_{42}H_{42}N_{28}O_{14}$) ($[M+2CB[7]+H]^+$): 3203; found: 3208. UV-VIS (H_2O) λ_{max} / nm (ε / $M^{-1}\cdot cm^{-1}$): 550 (1819). Emission (H_2O, λ_{ex} = 369 nm) [7]+CB[7] λ_{max} / nm: 547, 587. τ / ns (H_2O, $\chi2$) [7]+CB[7] (1:100 in mol/mol): 6.20 (1.03). Φ (H_2O, λ_{ex} = 445 nm) [7]+CB[7] (1:100 in mol): 0.20 ± 0.01. Emission (H_2O, λ_{ex} = 369 nm) [7]+TNB (1:150 in mol/mol) λ_{max} / nm: 632. τ / ns (H_2O, $\chi2$) [7]+TNB (1:150 in mol/mol): 4.95 (1.12). Φ (H_2O, λ_{ex} = 445 nm) [7]+TNB (1:150 in mol): 0.02 ± 0.01. Emission (H_2O, λ_{ex} = 369 nm) [7]+TNT (1:150 in mol/mol) λ_{max} / nm: 595. τ / ns (H_2O, $\chi2$) [7]+TNT (1:150 in mol): 4.62 (1.26). Φ (H_2O, λ_{ex} = 445 nm) [7]+TNT (1:150 in mol): 0.03 ± 0.01.

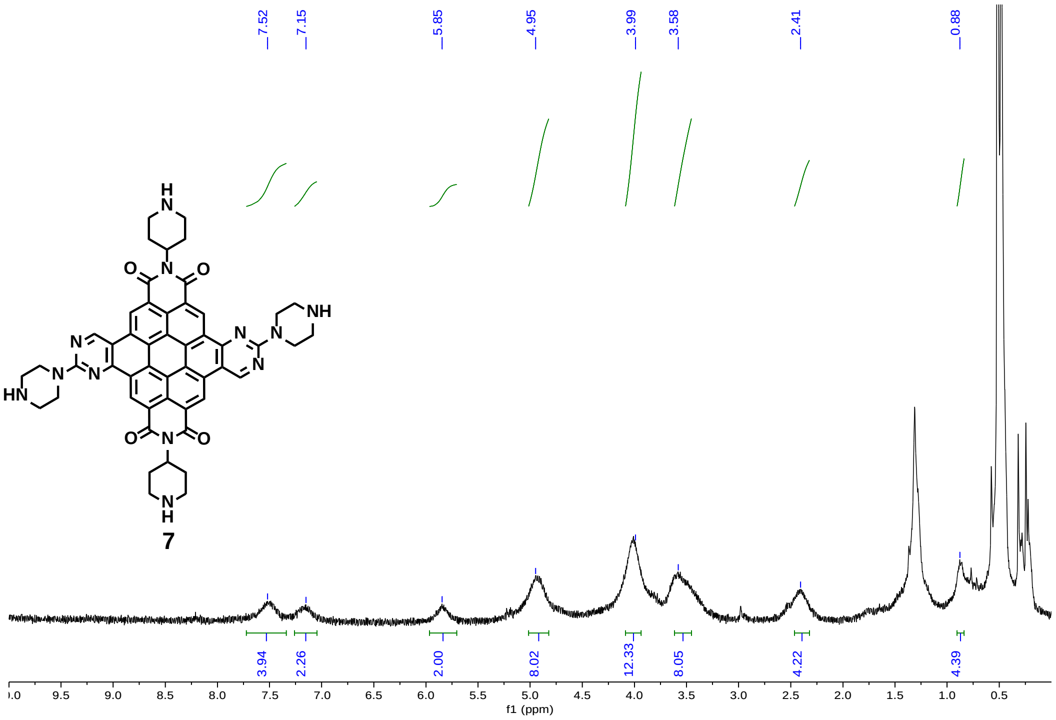

Figure S49: ^{1}H NMR (300 MHz, DTFA) of 7.

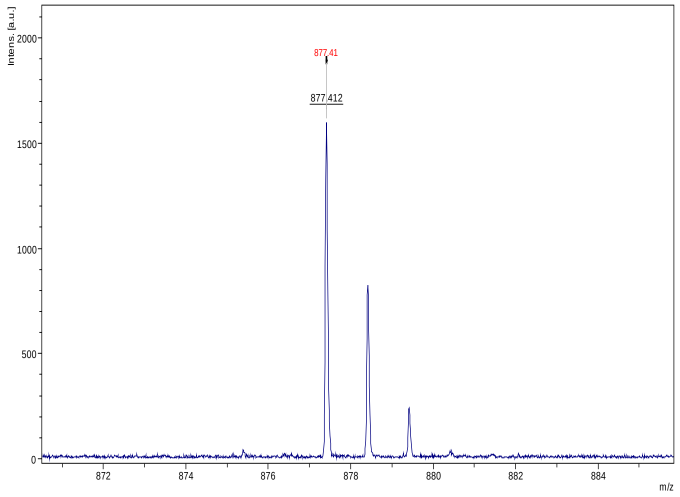

Figure S50: HRMS (MALDI-, DCTB) of 7.

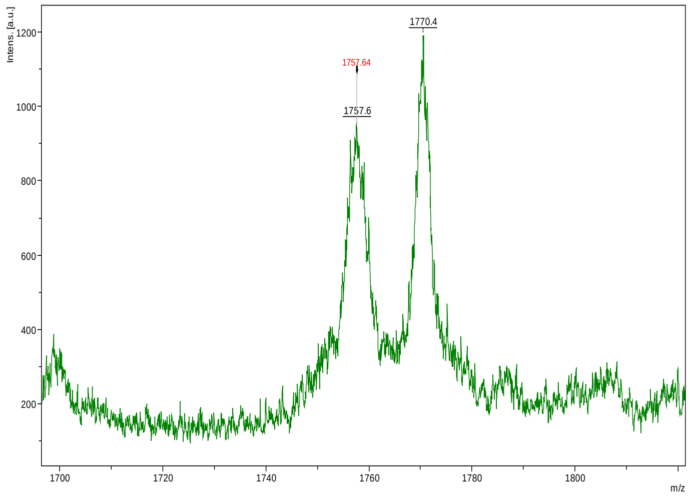

Figure S51: HR-MS (MALDI+, DCTB) of 2[7] stacked.

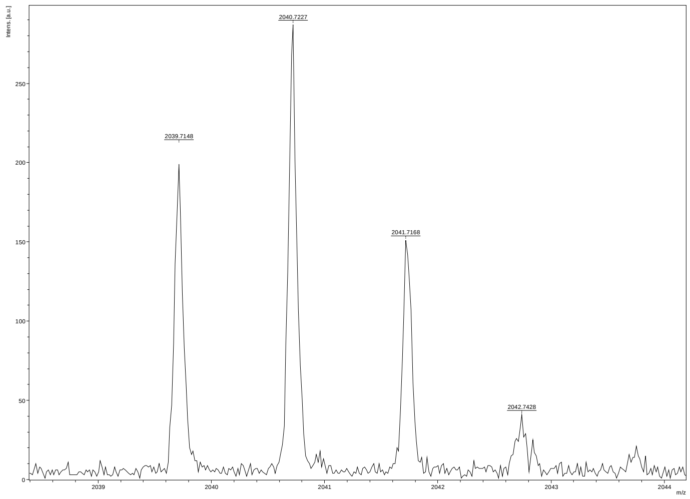

Figure S52: HRMS (MALDI+, DCTB) 7 + 1CB[7]

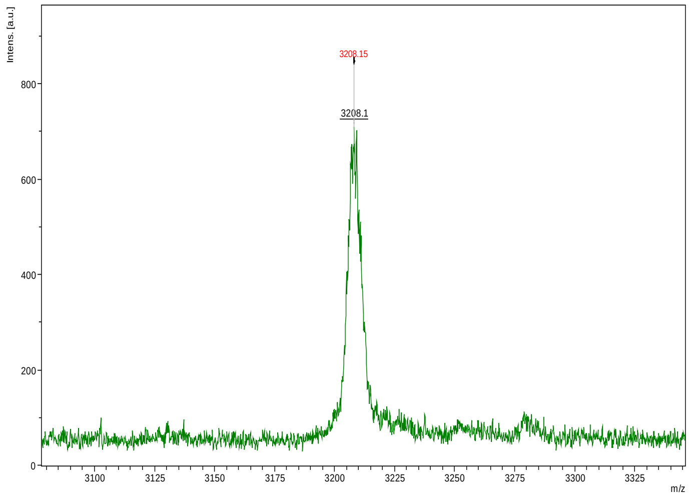

Figure S53: HRMS (MALDI+, DCTB) 7 + 2CB[7]

Solvatochromism tests

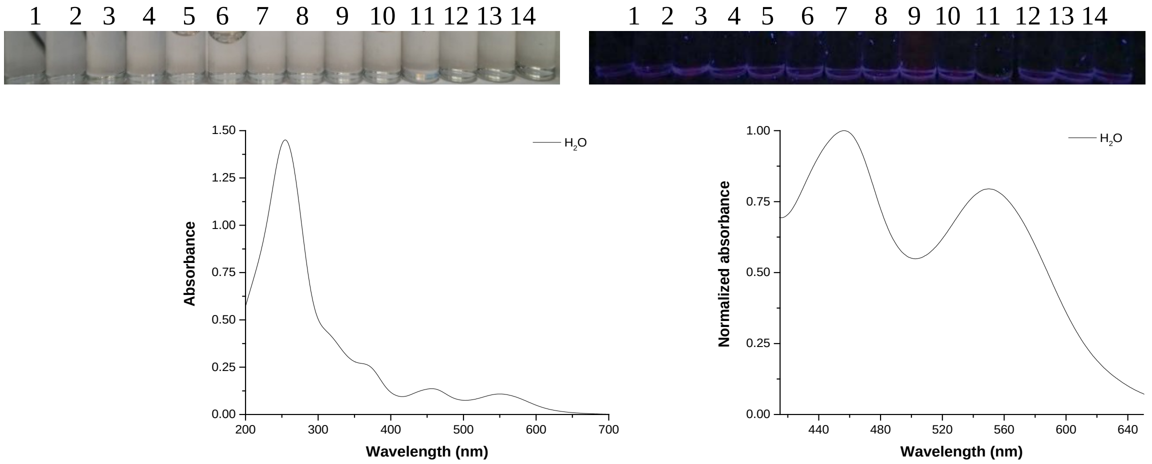

Figure S54: Solvatochromism test of 7: Up: Photos under white and 366 nm lights. The employed solvents were: 1: H_2O, 2: MeOH (methanol), 3: DMSO (dimethylsulfoxide), 4: DMF (*N*,*N'*-dimethylformamide), 5: MeCN (acetonitrile), 6: Acetone, 7: EtOAc (ethyl acetate), 8: THF (tetrahydrofuran), 9: $CHCl_3$, 10: CH_2Cl_2 (dichloromethane), 11: Toluene, 12: Et_2O (diethyl ether), 13: *n*-Hx (hexane), 14: *c*-Hx (cyclohexane). Down: Absorption and normalized absorption spectrum (left) in water.

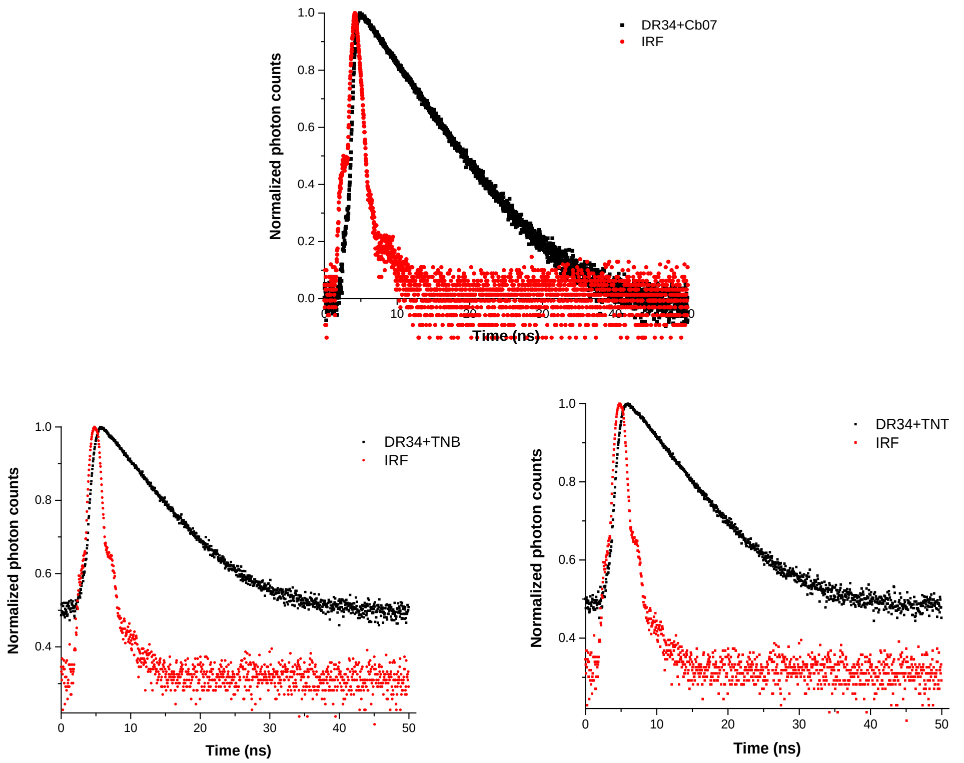

Figure S55: Normalized emission lifetime decay curves of 7 with CB[7] (1:100 in mol) (black) and blank (red), 7 with TNB (1:150 in mol) (black) and blank (red) and 7 with TNT (1:150 in mol) (black) and blank (red). The lasers employed were 375 nm (a) and 445 nm (rest) and the emission wavelengths 550 nm (b), 632 nm (c) and 592 nm (d).

Lambert-Beer study of 7: Compound was dissolved in water, in a concentration range of 5 to 1000 µM (11 points). Absorbance and emission (excitation wavelength 369 nm) were measured for each concentration. Selected work concentration was 10 µM.

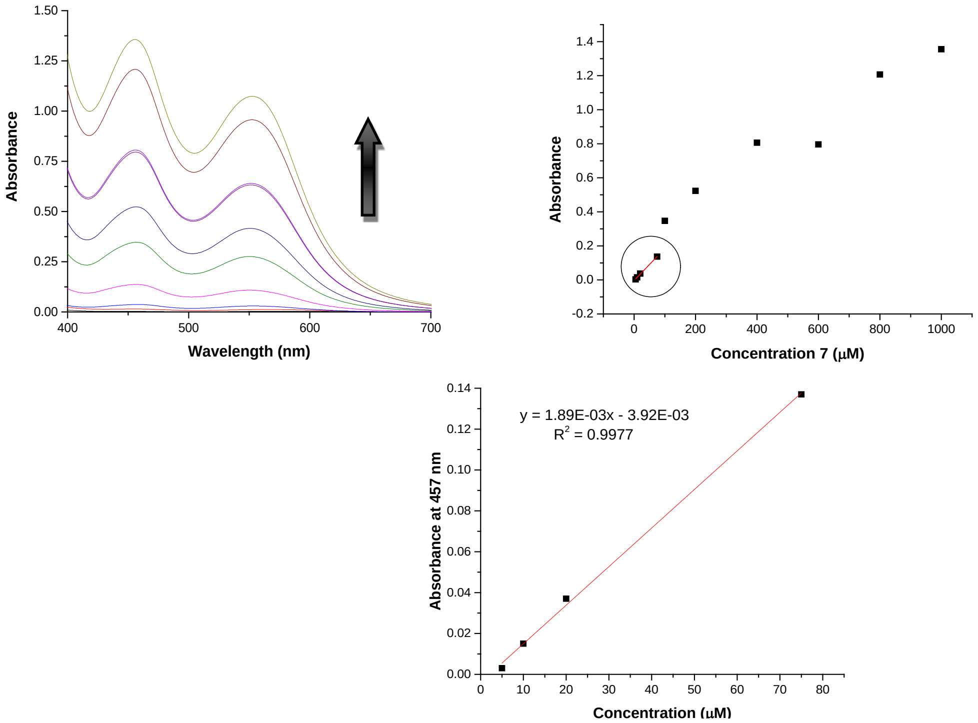

Figure S56: Absorbance spectra at different concentrations and representation of absorbance maxima versus concentration.

Kinetic effect study: Compound **7** was dissolved in water 10 µM. Absorbance and emission (excitation wavelength 457 nm) were measured for 270 minutes with a rate of 1 spectrum by every 3 minutes. No significant changes in the spectra were appreciated during the study.

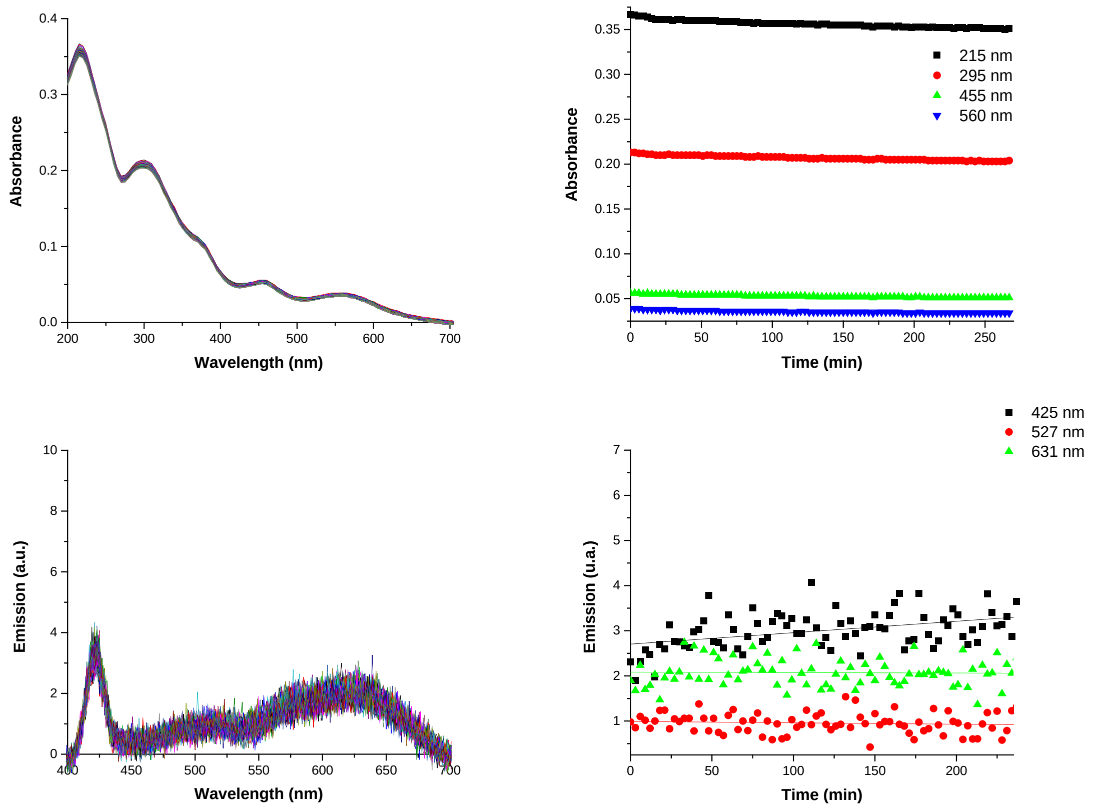

Figure S57: Up: Left: Absorbance spectra at different time points. Right: Representation of absorbance maxima along the time. Down: Left: Emission spectra at different times. Right: Representation of emission maxima along the time.

Morphology of 7: AFM images of samples prepared in water at different concentrations. Solutions were prepared freshly or five days before AFM study.

**Solution**				
**Freshly prepared**			**Prepared after five days**	
**0.1 µg/mL**	**1 µg/mL**	**0.1 mg/mL**	**0.1 µg/mL**	**1 µg/mL**

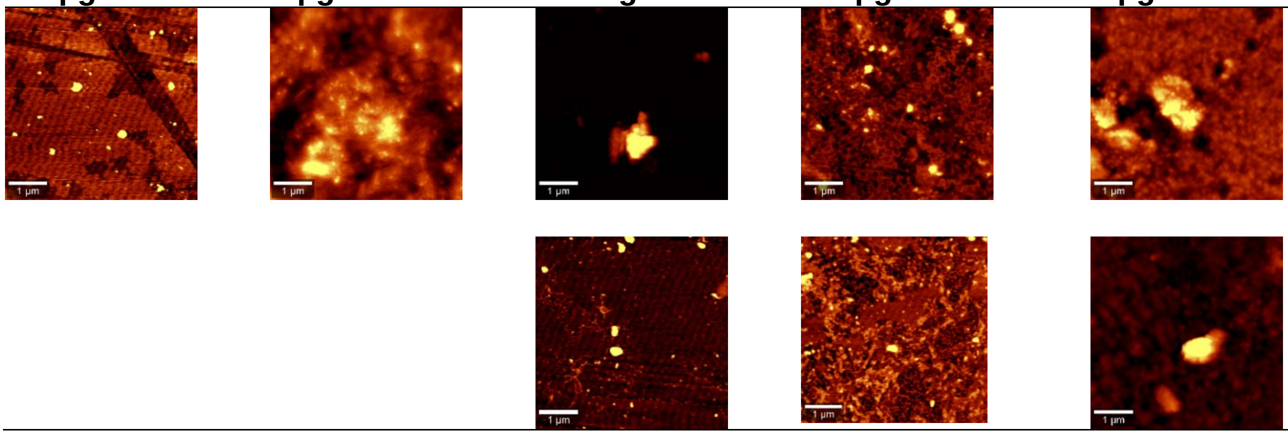

Figure S58: AFM images of samples of 7 in water

DLS (Dynamic Light Scattering) study, samples prepared in water 0.1 μg/mL:
As prepared:

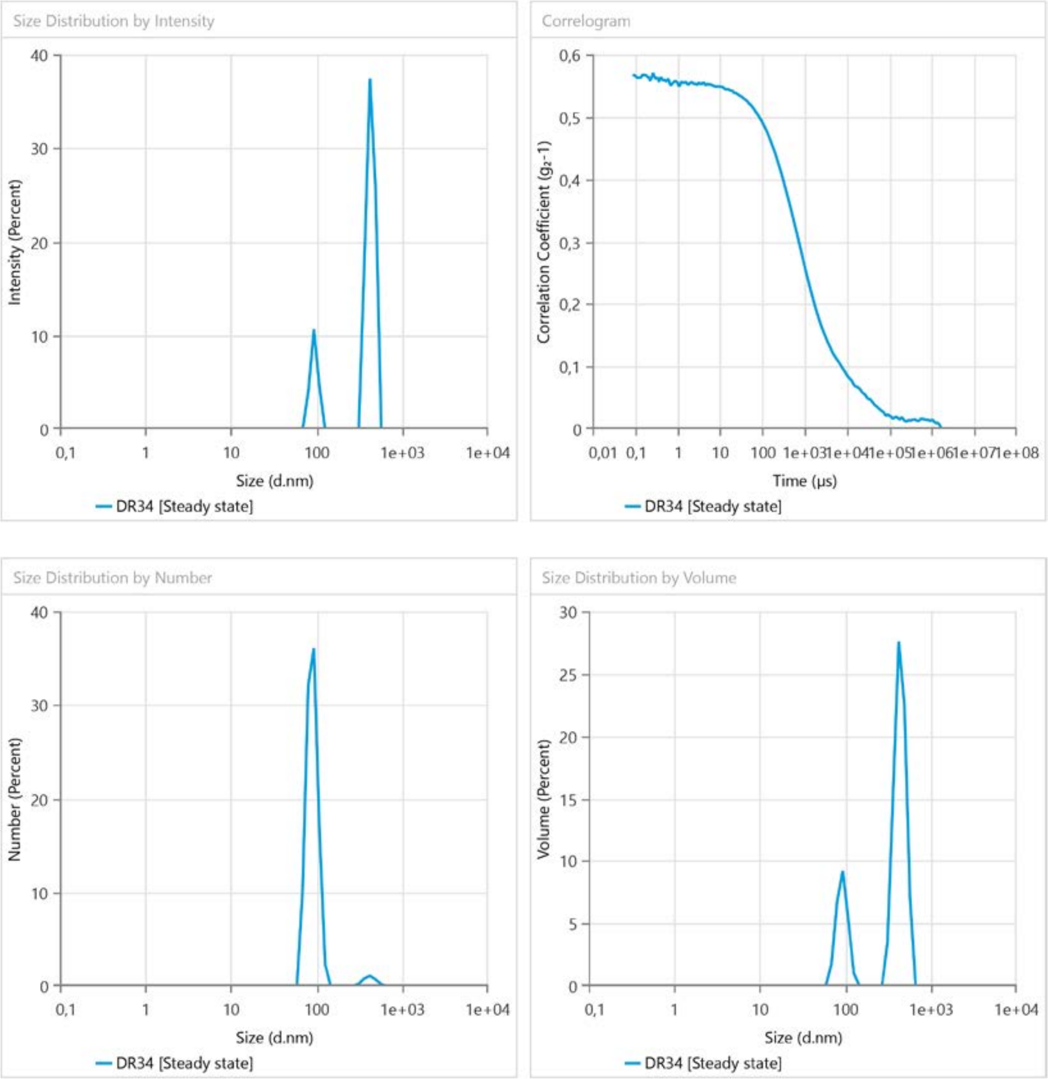

Parameter List

**Z-Average (nm)**	: 718,2
**Polydispersity Index (PI)**	: 0,6788
**Intercept**	: 0,7274
**Peak One Mean by Intensity (nm)**	: 428,8
**Peak Two Mean by Intensity (nm)**	: 93,75
**Peak Three Mean by Intensity (nm)**	:
**Peak One Mean by Number (nm)**	: 421,4
**Peak Two Mean by Number (nm)**	: 89,42
**Peak Three Mean by Number (nm)**	:
**Instrument Serial Number**	: MAL1259671
**Software Version**	: 2.0.1.1

Parameter List

**In Range (%)**	: 87,1
**Fit Error**	: 0,008954
**Run Retention (%)**	: 97,78
**Run Duration (s)**	: 1,68
**Number Of Runs**	: 45
**Derived Mean Count Rate (kcps)**	: 918,1
**Attenuator**	: 10
**Cuvette Position (mm)**	: 3
**Detector Angle (°)**	: 173
**Seek Optimum Attenuator Position**	: True

Figure S59: DLS results, sample of 7 prepared in water 0.1 μg/mL.

Filtered, 0.45 µm:

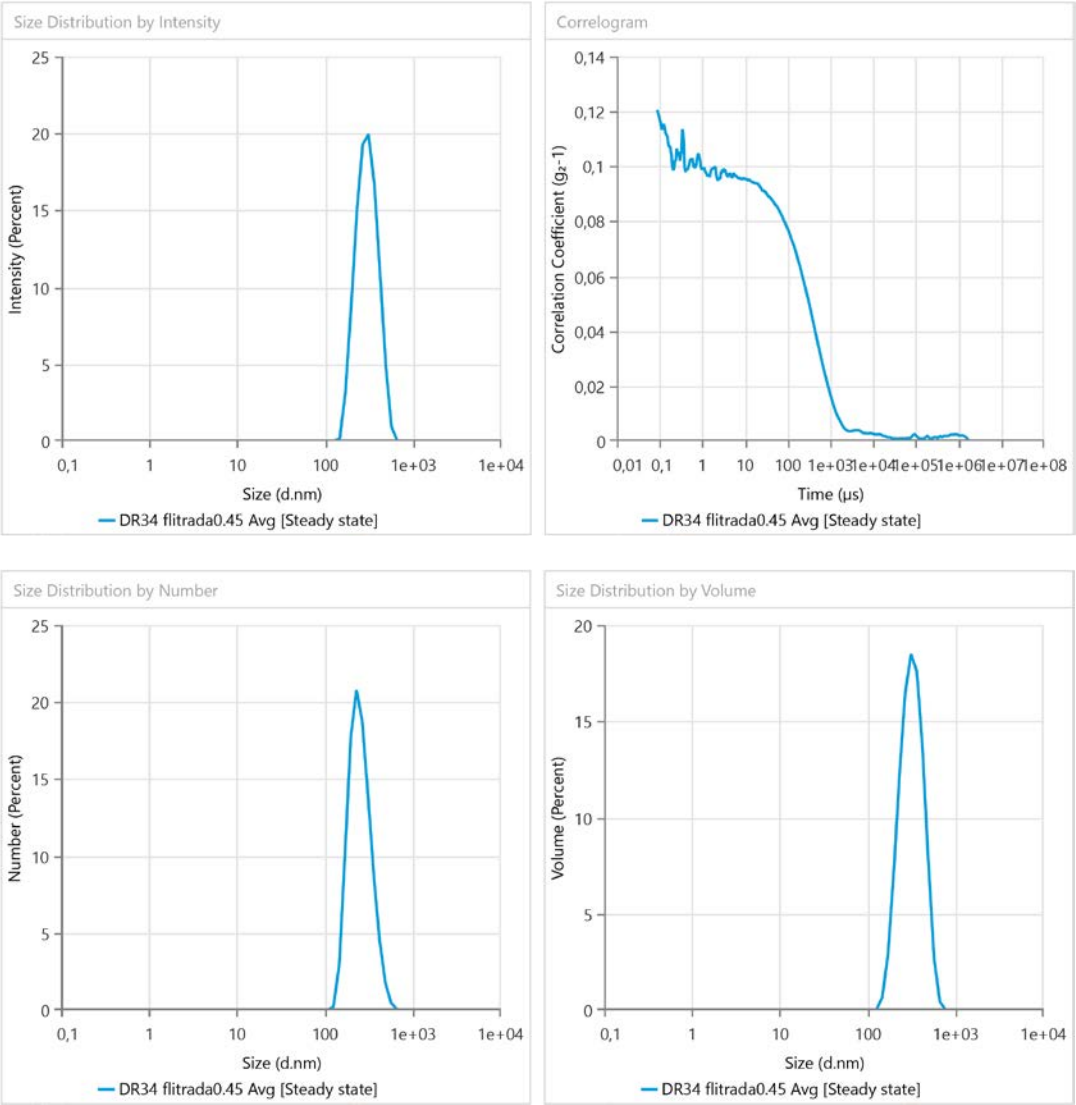

Parameter List

**Z-Average (nm)**	: 292,7
**Polydispersity Index (PI)**	: 0,3657
**Intercept**	: 0,3102
**Peak One Mean by Intensity (nm)**	: 306,8
**Peak Two Mean by Intensity (nm)**	:
**Peak Three Mean by Intensity (nm)**	:
**Peak One Mean by Number (nm)**	: 260,1
**Peak Two Mean by Number (nm)**	:
**Peak Three Mean by Number (nm)**	:
**Instrument Serial Number**	: MAL1259671
**Software Version**	: 2.0.1.1

Parameter List

**In Range (%)**	: 93,02
**Fit Error**	: 0,001483
**Run Retention (%)**	: 97,78
**Run Duration (s)**	: 1,68
**Number Of Runs**	: 25
**Derived Mean Count Rate (kcps)**	: 167,6
**Attenuator**	: 11
**Cuvette Position (mm)**	: 3
**Detector Angle (°)**	: 173
**Seek Optimum Attenuator Position**	: True

Figure S60: DLS results, sample of 7 prepared in water 0.1 µg/mL, filtered 0.45 µm.

Tests with cucurbiturils of 7: The compound was dissolved in water. These tests resulted **positive** for **7** with **CB[7]**.

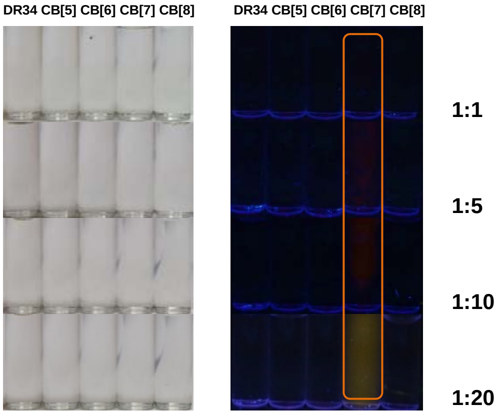

Figure S61: Photos were taken under white (left) and 366 nm (right) lights. From top to bottom, molar proportion between dye and cucurbituril of 1:1, 1:5, 1:10 and 1:20. Each tube in every image corresponds (from left to right) to: dye, dye with CB[5], CB[6], CB[7] and CB[8].

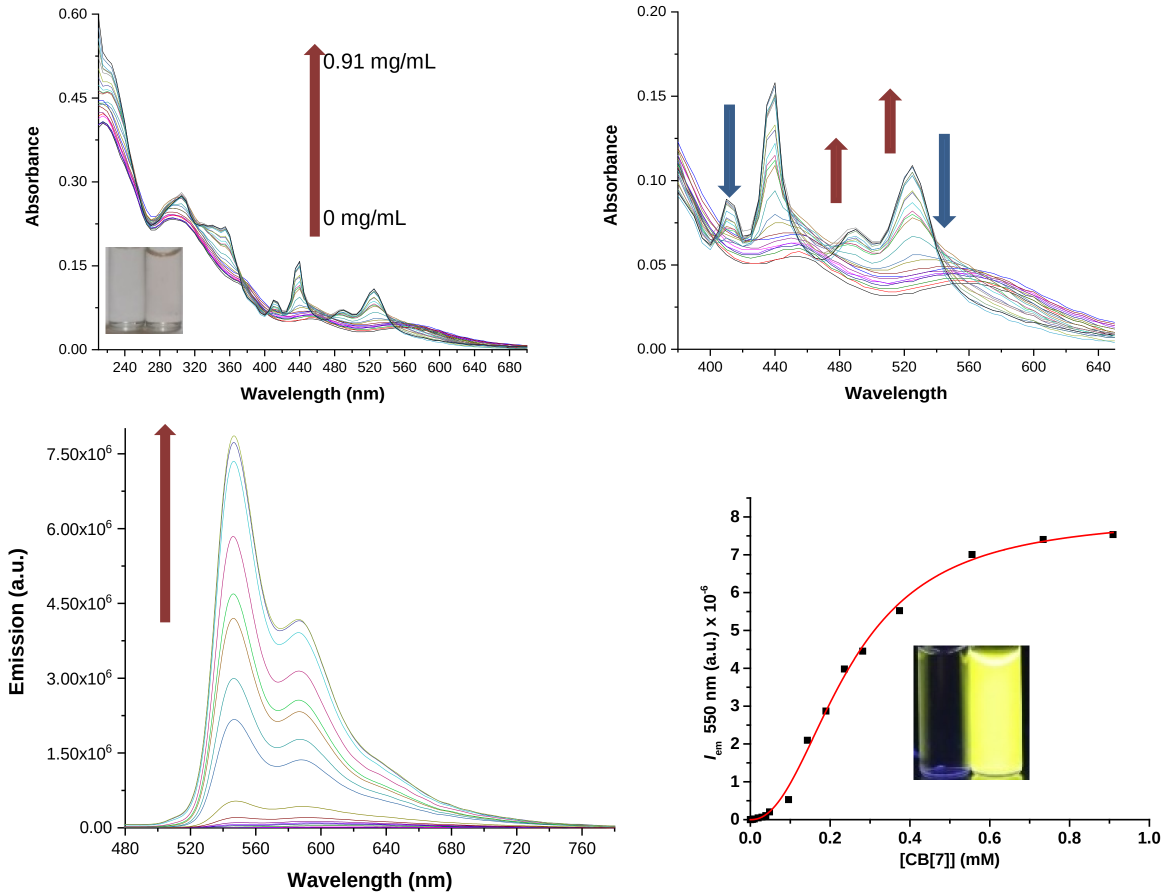

Figure S62: Full absorption titration curve and area magnification from 400 to 640 nm (top); full fluorescence titration curve (bottom left). Titration graph (bottom right) between 7 and CB[7] in water (10 μM 7, 0 to 0.91 mg/mL CB[7]). Insertion images: absorption of 7 (10 μM, top left) and after addition of 0.91 mg/mL CB[7] (top right), fluorescence of 7 (10 μM, bottom left), and after addition 0.91 mg/mL of CB [7] (bottom right).

Kinetic effect study with cucurbituril and 7: Compound was dissolved in water. Cucurbituril[7] was added until a molar proportion of 1:20 (**7**:CB[7]) Emission (excitation wavelength 457 nm) were measured for 80 minutes with a rate of 1 spectrum by every 3 minutes. No significant changes in the spectra were appreciated after 40 min.

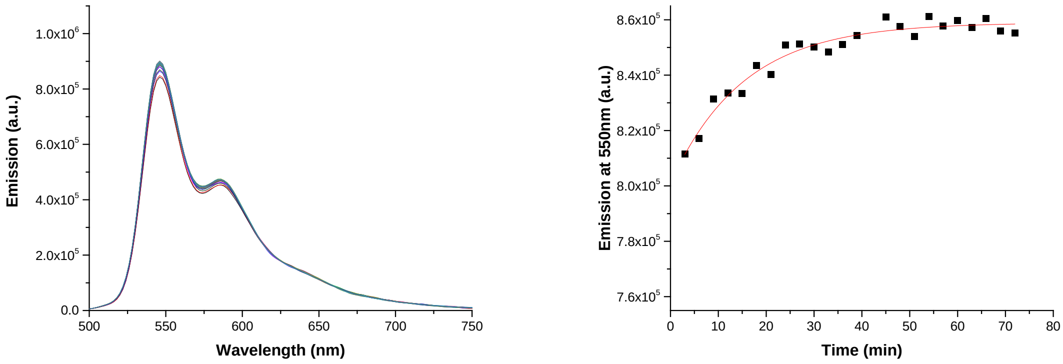

Figure S63: Left: Emission spectra at different time points. Right: Representation of emission maxima along the time.

Tests with cations, anions, oxidizing and reducing agents, in the presence of CB[7]: The compound was dissolved in water, 10 µM, with **CB[7]** as an additive (molar proportion 1:100, **7**:CB[7]). These tests resulted **positive** for **7**+CB[7] with Au(III), Pd(II) and Ir(III) (from the first addition) and **negative** for the rest.

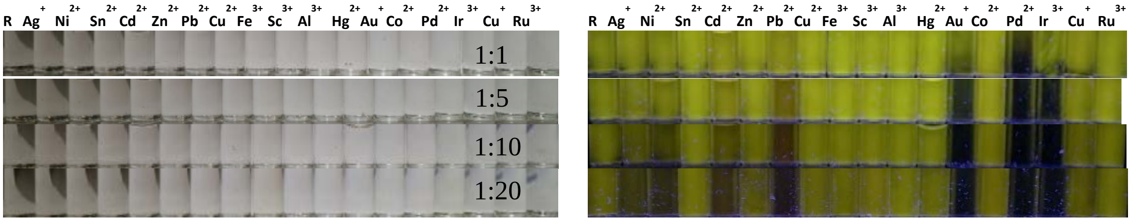

Figure S64A: Photos were taken under white (left) and 366 nm (right) lights. From top to bottom, molar proportion between dye and cations of 1:1, 1:5, 1:10 and 1:20. Each tube in every image corresponds (from left to right) to: dye (R), dye with Ag^{+}, Ni^{2+}, Sn^{2+}, Cd^{2+}, Zn^{2+}, Pb^{2+}, Cu^{2+}, Fe^{3+}, Sc^{3+}, Al^{3+}, Hg^{2+}, Au^{+}, Co^{2+}, Pd^{2+}, Ir^{3+}, Cu^{+} and Ru^{3+}.

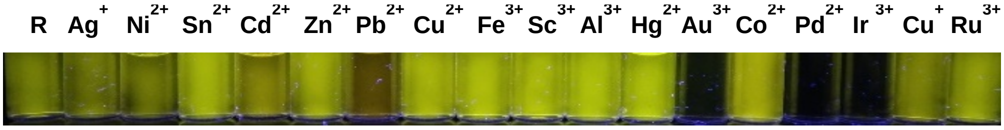

Figure S64B: Amplification of the fluorescence of 10 µM solutions of 7 in H_2O with a 1:5 proportion of every cation (λ_{exc} = 366 nm).

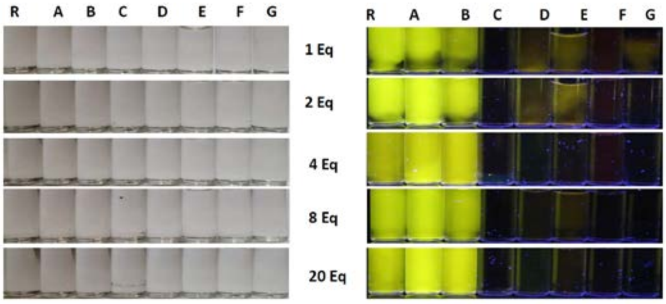

Figure S65: Photos were taken under white (left) and 366 nm (right) lights. From top to bottom, molar proportion between dye and oxidizing of 1:1, 1:2, 1:4, 1:8 and 1:20. Each tube in every image correspond (from left to right) to: dye (R), dye with HCl (A), HNO_3 (B), *m*-CPBA (C), oxone (D), hydrazine (E), TNB (F) and H_2O_2 (G).

Tests with cations, anions, oxidizing and reducing agents without the presence of CB[7]: The compound was dissolved in water, 10 µM. These tests resulted **positive** for **7** with TNB and **negative** for the rest. As an example of negative tests, the tests with cations are shown.

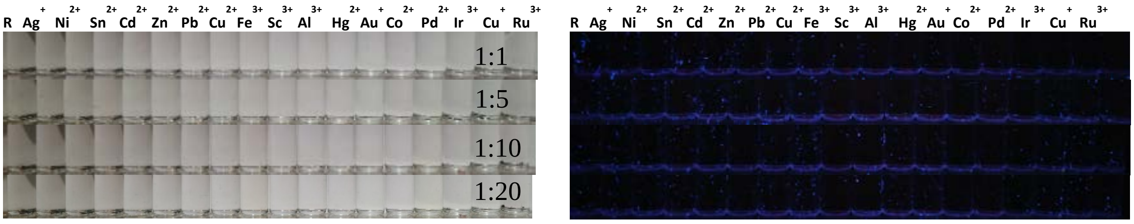

Figure S66: Photos were taken under white (left) and 366 nm (right) lights. From top to bottom, molar proportion between dye and cations of 1:1, 1:5, 1:10 and 1:20. Each tube in every image corresponds (from left to right) to: dye (R), dye with Ag^{+}, Ni^{2+}, Sn^{2+}, Cd^{2+}, Zn^{2+}, Pb^{2+}, Cu^{2+}, Fe^{3+}, Sc^{3+}, Al^{3+}, Hg^{2+}, Au^{+}, Co^{2+}, Pd^{2+}, Ir^{3+}, Cu^{+} and Ru^{3+}.

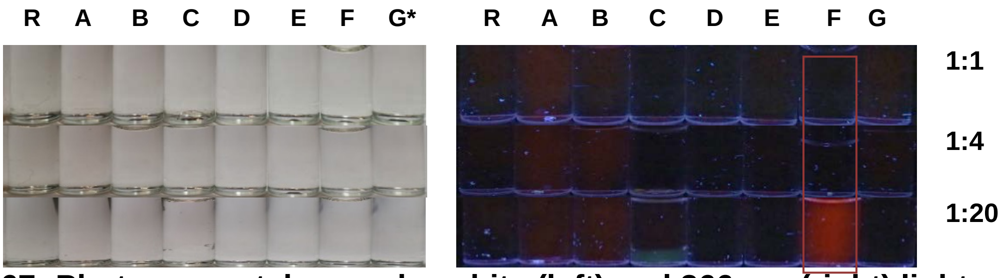

Figure S67: Photos were taken under white (left) and 366 nm (right) lights. From top to bottom, molar proportion between dye and oxidizing of 1:1, 1:4 and 1:20. Each tube in every image correspond (from left to right) to: dye (R), dye with HCl (A), HNO_3 (B), *m*-CPBA (C), oxone (D), hydrazine (E), TNB (F) and H_2O_2 (G).

UV-vis absorption effect of addition of TNB or TNT:

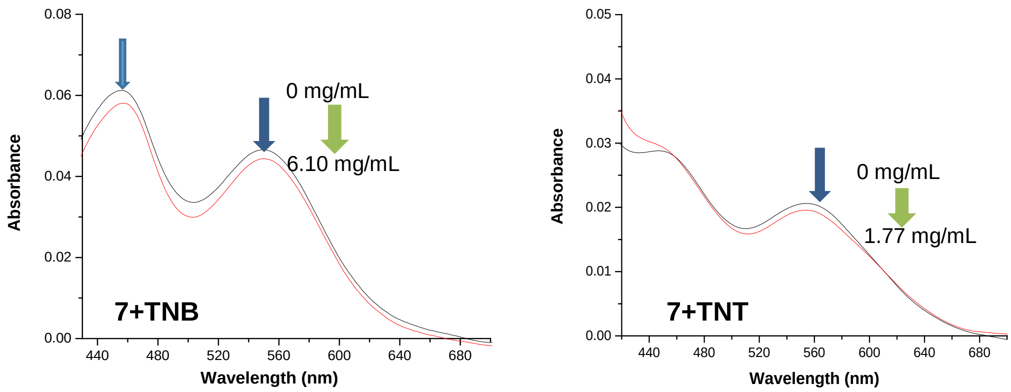

Figure S68: UV-vis absorption effect of addition of TNB or TNT on 7 solutions.

Kinetic effect study with TNB: Compound was dissolved in water, 10 µM. TNB was added until a molar proportion of 1:20 (**7**:TNB). Emission (excitation wavelength 435 nm) was measured for 145 minutes with a rate of 1 spectrum by 2.5 minutes. No significant changes in the spectra were appreciated during the study.

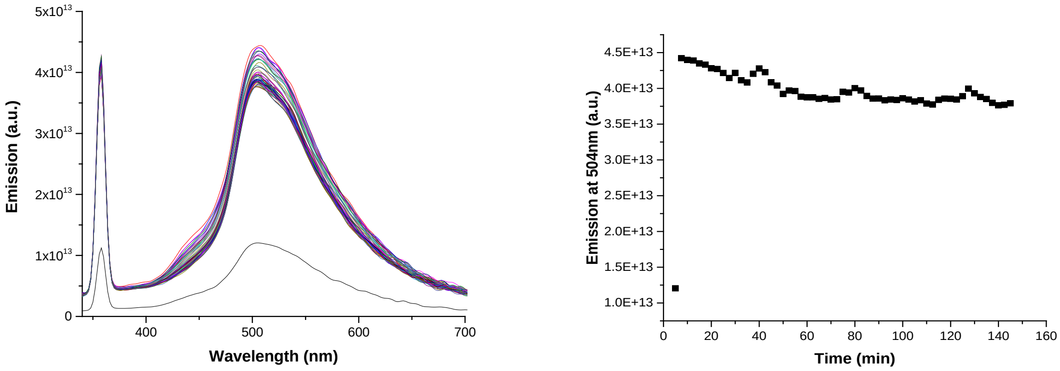

Figure S69: Left: Emission spectra at different time points. Right: Representation of emission maxima along the time.

Test of pH effect: 7 was not affected by changes in pH values.

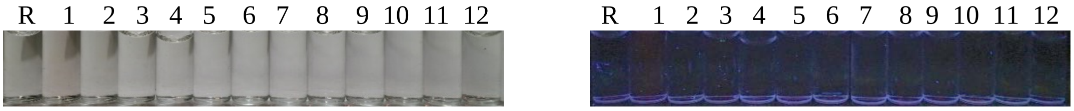

Figure S70: Photos were taken under white (left) and 366 nm (right) lights. Each tube in every image corresponds (from left to right) to: dye (R), dye in buffer of pH 5.44 (1), 5.68 (2), 6.77 (3), 6.94 (4), 7.07 (5), 7.09 (6), 7.25 (7), 7.40 (8), 7.46 (9), 7.96 (10), 9.14 (11) and 10.49 (12)

Emission spectra corresponding to the test of pH effect of compound 7.

The compound was dissolved in water at a concentration of 10µM and subjected to pH buffer solutions (HEPES) of pH between 5 and 11. Measurements correspond to dye (R), dye in buffer of pH 5.44 (1), 5.68 (2), 6.77 (3), 6.94(4), 7.07 (5), 7.09 (6), 7.25 (7), 7.40 (8), 7.46 (9), 7.96 (10), 9.14 (11) and 10.49 (12). Excitation wavelength was 369nm. **Figure S72** shows that the emission of compound **7** was not affected by changes in pH values.

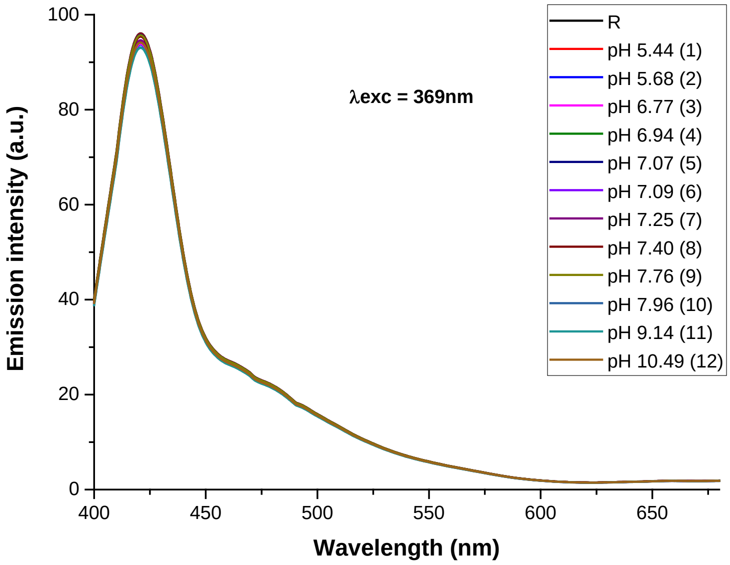

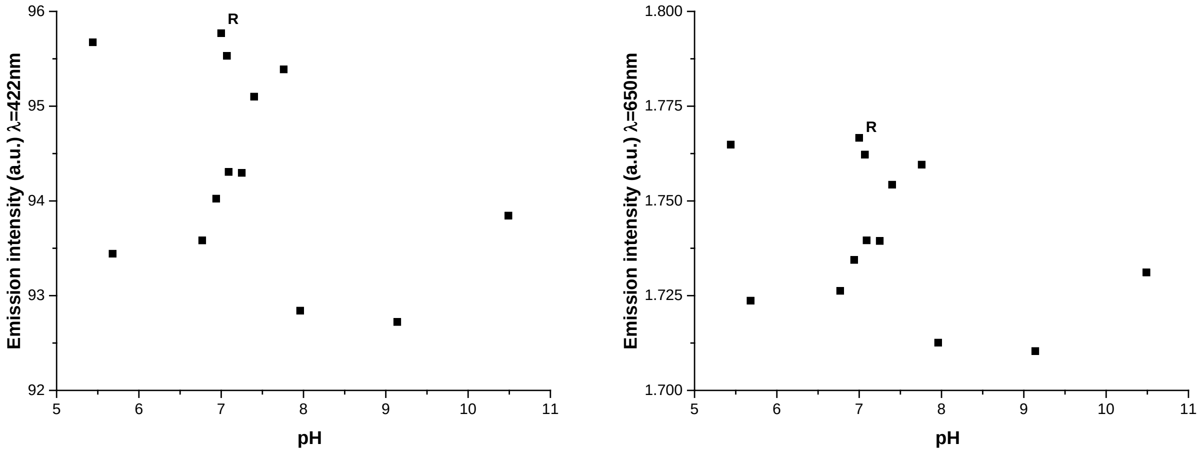

Figure S71. Emission spectra of 7 (10 μM) in increasing pH buffer solutions (up) and representation of emission versus pH at λ = 422 nm (down left) and 650nm (down right) λexc = 369 nm. The reference R in water was given an arbitrary pH = 7 to be introduced for comparative purposes.

Further interferent tests with some common feasible water-soluble interferents:

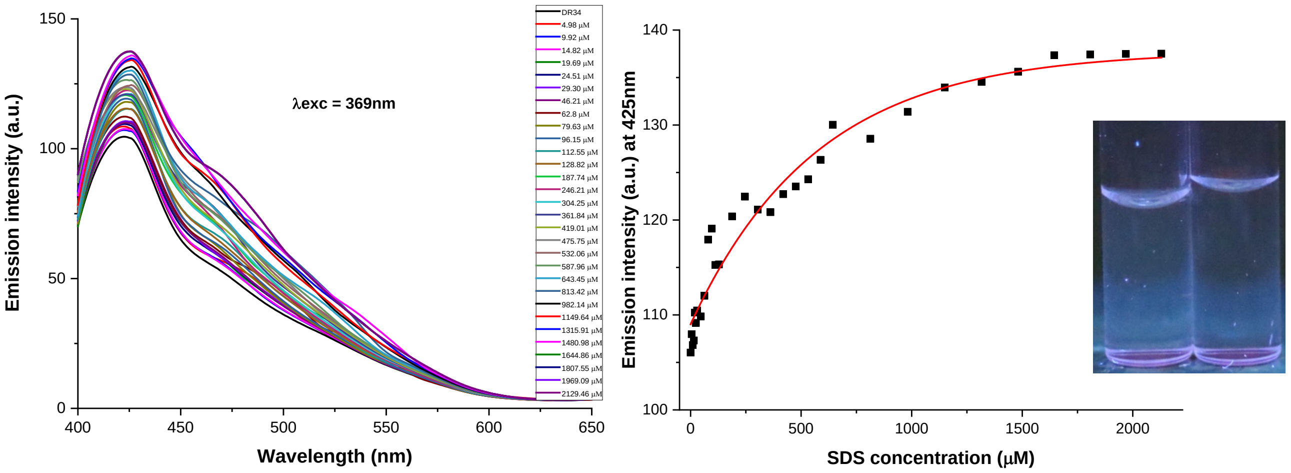

Figure S72: Fluorescence titration curve (left) and titration plot (right) between 7 and SDS in water (10 μM 7, 0 to 2.1 mM SDS). Insertion image: Solutions of 7 (10 μM, left), and after addition 2.2 mM SDS (right).

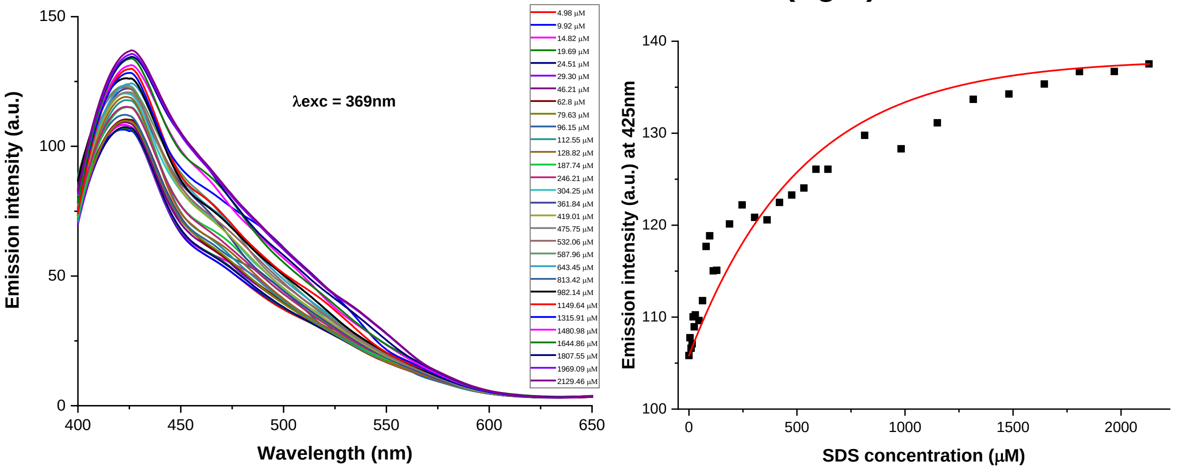

Figure S73: Blank fluorescence titration curve (left) and titration plot (right) between water and SDS in water (0 to 2.1 mM SDS) showing that the variation of the blue emission is due to the SDS addition.

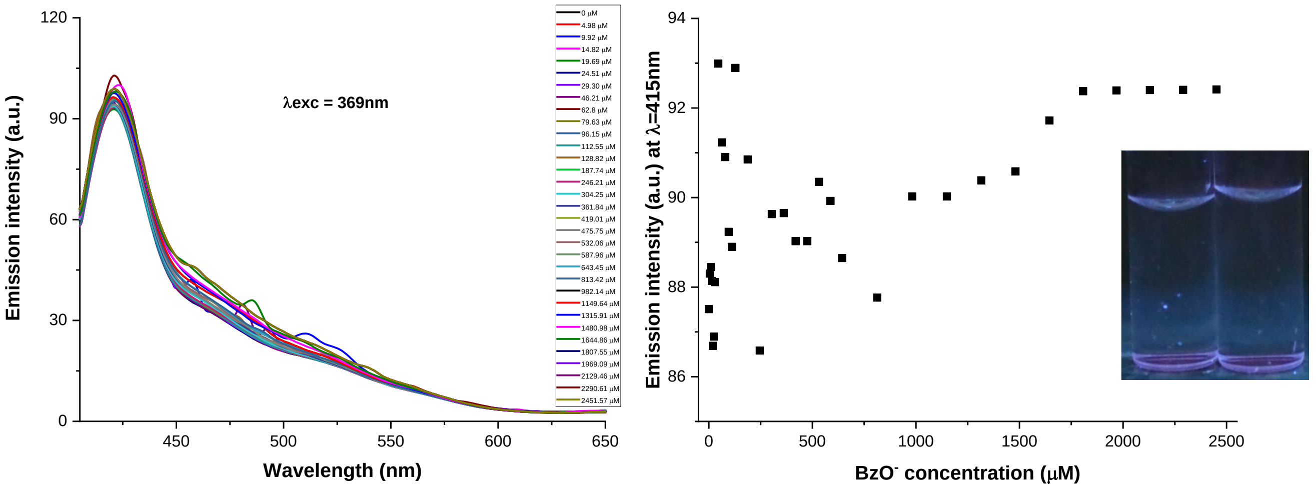

Figure S74: Fluorescence titration curve (left) and titration plot (right) between 7 and tetrabutylammonium benzoate in water (10 μM 7, 0 to 2.5 mM BzO^-). Insertion image: Solutions of 7 (10 μM, left), and after addition 2.5 mM BzO^- (right).

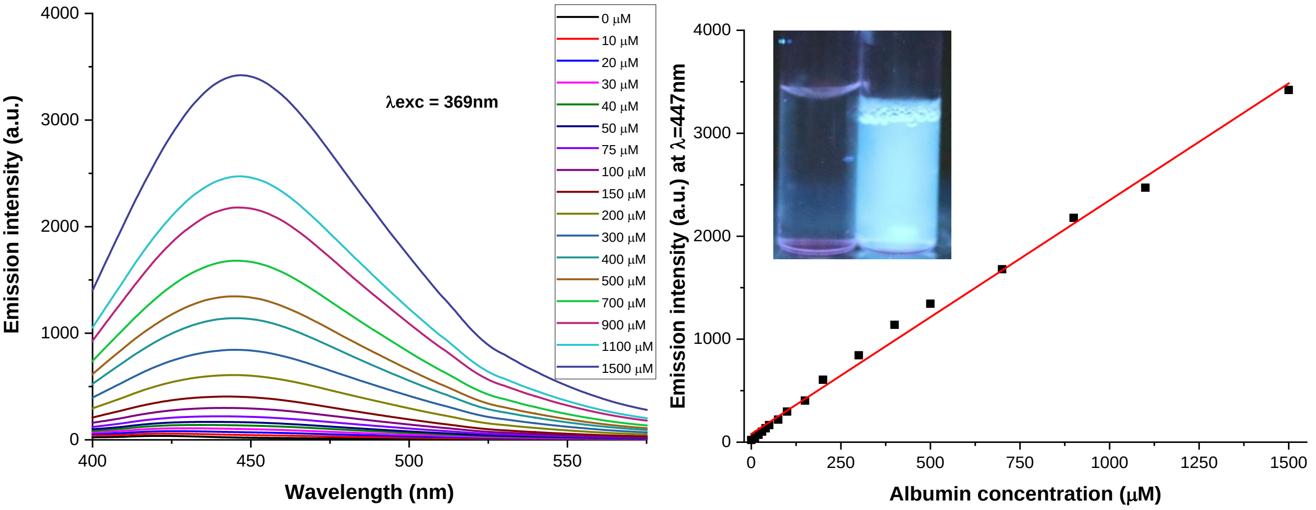

Figure S75: Fluorescence titration curve (left) and titration plot (right) between 7 and Albumin (BSA) in water (10 μM 7, 0 to 1.5 mM BSA). Insertion image: Solutions of 7 (10 μM, left), and after addition 1.5 mM BSA (right).

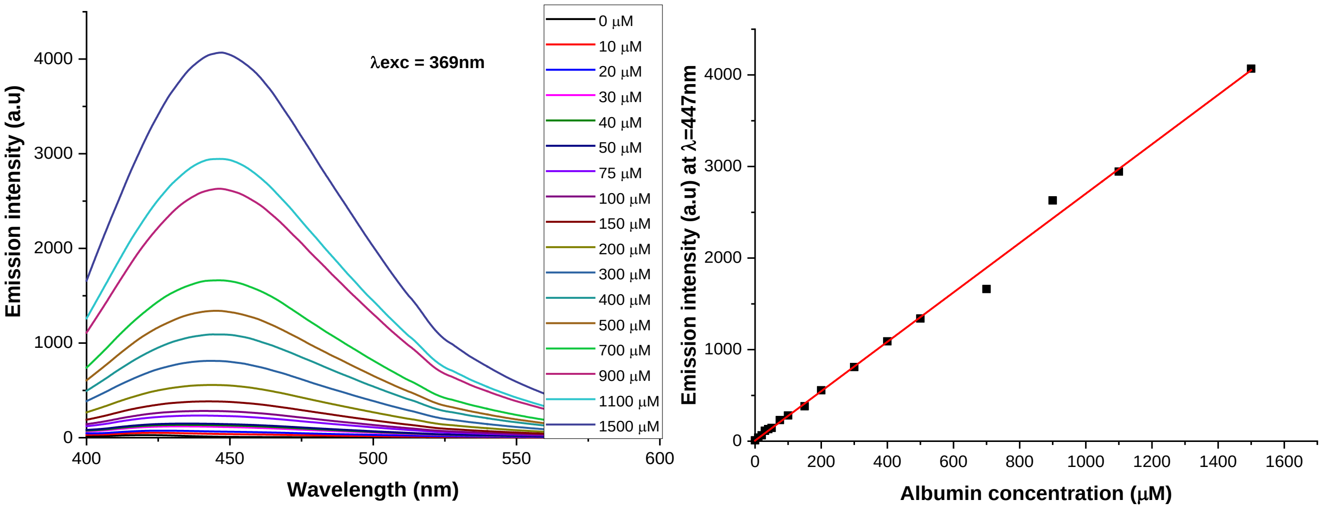

Figure S76: Blank fluorescence titration curve (left) and titration plot (right) between water and BSA in water (0 to 1.5 mM BSA) showing that the variation of the blue emission is due to the BSA addition. The quenching effect by the presence of 7 was observed to be very low.

Titration of 7 and carbazole by exciting at the wavelength used for the titrations of 7 (369nm).

The compound was dissolved in water at a concentration of 10 µM and subjected to increasing concentrations (0 to 2.56 mM) of carbazole. Excitation wavelength was 369 nm. The emission of carbazole at those same concentrations and excitation wavelength was also measured, to use as a reference. Slits, which due to the compound's low fluorescence emission, remained open at a máximum (20-20) in the other titrations, but they have to be be closed in this case at 5-2.5 due to the intense fluorescence of carbazole. **Figures S77** and **S78** show only small oscillations in the measurements. Therefore it can be concluded that the emission of compound **7** was not affected by the presence of carbazole.

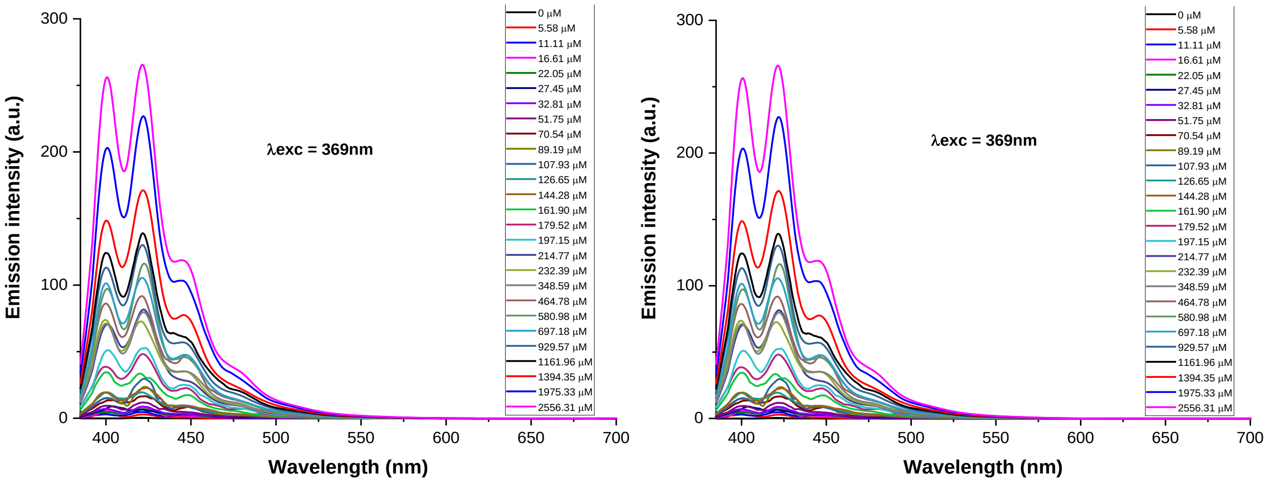

Figure S77. Fluorescence titration curve between 7 and carbazole (left) and emission spectra of increasing carbazole concentrations (right) in water (10 µM 7, 0 to 2.56 mM carbazole) λexc=369nm.

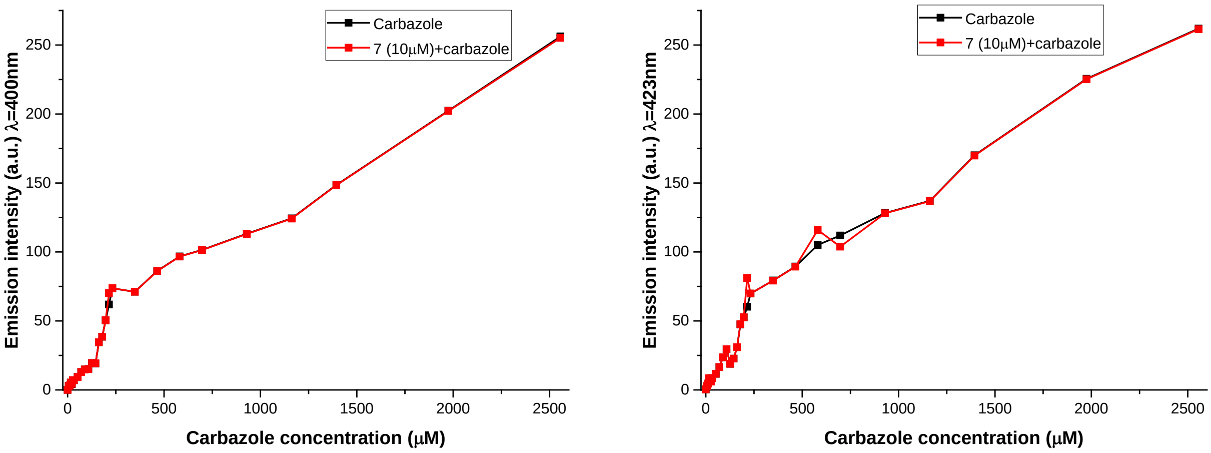

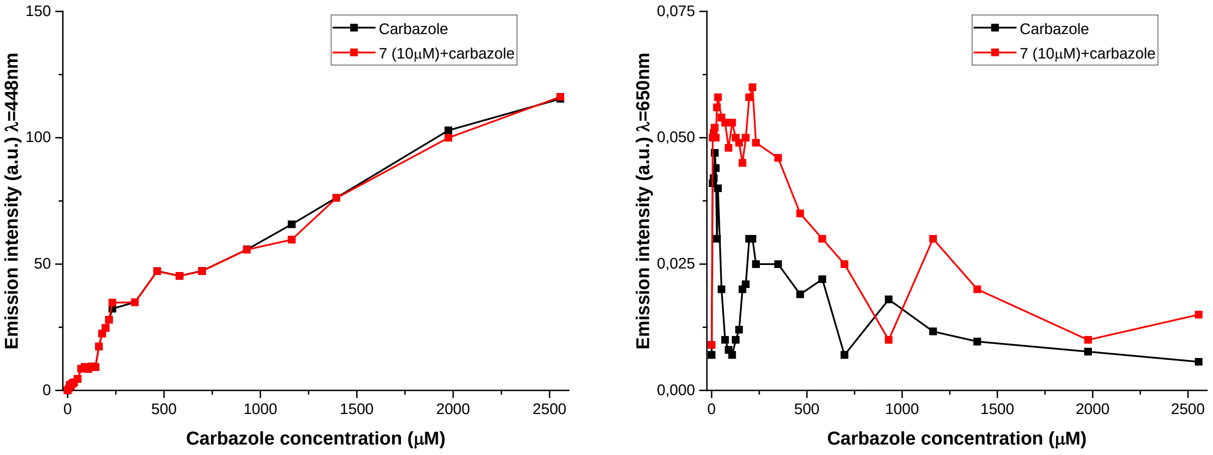

Figure S78. Representation of emission versus carbazole concentration at λ = 400 nm (upper left), 423 nm (upper right) 448 nm (down left) and 650 nm (down right) representing simultaneously the titration between 7 and carbazole and the measurements of only carbazole at the same concentrations.

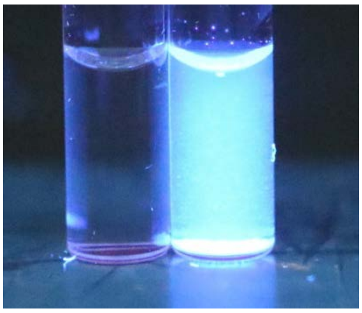

Figure S79. Fluorescence of 7 (10 μM) before (left) and after (right) the adition of 2.56 mM carbazole.

Titration between 7 and tryptophan by exciting at the wavelength used for the titrations of 7 (369nm).

The compound was dissolved in water at a concentration of 10 μM and subjected to increasing concentrations (0 to 2.56 mM) of tryptophan. Excitation wavelength was 369 nm. The emission of tryptophan at those same concentrations and excitation wavelength was also measured, to be used as a reference. **Figure S80** shows only random oscillations in the measurements. Trptophan showed no fluorescence emission when excited at that wavelength. Therefore it can be concluded that the emission of compound **7** was not affected by the presence of tryptophan.

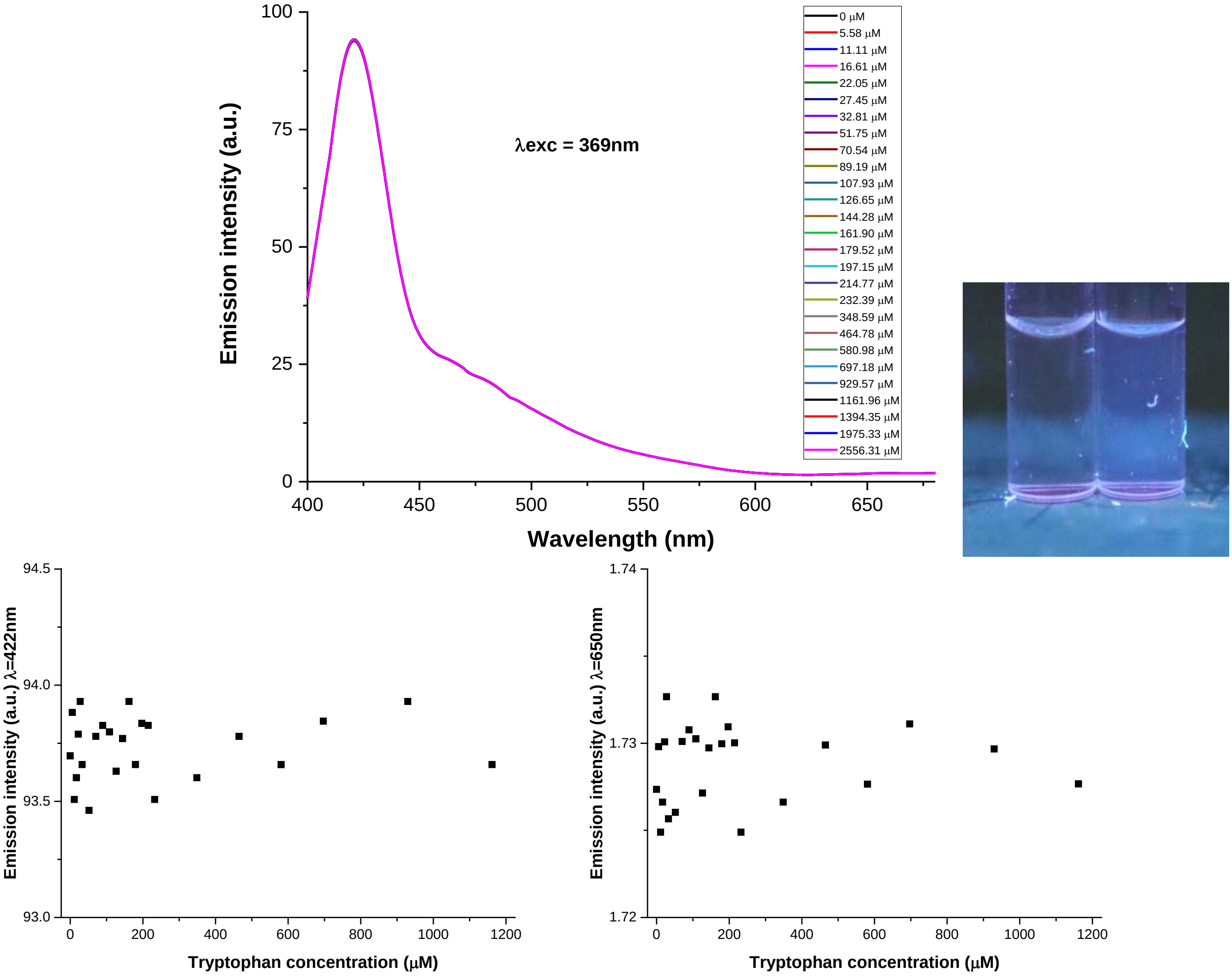

Figure S80. Fluorescence titration curve between 7 and tryptophan (up) and representation of emission versus tryptophan concentration at λ = 422 (down left) and 650 nm (down right) in water (10 µM 7, 0 to 2.56 mM tryptophan) λ exc = 369 nm. Inset: Fluorescence of 7 (10µM) before (left) and after (right) the adition of 2.56 mM tryptophan.

Titration of 7 and dinitrotoluene by exciting to the wavelength used for the titrations of 7 (369nm).

The compound **7** was dissolved in water at a concentration of 10 µM and subjected to increasing concentrations (0 to 2.56 mM) of dinitrotoluene. Excitation wavelength was 369 nm. The emission of dinitrotoluene at those same concentrations and excitation wavelength was also measured, to be used as a reference. **Figure S81** shows only random oscillations in the measurements. Therefore it can be concluded that the emission of compound **7** was not affected by the presence of dinitrotoluene.

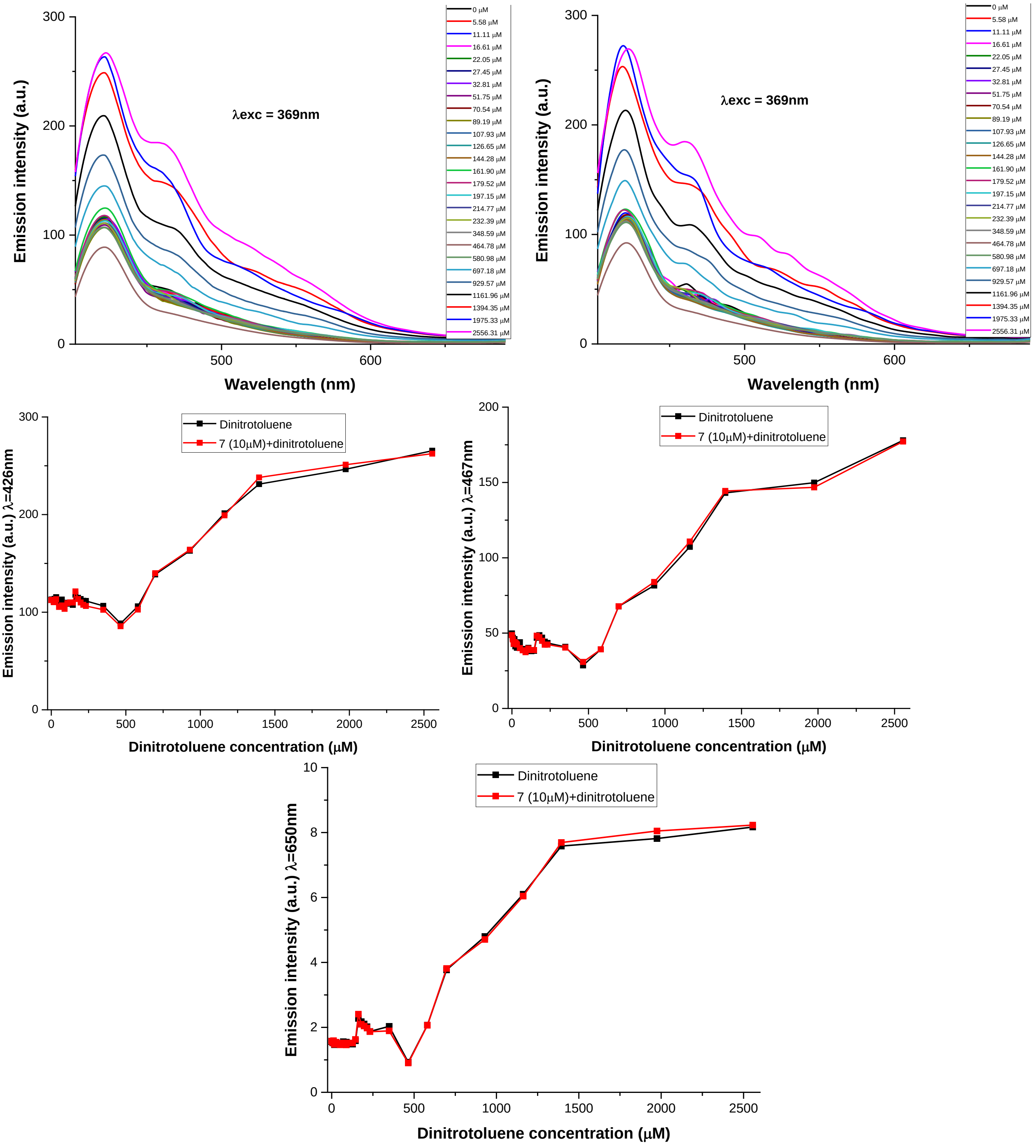

Figure S81. Fluorescence titration curves between 7 and dinitrotoluene (up left), emission spectra of increasing dinitrotoluene concentrations (up right) in water (10 μM 7, 0 to 2.56 mM dinitrotoluene) λexc = 369 nm and representation of emission versus dinitrotoluene concentration at λ = 426nm (middle left), 467 nm (middle right) and 650 nm (down) representing simultaneously the titration between 7 and dinitrotoluene and the measurements of only dinitrotoluene.

Titration between 7 and dinitrotoluene by exciting to the wavelength of the dinitrotoluene (295 nm).

The compound was dissolved in water at a concentration of 10 µM and subjected to increasing concentrations (0 to 2.56 mM) of dinitrotoluene. Excitation wavelength was 295 nm. The emission of dinitrotoluene at those same concentrations and excitation wavelength was also measured, to be used as a reference. **Figure S82** shows only random oscillations in the measurements. Therefore it can be concluded that the emission of dinitrotoluene was not affected by the presence of compound **7**.

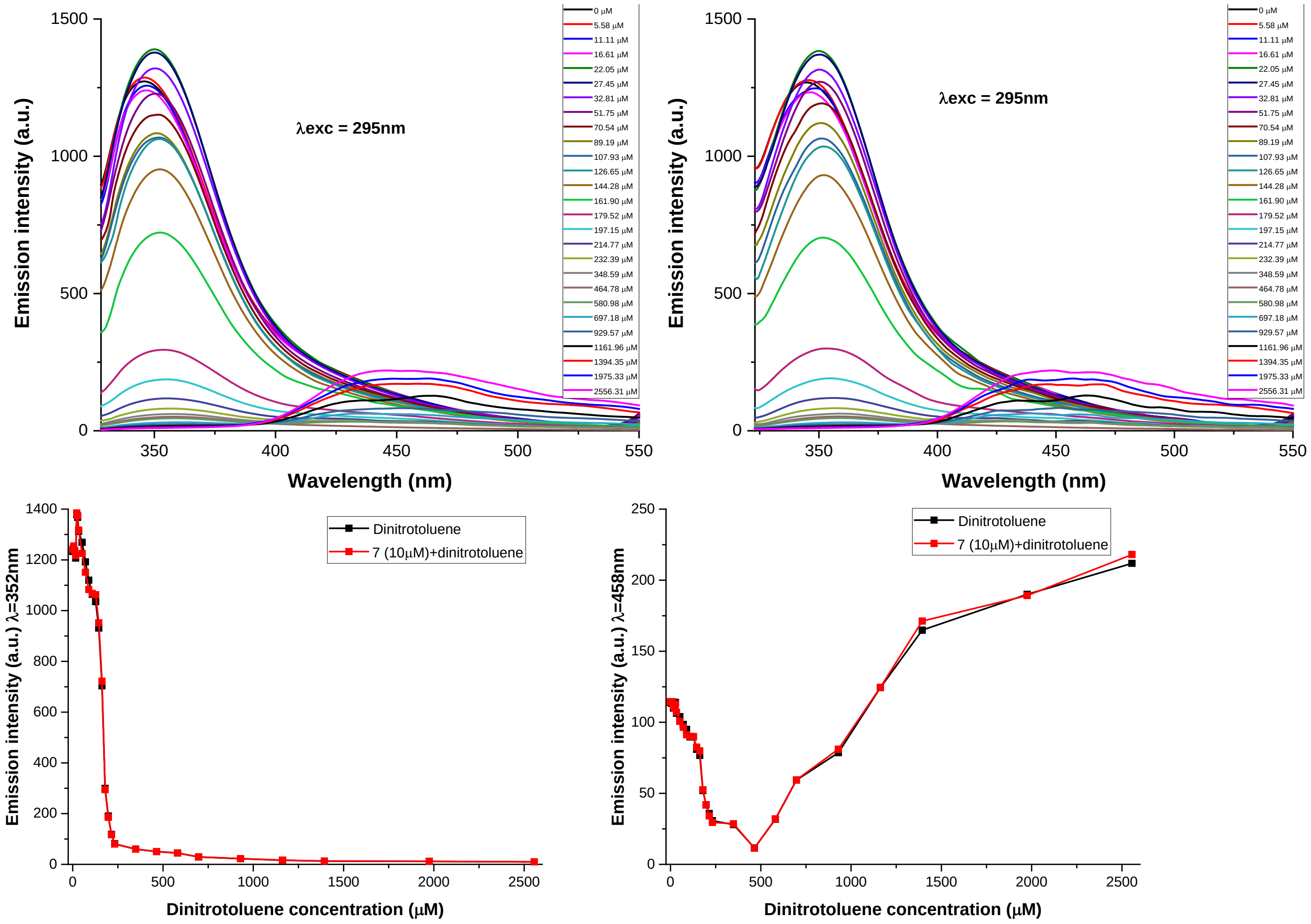

Figure S82. Fluorescence titration curves between 7 and dinitrotoluene (up left), emission spectra of increasing dinitrotoluene concentrations (up right) in water (10 µM 7, 0 to 2.56 mM dinitrotoluene) λexc = 295 nm and representation of emission versus dinitrotoluene concentration at λ = 352 nm (down left) and 458 nm (down right) representing simultaneously the titration between 7 and dinitrotoluene and the measurements of only dinitrotoluene.

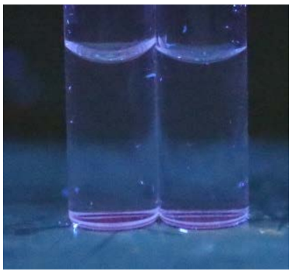

Figure S83. Fluorescence of 7 (10 µM) before (left) and after (right) the adition of 2.56 mM dinitrotoluene.

Titration between 7 and nitrobenzene by exciting to the wavelength used for the titrations of 7 (369nm).

The compound was dissolved in water at a concentration of 10 µM and subjected to increasing concentrations (0 to 2.56 mM) of nitrobenzene. Excitation wavelength was 369 nm. The emission of nitrobenzene at those same concentrations and excitation wavelength was also measured, to be used as a reference. **Figure S84** shows only random oscillations in the measurements. Therefore it can be concluded that the emission of compound **7** was not affected by the presence of nitrobenzene.

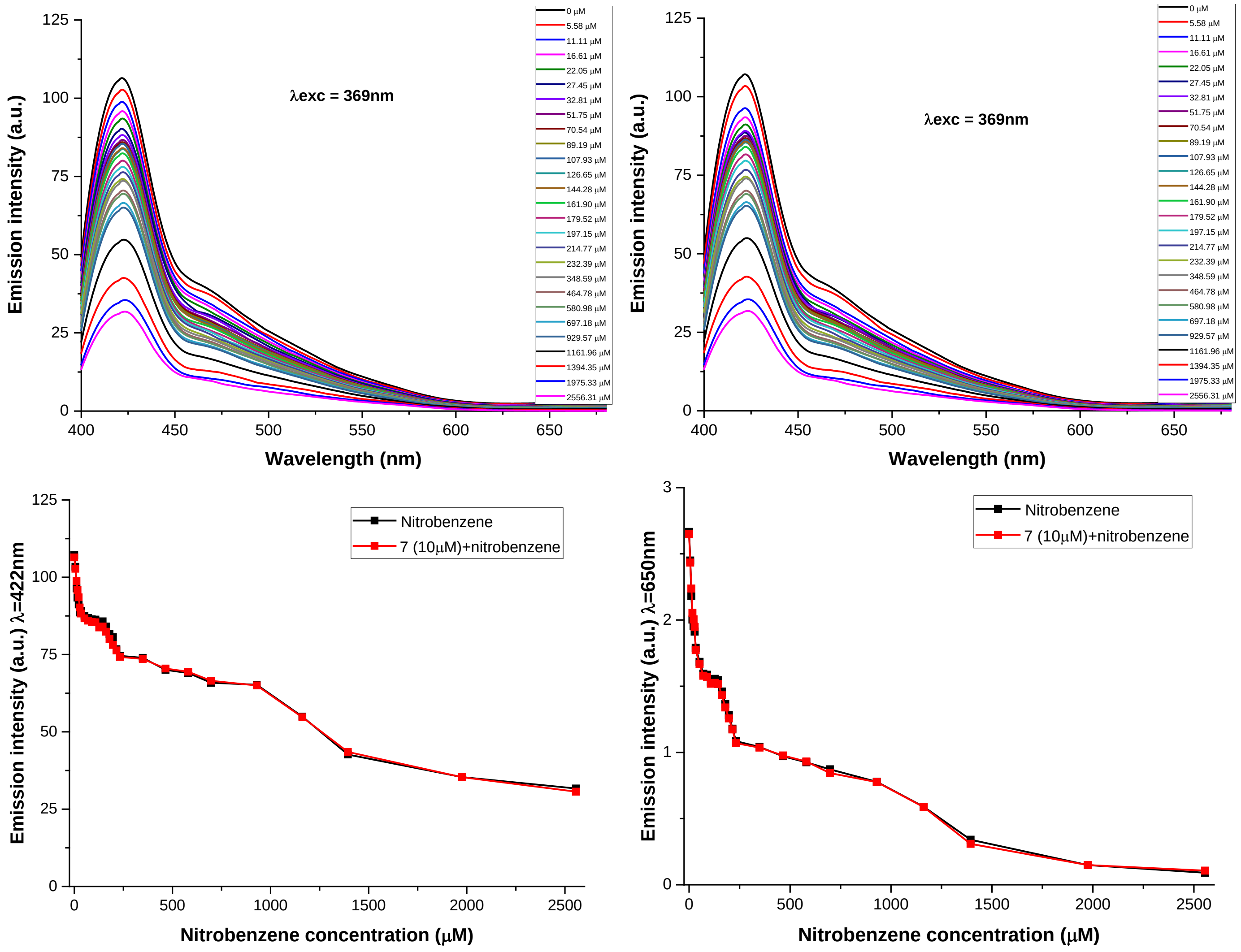

Figure S84. Fluorescence titration curves between 7 and nitrobenzene (up left), emission spectra of increasing nitrobenzene concentrations (up right) in water (10 µM 7, 0 to 2.56 mM nitrobenzene) λexc = 369nm and representation of emission versus nitrobenzene concentration at λ = 422 nm (down left) and 650 nm (down right) representing simultaneously the titration between 7 and nitrobenzene and the measurements of only nitrobenzene.

Titration of 7 and nitrobenzene by exciting to the wavelength of the nitrobenzene (280 nm).

The compound **7** was dissolved in water at a concentration of 10µM and subjected to increasing concentrations (0 to 2.56 mM) of nitrobenzene. Excitation wavelength was 280 nm. The emission of nitrobenzene at those same concentrations and excitation wavelength was also

measured, to be used as a reference. **Figure S85** shows only random oscillations in the measurements. Therefore it can be concluded that the emission of nitrobenzene was not affected by the presence of **7**.

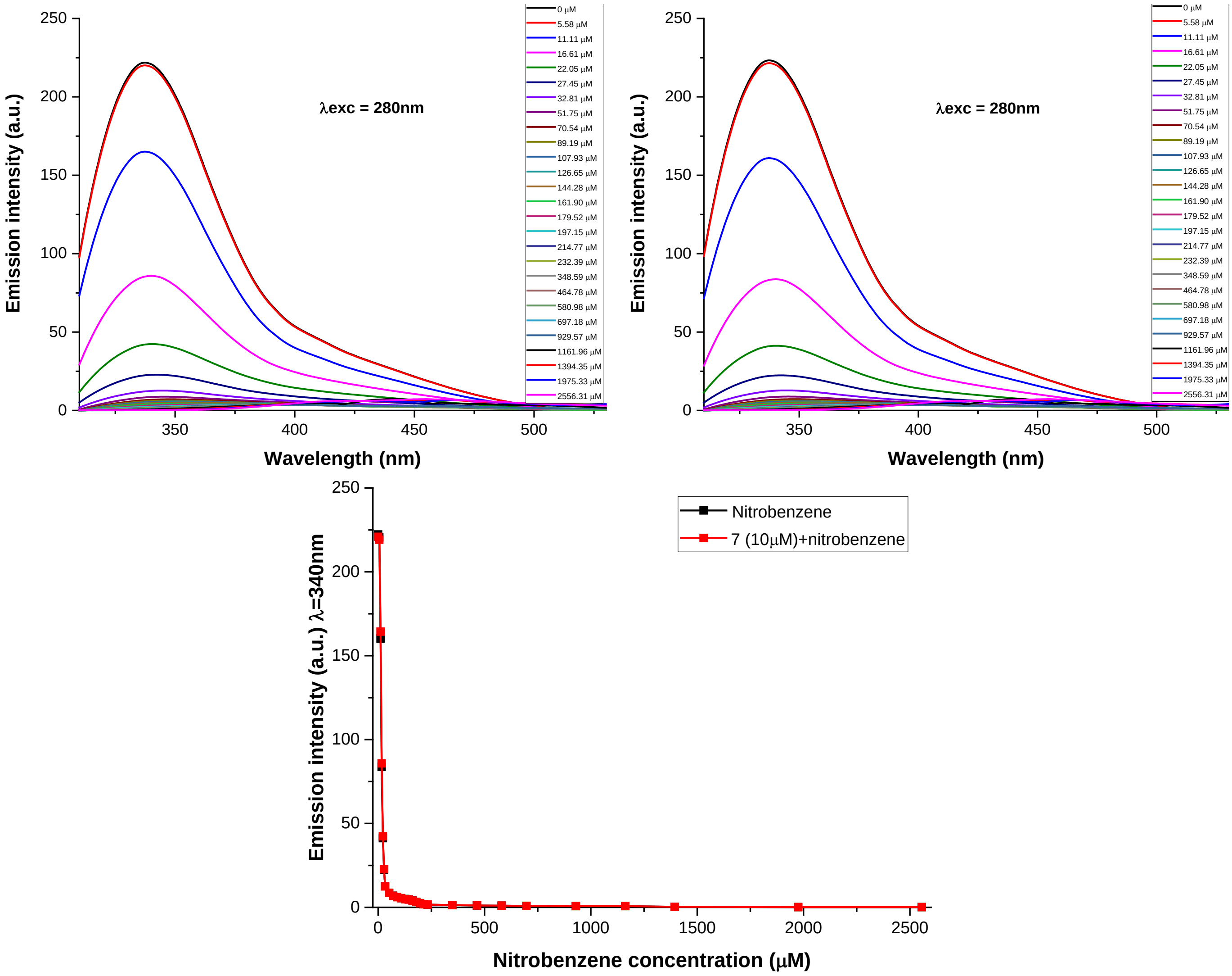

Figure S85. Fluorescence titration curve between 7 and nitrobenzene (up left), emission spectra of increasing nitrobenzene concentrations (up right) in water (10 μM 7, 0 to 2.56 mM nitrobenzene) λexc = 280 nm and representation of emission versus nitrobenzene concentration at λ = 340 nm (down left) representing simultaneously the titration between 7 and nitrobenzene and the measurements of only nitrobenzene.

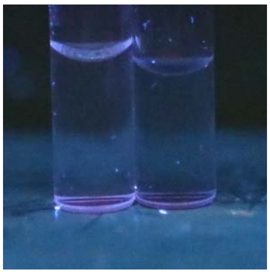

Figure S86. Fluorescence of 7 (10μM) before (left) and after (right) the adition of 2.56 mM nitrobenzene.

Interference tests in the presence of ions usually found in seawater (see for example: https://www.britannica.com/science/seawater):

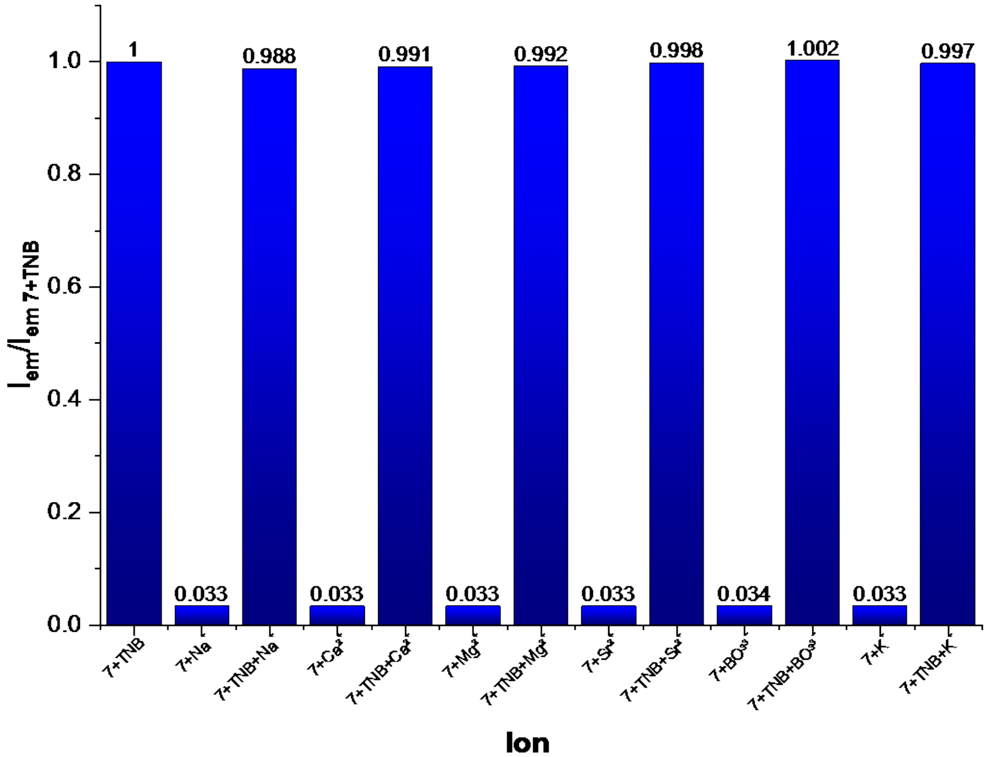

Figure S87: Interference tests between 7 and common ions found in sea water before and after the addition of TNB. Conditions: 7 in water (2.7 mM), Na^+ (from Na_2CO_3), 23.6 mM, Ca^{2+} (from $CaCl_2$), 22.5 mM, Mg^{2+} (from $MgSO_4$) 20.8 mM, Sr^{2+} (from $SrCO_3$), 16.9 mM, K^+ (from KCl), 33.5 mM, BO_3^{3-} (from H_3BO_3), 40.4 mM, TNB, 7.8 mM, after addition of every salt to every solution.

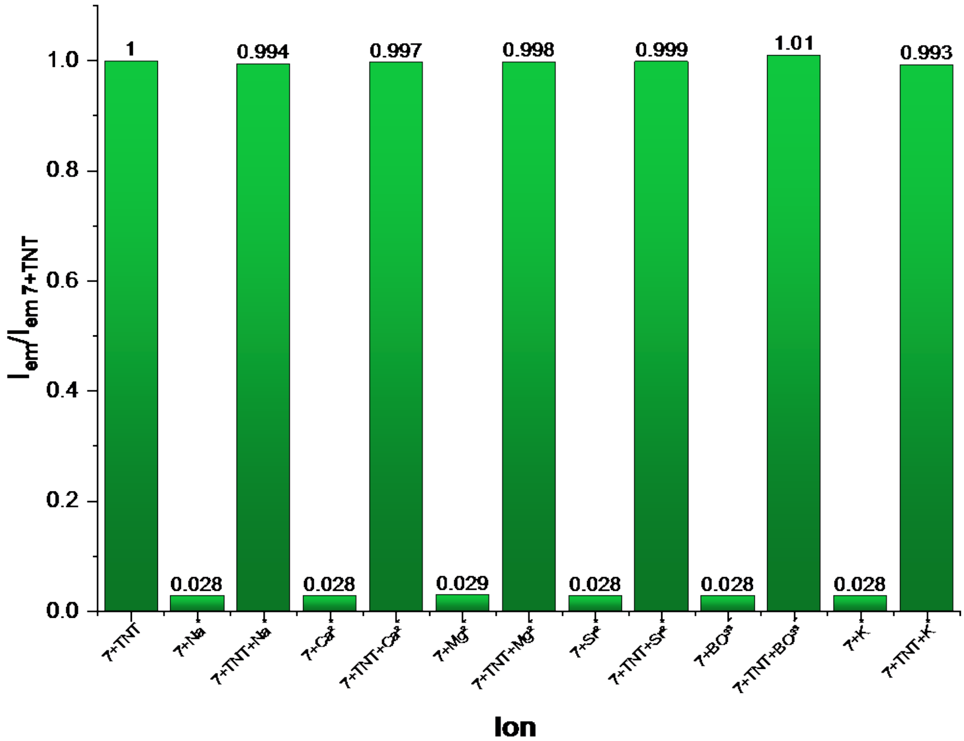

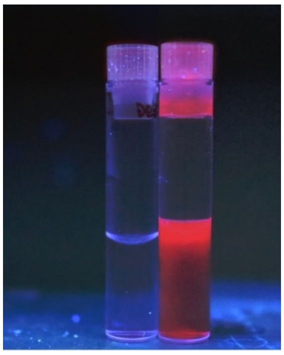

Figure S88: Interference tests between 7 and common ions found in sea water before and after the addition of TNT. Conditions: 7 in water (2.7 mM), Na^+ (from Na_2CO_3), 23.6 mM, Ca^{2+} (from $CaCl_2$), 22.5 mM, Mg^{2+} (from $MgSO_4$) 20.8 mM, Sr^{2+} (from $SrCO_3$), 16.9 mM, K^+

(from KCl), 33.5 mM, BO_3^{3-} (from H_3BO_3), 40.4 mM, TNT, 7.3 mM after addition of every salt to every solution. Inset: Solutions of 7 in water (2.7 mM) and KCl, 33.5 mM, before and after the addition of TNT

ITC measurements. The binding isotherm was fitted by a dimer dissociation model (NanoAnalyze Software, TA Instruments). The obtained thermodynamic parameters, collected in Table S1, agree with the aggregation process.

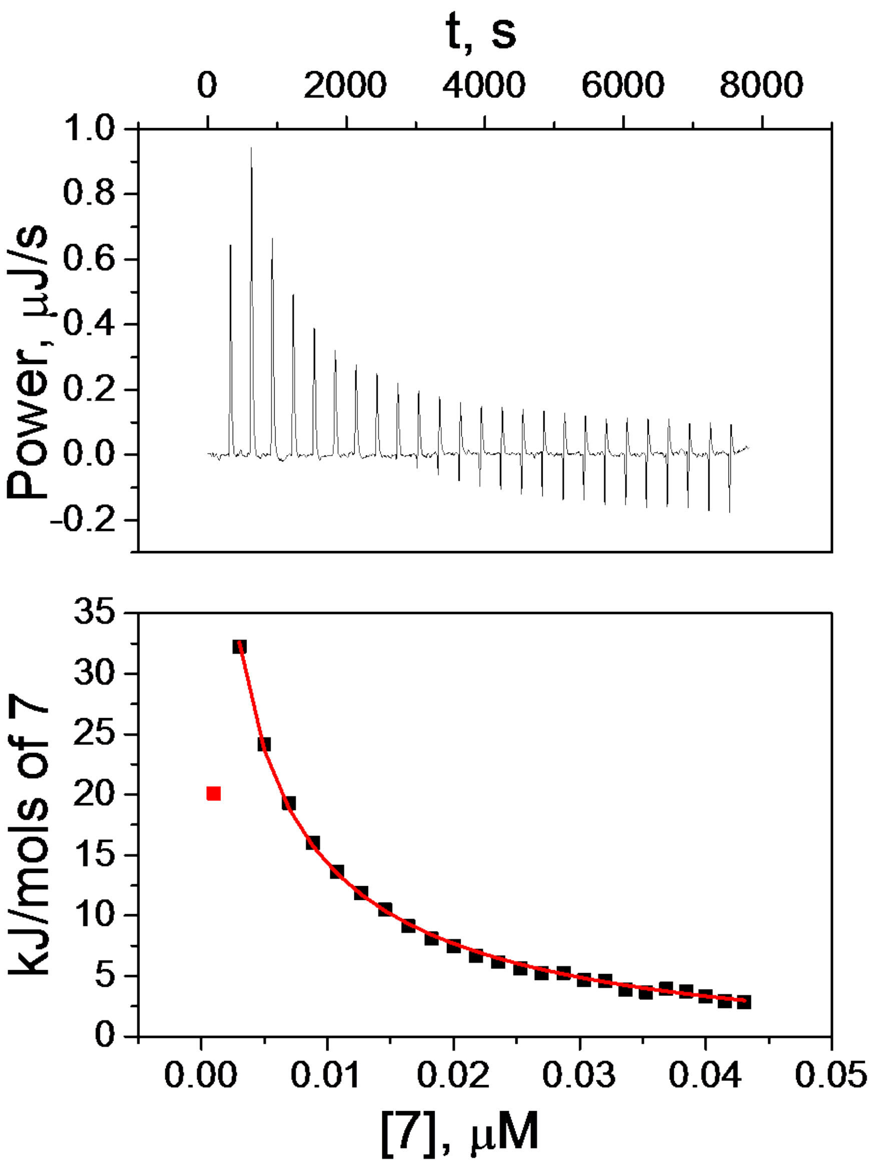

Figure S89: ITC profile of 7 dissociation. $C_{[7]}$ = 1 mM, T = 25°C.

Table S1. ITC Thermodynamic parameters for the aggregation of 7.

K_{agg}, M^{-1}	ΔH, $kJmol^{-1}$	ΔS, $Jmol^{-1}$ K^{-1}
$(1.39 \pm 0.4)\times10^{4}$	-66 ± 2	-142 ± 9

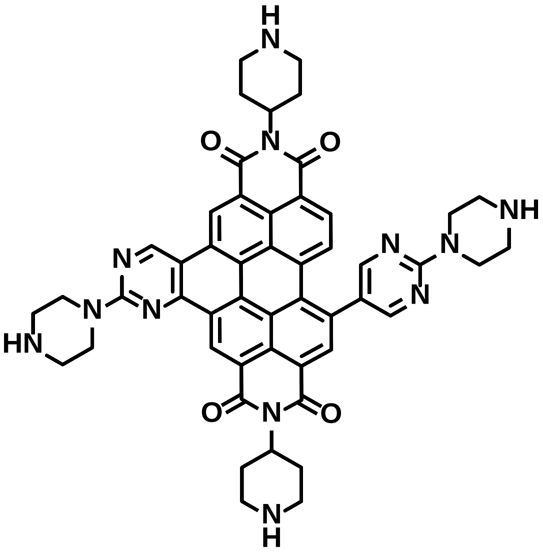

8

***N*,*N'*-Bis-(piperidin-4-yl)-12-((piperazin-4-yl)-1,3-pyrimidin)-[6,7-e]-(2-(1,4-piperazin-1-yl)-1,3-pyrimidin)perylene-3,4:9,10-tetracarboxylic diimide 8**. MP (ºC): > 350ºC. R_f (CH_2Cl_2:MeOH, 50:4): 0.01. FT-IR (KBr, cm^{-1}): 3452 (N-H, amine), 2961 (C-H, aromatic), 2919 (C-H, aliphatic), 2850 (C-H, aliphatic), 1682 (C=O, imide), 1522 (C=C), 1442 (C-N), 1365 (C-N), 1323, 1205 (C-N), 1142. ^1H NMR (300 MHz, DTFA) δ: 10.85 – 10.66 (m, 2H, Ar-H), 10.59 (s, 1H, Ar-H), 10.46 – 10.37 (m, 1H, Ar-H), 10.02 (s, 1H, Ar-H), 8.98 (s, 1H, Ar-H), 8.80 (d, J = 8.6 Hz, 1H, Ar-H), 8.70 (d, J = 8.7 Hz, 1H, Ar-H), 7.51 – 7.18 (m, 2H, NH), 7.13 – 6.77 (m, 2H, NH), 5.69 – 5.53 (m, 2H, N-CH), 4.77 – 4.67 (m, 4H, CH_2), 3.88 – 3.69 (m, 12H, CH_2), 3.45 – 3.17 (m, 8H, CH_2), 2.29 – 2.09 (m, 6H, CH_2), 1.36 – 1.17 (m, 2H, CH_2). HR-MS (MALDI+, HCCA): *m/z* calcd. for $C_{50}H_{46}N_{12}O_4$ ($[M+H]^+$): 879.3838; found: 879.3848. HR-MS (MALDI+, DCTB) 2[8]: m/z calcd. for 2($C_{50}H_{46}N_{12}O_4$) ($[2M+H]^+$): 1757; found: 1757. HR-MS (MALDI+, DCTB) [8]+CB[7]: m/z calcd. for ($C_{50}H_{47}N_{12}O_4$+$C_{42}H_{42}N_{28}O_{14}$) ($[M+CB[7]+H]^+$): 2041.7273; found: 2041.7214. MS (MALDI+, DCTB) [8]+2CB[7]: m/z calcd. for ($C_{50}H_{47}N_{12}O_4$+2$C_{42}H_{42}N_{28}O_{14}$) ($[M+2CB[7]+H]^+$): 3205; found: 3209. UV-VIS (H_2O) λ_{max} / nm (ε / $M^{-1}\cdot cm^{-1}$): 458 (329).

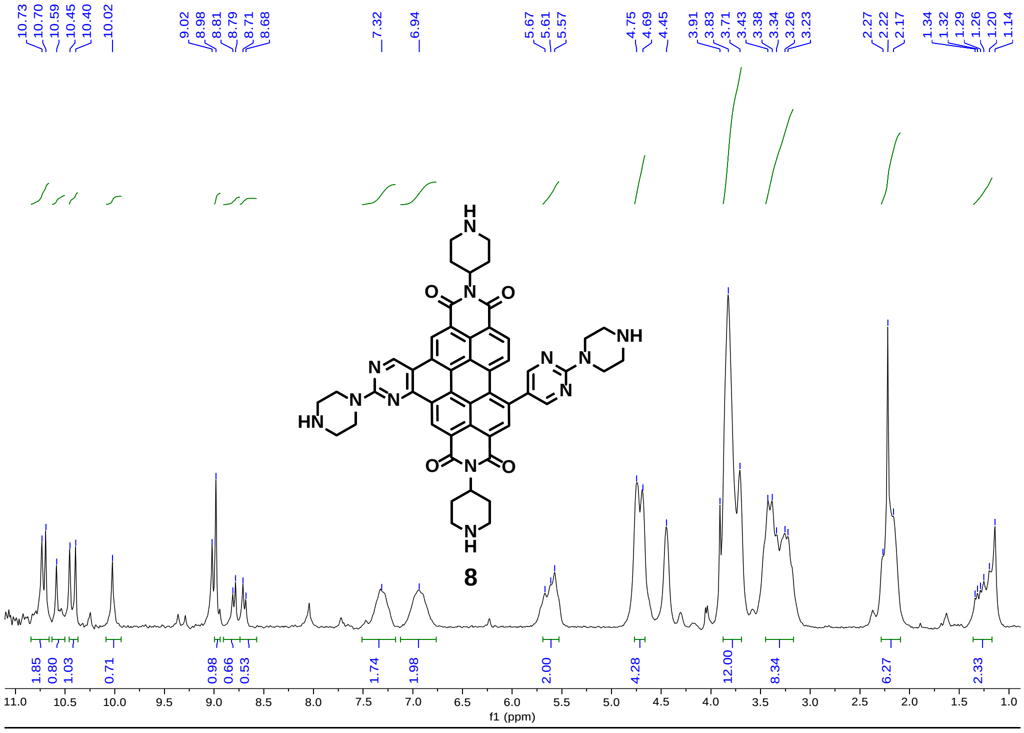

Figure S90: ^{1}H NMR (300 MHz, DTFA) of 8.

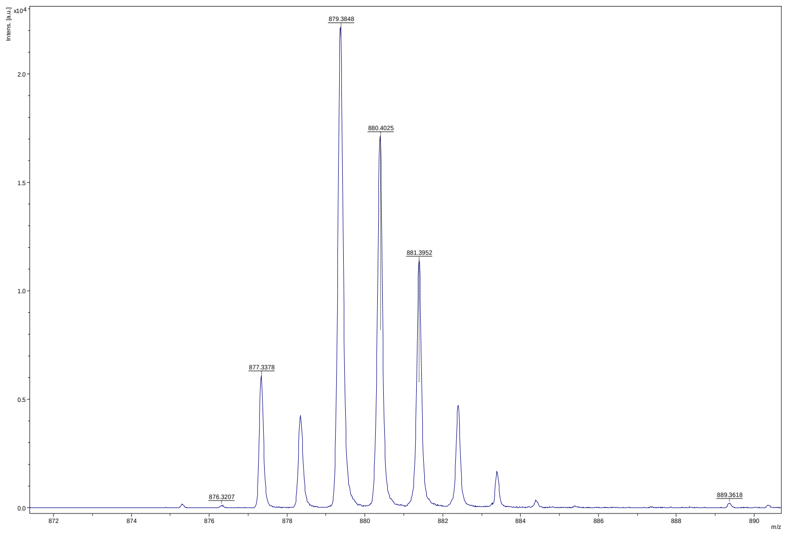

Figure S91: HRMS (MALDI+, HCCA) of 8.

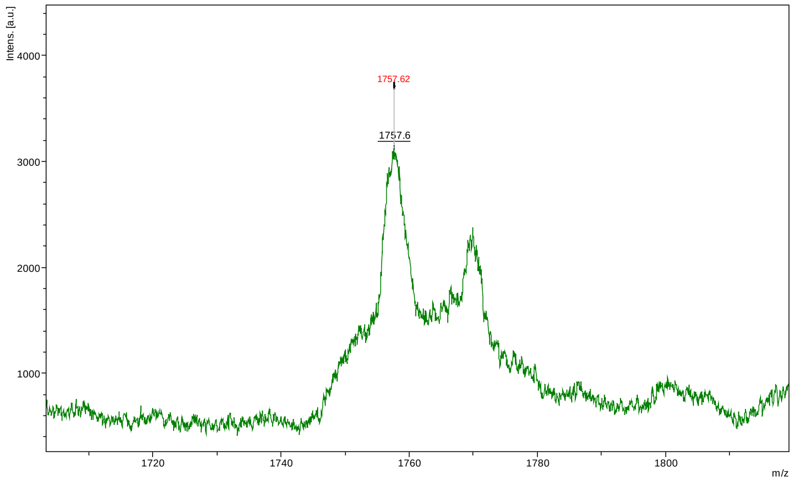

Figure S92: HRMS (MALDI+, DCTB) 2[8] stacked.

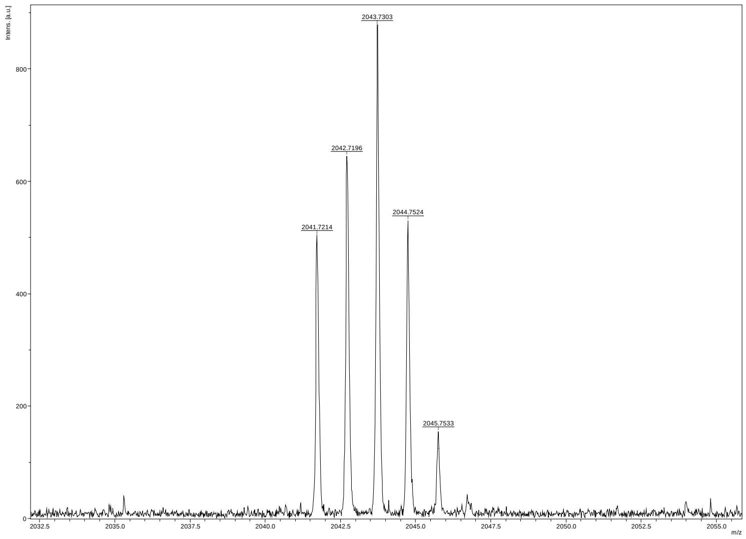

Figure S93: HRMS (MALDI+, DCTB) [8] + 1CB[7].

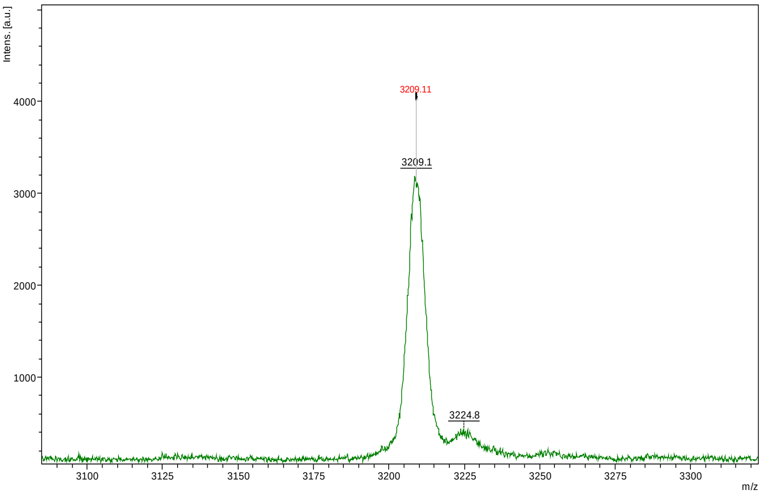

Figure S94: HRMS (MALDI+, DCTB) [8] + 2CB[7].

Solvatochromism tests:

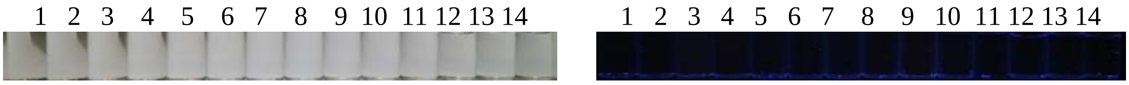

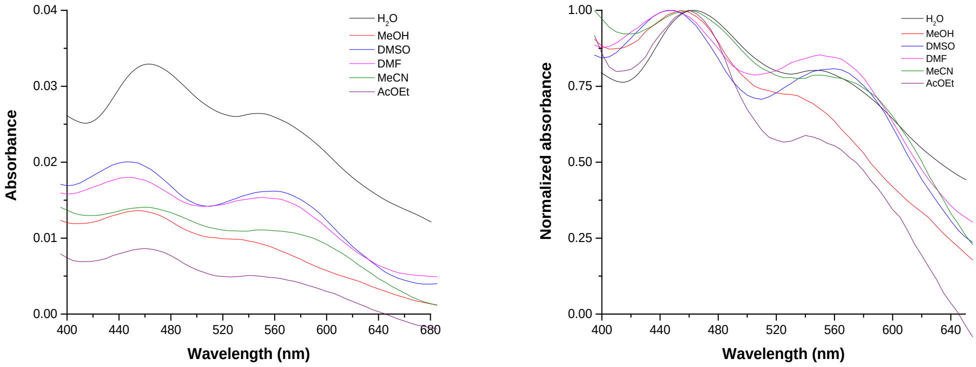

Figure S95: Solvatochromism test: Up: Photos under white and 366 nm lights. Down: Absorption (left) and emission spectra (right) under excitation wavelength of 458 nm. The employed solvents were: 1: H_2O, 2: MeOH (methanol), 3: DMSO (dimethylsulfoxide), 4: DMF (*N*,*N'*-dimethylformamide), 5: MeCN (acetonitrile), 6: Acetone, 7: EtOAc (ethyl acetate), 8: THF (tetrahydrofuran), 9: $CHCl_3$, 10: CH_2Cl_2 (dichloromethane), 11: Toluene, 12: Et_2O (diethyl ether), 13: *n*-Hx (hexane), 14: *c*-Hx (cyclohexane).

Kinetic effect study:

Compound was dissolved in water. Absorbance and emission (excitation wavelength 458 nm) were measured for 270 minutes with a rate of 1 spectrum by 3 minute. No significant changes in the spectra were appreciated after 50 minutes.

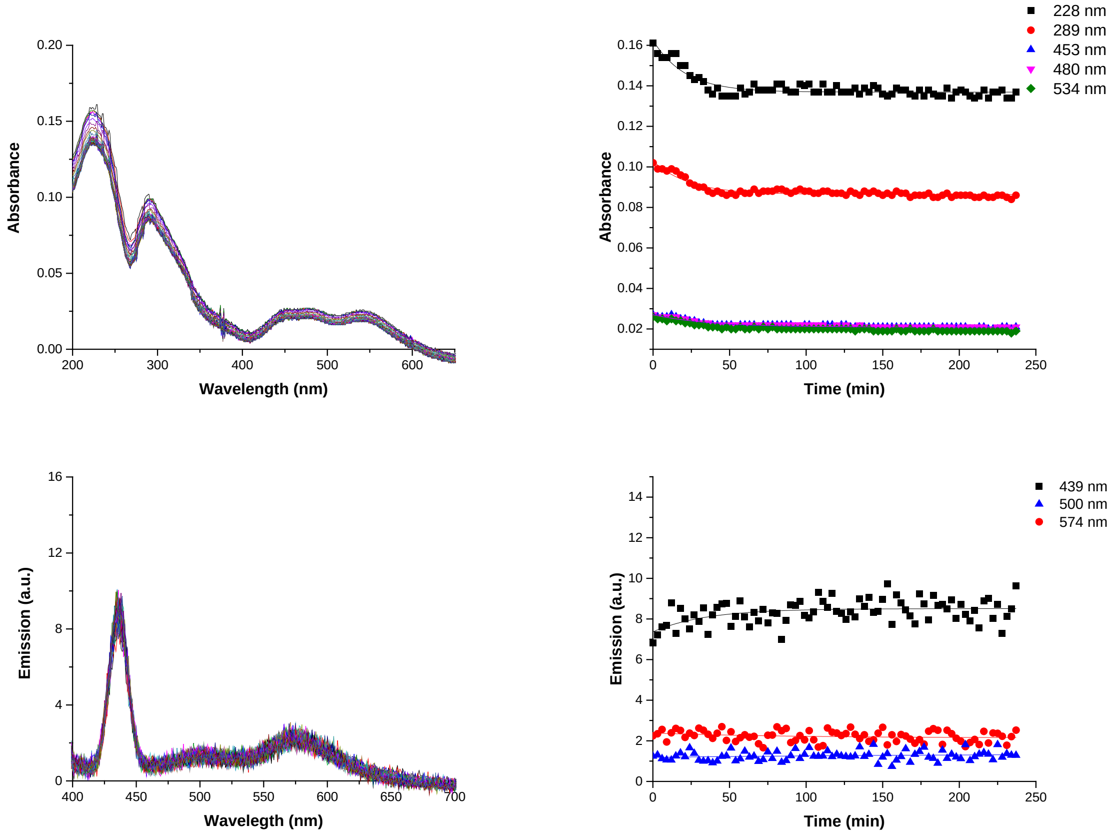

Figure S96: Up: Left: Absorbance spectra at different time points. Right: Representation of absorbance maxima along the time. Down: Left: Emission spectra at different times. Right: Representation of emission maxima along the time.

Morphology: AFM images of samples prepared in water in different concentrations. Solutions were prepared freshly or five days before.

**Solution**			
**Freshly prepared**		**Prepared after five days**	
**0.1 µg/mL**	**1 µg/mL**	**0.1 µg/mL**	**1 µg/mL**

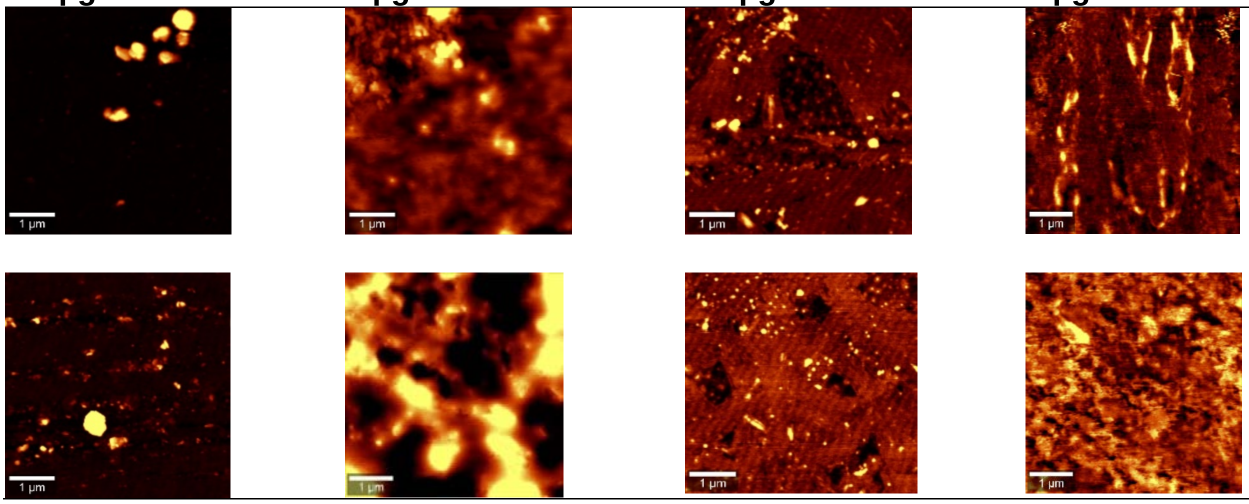

Figure S97: AFM images of samples of 8 in water.

DLS (Dynamic Light Scattering) study, samples prepared in water 0.1 μg/mL:
As prepared:

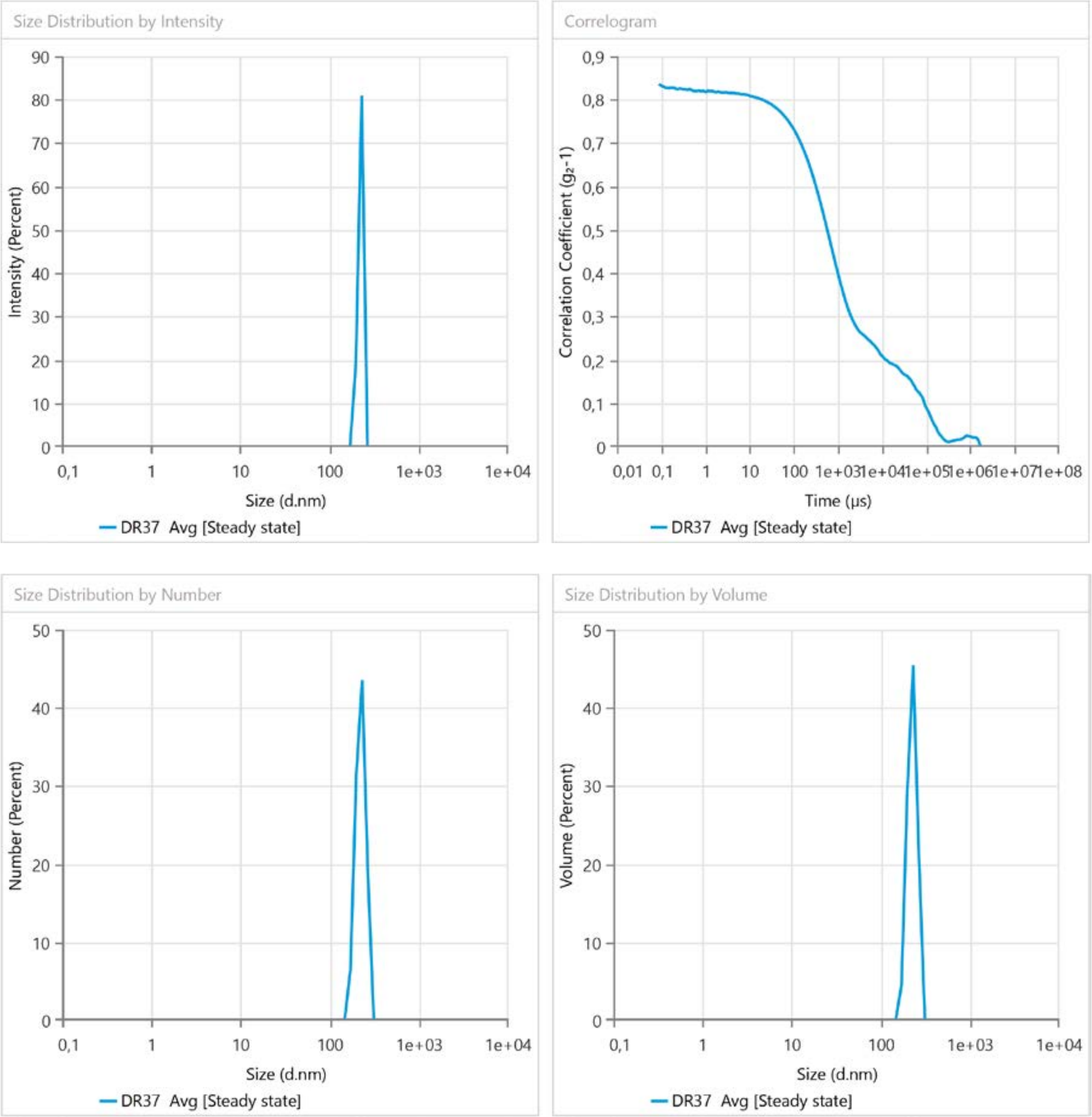

Parameter List

**Z-Average (nm)**	: 844
**Polydispersity Index (PI)**	: 0,6187
**Intercept**	: 0,8637
**Peak One Mean by Intensity (nm)**	: 223,6
**Peak Two Mean by Intensity (nm)**	:
**Peak Three Mean by Intensity (nm)**	:
**Peak One Mean by Number (nm)**	: 222,7
**Peak Two Mean by Number (nm)**	:
**Peak Three Mean by Number (nm)**	:
**Instrument Serial Number**	: MAL1259671
**Software Version**	: 2.0.1.1

Parameter List

**In Range (%)**	: 81,03
**Fit Error**	: 0,01796
**Run Retention (%)**	: 96
**Run Duration (s)**	: 1,68
**Number Of Runs**	: 30
**Derived Mean Count Rate (kcps)**	: 409,4
**Attenuator**	: 11
**Cuvette Position (mm)**	: 4,64
**Detector Angle (°)**	: 173
**Seek Optimum Attenuator Position**	: True

Figure S98: DLS results, sample of 8 prepared in water 0.1 μg/mL.

As prepared, three repetitions, superposed:

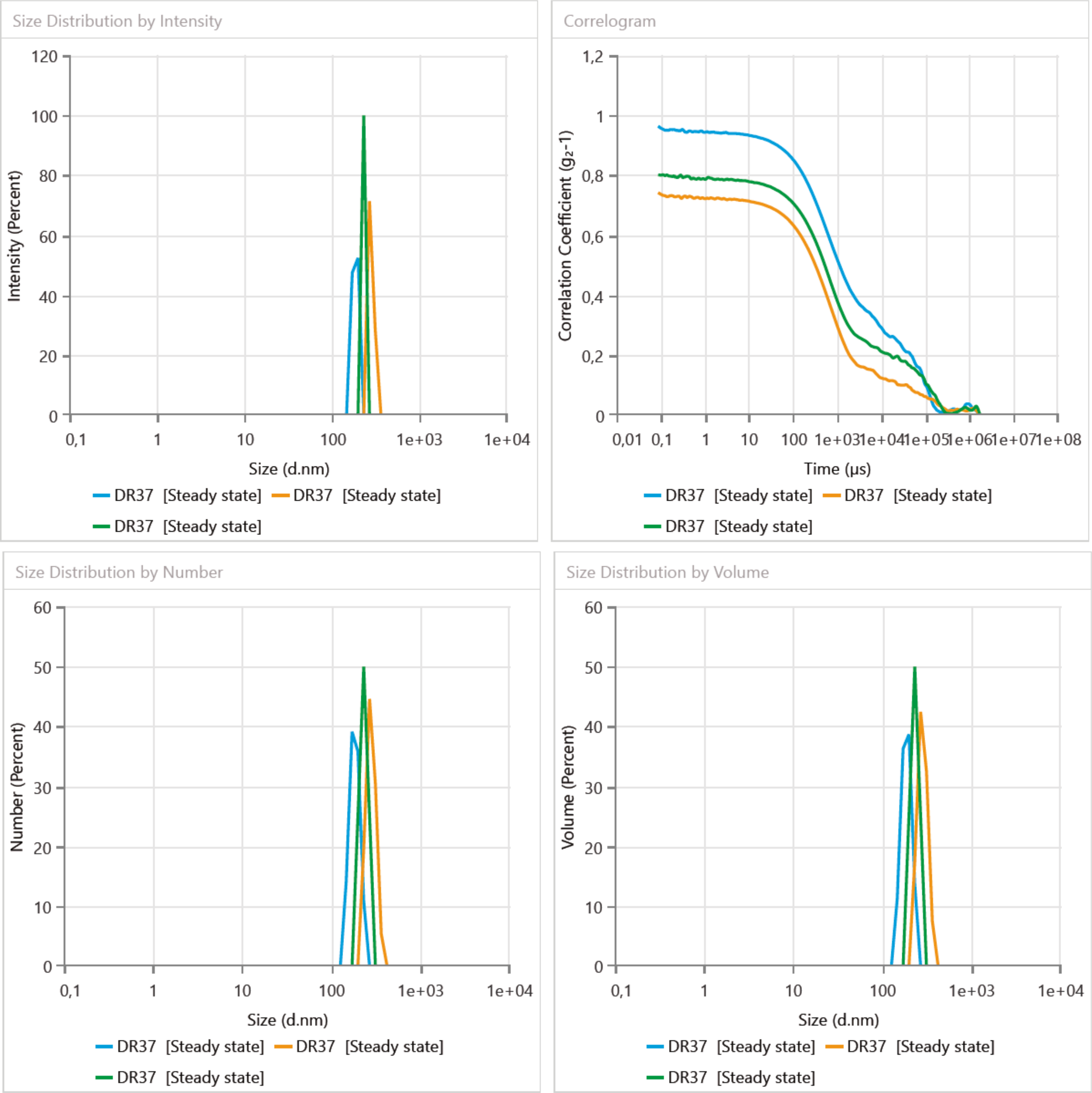

Parameter List

**Z-Average (nm)**	: 1106
**Polydispersity Index (PI)**	: 0,7977
**Intercept**	: 0,9356
**Peak One Mean by Intensity (nm)**	: 184,4
**Peak Two Mean by Intensity (nm)**	:
**Peak Three Mean by Intensity (nm)**	:
**Peak One Mean by Number (nm)**	: 183
**Peak Two Mean by Number (nm)**	:
**Peak Three Mean by Number (nm)**	:
**Instrument Serial Number**	: MAL1259671
**Software Version**	: 2.0.1.1

Parameter List

**In Range (%)**	: 84,26
**Fit Error**	: 0,02092
**Run Retention (%)**	: 100
**Run Duration (s)**	: 1,68
**Number Of Runs**	: 30
**Derived Mean Count Rate (kcps)**	: 402,7
**Attenuator**	: 11
**Cuvette Position (mm)**	: 4,64
**Detector Angle (°)**	: 173
**Seek Optimum Attenuator Position**	: True

Figure S99: DLS results, sample of 8 prepared in water 0.1 µg/mL.

Filtered, 0.22 µm: As prepared:

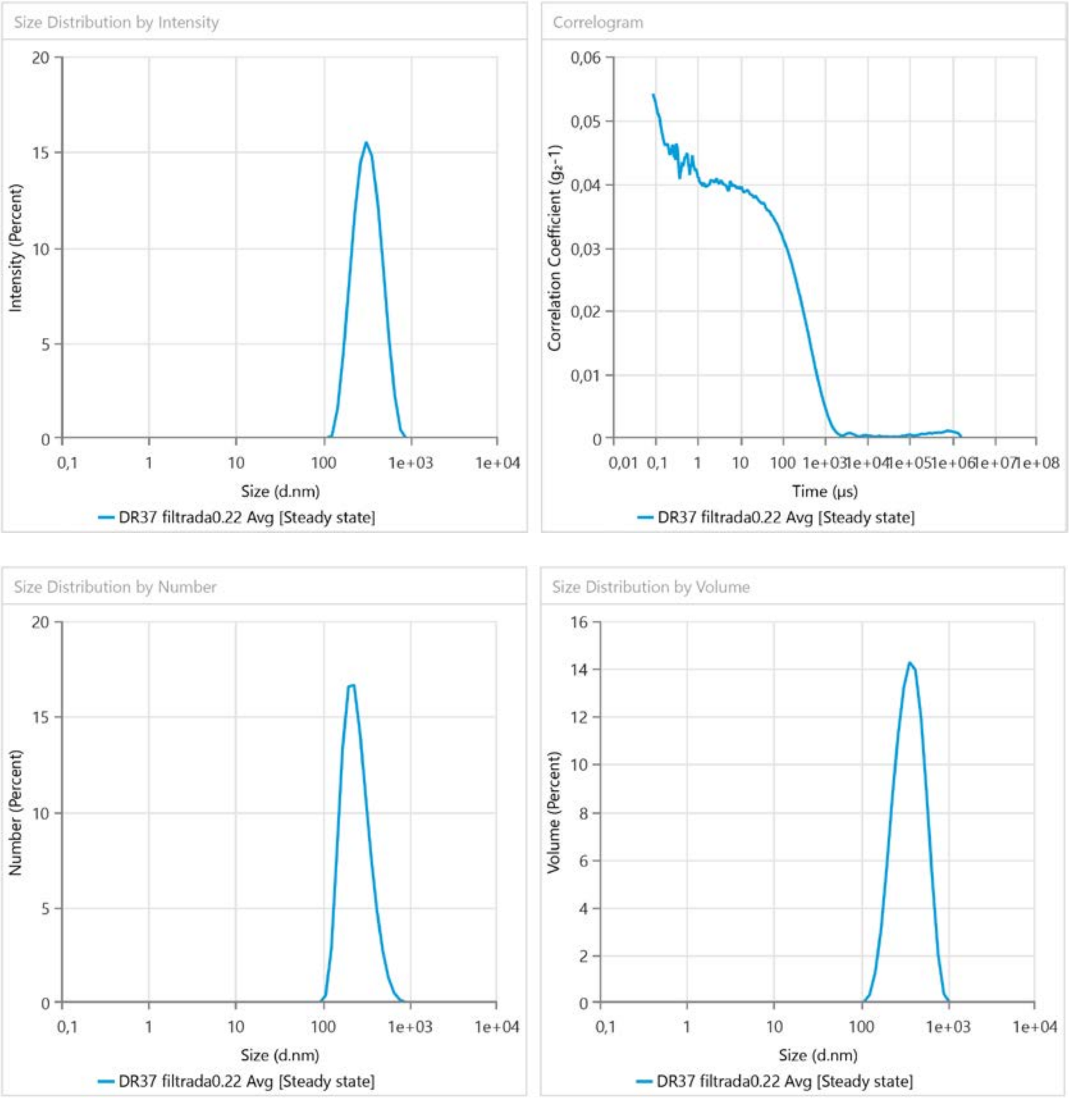

Parameter List

**Z-Average (nm)**	: 288,6
**Polydispersity Index (PI)**	: 0,1305
**Intercept**	: 0,1988
**Peak One Mean by Intensity (nm)**	: 337
**Peak Two Mean by Intensity (nm)**	:
**Peak Three Mean by Intensity (nm)**	:
**Peak One Mean by Number (nm)**	: 254,2
**Peak Two Mean by Number (nm)**	:
**Peak Three Mean by Number (nm)**	:
**Instrument Serial Number**	: MAL1259671
**Software Version**	: 2.0.1.1

Parameter List

**In Range (%)**	: 92,86
**Fit Error**	: 0,0008831
**Run Retention (%)**	: 93,94
**Run Duration (s)**	: 1,68
**Number Of Runs**	: 15
**Derived Mean Count Rate (kcps)**	: 353,2
**Attenuator**	: 11
**Cuvette Position (mm)**	: 3
**Detector Angle (°)**	: 173
**Seek Optimum Attenuator Position**	: True

Figure S100: DLS results, sample of 8 prepared in water 0.1 µg/mL, filtered 0.22 µm.

DLS Forward, as prepared, three repetitions, superposed:

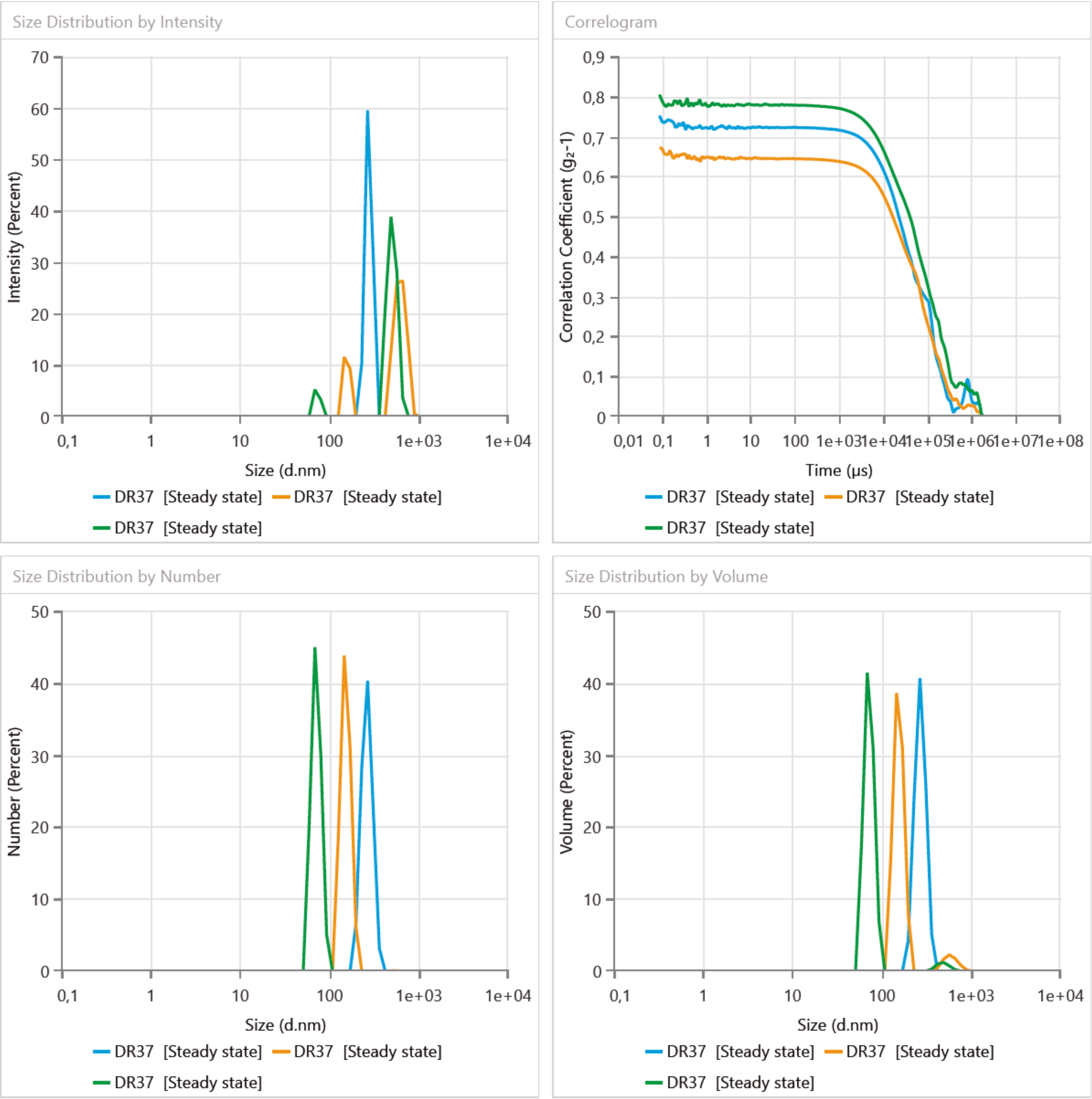

Parameter List

**Z-Average (nm)**	: 337,6
**Polydispersity Index (PI)**	: 0,4698
**Intercept**	: 0,7352
**Peak One Mean by Intensity (nm)**	: 276,5
**Peak Two Mean by Intensity (nm)**	:
**Peak Three Mean by Intensity (nm)**	:
**Peak One Mean by Number (nm)**	: 264,4
**Peak Two Mean by Number (nm)**	:
**Peak Three Mean by Number (nm)**	:
**Instrument Serial Number**	: MAL1259671
**Software Version**	: 2.0.1.1

Parameter List

**In Range (%)**	: 59,71
**Fit Error**	: 0,004276
**Run Retention (%)**	: 56
**Run Duration (s)**	: 1,68
**Number Of Runs**	: 25
**Derived Mean Count Rate (kcps)**	: 7764
**Attenuator**	: 9
**Cuvette Position (mm)**	: 4,64
**Detector Angle (°)**	: 17
**Seek Optimum Attenuator Position**	: True

Figure S101: DLS results, sample of 8 prepared in water 0.1 µg/mL

Tests with cucurbiturils: The compound was dissolved in water. These tests resulted positive for **8** with **CB[7]**.

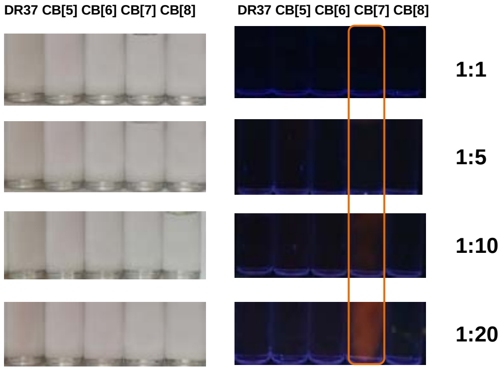

Figure S102: Photos were taken under white (left) and 366 nm (right) lights. From top to bottom, molar proportion between dye and cucurbituril of 1:1, 1:5, 1:10 and 1:20. Each tube in every image correspond (from left to right) to: dye, dye with CB[5], CB[6], CB[7] and CB[8].

Lambert-Beer study:

Compound was dissolved in water, in a concentration range of 1 to 1000 µM (10 points). Absorbance (excitation wavelength 453 nm) was measured for each concentration. The selected work concentration was 10 µM.

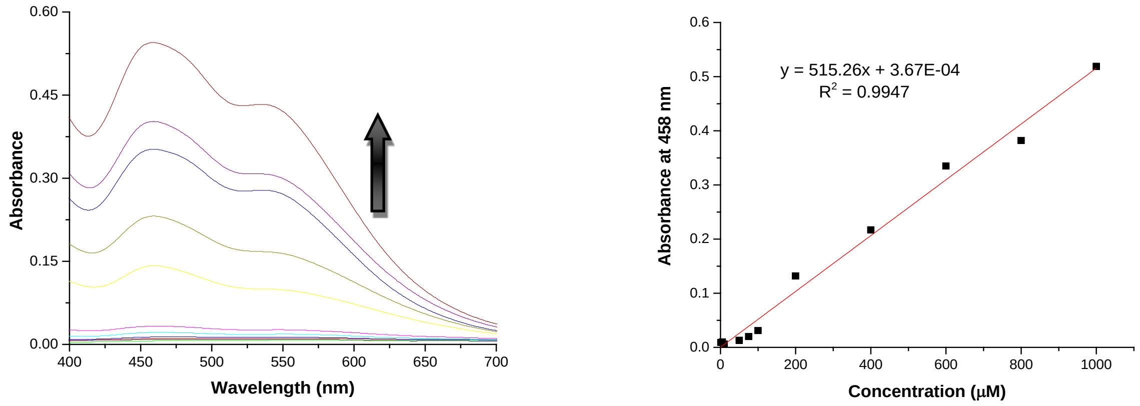

Figure S103: Up: Left: Absorbance spectra at different concentrations. Right: Representation of absorbance maxima versus concentration. Down: Left: Emission spectra at different concentrations. Right: Representation of emission maxima versus concentration.

Tests of 8 with cations, anions, oxidizing and reducing agents: The compound was dissolved in water, 10 µM. These tests resulted **negative** for all experiments.

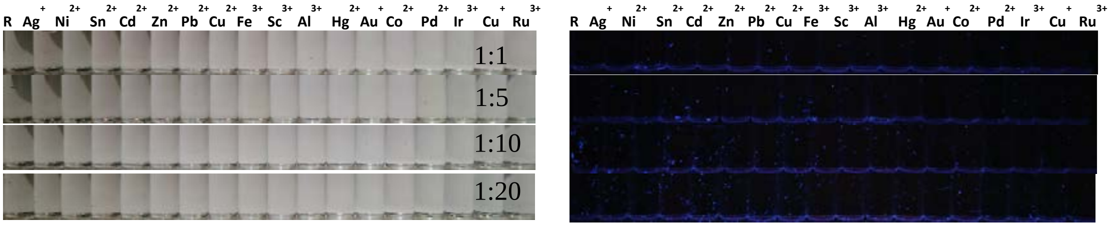

Figure S104: Photos were taken under white (left) and 366 nm (right) lights. From top to bottom, molar proportion between dye and cations of 1:1, 1:5, 1:10 and 1:20. Each tube in every image corresponds (from left to right) to: dye (R), dye with Ag^{+}, Ni^{2+}, Sn^{2+}, Cd^{2+}, Zn^{2+}, Pb^{2+}, Cu^{2+}, Fe^{3+}, Sc^{3+}, Al^{3+}, Hg^{2+}, Au^{+}, Co^{2+}, Pd^{2+}, Ir^{3+}, Cu^{+} and Ru^{3+}.

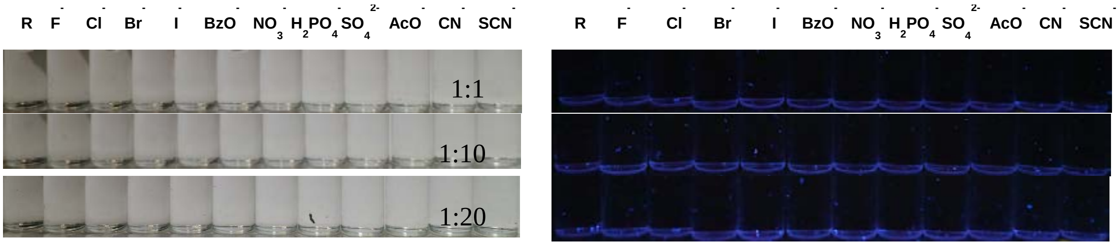

Figure S105: Photos were taken under white (left) and 366 nm (right) lights. From top to bottom, molar proportion between dye and anions of 1:1, 1:10 and 1:20. Each tube in every image corresponds (from left to right) to: dye (R), dye with F^{-}, Cl^{-}, Br^{-}, I^{-}, BzO^{-}, NO_3^{-}, $H_2PO_4^{-}$, SO_4^{2-}, AcO^{-}, CN^{-} and SCN^{-}.

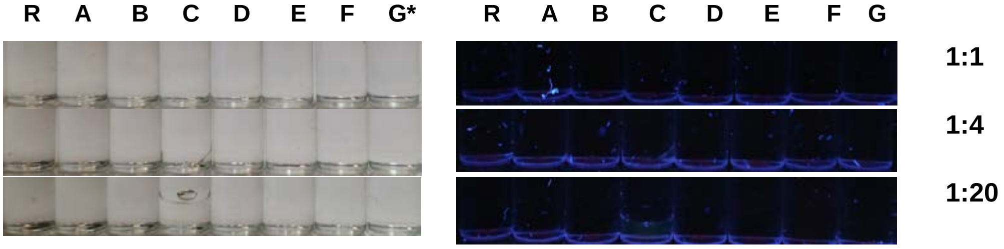

Figure S106: Photos were taken under white (left) and 366 nm (right) lights. From top to bottom, molar proportion between dye and oxidizing of 1:1, 1:4 and 1:20. Each tube in every image correspond (from left to right) to: dye (R), dye with HCl (A), HNO_3 (B), *m*-CPBA (C), oxone (D), hydrazine (E), TNB (F) and H_2O_2 (G).

Test of pH effect: This test showed that compound was not affected by change in pH.

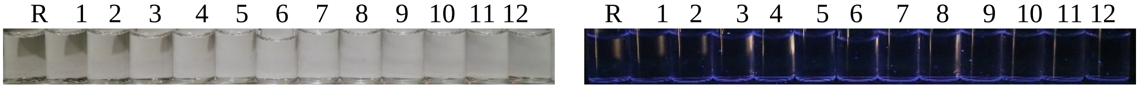

Figure S107: Photos were taken under white (left) and 366 nm (right) lights. Each tube in every image correspond (from left to right) to: dye (R), dye in buffer of pH 5.44 (1), 5.68 (2), 6.77 (3), 6.94 (4), 7.07 (5), 7.09 (6), 7.25 (7), 7.40 (8), 7.46 (9), 7.96 (10), 9.14 (11) and 10.49 (12).

Emission spectra corresponding to the test of pH effect of compound 8.

The compound was dissolved in water at a concentration of 10µM and subjected to pH buffer solutions (HEPES) of pH between 5 and 11. Measurements correspond to dye (R), dye in buffer of pH 5.44 (1), 5.68 (2), 6.77 (3), 6.94(4), 7.07 (5), 7.09 (6), 7.25 (7), 7.40 (8), 7.46 (9), 7.96 (10), 9.14 (11) and 10.49 (12). Excitation wavelength was 439 nm. **Figure S108** shows that the emission of compound **8** was not affected by changes in pH values.

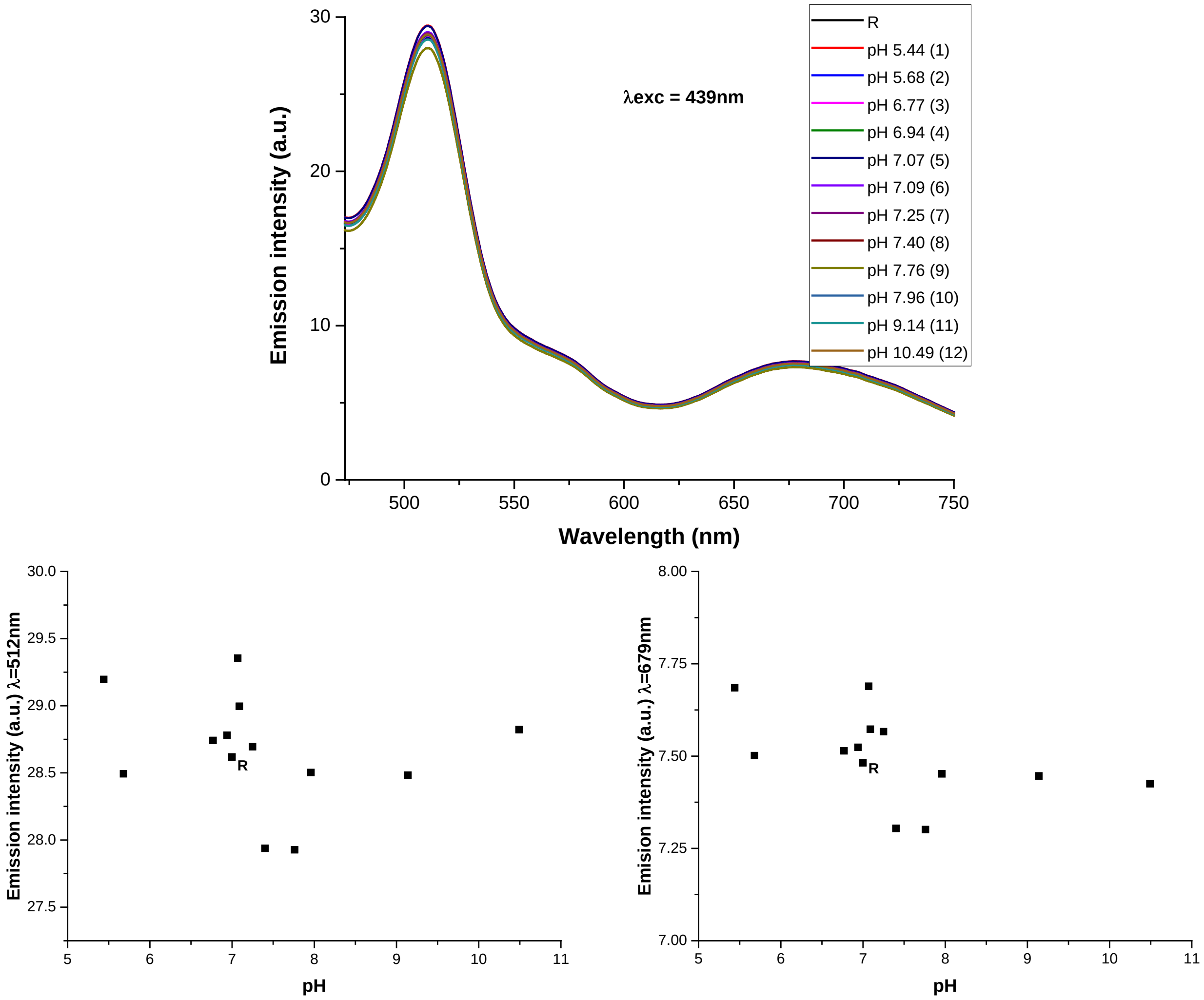

Figure S108. Emission spectra of 8 (10 µM) in increasing pH buffer solutions (up) and representation of emission versus pH at λ = 512 nm (down left) and 679 nm (down right) λexc = 439 nm. The reference R in water was given an arbitrary pH = 7 to be introduced for comparative purposes.

Titration experiments.

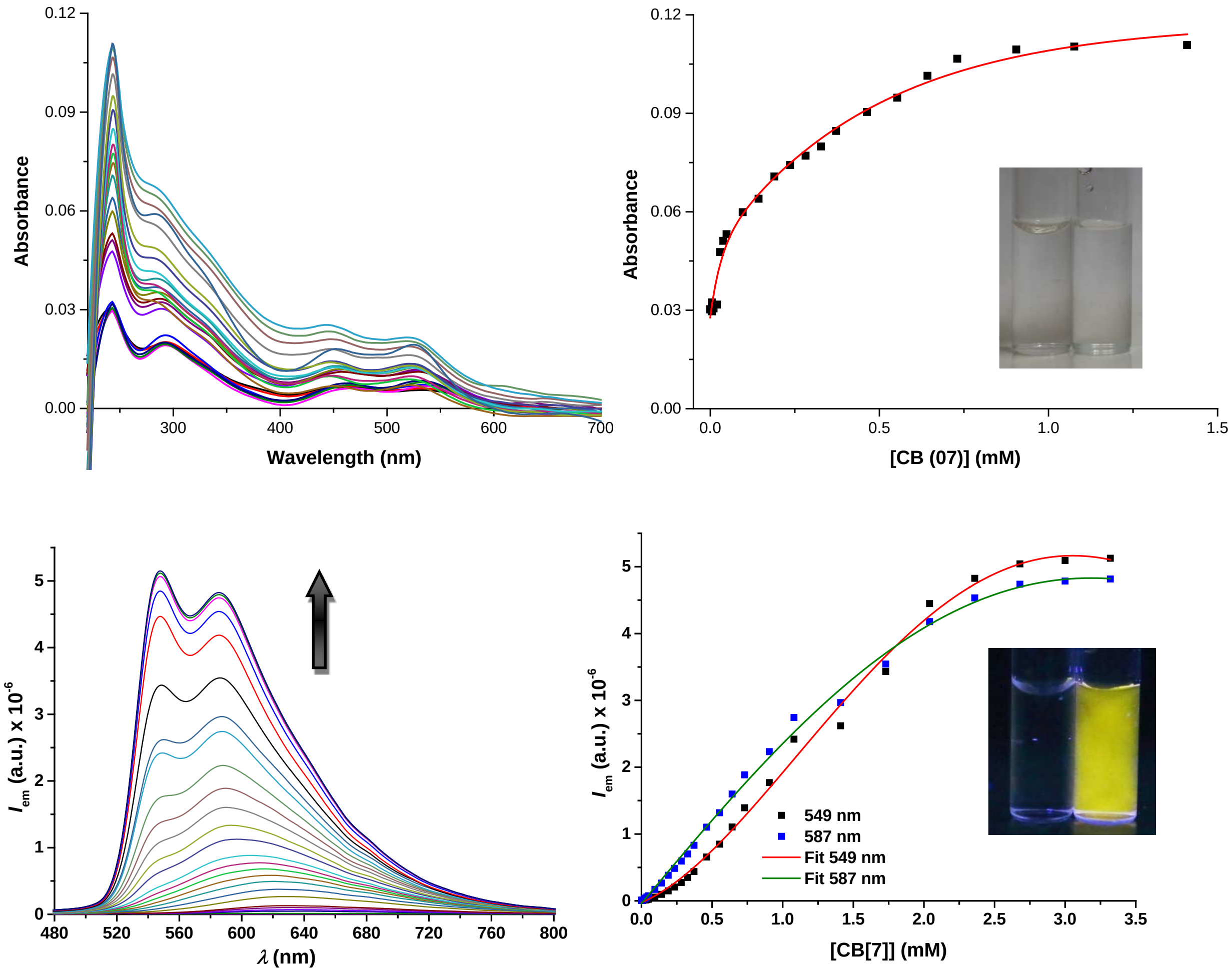

Figure S109: (top) Absorption titration curve and titration profile; (bottom) fluorescence titration curve and titration profile between 8 and CB[7] in water (10 μM 8). Insertion images: absorption of 8 (10 μM, top left) and after addition of CB[7] (top right), fluorescence of 8 (10 μM, bottom left), and after addition of CB [7].

Further characterization studies:

We experienced troubles with NMR characterization of the derivatives because of the tendency to aggregate in solution. See for example figures of compared ^{1}H RMN and ^{13}C RMN spectra of 1, 3, 4 and 5, and ^{1}H RMN spectra of 6, 7, and 8.

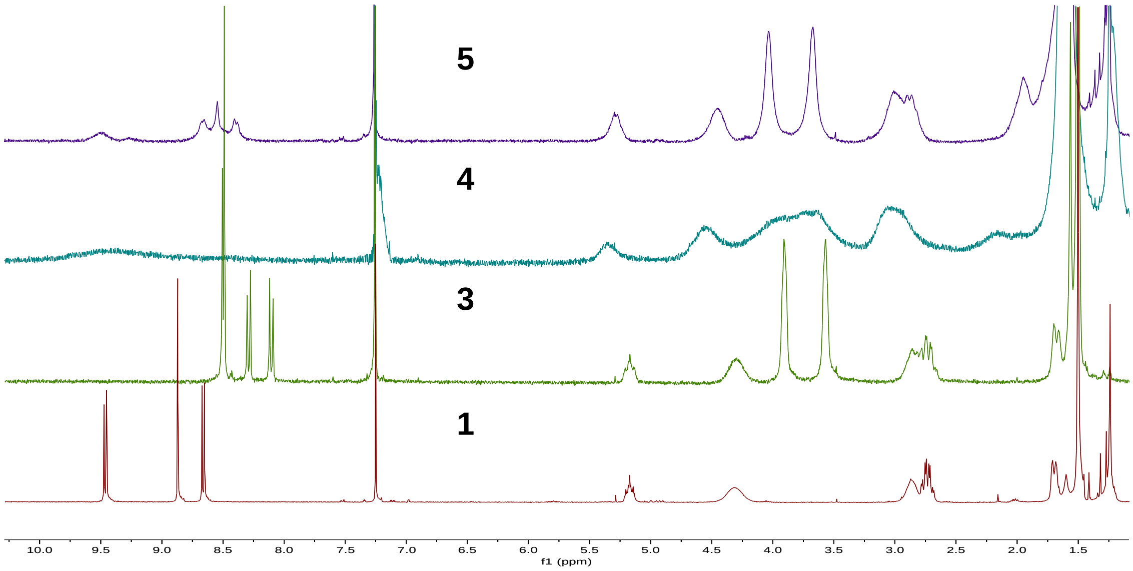

Figure S97: ^{1}H RMN spectra of 1, 3, 4 and 5.

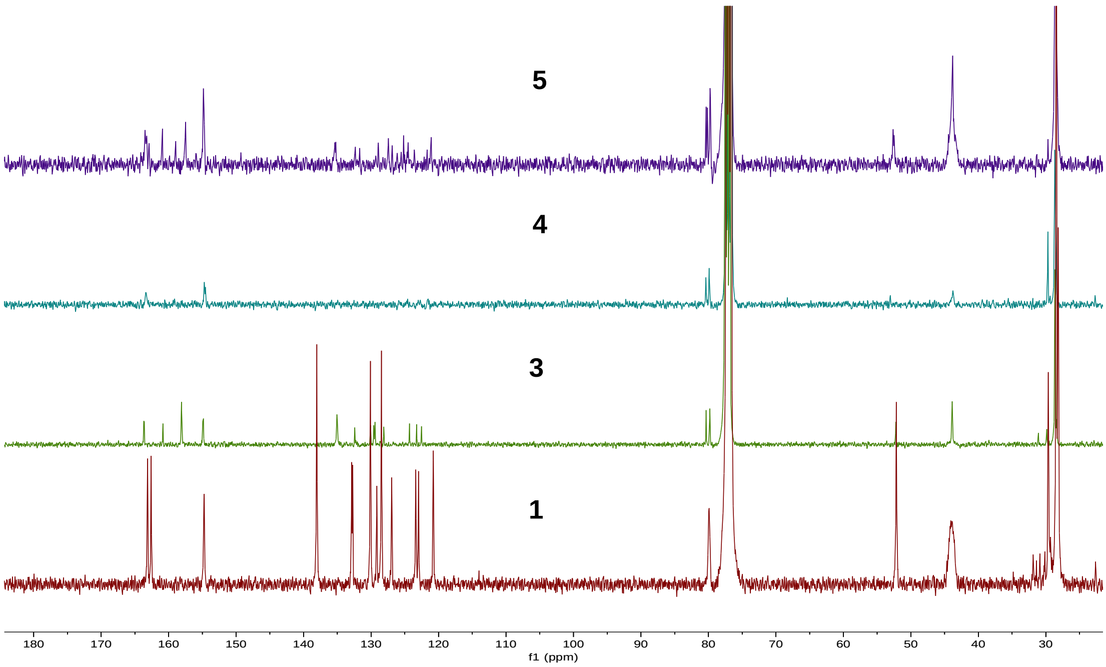

Figure S98: ^{13}C RMN spectra of 1, 3, 4 and 5.

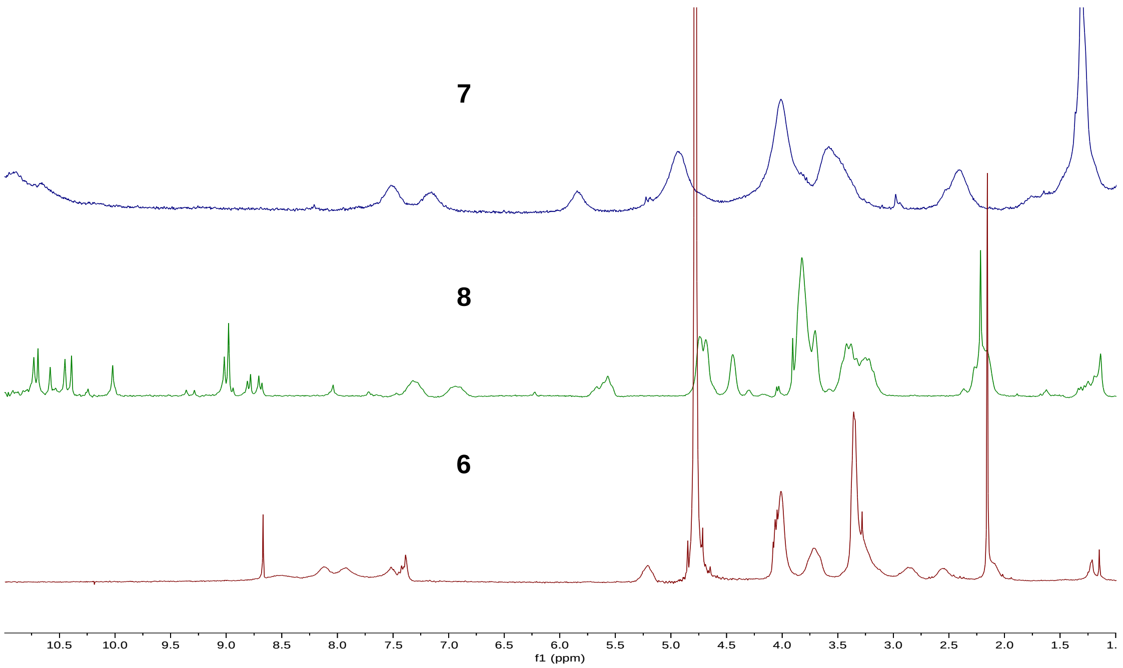

Figure S99: ^{1}H RMN spectra of 6, 7 and 8.

In order to get a compound easily characterizable by NMR we prepared a derivative by amidation with an Fmoc polyethyleneglycol acid.

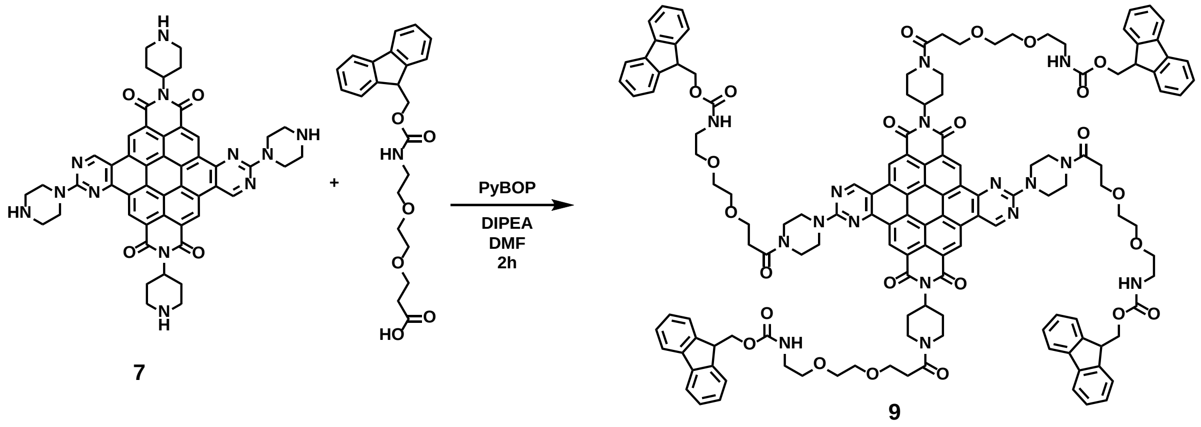

Figure S100: Synthesis of 9.

Synthesis:

25.0 mg (0.20 mmol) of *N,N*-diisopropylethylamine were added to a stirred solution of 1-(9*H*-fluoren-9-yl)-3-oxo-2,7,10-trioxa-4-azatridecan-13-oic acid (39.0 mg, 0.09 mmol) dissolved in 10 ml of DMF. Then, *N,N'*-bis-(piperidin-4-yl)-[3,4-e]-[9,10-e]-bis-((1,4-piperazin-1-yl)-1,3-pyrimidin)coronene-1,12:6,7-tetracarboxylic diimide **7** (21.0 mg, 0.02 mmol) dissolved in DMF (2.0 ml) was added to the previous solution. Finally, 51.0 mg (0.09 mmol) of benzotriazol-1-yl-oxytripyrrolidinophosphonium hexafluorophosphate PyBOP was added. The reaction mixture was stirred at 25 C for two hours. The solvent was removed under vacuum and the residue was subjected to column chromatography (silica gel, CH_2Cl_2:MeCN:MeOH, 5:1:1) to afford the coronenediimide **9** as a red wine solid, obtained in 85% yield (49 mg).

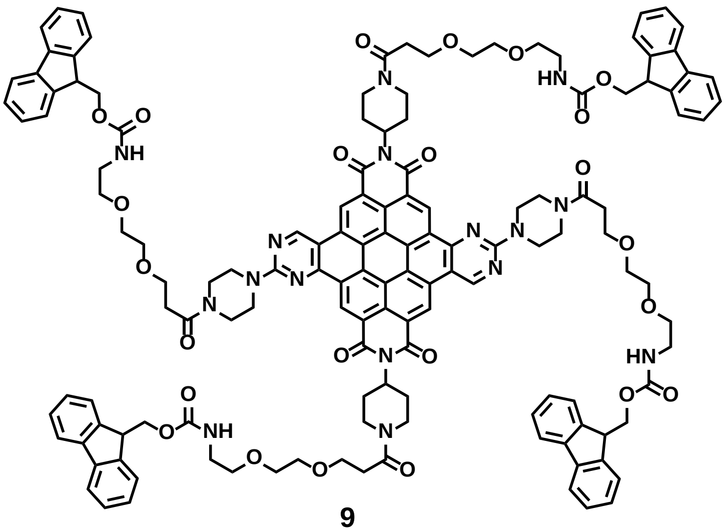

9

***N*,*N’*-Bis-(1-(1-(9H-fluoren-9-yl)-3-oxo-2,7,10-trioxa-4-azatridecan-13-carbonyl)piperidin-4-yl)-[3,4-e]-[9,10-e]-bis-((1,4-(4-(1-(9H-fluoren-9-yl)-3-oxo-2,7,10-trioxa-4-azatridecan-13-carbonyl))piperazin-1-yl)-1,3-pyrimidin)coronene-1,12:6,7-tetracarboxylic diimide 9**. MP (ºC): >350 ºC. R_f (CH_2Cl_2:MeOH, 50:4): 0.38. FT-IR (KBr, cm^{-1}): 3428 (N-H, amine), 2961 (C-H, aromatic), 2877 (C-H, aliphatic), 1710 (C=O, carbamate), 1630 (C=O, imide), 1532 (C=C), 1452, 1400 (C-N), 1386, 1257, 1205, 1131(C-N), 1090. ^{1}H NMR (400 MHz, $CDCl_3$) *δ*: 7.78 – 7.70 (m, 10H, Ar-H), 7.62 – 7.53 (m, 10H, Ar-H), 7.38 – 7.27 (m, 18H, Ar-H), 5.72 – 5.58 (m, 2H, N-CH), 4.37 – 4.10 (m, 12H), 3.74 – 3.31 (m, 22H), 3.10 – 3.05 (m, 22H), 2.62 – 2.35 (m, 10H), 1.79 – 1.76 (m, 22H), 1.37 – 1.35 (m, 8H). ^{13}C NMR (101 MHz, $CDCl_3$) *δ*: 171.5 (C), 170.0 (C), 157.3 (C), 144.1 (C), 144.0 (C), 142.3 (C), 141.3 (C), 141.3 (C), 128.3 (C), 127.8 (CH), 127.2 (CH), 127.1 (CH), 125.8 (CH), 125.3 (CH), 125.0 (CH), 120.0 (CH), 118.0 (CH), 111.0 (CH), 70.7 (CH_2), 70.2 (CH_2), 70.2 (CH_2), 70.0 (CH_2), 69.9 (CH_2), 67.1 (CH_2), 67.0 (CH_2), 55.0 (CH), 46.4 (CH_2), 46.4 (CH_2), 43.1 (CH_2), 26.5 (CH_2), 26.4 (CH_2), 18.9 (CH). HR-MS (MALDI+, DIT): *m/z* calcd. for $C_{138}H_{136}N_{16}O_{24}Na$ ($[M+Na]^+$): 2423.9806; found: 2423.9864. UV-VIS (CH_2Cl_2) λ_{max} / nm (ε / $M^{-1}\cdot cm^{-1}$): 450 (56300). Emission (CH_2Cl_2, λ_{ex} = 450 nm) λ_{max} / nm: 539. τ / ns (CH_2Cl_2, χ2): 1.32 (18.04%) and 6.91 (81.96%) (1.11). Φ (CH_2Cl_2, λ_{ex} = 450 nm): 0.02± 0.01.

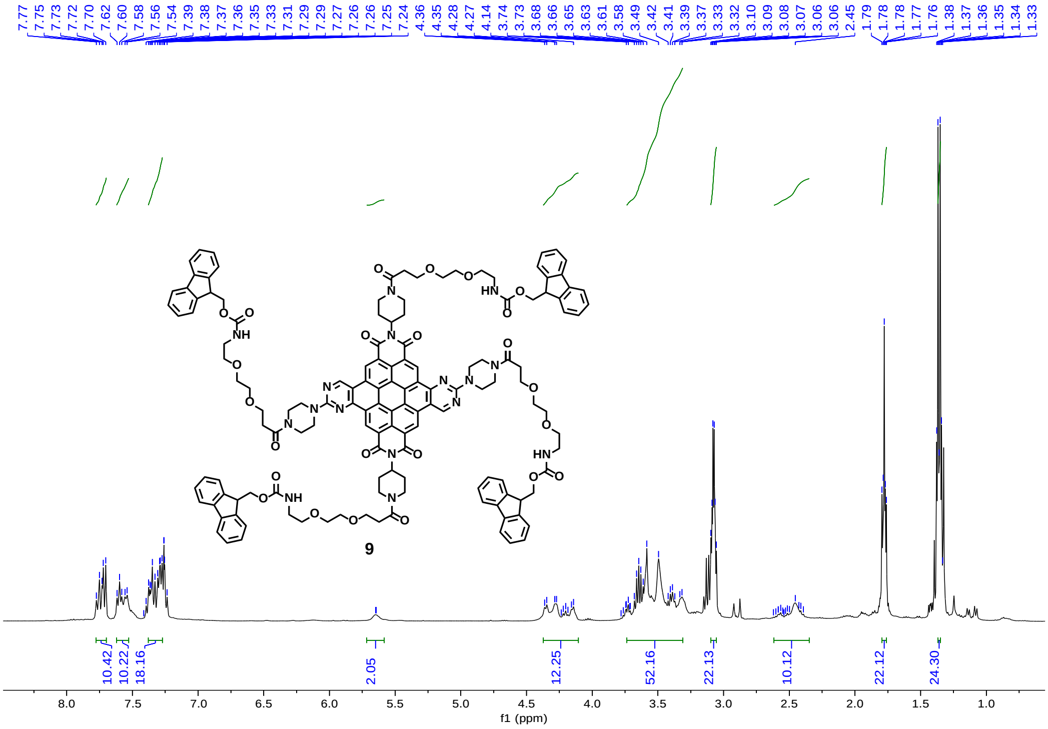

Figure S101: ^{1}H NMR (400 MHz, $CDCl_3$) of 9.

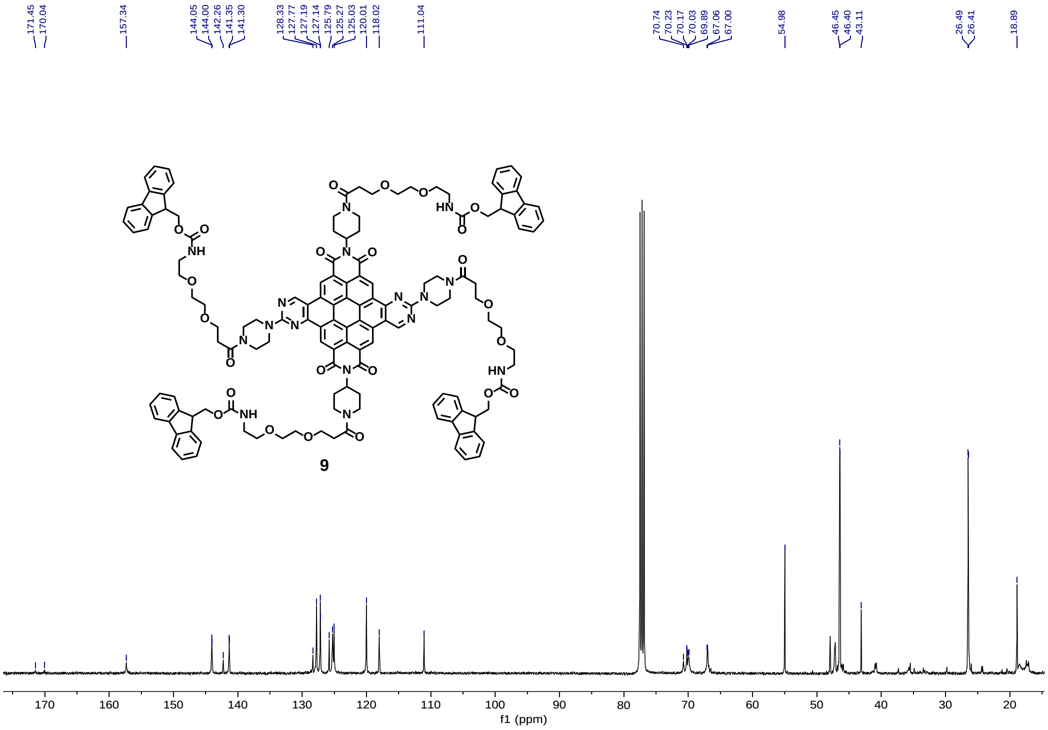

Figure S102: ^{13}C NMR (101 MHz, $CDCl_3$) of 9.

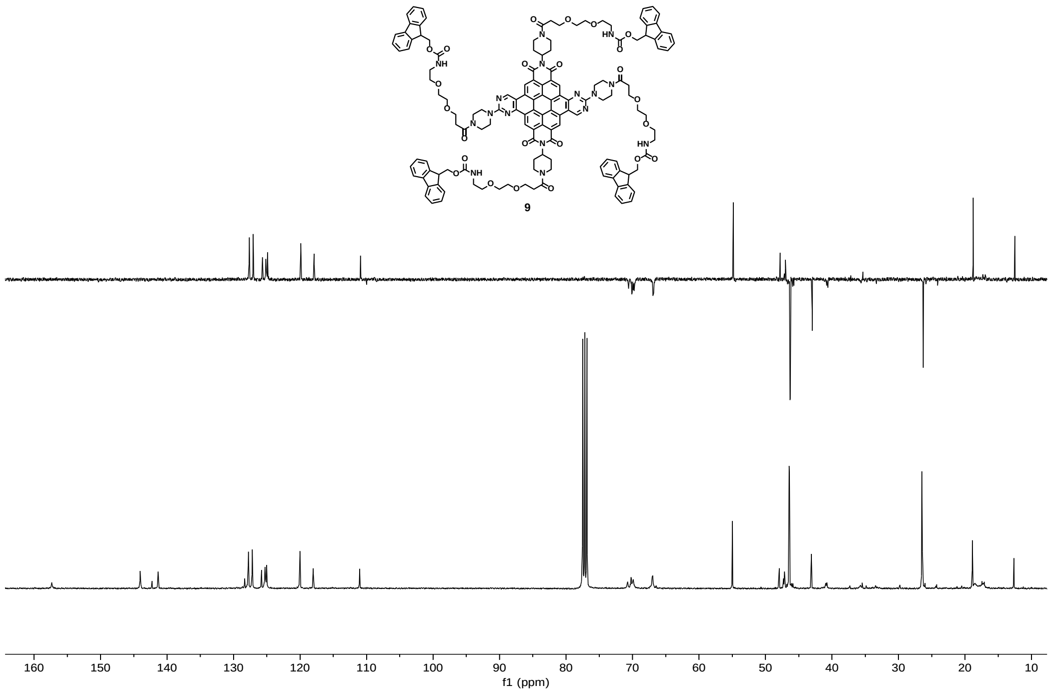

Figure S103: ^{13}C-DEPT 135 NMR (101 MHz, $CDCl_3$) of 9.

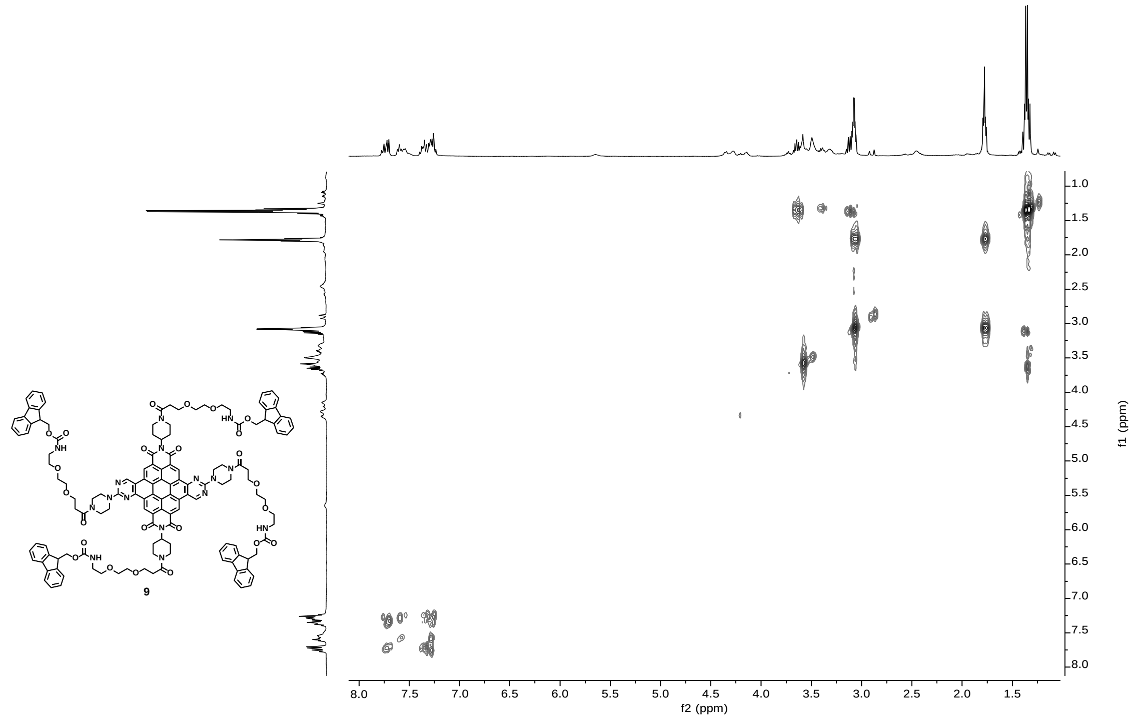

Figure S104: ^{1}H-^{1}H-COSY NMR (300MHz, $CDCl_3$) of 9.

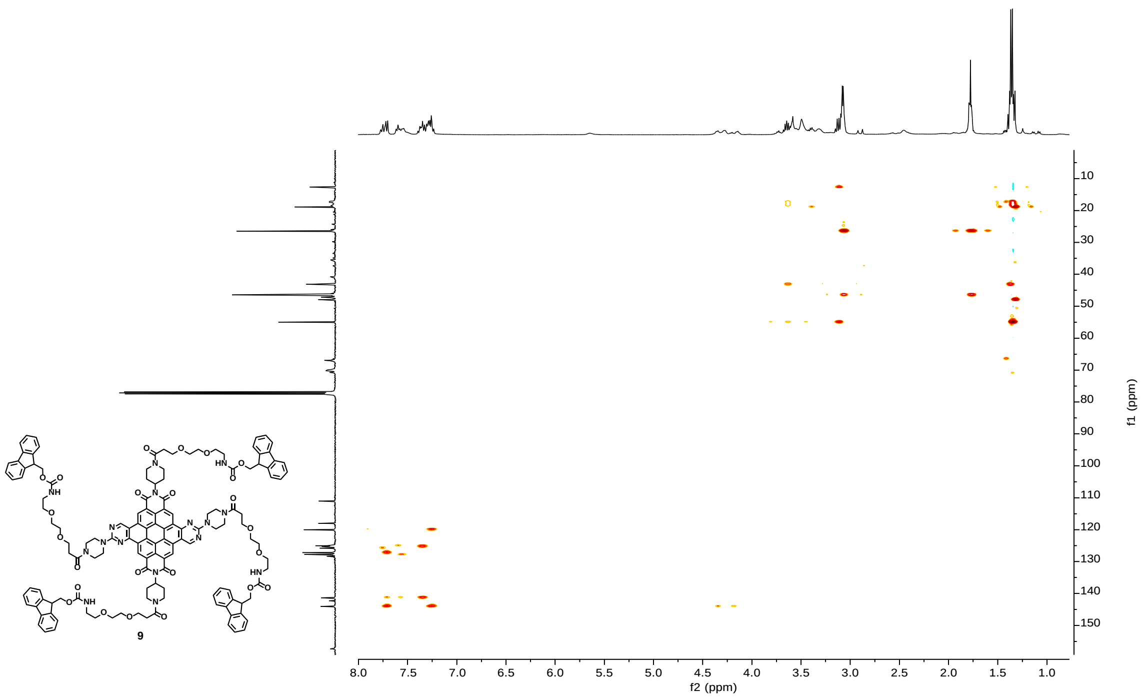

Figure S105: ^{1}H-^{13}C-HMBC NMR (300MHz, $CDCl_3$) of 9.

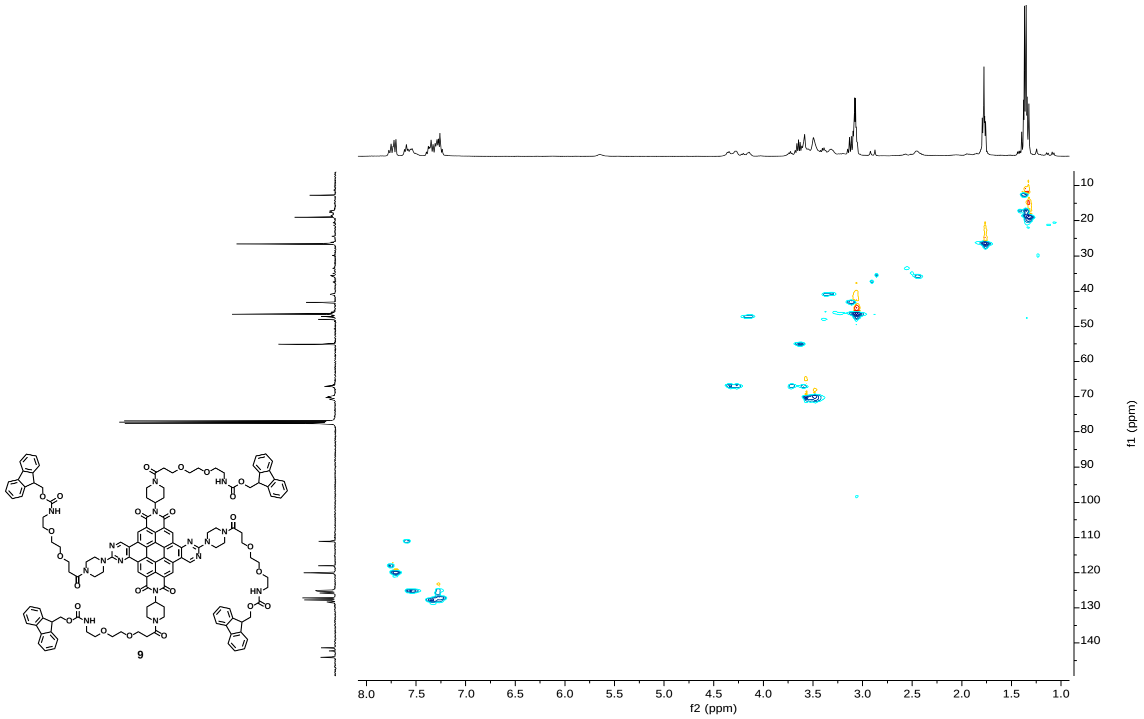

Figure S106: ^{1}H-^{13}C-HMQC NMR (300 MHz, $CDCl_3$) of 9.

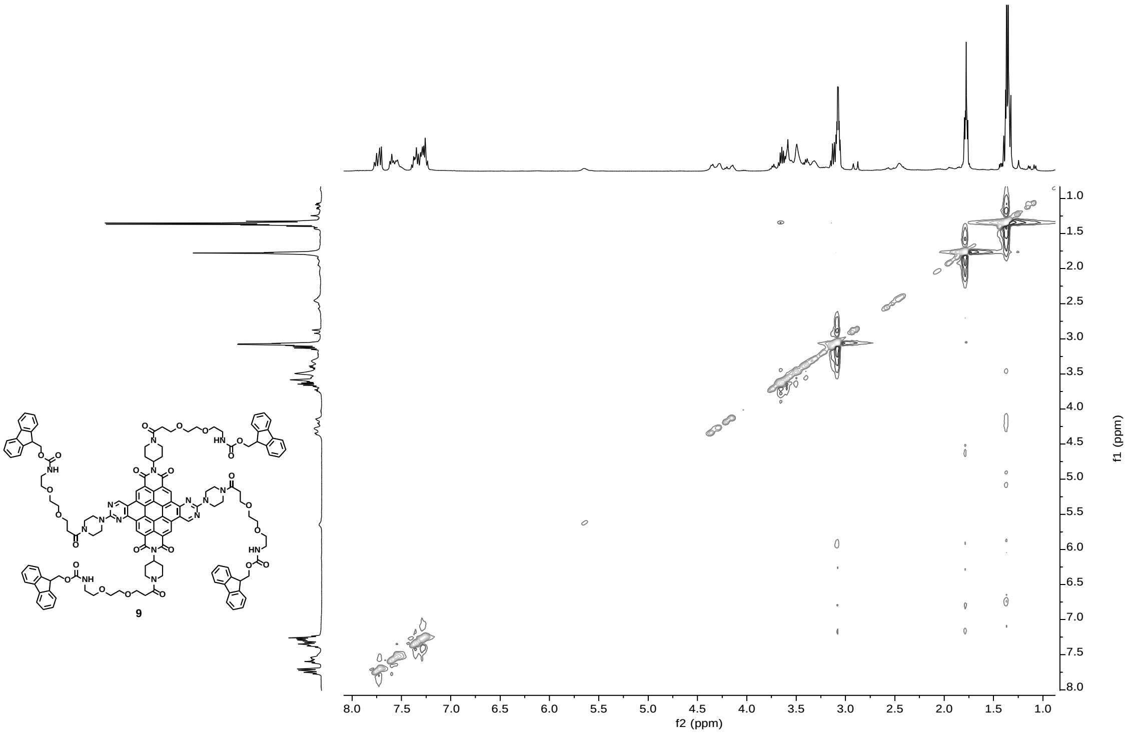

Figure S107: ^{1}H-^{1}H-NOESY NMR (300 MHz, $CDCl_3$) of 9.

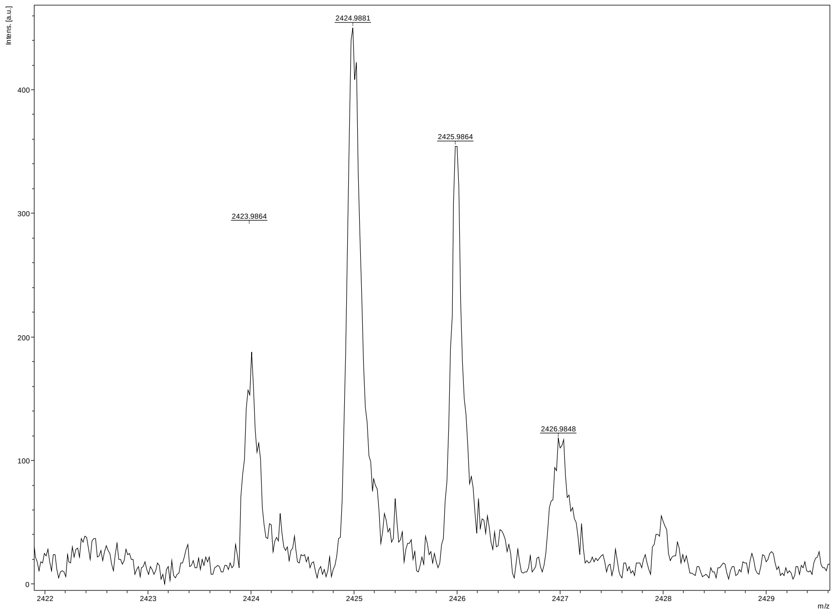

Figure S108: HRMS (MALDI+, DIT) of 9.

Solvatochromism tests:

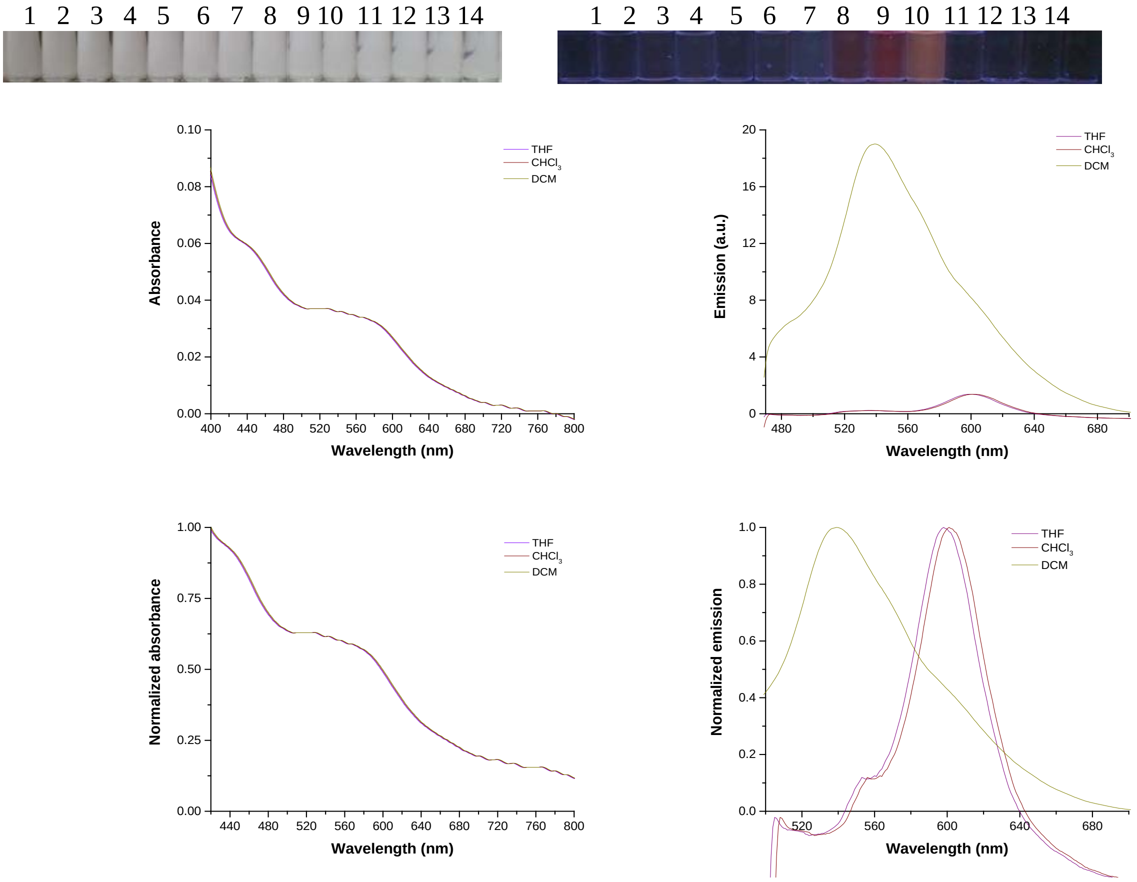

Figure S109: Solvatochromism test: Up: Photos under white and 366 nm lights. Medium: Absorption (left) and emission spectra (right) under excitation wavelength of 450 nm. Down: Normalized absorption spectrum (left) and normalized emission one (right) under excitation wavelength of 450 nm. The employed solvents were: 1: H_2O, 2: MeOH (methanol), 3: DMSO (dimethylsulfoxide), 4: DMF (*N*,*N'*-dimethylformamide), 5: MeCN (acetonitrile), 6: Acetone, 7: EtAcO (ethyl acetate), 8: THF (tetrahydrofuran), 9: $CHCl_3$, 10: CH_2Cl_2 (dichloromethane), 11: Toluene, 12: Et_2O (diethyl ether), 13: *n*-Hx (hexane), 14: *c*-Hx (cyclohexane).

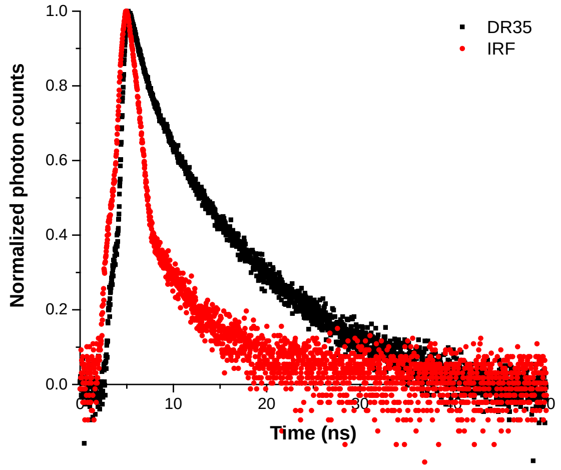

Figure S110: Normalized emission lifetime decay curve of fluorophore 9 (black) and blank (red). The laser employed was 539 nm and the emission wavelength 445 nm.

Theoretical calculations. Calculations with coronene **7**, CB[7] and TNB were performed using Gaussian 09. Revision D.01.[1] Each structure was verified to be a true minimum by the absence of imaginary frequencies in the vibrational analysis. As we are dealing with a supramolecular system, dispersion-accounting DFT approach is appropriate to obtain quantitative predictions on noncovalent interactions with chemical accuracy.[2-4] The hybrid exchange-correlation functional PBE0[5] was employed in combination with the D3 version of Grimme's dispersion with the original D3 damping function.[6] The double-zeta Pople's 6-31G** basis set[7-8] was employed throughout. The interaction energy (E_{int}) was defined as the energy difference between the host-guest complexes and the individual moieties separately, all of them at the geometry of the complex. The basis set superposition error (BSSE) was corrected according to the counterpoise scheme.[9-10]

Table S2. Cartesian coordinates of TNB

Center Number	Atomic Number	Atomic Type	Coordinates (Angstroms) X	Y	Z
1	6	0	0.633830	-1.212009	-0.000012
2	6	0	1.403644	-0.058550	-0.000011
3	6	0	0.732574	1.155033	-0.000009
4	6	0	-0.651363	1.245005	-0.000012
5	6	0	-1.366802	0.057065	-0.000011
6	6	0	-0.752734	-1.186357	-0.000009
7	1	0	2.485898	-0.103770	0.000001
8	1	0	-1.153359	2.204864	-0.000012
9	1	0	-1.332952	-2.101059	-0.000017
10	7	0	1.315466	-2.515319	-0.000012
11	8	0	0.610389	-3.507473	0.000002
12	8	0	2.532571	-2.502064	0.000009
13	7	0	1.520933	2.396681	-0.000006
14	8	0	0.901556	3.444446	-0.000058
15	8	0	2.732624	2.281494	0.000070
16	7	0	-2.836327	0.118603	-0.000009
17	8	0	-3.433687	-0.941902	0.000001
18	8	0	-3.342826	1.225385	0.000049

Zero-point correction = 0.109636 (Hartree/Particle)
Thermal correction to Energy = 0.121497
Thermal correction to Enthalpy = 0.122441
Thermal correction to Gibbs Free Energy = 0.069473
Sum of electronic and zero-point Energies = -844.745242
Sum of electronic and thermal Energies = -844.733381
Sum of electronic and thermal Enthalpies = -844.732437
Sum of electronic and thermal Free Energies = -844.785404

Table S3. Cartesian coordinates of [7]

Center Number	Atomic Number	Atomic Type	Coordinates (Angstroms) X	Y	Z
1	6	0	3.703428	0.517949	-0.097484
2	6	0	2.527937	1.293573	-0.098613
3	6	0	1.266744	0.663726	-0.099595
4	6	0	1.192548	-0.757774	-0.099501
5	6	0	2.386877	-1.509563	-0.099272
6	6	0	3.638411	-0.857021	-0.097632
7	6	0	0.058848	1.431737	-0.101360
8	6	0	-0.058758	-1.431717	-0.100933
9	6	0	-1.266654	-0.663705	-0.099262
10	6	0	-1.192457	0.757795	-0.099581
11	6	0	-2.527848	-1.293551	-0.097970
12	6	0	-0.109281	-2.844868	-0.105209
13	6	0	1.107036	-3.564554	-0.104577
14	6	0	2.321602	-2.918633	-0.101473
15	1	0	1.128439	-4.648833	-0.105496
16	1	0	4.668213	1.011482	-0.098217
17	6	0	0.109372	2.844887	-0.106109
18	6	0	-1.106946	3.564571	-0.105863
19	6	0	-2.321511	2.918651	-0.102332
20	6	0	-2.386786	1.509583	-0.099402
21	1	0	-1.128353	4.648849	-0.107464
22	6	0	-3.703340	-0.517926	-0.096749
23	1	0	-4.668124	-1.011462	-0.097115
24	6	0	-3.638321	0.857043	-0.097231
25	6	0	3.563066	-3.728859	-0.100820
26	6	0	4.892562	-1.642044	-0.096512
27	6	0	-4.892469	1.642069	-0.095753
28	6	0	-3.562975	3.728878	-0.102130
29	8	0	3.519814	-4.949154	-0.103056
30	8	0	5.983638	-1.098940	-0.094508
31	8	0	-3.519720	4.949170	-0.106138
32	8	0	-5.983548	1.098972	-0.093247
33	7	0	4.776208	-3.042474	-0.097653
34	7	0	-4.776112	3.042502	-0.097052
35	6	0	2.594937	2.741837	-0.102215
36	6	0	1.396504	3.491444	-0.109689
37	6	0	-1.396417	-3.491426	-0.108703
38	6	0	-2.594847	-2.741814	-0.101389
39	7	0	3.800764	3.320345	-0.103057
40	6	0	3.845890	4.653409	-0.110997
41	7	0	2.757979	5.473686	-0.132007
42	6	0	1.585518	4.890669	-0.129222
43	1	0	0.729152	5.562864	-0.146152
44	7	0	-3.800675	-3.320321	-0.102294

45	6	0	-3.845805	-4.653383	-0.110215
46	7	0	-2.757893	-5.473665	-0.131096
47	6	0	-1.585430	-4.890650	-0.128193
48	1	0	-0.729066	-5.562848	-0.145044
49	6	0	-6.038704	3.812675	-0.094758
50	6	0	-6.217980	4.658097	1.166325
51	6	0	-6.228163	4.648110	-1.360963
52	1	0	-6.810230	3.037702	-0.088569
53	6	0	-7.576234	5.366612	1.107723
54	1	0	-5.415516	5.398814	1.235640
55	1	0	-6.161606	4.017016	2.054664
56	6	0	-7.585762	5.357432	-1.297108
57	1	0	-5.426029	5.387968	-1.442670
58	1	0	-6.179112	3.999926	-2.244576
59	1	0	-7.700465	6.016327	1.980669
60	1	0	-8.380671	4.618632	1.156845
61	1	0	-7.716779	6.000492	-2.173993
62	1	0	-8.390628	4.609210	-1.334185
63	6	0	6.038799	-3.812644	-0.096058
64	6	0	6.220097	-4.655712	1.166301
65	6	0	6.226247	-4.650436	-1.361014
66	1	0	6.810334	-3.037664	-0.092546
67	6	0	7.578227	-5.364398	1.106880
68	1	0	5.417680	-5.396236	1.238305
69	1	0	6.165186	-4.012950	2.053515
70	6	0	7.584010	-5.359519	-1.297978
71	1	0	5.424081	-5.390536	-1.440039
72	1	0	6.175714	-4.003926	-2.245772
73	1	0	7.703783	-6.012562	1.980788
74	1	0	8.382747	-4.616342	1.153439
75	1	0	7.713728	-6.004131	-2.173916
76	1	0	8.388767	-4.611310	-1.337622
77	6	0	6.299972	4.501611	-0.103172
78	6	0	5.222187	6.698559	-0.182055
79	6	0	7.240418	5.032192	0.979924
80	1	0	6.774783	4.608437	-1.092013
81	1	0	6.066876	3.447561	0.046685
82	6	0	6.185003	7.181994	0.902953
83	1	0	5.631061	6.948576	-1.174862
84	1	0	4.241789	7.166154	-0.091581
85	1	0	8.206933	4.522198	0.909575
86	1	0	6.816632	4.792314	1.963798
87	1	0	6.375794	8.252882	0.775206
88	1	0	5.708094	7.050280	1.882967
89	6	0	-6.299841	-4.501503	-0.103110
90	6	0	-5.222092	-6.698475	-0.181997
91	6	0	-7.241248	-5.032513	0.978929
92	1	0	-6.773838	-4.607794	-1.092408
93	1	0	-6.066808	-3.447538	0.047453

94	6	0	-6.185886	-7.182316	0.901947
95	1	0	-5.630068	-6.948134	-1.175273
96	1	0	-4.241796	-7.166148	-0.090818
97	1	0	-8.207669	-4.522428	0.907951
98	1	0	-6.818314	-4.793091	1.963280
99	1	0	-6.376608	-8.253138	0.773559
100	1	0	-5.709838	-7.051052	1.882439
101	7	0	5.059528	5.257445	-0.092225
102	7	0	-5.059449	-5.257406	-0.091457
103	7	0	7.453128	6.469895	0.923413
104	1	0	7.981725	6.699268	0.085505
105	7	0	-7.453995	-6.470174	0.921609
106	1	0	-7.981900	-6.699165	0.083161
107	7	0	-7.787109	6.155217	-0.096913
108	1	0	-7.132238	6.932911	-0.102503
109	7	0	7.787251	-6.155148	-0.096677
110	1	0	7.132405	-6.932877	-0.099839

Zero-point correction = 0.901383 (Hartree/Particle)
Thermal correction to Energy = 0.951330
Thermal correction to Enthalpy = 0.952274
Thermal correction to Gibbs Free Energy = 0.814364
Sum of electronic and zero-point Energies = -2885.913885
Sum of electronic and thermal Energies = -2885.863937
Sum of electronic and thermal Enthalpies = -2885.862993
Sum of electronic and thermal Free Energies = -2886.000903

Table S4. Cartesian coordinates of [7]_dimer

Center Number	Atomic Number	Atomic Type	Coordinates (Angstroms) X	Y	Z
1	6	0	3.636563	-0.864040	1.777633
2	6	0	2.821871	0.283645	1.752497
3	6	0	1.420188	0.156490	1.775404
4	6	0	0.834540	-1.140712	1.779261
5	6	0	1.673945	-2.273718	1.754015
6	6	0	3.076276	-2.120201	1.777250
7	6	0	0.576947	1.312323	1.755503
8	6	0	-0.574682	-1.314206	1.755837
9	6	0	-1.417909	-0.158357	1.775310
10	6	0	-0.832267	1.138835	1.778865
11	6	0	-2.819586	-0.285504	1.752305
12	6	0	-1.136017	-2.610524	1.682957
13	6	0	-0.263686	-3.720954	1.639207
14	6	0	1.102121	-3.560431	1.675508
15	1	0	-0.637448	-4.735538	1.553477
16	1	0	4.714512	-0.753472	1.772293

17	6	0	1.138262	2.608621	1.682190
18	6	0	0.265933	3.719038	1.638088
19	6	0	-1.099870	3.558524	1.674517
20	6	0	-1.671678	2.271832	1.753304
21	1	0	0.639706	4.733590	1.552053
22	6	0	-3.634296	0.862164	1.777005
23	1	0	-4.712250	0.751598	1.771530
24	6	0	-3.074009	2.118315	1.776441
25	6	0	1.967106	-4.755627	1.545977
26	6	0	3.958611	-3.305726	1.756732
27	6	0	-3.956354	3.303831	1.756056
28	6	0	-1.964873	4.753692	1.544964
29	8	0	1.486177	-5.868538	1.394999
30	8	0	5.167762	-3.201590	1.863618
31	8	0	-1.483982	5.866523	1.393301
32	8	0	-5.165519	3.199583	1.862797
33	7	0	3.345455	-4.556395	1.581131
34	7	0	-3.343223	4.554585	1.581184
35	6	0	3.409547	1.602509	1.642368
36	6	0	2.569957	2.738771	1.605035
37	6	0	-2.567731	-2.740685	1.605959
38	6	0	-3.407273	-1.604387	1.642810
39	7	0	4.737259	1.686496	1.528193
40	6	0	5.266690	2.898556	1.356732
41	7	0	4.555634	4.060513	1.303430
42	6	0	3.255595	3.960211	1.427661
43	1	0	2.706242	4.898382	1.368665
44	7	0	-4.735006	-1.688362	1.528876
45	6	0	-5.264549	-2.900480	1.358336
46	7	0	-4.553557	-4.062511	1.305574
47	6	0	-3.253474	-3.962191	1.429406
48	1	0	-2.704116	-4.900390	1.370818
49	6	0	-4.242243	5.713676	1.404284
50	6	0	-4.099895	6.761301	2.506562
51	6	0	-4.116175	6.329567	0.010862
52	1	0	-5.242052	5.278717	1.485964
53	6	0	-5.104158	7.890994	2.252811
54	1	0	-3.082941	7.164435	2.507730
55	1	0	-4.280495	6.297905	3.484102
56	6	0	-5.113737	7.485843	-0.117480
57	1	0	-3.097767	6.705514	-0.134802
58	1	0	-4.322073	5.561354	-0.743700
59	1	0	-4.987189	8.676779	3.006773
60	1	0	-6.127372	7.502899	2.360262
61	1	0	-5.001583	7.975744	-1.090703
62	1	0	-6.137673	7.086607	-0.084263
63	6	0	4.244468	-5.715165	1.402121
64	6	0	4.104193	-6.763814	2.503697
65	6	0	4.116289	-6.329834	0.008346

66	1	0	5.244303	-5.280050	1.482624
67	6	0	5.108286	-7.893041	2.247192
68	1	0	3.087306	-7.167115	2.506229
69	1	0	4.286370	-6.301270	3.481345
70	6	0	5.113844	-7.485776	-0.122745
71	1	0	3.097731	-6.705876	-0.136012
72	1	0	4.320825	-5.560860	-0.745807
73	1	0	4.992799	-8.679519	3.000660
74	1	0	6.131581	-7.504778	2.353259
75	1	0	5.000135	-7.974827	-1.096217
76	1	0	6.137764	-7.086384	-0.090910
77	6	0	7.450263	1.813343	1.127815
78	6	0	7.260952	4.242095	0.855906
79	6	0	8.752457	2.029601	1.893825
80	1	0	7.655026	1.615471	0.065293
81	1	0	6.893752	0.963086	1.521477
82	6	0	8.569386	4.398224	1.629624
83	1	0	7.469764	4.225297	-0.226918
84	1	0	6.576563	5.066188	1.056966
85	1	0	9.413764	1.170615	1.737163
86	1	0	8.531610	2.079449	2.968020
87	1	0	9.095409	5.293650	1.281528
88	1	0	8.335308	4.549374	2.691484
89	6	0	-7.447941	-1.815013	1.128969
90	6	0	-7.258954	-4.243989	0.858163
91	6	0	-8.750528	-2.030771	1.894432
92	1	0	-7.652147	-1.617381	0.066301
93	1	0	-6.891445	-0.964739	1.522640
94	6	0	-8.567788	-4.399498	1.631308
95	1	0	-7.467248	-4.227756	-0.224780
96	1	0	-6.574841	-5.068112	1.059992
97	1	0	-9.411582	-1.171702	1.737139
98	1	0	-8.530246	-2.080280	2.968759
99	1	0	-9.093792	-5.295000	1.283384
100	1	0	-8.334258	-4.550185	2.693355
101	7	0	6.610742	2.997258	1.228326
102	7	0	-6.608665	-2.999091	1.230250
103	7	0	9.455985	3.250011	1.527430
104	1	0	9.789206	3.168506	0.569792
105	7	0	-9.454123	-3.251172	1.528142
106	1	0	-9.786757	-3.169989	0.570272
107	7	0	-5.001617	8.490321	0.930058
108	1	0	-4.098624	8.950706	0.847839
109	7	0	5.003666	-8.491224	0.924084
110	1	0	4.100588	-8.951640	0.842964
111	6	0	3.251473	1.840904	-1.788510
112	6	0	1.873763	2.128888	-1.797764
113	6	0	0.932851	1.081197	-1.823298
114	6	0	1.385848	-0.267938	-1.823738

115	6	0	2.772213	-0.528147	-1.797634
116	6	0	3.695601	0.539102	-1.788248
117	6	0	-0.471656	1.355218	-1.822222
118	6	0	0.469595	-1.353395	-1.821760
119	6	0	-0.934906	-1.079385	-1.822922
120	6	0	-1.387901	0.269755	-1.823774
121	6	0	-1.875806	-2.127093	-1.797172
122	6	0	0.940871	-2.686271	-1.797432
123	6	0	2.335950	-2.909071	-1.766633
124	6	0	3.228607	-1.862195	-1.763410
125	1	0	2.748485	-3.911031	-1.726148
126	1	0	3.966562	2.655005	-1.766783
127	6	0	-0.942939	2.688106	-1.798372
128	6	0	-2.338028	2.910913	-1.767534
129	6	0	-3.230680	1.864023	-1.763879
130	6	0	-2.774265	0.529967	-1.797709
131	1	0	-2.750559	3.912886	-1.727259
132	6	0	-3.253506	-1.839102	-1.787998
133	1	0	-3.968607	-2.653184	-1.766123
134	6	0	-3.697622	-0.537300	-1.787977
135	6	0	4.679301	-2.154433	-1.683187
136	6	0	5.147829	0.273279	-1.749766
137	6	0	-5.149842	-0.271500	-1.749484
138	6	0	-4.681379	2.156239	-1.683524
139	8	0	5.088829	-3.303352	-1.620165
140	8	0	5.962580	1.180875	-1.773863
141	8	0	-5.090974	3.305148	-1.620944
142	8	0	-5.964572	-1.179129	-1.773530
143	7	0	5.552904	-1.068715	-1.676662
144	7	0	-5.554928	1.070455	-1.676229
145	6	0	1.404193	3.497781	-1.746426
146	6	0	0.016152	3.760895	-1.764263
147	6	0	-0.018201	-3.759060	-1.763045
148	6	0	-1.406247	-3.495987	-1.745667
149	7	0	2.315749	4.471581	-1.656796
150	6	0	1.871012	5.726358	-1.575717
151	7	0	0.562133	6.098252	-1.632979
152	6	0	-0.318370	5.132988	-1.717953
153	1	0	-1.358715	5.451207	-1.746255
154	7	0	-2.317809	-4.469826	-1.656364
155	6	0	-1.873069	-5.724569	-1.574975
156	7	0	-0.564098	-6.096389	-1.631036
157	6	0	0.316390	-5.131106	-1.715895
158	1	0	1.356777	-5.449278	-1.743248
159	6	0	-7.006869	1.316765	-1.560816
160	6	0	-7.389115	1.934973	-0.218542
161	6	0	-7.583638	2.113223	-2.729179
162	1	0	-7.439251	0.313189	-1.603475
163	6	0	-8.912682	2.066971	-0.147435

164	1	0	-6.920783	2.915995	-0.105222
165	1	0	-7.017927	1.309729	0.600849
166	6	0	-9.100312	2.236586	-2.541097
167	1	0	-7.131572	3.108640	-2.760895
168	1	0	-7.354142	1.607839	-3.675242
169	1	0	-9.205649	2.551953	0.789590
170	1	0	-9.367635	1.064669	-0.138160
171	1	0	-9.532145	2.844046	-3.343586
172	1	0	-9.559706	1.240358	-2.620845
173	6	0	7.004871	-1.315066	-1.561653
174	6	0	7.387568	-1.932740	-0.219268
175	6	0	7.581246	-2.112053	-2.729847
176	1	0	7.437275	-0.311522	-1.604869
177	6	0	8.911153	-2.064586	-0.148535
178	1	0	6.919365	-2.913752	-0.105401
179	1	0	7.016506	-1.307175	0.599916
180	6	0	9.097992	-2.235272	-2.542202
181	1	0	7.129200	-3.107498	-2.760933
182	1	0	7.351424	-1.607130	-3.676077
183	1	0	9.204422	-2.549163	0.788604
184	1	0	9.366041	-1.062246	-0.139813
185	1	0	9.529578	-2.843108	-3.344537
186	1	0	9.557316	-1.239064	-2.622603
187	6	0	4.209655	6.457543	-1.393850
188	6	0	2.409135	8.122588	-1.382447
189	6	0	4.867241	7.206315	-0.234818
190	1	0	4.640144	6.794472	-2.350929
191	1	0	4.368997	5.383218	-1.304655
192	6	0	3.099438	8.819376	-0.210091
193	1	0	2.727900	8.590676	-2.328251
194	1	0	1.323571	8.195684	-1.315660
195	1	0	5.954435	7.091011	-0.300191
196	1	0	4.551955	6.738176	0.705711
197	1	0	2.881469	9.892273	-0.239571
198	1	0	2.682886	8.427368	0.727156
199	6	0	-4.211801	-6.456025	-1.395463
200	6	0	-2.411108	-8.120880	-1.382847
201	6	0	-4.870757	-7.205675	-0.237758
202	1	0	-4.641078	-6.792285	-2.353328
203	1	0	-4.371322	-5.381771	-1.305716
204	6	0	-3.102753	-8.818454	-0.211766
205	1	0	-2.728672	-8.588460	-2.329313
206	1	0	-1.325619	-8.193914	-1.314787
207	1	0	-5.957884	-7.090514	-0.304510
208	1	0	-4.556717	-6.737973	0.703400
209	1	0	-2.884569	-9.891298	-0.241611
210	1	0	-2.687469	-8.426936	0.726245
211	7	0	2.779416	6.717704	-1.404964
212	7	0	-2.781513	-6.716015	-1.404989

213	7	0	4.540329	8.622435	-0.177853
214	1	0	4.959061	9.096417	-0.974457
215	7	0	-4.543720	-8.621775	-0.181246
216	1	0	-4.961323	-9.095313	-0.978710
217	7	0	-9.499947	2.800238	-1.259916
218	1	0	-9.189970	3.767482	-1.214429
219	7	0	9.498103	-2.798279	-1.260888
220	1	0	9.188299	-3.765547	-1.214848

Zero-point correction = 1.805240 (Hartree/Particle)
Thermal correction to Energy = 1.906346
Thermal correction to Enthalpy = 1.907290
Thermal correction to Gibbs Free Energy = 1.664143
Sum of electronic and zero-point Energies = -5771.837152
Sum of electronic and thermal Energies = -5771.736046
Sum of electronic and thermal Enthalpies = -5771.735102
Sum of electronic and thermal Free Energies = -5771.978248

Counterpoise corrected energy = -5773.882138230316
BSSE energy = 0.025211337377
sum of monomers = -5773.798884503853
complexation energy = -68.06 kcal/mole (raw)
complexation energy = -52.24 kcal/mole (corrected)

Table S5. Cartesian coordinates of [7]_TNB

Center Number	Atomic Number	Atomic Type	Coordinates (Angstroms) X	Y	Z
1	6	0	3.638686	1.141459	-0.765179
2	6	0	2.403111	1.817734	-0.761784
3	6	0	1.199701	1.092283	-0.869254
4	6	0	1.244411	-0.326438	-0.984075
5	6	0	2.496427	-0.978267	-0.972435
6	6	0	3.688113	-0.229712	-0.864526
7	6	0	-0.067548	1.755339	-0.834280
8	6	0	0.054298	-1.098217	-1.066643
9	6	0	-1.213269	-0.435079	-1.026484
10	6	0	-1.258106	0.982794	-0.910517
11	6	0	-2.417003	-1.168255	-1.047800
12	6	0	0.121601	-2.508840	-1.148031
13	6	0	1.392328	-3.128164	-1.119846
14	6	0	2.547254	-2.386543	-1.032322
15	1	0	1.500665	-4.207091	-1.140737
16	1	0	4.558223	1.707052	-0.672346
17	6	0	-0.136014	3.160931	-0.695823
18	6	0	-1.408781	3.772502	-0.634510
19	6	0	-2.563887	3.029975	-0.698040

20	6	0	-2.511369	1.627353	-0.835718
21	1	0	-1.520883	4.844882	-0.519468
22	6	0	-3.653545	-0.498568	-0.971937
23	1	0	-4.573450	-1.071378	-0.980387
24	6	0	-3.702708	0.872338	-0.869606
25	6	0	3.848070	-3.095533	-0.954163
26	6	0	5.000540	-0.912148	-0.825964
27	6	0	-5.017590	1.541845	-0.757930
28	6	0	-3.868968	3.726558	-0.598377
29	8	0	3.897058	-4.315357	-0.960383
30	8	0	6.040736	-0.282814	-0.752307
31	8	0	-3.928111	4.941568	-0.498125
32	8	0	-6.056984	0.906445	-0.782224
33	7	0	4.997350	-2.315807	-0.871608
34	7	0	-5.018017	2.938631	-0.619319
35	6	0	-0.937736	-0.281291	2.361870
36	6	0	0.425799	-0.040325	2.405833
37	6	0	1.271011	-1.137294	2.318562
38	6	0	0.800968	-2.434162	2.185905
39	6	0	-0.575506	-2.612830	2.153144
40	6	0	-1.469374	-1.557157	2.236582
41	1	0	0.816907	0.965637	2.492518
42	1	0	1.480517	-3.273002	2.102652
43	1	0	-2.539176	-1.719100	2.191882
44	7	0	-1.857462	0.862369	2.433827
45	8	0	-3.049780	0.618019	2.398120
46	8	0	-1.359755	1.970217	2.521273
47	7	0	2.721590	-0.909723	2.351431
48	8	0	3.440877	-1.884365	2.216661
49	8	0	3.099185	0.236598	2.507322
50	7	0	-1.103687	-3.974967	2.004866
51	8	0	-2.314808	-4.107551	2.014135
52	8	0	-0.291599	-4.874037	1.880764
53	6	0	2.348103	3.259520	-0.628643
54	6	0	1.090981	3.906814	-0.604984
55	6	0	-1.104742	-3.258940	-1.185728
56	6	0	-2.361606	-2.615195	-1.107081
57	7	0	3.500152	3.928872	-0.526007
58	6	0	3.433838	5.255431	-0.400095
59	7	0	2.281407	5.984769	-0.387152
60	6	0	1.162431	5.313290	-0.485340
61	1	0	0.253926	5.912965	-0.472808
62	7	0	-3.510182	-3.293724	-1.051229
63	6	0	-3.438511	-4.626460	-1.070828
64	7	0	-2.292313	-5.346839	-1.239632
65	6	0	-1.176321	-4.668279	-1.288948
66	1	0	-0.271678	-5.259438	-1.418362
67	6	0	-6.337215	3.590501	-0.482682
68	6	0	-6.509599	4.294543	0.862500

69	6	0	-6.664473	4.518734	-1.651238
70	1	0	-7.041536	2.754557	-0.514478
71	6	0	-7.922560	4.882687	0.940911
72	1	0	-5.770648	5.095328	0.961172
73	1	0	-6.343153	3.580549	1.678078
74	6	0	-8.069608	5.097554	-1.450878
75	1	0	-5.932273	5.330569	-1.697806
76	1	0	-6.610364	3.961013	-2.594118
77	1	0	-8.050056	5.434248	1.878467
78	1	0	-8.659108	4.066343	0.954449
79	1	0	-8.304319	5.805152	-2.253226
80	1	0	-8.811066	4.288416	-1.518999
81	6	0	6.310513	-2.984555	-0.762281
82	6	0	6.450712	-3.781255	0.534252
83	6	0	6.654885	-3.831740	-1.985854
84	1	0	7.020092	-2.153594	-0.721073
85	6	0	7.858359	-4.382800	0.599773
86	1	0	5.704525	-4.581365	0.560380
87	1	0	6.268126	-3.123966	1.392516
88	6	0	8.052883	-4.432102	-1.798084
89	1	0	5.920974	-4.634704	-2.101509
90	1	0	6.621626	-3.211075	-2.889586
91	1	0	7.965481	-4.998364	1.499278
92	1	0	8.597739	-3.573228	0.683210
93	1	0	8.299318	-5.084357	-2.642760
94	1	0	8.800617	-3.625888	-1.795910
95	6	0	5.885442	5.294022	-0.202785
96	6	0	4.633244	7.387709	-0.087681
97	6	0	6.560791	5.649327	1.124175
98	1	0	6.504760	5.647436	-1.041280
99	1	0	5.743618	4.218746	-0.310074
100	6	0	5.329265	7.707294	1.237101
101	1	0	5.199959	7.832119	-0.920408
102	1	0	3.615573	7.776593	-0.115961
103	1	0	7.570167	5.225572	1.151990
104	1	0	5.990908	5.188186	1.941405
105	1	0	5.436833	8.791569	1.348748
106	1	0	4.693089	7.355383	2.059873
107	6	0	-5.827638	-4.690360	-0.542064
108	6	0	-4.575123	-6.770636	-0.703043
109	6	0	-6.106192	-4.988702	0.935211
110	1	0	-6.632876	-5.100988	-1.166991
111	1	0	-5.750056	-3.620230	-0.732788
112	6	0	-4.868319	-7.044545	0.775873
113	1	0	-5.357208	-7.219002	-1.331788
114	1	0	-3.608581	-7.173041	-1.005351
115	1	0	-7.073305	-4.565860	1.227653
116	1	0	-5.334383	-4.493987	1.539031
117	1	0	-4.932586	-8.123642	0.952690

118	1	0	-4.028563	-6.662095	1.370553
119	7	0	4.590370	5.948204	-0.280436
120	7	0	-4.583764	-5.334119	-0.935321
121	7	0	6.634551	7.080312	1.378653
122	1	0	7.283847	7.504983	0.721297
123	7	0	-6.087679	-6.408763	1.253879
124	1	0	-6.891424	-6.858988	0.823440
125	7	0	-8.262967	5.760285	-0.169474
126	1	0	-7.669103	6.584846	-0.131813
127	7	0	8.216892	-5.183523	-0.562267
128	1	0	7.617331	-6.004247	-0.593903

Zero-point correction = 1.013717 (Hartree/Particle)
Thermal correction to Energy = 1.077028
Thermal correction to Enthalpy = 1.077972
Thermal correction to Gibbs Free Energy = 0.909193
Sum of electronic and zero-point Energies = -3730.780268
Sum of electronic and thermal Energies = -3730.716958
Sum of electronic and thermal Enthalpies = -3730.716014
Sum of electronic and thermal Free Energies = -3730.884793

Counterpoise corrected energy = -3731.793985700061
BSSE energy = 0.009785488185
sum of monomers = -3731.765022087233
complexation energy = -24.32 kcal/mole (raw)
complexation energy = -18.17 kcal/mole (corrected)

Table S6. Cartesian coordinates of [7]_CB7_ax

Center Number	Atomic Number	Atomic Type	Coordinates (Angstroms) X	Y	Z
1	6	0	-7.382038	2.451602	0.089556
2	6	0	-6.886446	1.132846	0.122952
3	6	0	-5.492952	0.910888	0.058041
4	6	0	-4.606200	2.016549	-0.051775
5	6	0	-5.123078	3.329827	-0.098016
6	6	0	-6.518704	3.518144	-0.019787
7	6	0	-5.000018	-0.422658	0.097054
8	6	0	-3.190666	1.788057	-0.111304
9	6	0	-2.699956	0.457378	-0.021791
10	6	0	-3.588814	-0.650289	0.066364
11	6	0	-1.305881	0.232206	-0.030594
12	6	0	-0.407071	1.313691	-0.157190
13	6	0	-0.886944	2.601231	-0.284464
14	6	0	-2.277569	2.855680	-0.256728
15	6	0	-2.805675	4.205740	-0.366827
16	6	0	-4.200317	4.424928	-0.244882

17	1	0	-6.963275	4.506542	-0.051123
18	1	0	-0.193044	3.425722	-0.394565
19	6	0	-7.778596	0.041589	0.217118
20	6	0	-7.295047	-1.246879	0.236241
21	6	0	-5.911516	-1.496950	0.175250
22	1	0	-7.984963	-2.080242	0.300650
23	6	0	-5.403827	-2.851064	0.177111
24	6	0	-4.010211	-3.067648	0.143143
25	6	0	-3.082531	-1.966173	0.123896
26	6	0	-0.823249	-1.088765	0.084313
27	6	0	-1.683716	-2.155054	0.160491
28	1	0	-1.250936	-3.141229	0.252578
29	6	0	1.059630	1.057582	-0.166450
30	6	0	0.628594	-1.357488	0.122329
31	6	0	-8.834984	2.711510	0.163723
32	6	0	-9.234547	0.269370	0.289994
33	7	0	-9.677864	1.604199	0.262698
34	7	0	1.485617	-0.256585	0.053144
35	8	0	-10.028772	-0.653755	0.371856
36	8	0	-9.282234	3.849614	0.140208
37	8	0	1.871278	1.949602	-0.351242
38	8	0	1.063525	-2.496835	0.210770
39	6	0	2.930983	-0.569025	0.090825
40	6	0	3.606213	-0.457014	-1.273166
41	6	0	3.722983	0.177804	1.159619
42	1	0	2.947129	-1.627433	0.365035
43	6	0	5.039654	-0.999110	-1.155130
44	1	0	3.607291	0.588755	-1.600196
45	1	0	3.041520	-1.042248	-2.009571
46	6	0	5.146807	-0.406272	1.184960
47	1	0	3.752621	1.247637	0.931533
48	1	0	3.239358	0.054769	2.137357
49	1	0	5.575755	-0.878084	-2.100406
50	1	0	4.990754	-2.079898	-0.958646
51	1	0	5.768206	0.127224	1.909092
52	1	0	5.096132	-1.451572	1.524170
53	6	0	-11.135351	1.818782	0.342149
54	6	0	-11.553375	2.575320	1.602739
55	6	0	-11.707126	2.467585	-0.918010
56	1	0	-11.542619	0.806083	0.410556
57	6	0	-13.079142	2.708807	1.628307
58	1	0	-11.093881	3.568263	1.607655
59	1	0	-11.200363	2.036446	2.490500
60	6	0	-13.225543	2.605871	-0.769677
61	1	0	-11.256626	3.454319	-1.061974
62	1	0	-11.460745	1.853755	-1.792994
63	1	0	-13.393838	3.291541	2.501020
64	1	0	-13.532731	1.712836	1.735935
65	1	0	-13.647028	3.113368	-1.644298

66	1	0	-13.683296	1.606570	-0.735614
67	7	0	5.821578	-0.390353	-0.097934
68	1	0	6.091887	0.555574	-0.343829
69	7	0	-13.643377	3.317098	0.430936
70	1	0	-13.316951	4.278490	0.369675
71	6	0	-4.594969	5.775882	-0.278083
72	6	0	-2.465659	6.449987	-0.636859
73	1	0	-5.640534	6.047543	-0.144098
74	7	0	-3.771961	6.780923	-0.459272
75	7	0	-1.958723	5.221002	-0.571560
76	6	0	-0.210558	7.259226	-1.150577
77	6	0	-2.022965	8.870553	-0.874071
78	6	0	0.619799	8.048311	-0.140300
79	1	0	0.025078	7.601768	-2.172892
80	1	0	-0.021478	6.188397	-1.074698
81	6	0	-1.160413	9.634509	0.133695
82	1	0	-1.885064	9.304314	-1.878350
83	1	0	-3.082843	8.922602	-0.623360
84	1	0	1.687720	7.954093	-0.371539
85	1	0	0.464169	7.604273	0.850156
86	1	0	-1.401545	10.702999	0.100173
87	1	0	-1.400787	9.275071	1.142839
88	7	0	-1.617531	7.480799	-0.902270
89	7	0	0.273501	9.463817	-0.070485
90	1	0	0.528261	9.905451	-0.951571
91	6	0	-3.624346	-4.426008	0.091289
92	6	0	-5.795783	-5.097993	0.168732
93	1	0	-2.573625	-4.692109	0.033875
94	7	0	-6.285063	-3.861519	0.198408
95	7	0	-4.477380	-5.425616	0.101284
96	6	0	-6.267966	-7.523147	0.166246
97	6	0	-8.113240	-5.926317	0.246820
98	6	0	-6.975211	-8.248613	-0.979837
99	1	0	-6.543090	-7.996755	1.123637
100	1	0	-5.182898	-7.561000	0.064794
101	6	0	-8.785994	-6.676317	-0.903105
102	1	0	-8.495358	-6.306900	1.208477
103	1	0	-8.311780	-4.855324	0.202210
104	1	0	-6.735316	-9.317566	-0.950395
105	1	0	-6.595690	-7.853245	-1.931293
106	1	0	-9.874878	-6.590392	-0.819648
107	1	0	-8.491826	-6.204361	-1.849606
108	7	0	-6.679481	-6.134238	0.193840
109	7	0	-8.422866	-8.085743	-0.974308
110	1	0	-8.805172	-8.564826	-0.162122
111	6	0	4.274636	4.011411	-3.312458
112	6	0	3.797407	2.958346	-4.345662
113	7	0	2.490445	2.596652	-3.840223
114	6	0	1.998544	3.578833	-2.989961

115	7	0	3.064809	4.400669	-2.665722
116	7	0	5.232436	3.266667	-2.530016
117	6	0	5.645631	2.118705	-3.186084
118	7	0	4.816457	1.961190	-4.281925
119	8	0	0.847048	3.739808	-2.660833
120	8	0	6.587447	1.418983	-2.891481
121	6	0	1.558522	1.787146	-4.578879
122	6	0	5.015451	0.928057	-5.250286
123	7	0	1.849040	0.382247	-4.591833
124	7	0	4.159168	-0.213885	-5.105356
125	6	0	2.774452	-0.233777	-5.495340
126	6	0	2.405485	-1.745902	-5.419422
127	7	0	1.449787	-1.803002	-4.339796
128	6	0	1.053291	-0.535452	-3.930100
129	6	0	4.707262	-1.480924	-5.026663
130	7	0	3.670689	-2.378065	-5.199756
131	6	0	0.569924	-2.927100	-4.147219
132	6	0	3.896747	-3.791917	-5.163561
133	8	0	5.878792	-1.751674	-4.895549
134	8	0	0.143037	-0.280937	-3.174325
135	1	0	3.718556	3.354499	-5.370777
136	6	0	2.935710	5.488884	-1.744097
137	6	0	6.078927	3.870793	-1.542175
138	1	0	1.499775	2.136776	-5.623366
139	1	0	0.586224	1.922433	-4.095756
140	1	0	6.038604	0.557834	-5.135647
141	1	0	4.888857	1.366529	-6.252429
142	1	0	2.655795	0.193082	-6.504247
143	1	0	1.946730	-2.128878	-6.344731
144	7	0	1.108805	-3.975583	-3.327257
145	1	0	0.288306	-3.327352	-5.136208
146	1	0	-0.326404	-2.563812	-3.634800
147	1	0	4.982133	-3.923898	-5.195877
148	7	0	3.416228	-4.466614	-3.995274
149	6	0	4.222902	-4.688764	-2.890761
150	6	0	2.086180	-4.938056	-3.794692
151	6	0	2.227256	-5.868528	-2.565117
152	1	0	1.721955	-5.452675	-4.697435
153	6	0	0.353601	-4.503090	-2.290976
154	8	0	-0.719999	-4.103866	-1.900464
155	7	0	1.018715	-5.630522	-1.840951
156	1	0	3.435999	-4.259205	-6.044261
157	8	0	5.384067	-4.374475	-2.778942
158	7	0	3.459108	-5.396295	-1.976176
159	6	0	4.091922	-6.017122	-0.849720
160	6	0	0.561632	-6.395623	-0.716619
161	1	0	2.306901	-6.935150	-2.827321
162	7	0	3.529849	-5.654738	0.419015
163	1	0	-0.518897	-6.248798	-0.640322

164	7	0	1.110633	-6.031426	0.557904
165	1	0	0.775928	-7.454469	-0.910985
166	6	0	0.396003	-5.278341	1.464772
167	6	0	2.428129	-6.355660	1.022740
168	1	0	4.056627	-7.114401	-0.958341
169	1	0	2.589908	-7.443187	0.956202
170	6	0	2.430911	-5.820363	2.486809
171	6	0	4.307562	-4.988997	1.353590
172	1	0	5.134045	-5.680974	-0.854257
173	8	0	5.381507	-4.470720	1.158401
174	7	0	3.659791	-5.095948	2.571323
175	8	0	-0.770394	-4.958808	1.367548
176	7	0	1.215576	-5.047250	2.547379
177	6	0	0.685621	-4.459459	3.744734
178	6	0	4.273506	-4.656247	3.787297
179	1	0	2.410115	-6.619644	3.244600
180	1	0	1.877226	5.751163	-1.720682
181	1	0	3.519579	6.339594	-2.119654
182	7	0	3.324795	5.224317	-0.388671
183	7	0	5.466374	4.041081	-0.256364
184	1	0	6.430339	4.850464	-1.907223
185	1	0	6.935257	3.202167	-1.407335
186	6	0	4.669559	5.187862	0.100279
187	6	0	5.989692	3.400051	0.852787
188	6	0	2.396343	4.907741	0.583959
189	1	0	4.758342	4.887953	-3.772608
190	8	0	6.820527	2.519868	0.848483
191	7	0	5.438852	4.000280	1.968516
192	8	0	1.193598	4.938085	0.464375
193	7	0	3.093324	4.655201	1.749457
194	6	0	4.484932	5.013301	1.637523
195	6	0	2.379153	4.549904	2.990646
196	6	0	5.799972	3.604902	3.296919
197	1	0	4.692891	5.930622	2.209888
198	1	0	5.195936	6.115374	-0.173963
199	1	0	1.317785	4.624083	2.735099
200	1	0	2.660299	5.378271	3.658241
201	7	0	2.566861	3.320047	3.698581
202	1	0	6.774598	3.110809	3.239448
203	7	0	4.911815	2.661963	3.919965
204	1	0	5.879389	4.511676	3.915672
205	6	0	3.670055	3.031388	4.557329
206	6	0	1.672279	2.270459	3.602330
207	1	0	3.833572	3.865108	5.258221
208	6	0	3.238902	1.711614	5.256726
209	6	0	5.418363	1.476123	4.427252
210	8	0	0.650305	2.255544	2.956252
211	7	0	2.132165	1.264938	4.442777
212	8	0	6.532693	1.037258	4.259208

213	7	0	4.429304	0.920316	5.217802
214	6	0	1.228911	0.227107	4.854256
215	1	0	2.911864	1.855676	6.298618
216	6	0	4.632496	-0.309795	5.925710
217	1	0	0.307615	0.356838	4.277562
218	1	0	1.008112	0.341480	5.930175
219	7	0	1.695885	-1.108247	4.602552
220	1	0	5.708629	-0.505563	5.907347
221	7	0	3.993800	-1.465855	5.369677
222	1	0	4.290977	-0.182743	6.962191
223	6	0	2.607781	-1.788982	5.494485
224	6	0	0.862347	-2.005199	3.943965
225	1	0	2.281107	-1.669039	6.539685
226	6	0	2.539892	-3.254747	4.989517
227	6	0	4.696392	-2.408059	4.637453
228	8	0	5.881906	-2.394603	4.403012
229	7	0	3.792185	-3.398689	4.287129
230	8	0	-0.137493	-1.732636	3.320534
231	7	0	1.353137	-3.273679	4.194229
232	1	0	2.463942	-3.996103	5.800781
233	1	0	0.705983	-5.193693	4.564574
234	1	0	-0.350082	-4.191415	3.516560
235	1	0	4.136367	-5.440943	4.548485
236	1	0	5.340789	-4.517368	3.589261

Zero-point correction = 1.890130 (Hartree/Particle)
Thermal correction to Energy = 2.000551
Thermal correction to Enthalpy = 2.001495
Thermal correction to Gibbs Free Energy = 1.740096
Sum of electronic and zero-point Energies = -7093.076646
Sum of electronic and thermal Energies = -7092.966226
Sum of electronic and thermal Enthalpies = -7092.965282
Sum of electronic and thermal Free Energies = -7093.226680

Counterpoise corrected energy = -7094.966776433428
BSSE energy = 0.027966465505
sum of monomers = -7094.895716428531
complexation energy = -62.14 kcal/mole (raw)
complexation energy = -44.59 kcal/mole (corrected)

Table S7. Cartesian coordinates of [7]_CB7_ec

Center Number	Atomic Number	Atomic Type	Coordinates (Angstroms) X	Y	Z
1	6	0	4.251491	-3.021797	-0.070109
2	6	0	5.237488	-2.012757	0.016070
3	6	0	4.850682	-0.655534	-0.038154

4	6	0	3.476428	-0.323391	-0.180815
5	6	0	2.503481	-1.344359	-0.275119
6	6	0	2.921901	-2.696302	-0.208777
7	6	0	5.847897	0.355094	0.046440
8	6	0	3.084717	1.051049	-0.240355
9	6	0	4.085742	2.062997	-0.173565
10	6	0	5.460640	1.732494	-0.022390
11	6	0	3.703032	3.418508	-0.271983
12	6	0	2.343403	3.761852	-0.458077
13	6	0	1.375105	2.780077	-0.480455
14	6	0	1.728011	1.419233	-0.366859
15	6	0	0.717258	0.378137	-0.420443
16	6	0	1.127176	-0.973341	-0.422677
17	1	0	2.205326	-3.509930	-0.249271
18	1	0	0.335454	3.048480	-0.606074
19	6	0	6.598828	-2.354860	0.158297
20	6	0	7.560544	-1.372015	0.242213
21	6	0	7.202721	-0.013616	0.186248
22	1	0	8.602800	-1.647432	0.351401
23	6	0	8.213079	1.021254	0.266259
24	6	0	7.824560	2.377632	0.201147
25	6	0	6.438984	2.751642	0.052690
26	6	0	4.692148	4.421102	-0.189150
27	6	0	6.020003	4.094411	-0.026022
28	1	0	6.723397	4.917390	0.035233
29	6	0	1.968847	5.174092	-0.670256
30	6	0	4.321651	5.848409	-0.268759
31	6	0	4.638089	-4.448100	-0.007814
32	6	0	7.012852	-3.771248	0.222484
33	7	0	5.999593	-4.738194	0.136804
34	7	0	2.972493	6.140323	-0.489713
35	8	0	8.183995	-4.093169	0.346281
36	8	0	3.811471	-5.343885	-0.079401
37	8	0	0.845054	5.520316	-0.996033
38	8	0	5.155415	6.735570	-0.157491
39	6	0	2.617373	7.566064	-0.604324
40	6	0	2.205505	7.961310	-2.022683
41	6	0	1.577085	8.002989	0.427089
42	1	0	3.552129	8.085632	-0.374487
43	6	0	1.895114	9.460073	-2.064393
44	1	0	1.322295	7.387612	-2.319311
45	1	0	3.014698	7.717433	-2.721557
46	6	0	1.302635	9.500719	0.264867
47	1	0	0.651907	7.437263	0.282611
48	1	0	1.945923	7.785882	1.436881
49	1	0	1.548267	9.749212	-3.062755
50	1	0	2.814669	10.032114	-1.875047
51	1	0	0.525538	9.823166	0.967233
52	1	0	2.207964	10.071151	0.517256

53	6	0	6.424892	-6.148068	0.209378
54	6	0	5.876589	-6.867067	1.441864
55	6	0	6.127975	-6.926214	-1.072333
56	1	0	7.511631	-6.085811	0.316215
57	6	0	6.405817	-8.304522	1.466614
58	1	0	4.782876	-6.874047	1.407988
59	1	0	6.181905	-6.329798	2.348101
60	6	0	6.647642	-8.359188	-0.924321
61	1	0	5.049447	-6.938509	-1.255739
62	1	0	6.607993	-6.429726	-1.924441
63	1	0	5.983194	-8.849632	2.317995
64	1	0	7.495652	-8.292140	1.612319
65	1	0	6.403364	-8.945526	-1.817060
66	1	0	7.744485	-8.346412	-0.848132
67	7	0	0.906292	9.889962	-1.082807
68	1	0	0.020922	9.438407	-1.300505
69	7	0	6.138361	-9.058236	0.248692
70	1	0	5.131558	-9.164557	0.149004
71	6	0	0.089560	-1.912525	-0.602075
72	6	0	-1.466108	-0.264892	-0.579541
73	1	0	0.315374	-2.967030	-0.686719
74	7	0	-1.175065	-1.589351	-0.693154
75	7	0	-0.573120	0.720138	-0.475966
76	6	0	-3.187793	1.345440	-0.027479
77	6	0	-3.805962	-0.965161	-0.394691
78	6	0	-3.412960	1.151719	1.473500
79	1	0	-4.110969	1.669525	-0.521953
80	1	0	-2.402835	2.075214	-0.222629
81	6	0	-4.072935	-1.140914	1.105286
82	1	0	-4.717464	-0.632059	-0.907023
83	1	0	-3.467629	-1.896634	-0.849279
84	1	0	-3.734987	2.085494	1.941813
85	1	0	-2.444156	0.885094	1.917171
86	1	0	-4.896523	-1.844514	1.265668
87	1	0	-3.170321	-1.577930	1.556239
88	7	0	-2.789539	0.064262	-0.593044
89	7	0	-4.370755	0.106400	1.797553
90	1	0	-5.310062	0.415525	1.560492
91	6	0	8.892398	3.294262	0.288807
92	6	0	10.410251	1.614315	0.462316
93	1	0	8.703398	4.365825	0.253046
94	7	0	9.493132	0.650652	0.396342
95	7	0	10.152016	2.949553	0.415550
96	6	0	12.799249	2.211996	0.642576
97	6	0	12.129901	-0.137068	0.644483
98	6	0	13.824336	1.895215	-0.448017
99	1	0	13.281607	2.143876	1.631328
100	1	0	12.385086	3.214364	0.532210
101	6	0	13.165371	-0.412150	-0.446750

102	1	0	12.575509	-0.331540	1.633448
103	1	0	11.249708	-0.770994	0.536287
104	1	0	14.686474	2.564808	-0.354629
105	1	0	13.365360	2.086998	-1.426788
106	1	0	13.543415	-1.435839	-0.352583
107	1	0	12.673334	-0.333726	-1.424938
108	7	0	11.713698	1.251316	0.585843
109	7	0	14.288703	0.515294	-0.434589
110	1	0	14.838335	0.358536	0.406974
111	6	0	-3.028103	-5.707368	-2.837187
112	6	0	-2.988479	-4.801045	-4.101448
113	7	0	-1.845429	-3.957461	-3.841580
114	6	0	-1.034569	-4.481067	-2.853024
115	7	0	-1.736821	-5.509446	-2.250879
116	7	0	-4.162688	-5.186036	-2.115376
117	6	0	-4.958578	-4.376260	-2.911045
118	7	0	-4.272674	-4.174613	-4.094607
119	8	0	0.103191	-4.151055	-2.600479
120	8	0	-6.066156	-3.970937	-2.646678
121	6	0	-1.330811	-2.990262	-4.769723
122	6	0	-4.842207	-3.416917	-5.171668
123	7	0	-2.066510	-1.757855	-4.819365
124	7	0	-4.463524	-2.037072	-5.225709
125	6	0	-3.197696	-1.548744	-5.684702
126	6	0	-3.387606	-0.001451	-5.666110
127	7	0	-2.264904	0.460485	-4.907381
128	6	0	-1.464956	-0.565989	-4.439279
129	6	0	-5.350737	-1.030815	-4.889681
130	7	0	-4.684222	0.169585	-5.065377
131	6	0	-1.864896	1.833326	-4.816416
132	6	0	-5.413186	1.406837	-5.075205
133	8	0	-6.507042	-1.172226	-4.565619
134	8	0	-0.408136	-0.448610	-3.863741
135	1	0	-2.845634	-5.361648	-5.038353
136	6	0	-1.200258	-6.243936	-1.139876
137	6	0	-4.736742	-5.834277	-0.972220
138	1	0	-1.296270	-3.427000	-5.781331
139	1	0	-0.315305	-2.753238	-4.437042
140	1	0	-5.927942	-3.430006	-5.037986
141	1	0	-4.574644	-3.906947	-6.117771
142	1	0	-2.980717	-1.948644	-6.688070
143	1	0	-3.375939	0.450377	-6.671026
144	7	0	-2.606753	2.624153	-3.873447
145	1	0	-1.936343	2.288474	-5.817397
146	1	0	-0.824101	1.845385	-4.477973
147	1	0	-6.461044	1.150967	-4.890599
148	7	0	-5.027524	2.353330	-4.072102
149	6	0	-5.794941	2.573780	-2.945096
150	6	0	-3.888667	3.215625	-4.153665

151	6	0	-4.092944	4.191644	-2.955702
152	1	0	-3.869663	3.723111	-5.131618
153	6	0	-1.926570	3.307323	-2.877358
154	8	0	-0.755929	3.185008	-2.608010
155	7	0	-2.819149	4.192950	-2.302143
156	1	0	-5.312150	1.883712	-6.062246
157	8	0	-6.831347	2.017532	-2.662525
158	7	0	-5.187322	3.596146	-2.230864
159	6	0	-5.934326	4.304545	-1.229062
160	6	0	-2.412659	5.138575	-1.299845
161	1	0	-4.359706	5.214354	-3.267789
162	7	0	-5.320768	4.352913	0.064938
163	1	0	-1.319852	5.084456	-1.221043
164	7	0	-2.948314	4.909806	0.016840
165	1	0	-2.705557	6.147636	-1.627874
166	6	0	-2.123523	4.588583	1.079490
167	6	0	-4.275720	5.269530	0.433162
168	1	0	-6.120845	5.335329	-1.573311
169	1	0	-4.522019	6.284896	0.085886
170	6	0	-4.220666	5.144047	1.991492
171	6	0	-5.910131	3.737100	1.153303
172	1	0	-6.885306	3.775128	-1.113939
173	8	0	-6.839647	2.961828	1.124680
174	7	0	-5.276214	4.214735	2.283136
175	8	0	-0.948787	4.316124	1.028278
176	7	0	-2.882101	4.688259	2.233717
177	6	0	-2.254475	4.601781	3.523455
178	6	0	-5.668228	3.793047	3.596772
179	1	0	-4.400786	6.095536	2.514481
180	1	0	-0.117895	-6.084200	-1.148016
181	1	0	-1.419025	-7.310259	-1.282610
182	7	0	-1.665938	-5.848536	0.161347
183	7	0	-4.088974	-5.526344	0.269055
184	1	0	-4.729849	-6.926450	-1.126504
185	1	0	-5.769497	-5.479824	-0.891444
186	6	0	-2.919934	-6.221318	0.743874
187	6	0	-4.809454	-4.945548	1.300099
188	6	0	-0.907513	-5.054162	0.998734
189	1	0	-3.178616	-6.773545	-3.068366
190	8	0	-5.912791	-4.456093	1.221206
191	7	0	-4.057587	-5.089797	2.450173
192	8	0	0.216755	-4.656707	0.782903
193	7	0	-1.639652	-4.854807	2.155310
194	6	0	-2.796374	-5.718995	2.212062
195	6	0	-0.990457	-4.322696	3.324789
196	6	0	-4.556966	-4.684525	3.730840
197	1	0	-2.632134	-6.528696	2.940264
198	1	0	-3.063347	-7.310296	0.663649
199	1	0	0.002048	-3.992739	3.003174

200	1	0	-0.888124	-5.116268	4.082622
201	7	0	-1.643213	-3.198991	3.924839
202	1	0	-5.639763	-4.560720	3.633376
203	7	0	-4.049443	-3.435690	4.218649
204	1	0	-4.336255	-5.484247	4.454969
205	6	0	-2.753950	-3.295937	4.824185
206	6	0	-1.094580	-1.928510	3.881721
207	1	0	-2.581836	-4.106888	5.550395
208	6	0	-2.817795	-1.884071	5.479185
209	6	0	-4.921365	-2.421824	4.586408
210	8	0	-0.112936	-1.595090	3.257742
211	7	0	-1.840506	-1.132954	4.735969
212	8	0	-6.110941	-2.388500	4.372846
213	7	0	-4.187728	-1.504274	5.312917
214	6	0	-1.332325	0.133270	5.180268
215	1	0	-2.553776	-1.886263	6.549077
216	6	0	-4.810202	-0.372202	5.934910
217	1	0	-0.367718	0.286475	4.685891
218	1	0	-1.186802	0.096620	6.273324
219	7	0	-2.148179	1.263918	4.843349
220	1	0	-5.889472	-0.523367	5.838929
221	7	0	-4.517894	0.906135	5.358165
222	1	0	-4.528011	-0.348314	6.997428
223	6	0	-3.324565	1.660930	5.580499
224	6	0	-1.595465	2.316768	4.135815
225	1	0	-3.100312	1.706043	6.657941
226	6	0	-3.663576	3.048015	4.961997
227	6	0	-5.415400	1.548408	4.526515
228	8	0	-6.520097	1.163550	4.224422
229	7	0	-4.846171	2.769656	4.182871
230	8	0	-0.523093	2.326343	3.576923
231	7	0	-2.474086	3.381539	4.241686
232	1	0	-3.878308	3.821113	5.716118
233	1	0	-2.591720	5.441696	4.145563
234	1	0	-1.176866	4.672462	3.348833
235	1	0	-5.690136	4.674116	4.255641
236	1	0	-6.673875	3.370217	3.507827

Zero-point correction = 1.890507 (Hartree/Particle)
Thermal correction to Energy = 2.001145
Thermal correction to Enthalpy = 2.002089
Thermal correction to Gibbs Free Energy = 1.736904
Sum of electronic and zero-point Energies = -7093.069136
Sum of electronic and thermal Energies = -7092.958498
Sum of electronic and thermal Enthalpies = -7092.957554
Sum of electronic and thermal Free Energies = -7093.222739

Counterpoise corrected energy = -7094.959643414140
BSSE energy = 0.020515404352
sum of monomers = -7094.896362419682
complexation energy = -52.58 kcal/mole (raw)
complexation energy = -39.71 kcal/mole (corrected)

Table S8. Cartesian coordinates of [7]_2cucur_axial

Center Number	Atomic Number	Atomic Type	Coordinates (Angstroms) X	Y	Z
1	6	0	3.521523	-1.132640	-0.237611
2	6	0	2.846245	0.100508	-0.125406
3	6	0	1.435386	0.127427	-0.157814
4	6	0	0.710858	-1.084144	-0.324069
5	6	0	1.404655	-2.300156	-0.483332
6	6	0	2.815949	-2.300312	-0.404602
7	6	0	0.761210	1.371630	-0.039141
8	6	0	-0.720799	-1.065510	-0.330326
9	6	0	-1.394240	0.172068	-0.144960
10	6	0	-0.669211	1.389479	-0.017451
11	6	0	-2.805057	0.192499	-0.101787
12	6	0	-3.545533	-0.999866	-0.259221
13	6	0	-2.884774	-2.194856	-0.467417
14	6	0	-1.473452	-2.248226	-0.509004
15	6	0	-0.764155	-3.495141	-0.746851
16	6	0	0.648459	-3.497461	-0.728856
17	1	0	3.373009	-3.224039	-0.471611
18	1	0	-3.451696	-3.108028	-0.596258
19	6	0	3.576955	1.298371	0.013743
20	6	0	2.918565	2.508302	0.093278
21	6	0	1.510999	2.564054	0.055864
22	1	0	3.484782	3.426477	0.188382
23	6	0	0.807077	3.831638	0.100045
24	6	0	-0.601704	3.834890	0.172032
25	6	0	-1.356776	2.611059	0.135099
26	6	0	-3.468849	1.419901	0.094189
27	6	0	-2.764128	2.593402	0.220400
28	1	0	-3.323879	3.504707	0.378463
29	6	0	-5.028977	-0.955779	-0.204876
30	6	0	-4.937170	1.466842	0.173686
31	6	0	4.997622	-1.186604	-0.176321
32	6	0	5.050917	1.252021	0.087016
33	7	0	5.672898	-0.000110	0.080903
34	7	0	-5.631296	0.259888	0.118753
35	8	0	5.723708	2.272204	0.166081
36	8	0	5.608496	-2.238159	-0.336548
37	8	0	-5.714720	-1.947493	-0.421699

38	8	0	-5.537968	2.529357	0.293209
39	6	0	-7.105449	0.371379	0.236139
40	6	0	-7.810751	0.329659	-1.113372
41	6	0	-7.757131	-0.596069	1.213341
42	1	0	-7.249246	1.374254	0.647106
43	6	0	-9.292869	0.645481	-0.913918
44	1	0	-7.701817	-0.666690	-1.551198
45	1	0	-7.363036	1.057156	-1.797202
46	6	0	-9.235132	-0.214031	1.334612
47	1	0	-7.675655	-1.626132	0.857315
48	1	0	-7.268661	-0.538056	2.190994
49	1	0	-9.818349	0.570541	-1.870560
50	1	0	-9.392790	1.685003	-0.575093
51	1	0	-9.737872	-0.897497	2.022940
52	1	0	-9.315473	0.791264	1.767892
53	6	0	7.150343	-0.003035	0.209108
54	6	0	7.678280	-0.935714	1.293082
55	6	0	7.858107	-0.249606	-1.118396
56	1	0	7.387309	1.017079	0.522593
57	6	0	9.194405	-0.752833	1.393333
58	1	0	7.454171	-1.978769	1.053065
59	1	0	7.201402	-0.701920	2.250331
60	6	0	9.367037	-0.115252	-0.918293
61	1	0	7.625712	-1.255197	-1.479487
62	1	0	7.514186	0.470255	-1.867314
63	1	0	9.597470	-1.432820	2.148228
64	1	0	9.416643	0.267060	1.730929
65	1	0	9.882613	-0.334605	-1.857931
66	1	0	9.603404	0.924627	-0.659620
67	7	0	-9.961757	-0.209732	0.064742
68	1	0	-9.949263	-1.163085	-0.296208
69	7	0	9.909825	-0.969327	0.135746
70	1	0	9.757719	-1.940081	-0.136254
71	6	0	1.249452	-4.741553	-1.013723
72	6	0	-0.784121	-5.737227	-1.229021
73	1	0	2.329799	-4.832131	-1.054984
74	7	0	0.575066	-5.839314	-1.257427
75	7	0	-1.466408	-4.613163	-0.986671
76	6	0	-2.913785	-6.929403	-1.335729
77	6	0	-0.853890	-8.185889	-1.437437
78	6	0	-3.233263	-7.569936	0.011757
79	1	0	-3.337999	-7.539796	-2.143217
80	1	0	-3.320824	-5.923627	-1.400478
81	6	0	-1.186089	-8.816181	-0.087888
82	1	0	-1.261707	-8.806709	-2.244864
83	1	0	0.221667	-8.098297	-1.580230
84	1	0	-4.317867	-7.659189	0.126359
85	1	0	-2.873856	-6.908475	0.809262
86	1	0	-0.760884	-9.823674	-0.042828

87	1	0	-0.710369	-8.221300	0.702248
88	7	0	-1.474100	-6.868452	-1.535592
89	7	0	-2.620390	-8.886676	0.190889
90	1	0	-3.028349	-9.502290	-0.512114
91	6	0	-1.190753	5.114356	0.255161
92	6	0	0.842890	6.120136	0.081571
93	1	0	-2.267464	5.219618	0.354163
94	7	0	1.518016	4.968017	0.064936
95	7	0	-0.508502	6.235307	0.219847
96	6	0	0.964930	8.567167	0.283656
97	6	0	2.989952	7.258475	-0.139513
98	6	0	1.537550	9.674607	-0.587815
99	1	0	1.198032	8.778083	1.339549
100	1	0	-0.118208	8.520445	0.181416
101	6	0	3.508670	8.409348	-0.990243
102	1	0	3.403008	7.330925	0.878440
103	1	0	3.306583	6.303492	-0.556790
104	1	0	1.145672	10.633337	-0.235526
105	1	0	1.190420	9.537352	-1.619098
106	1	0	4.601811	8.414529	-0.938743
107	1	0	3.232405	8.232416	-2.036840
108	7	0	1.536670	7.276281	-0.081239
109	7	0	2.997860	9.722336	-0.603767
110	1	0	3.302443	9.892844	0.354612
111	6	0	8.084628	4.943804	2.458228
112	6	0	8.259477	5.383384	0.985896
113	7	0	6.908108	5.450200	0.498913
114	6	0	6.008707	4.933463	1.394726
115	7	0	6.703256	4.503289	2.498154
116	7	0	9.072831	3.913400	2.610860
117	6	0	9.627717	3.540513	1.416796
118	7	0	9.084838	4.327354	0.427160
119	8	0	4.790064	4.915181	1.266250
120	8	0	10.491095	2.681897	1.264182
121	6	0	6.531698	5.941460	-0.800352
122	6	0	9.769429	4.495473	-0.828387
123	7	0	6.757969	5.047473	-1.906323
124	7	0	9.001003	4.060845	-1.963764
125	6	0	8.008584	4.921976	-2.593257
126	6	0	7.639568	4.108024	-3.840429
127	7	0	6.424196	3.430937	-3.396682
128	6	0	5.828916	4.154651	-2.385005
129	6	0	9.543054	3.194736	-2.885456
130	7	0	8.790256	3.281271	-4.029885
131	6	0	5.641803	2.591035	-4.271439
132	6	0	9.058258	2.520481	-5.218130
133	8	0	10.538204	2.496338	-2.730645
134	8	0	4.667848	4.053751	-2.008885
135	1	0	8.755994	6.351952	0.867252

136	6	0	6.013049	4.203208	3.727124
137	6	0	9.454189	3.332839	3.865930
138	1	0	7.087478	6.862487	-0.997534
139	1	0	5.468407	6.169620	-0.765883
140	1	0	10.681597	3.897799	-0.800066
141	1	0	10.034662	5.553838	-0.948626
142	1	0	8.433625	5.908023	-2.807452
143	1	0	7.442521	4.709737	-4.733392
144	7	0	6.169875	1.258488	-4.447391
145	1	0	5.559716	3.059561	-5.260334
146	1	0	4.646694	2.508379	-3.830795
147	1	0	10.138317	2.399985	-5.312020
148	7	0	8.479193	1.209398	-5.255945
149	6	0	9.199846	0.080915	-4.970494
150	6	0	7.090846	0.934298	-5.519154
151	6	0	7.047393	-0.612948	-5.570830
152	1	0	6.775434	1.421896	-6.447103
153	6	0	5.490722	0.143858	-4.001916
154	8	0	4.581199	0.139003	-3.179887
155	7	0	5.976115	-0.952816	-4.672825
156	1	0	8.688107	3.095655	-6.071016
157	8	0	10.393874	0.037638	-4.689608
158	7	0	8.365375	-0.995626	-5.113076
159	6	0	8.881451	-2.329819	-5.243594
160	6	0	5.348971	-2.248354	-4.655213
161	1	0	6.855393	-1.020735	-6.568513
162	7	0	8.434454	-3.249811	-4.238869
163	1	0	4.340336	-2.132439	-4.255583
164	7	0	6.013535	-3.241276	-3.839753
165	1	0	5.287145	-2.604446	-5.690700
166	6	0	5.277631	-4.032962	-2.984000
167	6	0	7.197613	-3.966157	-4.293017
168	1	0	8.594770	-2.737541	-6.218692
169	1	0	7.033636	-4.382255	-5.292305
170	6	0	7.369282	-5.022001	-3.188288
171	6	0	9.098906	-3.465911	-3.059254
172	1	0	9.968874	-2.265168	-5.188173
173	8	0	10.143936	-2.924134	-2.714705
174	7	0	8.410007	-4.429784	-2.358387
175	8	0	4.109751	-3.852794	-2.661997
176	7	0	6.062866	-5.093549	-2.603904
177	6	0	5.650772	-6.110274	-1.671860
178	6	0	9.063217	-5.176572	-1.315515
179	1	0	7.683523	-6.005321	-3.553032
180	1	0	4.945630	4.315295	3.532824
181	1	0	6.313745	4.917019	4.501399
182	7	0	6.240393	2.882659	4.243104
183	7	0	8.602401	2.258416	4.315773
184	1	0	9.463023	4.130633	4.617697

185	1	0	10.461656	2.928293	3.759796
186	6	0	7.369558	2.518845	5.045736
187	6	0	9.149724	1.051791	4.694054
188	6	0	5.470723	1.792471	3.921544
189	1	0	8.250601	5.744061	3.186779
190	8	0	10.268417	0.641884	4.406777
191	7	0	8.235253	0.406539	5.487394
192	8	0	4.485285	1.799502	3.193051
193	7	0	5.975576	0.703245	4.601593
194	6	0	6.993492	1.118055	5.558227
195	6	0	5.154596	-0.459267	4.832062
196	6	0	8.481690	-0.863306	6.112824
197	1	0	6.593887	1.102181	6.577382
198	1	0	7.538623	3.257207	5.835819
199	1	0	4.249757	-0.344122	4.232712
200	1	0	4.881523	-0.502140	5.895240
201	7	0	5.770209	-1.709269	4.459701
202	1	0	9.560087	-0.972153	6.233794
203	7	0	8.007009	-2.018355	5.403694
204	1	0	8.010714	-0.849623	7.099429
205	6	0	6.636897	-2.446557	5.367969
206	6	0	5.135800	-2.559012	3.579233
207	1	0	6.210222	-2.460494	6.375933
208	6	0	6.727267	-3.835578	4.699478
209	6	0	8.811210	-2.784326	4.599798
210	8	0	4.248154	-2.253859	2.791142
211	7	0	5.631155	-3.822605	3.776640
212	8	0	10.009493	-2.608068	4.404235
213	7	0	8.050180	-3.811937	4.098111
214	6	0	5.181648	-4.971344	3.043743
215	1	0	6.638114	-4.675395	5.395562
216	6	0	8.667590	-4.981647	3.526132
217	1	0	4.131783	-4.823728	2.787219
218	1	0	5.269815	-5.842146	3.702082
219	7	0	5.889019	-5.219491	1.812015
220	1	0	9.747416	-4.833204	3.570436
221	7	0	8.310319	-5.254061	2.160734
222	1	0	8.401630	-5.861662	4.121155
223	6	0	7.134129	-5.968610	1.758724
224	6	0	5.199306	-5.336777	0.630672
225	1	0	7.040565	-6.904995	2.317746
226	6	0	7.339605	-6.154279	0.241587
227	6	0	8.982103	-4.728766	1.087692
228	8	0	9.989564	-4.032163	1.144665
229	7	0	8.355750	-5.159812	-0.061128
230	8	0	4.021797	-5.041918	0.458404
231	7	0	6.031845	-5.901601	-0.298529
232	1	0	7.685806	-7.153030	-0.042959
233	1	0	6.075110	-7.062184	-2.002259

234	1	0	4.562706	-6.176512	-1.705053
235	1	0	9.190291	-6.217339	-1.641229
236	1	0	10.045266	-4.729889	-1.153635
237	6	0	-7.937073	-4.311490	-3.429559
238	6	0	-7.649298	-3.217988	-4.472006
239	7	0	-6.386593	-2.662022	-3.994682
240	6	0	-5.748056	-3.570750	-3.177170
241	7	0	-6.647640	-4.557737	-2.857941
242	7	0	-8.907816	-3.663757	-2.556852
243	6	0	-9.479301	-2.582519	-3.190423
244	7	0	-8.793673	-2.367340	-4.358337
245	8	0	-4.572531	-3.531152	-2.835094
246	8	0	-10.455453	-1.947851	-2.805224
247	6	0	-5.610325	-1.723966	-4.774626
248	6	0	-9.134099	-1.343546	-5.301694
249	7	0	-6.068403	-0.361812	-4.726569
250	7	0	-8.452927	-0.092076	-5.111704
251	6	0	-7.104971	0.149866	-5.581266
252	6	0	-6.957609	1.685597	-5.452016
253	7	0	-6.005334	1.838377	-4.368009
254	6	0	-5.458855	0.628440	-3.994893
255	6	0	-9.150116	1.071252	-4.917054
256	7	0	-8.300857	2.115859	-5.167566
257	6	0	-5.317831	3.084936	-4.123181
258	6	0	-8.712246	3.486145	-5.066954
259	8	0	-10.338036	1.160274	-4.621778
260	8	0	-4.554026	0.469011	-3.183228
261	1	0	-7.541920	-3.589384	-5.496204
262	6	0	-6.359525	-5.651461	-1.970330
263	6	0	-9.655616	-4.409400	-1.574630
264	1	0	-5.603918	-2.037254	-5.825421
265	1	0	-4.591299	-1.753937	-4.386356
266	1	0	-10.203922	-1.147213	-5.220508
267	1	0	-8.914706	-1.728256	-6.303367
268	1	0	-6.982173	-0.225652	-6.602305
269	1	0	-6.582407	2.176671	-6.355337
270	7	0	-5.996066	3.968434	-3.206348
271	1	0	-5.170023	3.590808	-5.085852
272	1	0	-4.344578	2.855653	-3.685682
273	1	0	-9.798242	3.507082	-5.165801
274	7	0	-8.357846	4.152504	-3.845160
275	6	0	-9.119070	4.097631	-2.704732
276	6	0	-7.112710	4.816130	-3.613565
277	6	0	-7.382733	5.603357	-2.322867
278	1	0	-6.835877	5.434443	-4.473331
279	6	0	-5.317888	4.559002	-2.161152
280	8	0	-4.178394	4.295490	-1.799076
281	7	0	-6.128606	5.535480	-1.632247
282	1	0	-8.268565	4.048123	-5.893029

283	8	0	-10.193014	3.519240	-2.583990
284	7	0	-8.478573	4.840517	-1.740256
285	6	0	-9.195001	5.307121	-0.584982
286	6	0	-5.795697	6.335652	-0.481183
287	1	0	-7.679638	6.644955	-2.482947
288	7	0	-8.544321	4.991197	0.658804
289	1	0	-4.709614	6.423454	-0.429940
290	7	0	-6.244973	5.830418	0.791205
291	1	0	-6.226977	7.329217	-0.631622
292	6	0	-5.420607	5.177277	1.667634
293	6	0	-7.599388	5.904010	1.273885
294	1	0	-9.327091	6.394983	-0.649592
295	1	0	-7.970701	6.931791	1.208267
296	6	0	-7.490921	5.366974	2.721286
297	6	0	-9.171310	4.206114	1.597390
298	1	0	-10.174239	4.826442	-0.589009
299	8	0	-10.114032	3.447663	1.392867
300	7	0	-8.593896	4.453901	2.813125
301	8	0	-4.206442	5.043716	1.550925
302	7	0	-6.169042	4.765804	2.741184
303	6	0	-5.529636	4.307119	3.949334
304	6	0	-9.049713	3.849682	4.030562
305	1	0	-7.567495	6.140290	3.492552
306	1	0	-5.288953	-5.839748	-2.014560
307	1	0	-6.888126	-6.534291	-2.340353
308	7	0	-6.724715	-5.461501	-0.591549
309	7	0	-8.978257	-4.558030	-0.313246
310	1	0	-9.883739	-5.405196	-1.976496
311	1	0	-10.588651	-3.874382	-1.391682
312	6	0	-8.049286	-5.648269	-0.068671
313	6	0	-9.555225	-4.122328	0.856923
314	6	0	-5.853495	-5.016650	0.368401
315	1	0	-8.356150	-5.231364	-3.850234
316	8	0	-10.485637	-3.328964	0.953253
317	7	0	-8.941843	-4.768915	1.899600
318	8	0	-4.668086	-4.757668	0.207967
319	7	0	-6.528590	-4.957642	1.563555
320	6	0	-7.838923	-5.574402	1.458543
321	6	0	-5.802184	-4.881196	2.806238
322	6	0	-9.251716	-4.508750	3.278331
323	1	0	-7.845869	-6.546845	1.960449
324	1	0	-8.471606	-6.595847	-0.417565
325	1	0	-4.739126	-4.867435	2.562552
326	1	0	-6.019374	-5.768773	3.409679
327	7	0	-6.092460	-3.727214	3.608712
328	1	0	-10.307846	-4.243406	3.341715
329	7	0	-8.502357	-3.437926	3.888798
330	1	0	-9.081144	-5.434121	3.838695
331	6	0	-7.202667	-3.641336	4.509765

332	6	0	-5.453164	-2.522303	3.459013
333	1	0	-7.229761	-4.509919	5.175012
334	6	0	-6.959094	-2.299198	5.232068
335	6	0	-9.146561	-2.350332	4.429298
336	8	0	-4.525306	-2.295133	2.691640
337	7	0	-5.999670	-1.634145	4.361458
338	8	0	-10.314309	-2.029566	4.233600
339	7	0	-8.270250	-1.716533	5.271517
340	6	0	-5.244934	-0.481273	4.788960
341	1	0	-6.551141	-2.401186	6.242830
342	6	0	-8.618445	-0.549915	6.032196
343	1	0	-4.330498	-0.447162	4.194022
344	1	0	-4.983917	-0.601751	5.849235
345	7	0	-5.924171	0.779022	4.609829
346	1	0	-9.704083	-0.534091	6.133687
347	7	0	-8.210124	0.707844	5.472770
348	1	0	-8.166741	-0.640720	7.023950
349	6	0	-6.892191	1.258211	5.587992
350	6	0	-5.280248	1.839797	4.003590
351	1	0	-6.506430	1.134674	6.605165
352	6	0	-7.092346	2.721050	5.158728
353	6	0	-9.033412	1.504099	4.719200
354	8	0	-10.191934	1.250609	4.409813
355	7	0	-8.338962	2.653377	4.409524
356	8	0	-4.289528	1.771790	3.285963
357	7	0	-5.919264	2.993969	4.383428
358	1	0	-7.180947	3.426713	5.990879
359	1	0	-5.746321	5.003339	4.766621
360	1	0	-4.455224	4.298861	3.761220
361	1	0	-8.963663	4.595233	4.829303
362	1	0	-10.099043	3.580753	3.903414

Zero-point correction = 2.889543 (Hartree/Particle)
Thermal correction to Energy = 3.057477
Thermal correction to Enthalpy = 3.058421
Thermal correction to Gibbs Free Energy = 2.689814
Sum of electronic and zero-point Energies = -11300.694725
Sum of electronic and thermal Energies = -11300.526791
Sum of electronic and thermal Enthalpies = -11300.525847
Sum of electronic and thermal Free Energies = -11300.894454

Counterpoise corrected energy = -11302.998382715440
BSSE energy = 0.029234246889
sum of monomers = -11302.943778427740
complexation energy = -52.61 kcal/mole (raw)
complexation energy = -34.26 kcal/mole (corrected)

Table S9. Cartesian coordinates of [7]_2cucur_ecuatorial

Center Number	Atomic Number	Atomic Type	Coordinates (Angstroms) X	Y	Z
1	6	0	-0.729221	3.659955	-0.008109
2	6	0	0.371539	2.801003	-0.213411
3	6	0	0.196979	1.408561	-0.087621
4	6	0	-1.101749	0.888049	0.162377
5	6	0	-2.199889	1.758238	0.337382
6	6	0	-1.976515	3.151754	0.277922
7	6	0	1.324924	0.553104	-0.224455
8	6	0	-1.287474	-0.525530	0.250003
9	6	0	-0.160592	-1.381103	0.106388
10	6	0	1.137583	-0.861027	-0.144367
11	6	0	-0.337548	-2.774126	0.217886
12	6	0	-1.600868	-3.296484	0.564297
13	6	0	-2.698858	-2.468069	0.664764
14	6	0	-2.567149	-1.081238	0.465399
15	6	0	-3.720848	-0.200797	0.509353
16	6	0	-3.509618	1.194444	0.528246
17	1	0	-2.787923	3.849049	0.440914
18	1	0	-3.671628	-2.881456	0.896613
19	6	0	1.637863	3.326213	-0.547140
20	6	0	2.738667	2.497083	-0.628554
21	6	0	2.605827	1.109183	-0.433672
22	1	0	3.713948	2.909667	-0.852503
23	6	0	3.757406	0.224834	-0.485863
24	6	0	3.541304	-1.169701	-0.524112
25	6	0	2.232055	-1.733010	-0.332689
26	6	0	0.756980	-3.636272	-0.011302
27	6	0	2.004886	-3.127483	-0.293060
28	1	0	2.812976	-3.824314	-0.473424
29	6	0	-1.718958	-4.734283	0.860153
30	6	0	0.558786	-5.097753	0.015474
31	6	0	-0.549059	5.121448	-0.088992
32	6	0	1.761004	4.760229	-0.860901
33	7	0	0.686671	5.592891	-0.520658
34	7	0	-0.701692	-5.565830	0.387102
35	8	0	2.759478	5.219762	-1.403770
36	8	0	-1.458683	5.895778	0.193488
37	8	0	-2.665813	-5.195352	1.484859
38	8	0	1.462528	-5.877821	-0.272966
39	6	0	-0.910770	-7.029522	0.389580
40	6	0	-0.871587	-7.638364	1.788587
41	6	0	-2.165486	-7.449643	-0.373542
42	1	0	-0.051972	-7.418972	-0.162332
43	6	0	-1.038900	-9.153671	1.685978
44	1	0	-1.677716	-7.219843	2.398937

45	1	0	0.080879	-7.393017	2.272808
46	6	0	-2.256822	-8.975267	-0.375001
47	1	0	-3.064099	-7.037565	0.094488
48	1	0	-2.122845	-7.069959	-1.401079
49	1	0	-1.060861	-9.591343	2.688785
50	1	0	-0.167830	-9.580950	1.171676
51	1	0	-3.170894	-9.287041	-0.889832
52	1	0	-1.414095	-9.392028	-0.942245
53	6	0	0.882841	7.048316	-0.702806
54	6	0	-0.019456	7.649458	-1.777146
55	6	0	0.794875	7.828339	0.606577
56	1	0	1.910815	7.132082	-1.061226
57	6	0	0.314479	9.132436	-1.937080
58	1	0	-1.071236	7.541692	-1.495881
59	1	0	0.130688	7.123340	-2.726990
60	6	0	1.091391	9.301724	0.328362
61	1	0	-0.204389	7.733606	1.041054
62	1	0	1.515971	7.427351	1.328338
63	1	0	-0.355639	9.583127	-2.675676
64	1	0	1.334944	9.234720	-2.329506
65	1	0	0.993527	9.877847	1.253666
66	1	0	2.133078	9.406584	-0.002965
67	7	0	-2.236041	-9.576337	0.958703
68	1	0	-3.038772	-9.204035	1.464908
69	7	0	0.236282	9.902344	-0.695527
70	1	0	-0.723835	9.830765	-0.360727
71	6	0	-4.674737	1.966778	0.716167
72	6	0	-5.986067	0.110630	0.632111
73	1	0	-4.611311	3.043098	0.816630
74	7	0	-5.883576	1.459646	0.782566
75	7	0	-4.949942	-0.731759	0.545119
76	6	0	-7.508241	-1.571237	-0.254370
77	6	0	-8.418559	0.434805	0.757553
78	6	0	-8.038715	-1.079595	-1.599163
79	1	0	-8.260567	-2.199836	0.233551
80	1	0	-6.591987	-2.151243	-0.373252
81	6	0	-8.972414	0.878401	-0.589383
82	1	0	-9.178127	-0.163947	1.277633
83	1	0	-8.168554	1.290451	1.378740
84	1	0	-8.258164	-1.929318	-2.245681
85	1	0	-7.250524	-0.492773	-2.082315
86	1	0	-9.893243	1.447621	-0.431825
87	1	0	-8.251412	1.549062	-1.074209
88	7	0	-7.241743	-0.412842	0.593849
89	7	0	-9.232566	-0.242932	-1.490737
90	1	0	-9.955682	-0.822008	-1.062117
91	6	0	4.701071	-1.943386	-0.738003
92	6	0	6.020889	-0.095805	-0.622817
93	1	0	4.631704	-3.016818	-0.863140

94	7	0	4.988794	0.750269	-0.517913
95	7	0	5.911443	-1.440676	-0.802847
96	6	0	8.451469	-0.428330	-0.771591
97	6	0	7.557437	1.558968	0.291755
98	6	0	9.021751	-0.899941	0.558822
99	1	0	9.205802	0.179200	-1.289385
100	1	0	8.191051	-1.270323	-1.406958
101	6	0	8.105059	1.038986	1.619346
102	1	0	8.303560	2.197413	-0.193055
103	1	0	6.642894	2.136362	0.434134
104	1	0	9.939298	-1.467505	0.377723
105	1	0	8.305735	-1.578710	1.039726
106	1	0	8.334183	1.874501	2.280768
107	1	0	7.322470	0.443148	2.100446
108	7	0	7.279246	0.418614	-0.575872
109	7	0	9.295960	0.203020	1.478474
110	1	0	10.014471	0.789272	1.051736
111	6	0	6.213737	-1.956741	5.327727
112	6	0	6.475926	-3.364767	4.759882
113	7	0	7.846990	-3.268459	4.284215
114	6	0	8.479251	-2.168044	4.824611
115	7	0	7.533682	-1.410844	5.464386
116	7	0	5.390871	-1.342219	4.298201
117	6	0	4.888568	-2.294018	3.435270
118	7	0	5.472545	-3.496968	3.743635
119	8	0	9.678855	-1.925437	4.769373
120	8	0	4.041914	-2.111175	2.568419
121	6	0	8.607489	-4.455385	3.972687
122	6	0	5.226776	-4.705390	3.008287
123	7	0	8.490254	-4.902982	2.615971
124	7	0	6.138802	-4.947017	1.913231
125	6	0	7.390016	-5.666983	2.101822
126	6	0	7.827879	-5.973279	0.652657
127	7	0	8.916955	-5.040574	0.443026
128	6	0	9.346203	-4.492332	1.627337
129	6	0	5.645632	-5.167391	0.647508
130	7	0	6.636821	-5.721807	-0.114446
131	6	0	9.801900	-5.159868	-0.682392
132	6	0	6.459864	-6.070507	-1.502079
133	8	0	4.498780	-4.941940	0.277947
134	8	0	10.341872	-3.794158	1.785603
135	1	0	6.386502	-4.168933	5.497501
136	6	0	7.852473	-0.251491	6.250275
137	6	0	4.654224	-0.130240	4.561479
138	1	0	8.287032	-5.271913	4.628533
139	1	0	9.658698	-4.236311	4.163021
140	1	0	4.221499	-4.646050	2.588764
141	1	0	5.273860	-5.542940	3.712429
142	1	0	7.220821	-6.558187	2.714488

143	1	0	8.171016	-7.001655	0.498896
144	7	0	9.320852	-4.496266	-1.864499
145	1	0	9.960159	-6.222776	-0.905471
146	1	0	10.754156	-4.703511	-0.407995
147	1	0	5.387081	-6.112853	-1.694514
148	7	0	7.043850	-5.164175	-2.456621
149	6	0	6.349877	-4.159665	-3.085511
150	6	0	8.434706	-5.156044	-2.810603
151	6	0	8.482948	-4.207776	-4.024369
152	1	0	8.785666	-6.171302	-3.021084
153	6	0	10.100264	-3.553580	-2.490565
154	8	0	11.027497	-2.927854	-1.987540
155	7	0	9.687931	-3.467560	-3.795364
156	1	0	6.894623	-7.060481	-1.666115
157	8	0	5.147326	-3.945656	-2.982690
158	7	0	7.232350	-3.467798	-3.887888
159	6	0	6.751584	-2.554763	-4.902140
160	6	0	10.259030	-2.530183	-4.719143
161	1	0	8.519727	-4.716834	-4.992240
162	7	0	7.271902	-1.214893	-4.814751
163	1	0	11.325794	-2.451294	-4.505331
164	7	0	9.704402	-1.208849	-4.662206
165	1	0	10.125453	-2.931060	-5.728324
166	6	0	10.411176	-0.141049	-4.174696
167	6	0	8.528163	-0.812283	-5.396148
168	1	0	7.000747	-2.952179	-5.892597
169	1	0	8.593921	-1.157450	-6.432930
170	6	0	8.520124	0.729164	-5.242236
171	6	0	6.557272	-0.152250	-4.320042
172	1	0	5.666640	-2.500727	-4.799499
173	8	0	5.431078	-0.206272	-3.838688
174	7	0	7.307483	0.990675	-4.496263
175	8	0	11.478880	-0.193802	-3.572969
176	7	0	9.742197	0.998573	-4.530639
177	6	0	10.309812	2.305715	-4.365714
178	6	0	6.718672	2.306090	-4.452638
179	1	0	8.503862	1.270776	-6.193649
180	1	0	8.920916	-0.280901	6.466847
181	1	0	7.293322	-0.310738	7.188987
182	7	0	7.564482	1.014721	5.638502
183	7	0	5.432564	1.079318	4.447535
184	1	0	4.225759	-0.182846	5.571636
185	1	0	3.846582	-0.072264	3.829728
186	6	0	6.250099	1.581368	5.544063
187	6	0	4.992568	2.110091	3.645233
188	6	0	8.519261	1.811052	5.059645
189	1	0	5.691306	-1.951387	6.290100
190	8	0	4.202188	2.015570	2.713288
191	7	0	5.548652	3.275977	4.110155

192	8	0	9.711165	1.544550	4.956592
193	7	0	7.911806	2.981252	4.665243
194	6	0	6.532439	3.040133	5.124749
195	6	0	8.695754	4.158500	4.372354
196	6	0	5.358844	4.546406	3.468944
197	1	0	6.433112	3.767902	5.936212
198	1	0	5.716186	1.484605	6.494909
199	1	0	9.740184	3.912050	4.568516
200	1	0	8.388070	4.967922	5.042548
201	7	0	8.604997	4.648004	3.022680
202	1	0	4.343494	4.575411	3.071219
203	7	0	6.258729	4.822601	2.381283
204	1	0	5.467077	5.324627	4.230731
205	6	0	7.536804	5.482207	2.538001
206	6	0	9.435049	4.228597	2.014031
207	1	0	7.433261	6.374278	3.162992
208	6	0	7.951751	5.777885	1.072990
209	6	0	5.796111	4.889605	1.093182
210	8	0	10.388689	3.465930	2.138371
211	7	0	9.045536	4.852459	0.855527
212	8	0	4.692882	4.516771	0.715549
213	7	0	6.753350	5.498271	0.325748
214	6	0	9.903166	4.912598	-0.296872
215	1	0	8.280894	6.807142	0.899446
216	6	0	6.552194	5.812199	-1.063847
217	1	0	10.851898	4.445260	-0.027235
218	1	0	10.077925	5.965955	-0.550553
219	7	0	9.397602	4.231225	-1.462589
220	1	0	5.476551	5.860760	-1.237441
221	7	0	7.110555	4.887723	-2.013820
222	1	0	6.990327	6.795453	-1.256977
223	6	0	8.490561	4.873641	-2.400882
224	6	0	10.140795	3.249785	-2.079592
225	1	0	8.837204	5.884622	-2.638343
226	6	0	8.505249	3.900919	-3.596698
227	6	0	6.410303	3.850085	-2.576677
228	8	0	5.225908	3.598832	-2.392039
229	7	0	7.254224	3.173780	-3.428852
230	8	0	11.064817	2.617776	-1.578168
231	7	0	9.690742	3.132181	-3.369662
232	1	0	8.539437	4.390873	-4.574827
233	1	0	10.250528	2.838727	-5.320138
234	1	0	11.358080	2.173654	-4.095083
235	1	0	6.851962	2.781413	-5.432888
236	1	0	5.652813	2.189688	-4.251426
237	6	0	-8.392119	5.185390	2.737118
238	6	0	-8.430067	4.249481	3.960891
239	7	0	-7.173000	3.519808	3.830566
240	6	0	-6.297942	4.210329	3.019755

241	7	0	-7.001931	5.201475	2.380630
242	7	0	-9.275326	4.508985	1.800026
243	6	0	-10.045887	3.566699	2.437638
244	7	0	-9.627715	3.495103	3.741297
245	8	0	-5.093561	4.006351	2.917255
246	8	0	-10.970894	2.930311	1.944001
247	6	0	-6.686225	2.614186	4.848062
248	6	0	-10.197740	2.574065	4.682074
249	7	0	-7.206672	1.273557	4.770038
250	7	0	-9.642094	1.252765	4.651876
251	6	0	-8.454051	0.873461	5.374280
252	6	0	-8.440622	-0.670071	5.242805
253	7	0	-7.218236	-0.940202	4.519122
254	6	0	-6.478055	0.203029	4.312594
255	6	0	-10.334732	0.177197	4.162223
256	7	0	-9.650575	-0.954624	4.515551
257	6	0	-6.631780	-2.255996	4.490363
258	6	0	-10.221828	-2.265204	4.392878
259	8	0	-11.402867	0.217286	3.560203
260	8	0	-5.349647	0.253457	3.835837
261	1	0	-8.470760	4.768293	4.923454
262	6	0	-6.427545	6.100855	1.413905
263	6	0	-9.761421	5.154000	0.609650
264	1	0	-6.930631	3.017495	5.837449
265	1	0	-5.601785	2.560267	4.739741
266	1	0	-11.264322	2.489275	4.469581
267	1	0	-10.064991	2.994176	5.683491
268	1	0	-8.506890	1.233772	6.406674
269	1	0	-8.438259	-1.197957	6.202161
270	7	0	-7.167476	-3.135905	3.475804
271	1	0	-6.766744	-2.720020	5.475765
272	1	0	-5.565673	-2.142876	4.288633
273	1	0	-11.270732	-2.140434	4.121323
274	7	0	-9.607049	-3.121077	3.419925
275	6	0	-10.055155	-3.262919	2.131499
276	6	0	-8.412140	-3.871841	3.658358
277	6	0	-8.390182	-4.865616	2.479748
278	1	0	-8.439255	-4.344725	4.645062
279	6	0	-6.317494	-3.824261	2.638375
280	8	0	-5.132934	-3.571837	2.456299
281	7	0	-7.011204	-4.873141	2.088767
282	1	0	-10.160273	-2.769556	5.362765
283	8	0	-10.983238	-2.645766	1.619130
284	7	0	-9.305204	-4.249501	1.532241
285	6	0	-9.802086	-4.953502	0.376671
286	6	0	-6.446033	-5.812465	1.156732
287	1	0	-8.726043	-5.875707	2.736024
288	7	0	-8.943775	-4.900042	-0.775773
289	1	0	-5.370593	-5.851793	1.334065

290	7	0	-6.646276	-5.525074	-0.238802
291	1	0	-6.879087	-6.794354	1.367371
292	6	0	-5.691632	-4.924160	-1.015399
293	6	0	-7.843624	-5.820313	-0.982303
294	1	0	-9.964486	-6.005058	0.645438
295	1	0	-8.166014	-6.849583	-0.796759
296	6	0	-7.431182	-5.539427	-2.450948
297	6	0	-9.338105	-4.293372	-1.941937
298	1	0	-10.756083	-4.501558	0.099595
299	8	0	-10.297781	-3.539902	-2.075355
300	7	0	-8.504724	-4.718334	-2.945526
301	8	0	-4.587521	-4.546703	-0.645022
302	7	0	-6.157370	-4.869680	-2.302708
303	6	0	-5.260388	-4.599933	-3.394599
304	6	0	-8.600581	-4.247825	-4.301720
305	1	0	-7.321765	-6.438364	-3.065036
306	1	0	-5.355182	6.156941	1.605244
307	1	0	-6.872536	7.088363	1.564598
308	7	0	-6.601428	5.731179	0.031235
309	7	0	-8.877218	5.023969	-0.515214
310	1	0	-9.925085	6.218897	0.818637
311	1	0	-10.711605	4.688822	0.342819
312	6	0	-7.794314	5.960374	-0.739976
313	6	0	-9.304159	4.455756	-1.691028
314	6	0	-5.606701	5.169743	-0.720977
315	1	0	-8.750721	6.199909	2.938066
316	8	0	-10.297155	3.751540	-1.838826
317	7	0	-8.449423	4.853979	-2.685744
318	8	0	-4.457891	4.960363	-0.348201
319	7	0	-6.098966	4.921766	-1.981799
320	6	0	-7.353769	5.632245	-2.183525
321	6	0	-5.185352	4.663373	-3.071916
322	6	0	-8.564787	4.385762	-4.035879
323	1	0	-7.188452	6.514008	-2.810756
324	1	0	-8.144817	6.988794	-0.604177
325	1	0	-4.180355	4.614268	-2.650385
326	1	0	-5.233921	5.488100	-3.790984
327	7	0	-5.427808	3.441280	-3.785150
328	1	0	-9.615101	4.159891	-4.223178
329	7	0	-7.800024	3.197036	-4.329253
330	1	0	-8.247206	5.193682	-4.703666
331	6	0	-6.427820	3.288701	-4.801992
332	6	0	-4.837395	2.246792	-3.457231
333	1	0	-6.337964	4.080185	-5.553219
334	6	0	-6.159918	1.871375	-5.344272
335	6	0	-8.427424	2.084547	-4.849975
336	8	0	-3.989854	2.083039	-2.587408
337	7	0	-5.334489	1.278161	-4.304302
338	8	0	-9.626682	1.840261	-4.794571

339	7	0	-7.477472	1.317237	-5.471223
340	6	0	-4.589711	0.066989	-4.548372
341	1	0	-5.637449	1.851003	-6.306441
342	6	0	-7.789757	0.143409	-6.238393
343	1	0	-3.782457	0.025482	-3.815092
344	1	0	-4.160475	0.107260	-5.558805
345	7	0	-5.359706	-1.146241	-4.417519
346	1	0	-8.858689	0.162406	-6.453674
347	7	0	-7.492415	-1.111430	-5.607637
348	1	0	-7.232365	0.191655	-7.178872
349	6	0	-6.174025	-1.668018	-5.507051
350	6	0	-4.913521	-2.163110	-3.600939
351	1	0	-5.641391	-1.579514	-6.459406
352	6	0	-6.445752	-3.123497	-5.069124
353	6	0	-8.441697	-1.908149	-5.020270
354	8	0	-9.635690	-1.649637	-4.921551
355	7	0	-7.825736	-3.068859	-4.611222
356	8	0	-4.125601	-2.050918	-2.668955
357	7	0	-5.460804	-3.339042	-4.051075
358	1	0	-6.340474	-3.860801	-5.871146
359	1	0	-5.362978	-5.388313	-4.146698
360	1	0	-4.244643	-4.616646	-2.996991
361	1	0	-8.287435	-5.063797	-4.961359
362	1	0	-9.647021	-4.011837	-4.500071

Zero-point correction = 2.888145 (Hartree/Particle)
Thermal correction to Energy = 3.055339
Thermal correction to Enthalpy= 3.056283
Thermal correction to Gibbs Free Energy = 2.685889
Sum of electronic and zero-point Energies = -11300.656028
Sum of electronic and thermal Energies = -11300.488835
Sum of electronic and thermal Enthalpies = -11300.487890
Sum of electronic and thermal Free Energies = -11300.858285

Counterpoise corrected energy = -11302.961567138690
BSSE energy = 0.022805262786
sum of monomers = -11302.916380474630
complexation energy = -42.67 kcal/mole (raw)
complexation energy = -28.36 kcal/mole (corrected)

(1) Frisch, M. J.; Trucks, G. W.; Schlegel, H. B.; Scuseria, G. E.; Robb, M. A.; Cheeseman, J. R.; Scalmani, G.; Barone, V.; Mennucci, B.; Petersson, G. A.; et al. Gaussian 09. Gaussian, Inc.: Wallingford CT 2013.

(2) Calbo, J.; Ortí, E.; Sancho-García, J. C.; Aragó, J. The Nonlocal Correlation Density Functional VV10. In *Annual Reports in Computational Chemistry*, **2015**, pp 37–102.

(3) Calbo, J.; Ortí, E.; Sancho-García, J. C.; Aragó, J. Accurate Treatment of Large Supramolecular Complexes by Double-Hybrid Density Functionals Coupled with Nonlocal van Der Waals Corrections. *J. Chem. Theory Comput.* **2015**, *11* (3), 932–939.

(4) Grimme, S.; Hansen, A.; Brandenburg, J. G.; Bannwarth, C. Dispersion-Corrected Mean-Field Electronic Structure Methods. *Chem. Rev.* **2016**, *116* (9), 5105–5154.

(5) Adamo, C.; Barone, V. Toward Reliable Density Functional Methods without Adjustable Parameters: The PBE0 Model. *J. Chem. Phys.* **1999**, *110* (13), 6158–6170.

(6) Grimme, S.; Antony, J.; Ehrlich, S.; Krieg, H. A Consistent and Accurate Ab Initio Parametrization of Density Functional Dispersion Correction (DFT-D) for the 94 Elements H-Pu. *J. Chem. Phys.* **2010**, *132* (15), 154104.

(7) Francl, M. M.; Pietro, W. J.; Hehre, W. J.; Binkley, J. S.; Gordon, M. S.; DeFrees, D. J.; Pople, J. A. Self-Consistent Molecular Orbital Methods. XXIII. A Polarization-Type Basis Set for Second-Row Elements. *J. Chem. Phys.* **1982**, *77* (7), 3654–3665.

(8) Hariharan, P. C.; Pople, J. A. The Influence of Polarization Functions on Molecular Orbital Hydrogenation Energies. *Theor. Chim. Acta* **1973**, *28* (3), 213–222.

(9) Simon, S.; Duran, M.; Dannenberg, J. J. How Does Basis Set Superposition Error Change the Potential Surfaces for Hydrogen‐bonded Dimers? *J. Chem. Phys.* **1996**, *105* (24), 11024‐11031.

(10) Boys, S. F.; Bernardi, F. The Calculation of Small Molecular Interactions by the Differences of Separate Total Energies. Some Procedures with Reduced Errors. *Mol. Phys.* **1970**, *19* (4), 553–566.

Binding constants from fluorescence titration experiments.

Determining binding constants from fluorescence titration experiments was performed with Supramolecular tools.[11-13]

(11) http://supramolecular.org/, accessed 26/07/2021.

(12) Thordarson, P. Determining association constants from titration experiments in supramolecular chemistry. *Chem. Soc. Rev.* **2011**, *40*, 1305-1323.

(13) Hibbert, D. B.; Thordarson, P. The death of the Job plot, transparency, open science and online tools, uncertainty estimation methods and other developments in supramolecular chemistry data analysis. *Chem. Commun.* **2016**, *52*, 12792-12805.

The use of R program.

Determining LODs within the values measured was performed following previous report.[14]

(14) Ortiz, M. C.; Sarabia, L. A.; Sánchez, M. S. Tutorial on evaluation of type I and type II errors in chemical analyses: From the analytical detection to authentication of products and process control. *Anal. Chim. Acta.* **2010**, *674*, 123-142.

Solubility of TNT or TNB in water.

The solubility of of TNT in water was taken from previous report.[15]

(15) Prak, D. J. L.; O'Sullivan, D. W. Solubility of 4-Nitrotoluene, 2,6-Dinitrotoluene, 2,3-Dinitrotoluene, and 1,3,5-Trinitrobenzene in Pure Water and Seawater. *J. Chem. Eng. Data* **2007**, *52*, 2446-2450.

The solubility of of TNB in water was taken from previous report.[16]

(16) Prak, D. J. L.; O'Sullivan, D. W. Solubility of 2,4-Dinitrotoluene and 2,4,6-Trinitrotoluene in Seawater. *J. Chem. Eng. Data* **2006**, *51*, 448-450.
